# A Signature of Three Apoptosis-Related Genes Predicts Overall Survival in Breast Cancer

**DOI:** 10.3389/fsurg.2022.863035

**Published:** 2022-05-17

**Authors:** Rongyang Zou, Wanjun Zhao, Shuguang Xiao, Yaxing Lu

**Affiliations:** Department of Thyroid and Breast Surgery, The First Affiliated Hospital of Wannan Medical College, Wuhu, China

**Keywords:** apoptosis-related gene, breast cancer, TCGA, prognostic signature, overall survival

## Abstract

**Background:**

The commonest malignancy in women is known as breast cancer (BC). Numerous studies demonstrated that apoptosis appears to be critical to the management and clinical outcome of BC patients. The purpose of this study is to explore the potential connection between apoptosis and BC and establish the apoptosis-associated gene signature in BC.

**Methods:**

The data of BC patient transcripts and related clinical information comes from the Cancer Genome Atlas Database (TCGA), and the genes related to apoptosis come from the Molecular Characterization Database (MSigDB). We identified the abnormally expressed apoptosis-related genes in BC samples. The optimal apoptosis-related genes screened by Cox regression analysis were designed to construct a prognostic model for predicting BC patients. Using the Nom Chart to Predict 1-Year, 3-Year, and 5-Year overall survival for BC patients. The gene signature-related functional pathways were explored by gene set enrichment analysis (GSEA).

**Results:**

Three genes [alpha subunit of the interleukin 3 receptor (*IL3RA*), apoptosis-inducing factor mitochondrial-associated 1 (*AIFM1*)*,* and phosphatidylinositol-3 kinase catalytic alpha (*PIK3CA*)] correlated with apoptosis were shown to be strongly linked to the overall survival of BC. Survival analysis shows that the risk score is directly proportional to the poor prognosis of BC patients. Risk assessment based on three genetic characteristics (age, pathological stage N, and pathological stage M) can independently predict the prognosis of patients with BC. The Nom chart is most suitable for assessing the long-term survival rate of BC patients. The results of GSEA demonstrated that numerous cell cycle-related pathways were abundant in the high-risk group.

**Conclusion:**

We constructed an apoptosis-associated gene signature in BC, which had a potential clinical application prospect for BC patients.

## Introduction

Breast cancer (BC) is more common in women than lung cancer and is the most common cancer. In 2022, Cancer statistics published by ACS showed BC is expected to be the most common cancer in the USA, and China’s cancer profile is becoming similar to that of the USA (1). There are many subtypes of breast cancer, and the prognosis largely depends on the diagnostic cycle and accuracy. Due to environmental, genetic, and other factors that can cause cancer, the incidence of breast cancer has steadily increased, and metastasis has become more serious after treatment, most breast cancer deaths are metastatic (2). Although local surgery, conventional chemotherapy, precision radiation therapy, endocrine therapy, and the use of monoclonal antibodies have significantly improved the prognosis of BC patients, there are still large numbers of patients at risk of relapse and death. It is currently widely believed that the prognosis of BC depends on clinical, pathological, and molecular features. In fact, these clinical and pathological features cannot accurately predict the survival of patients, leading to overtreatment of some low-risk patients, while high-risk patients face the risk of recurrence or metastasis due to inadequate treatment. Therefore, the identification of new prognostic markers can provide new insights for the early detection of BC and the reduction of mortality and relapse rates (3).

Apoptosis is also known as programmed cell death. It encompasses many biological and pathological processes, such as embryonic development, tissue and organ balance, tumor development, etc. Inhibition of apoptosis can lead to cancer, such as anti-apoptosis protein Bcl-2, which drives the development of B-cell lymphoma (4, 5). At the same time, recent studies have shown that low cell apoptosis can lead to tumor deterioration (6–8). Studies have shown that the occurrence of tumors is closely related to the imbalance of cell apoptosis and proliferation. Apoptosis is a common self-discovery phenomenon in malignant tumors. Several genes have been found to regulate apoptosis. *p53* is a tumor suppressor gene. Wild-type *p53* can trigger apoptosis. Mutations or deletions in *p53* are very common in breast cancer. The overexpression of Bcl-2 can inhibit apoptosis. Bcl-x and *Bax-α* are related genes of Bcl-2. *Bax-α* promotes apoptosis, while Bcl-XL inhibits apoptosis. Bargou et al. found that the expression of Bax-α in normal epithelial and breast cancer cells was higher than that in malignant ones (9). Under certain apoptosis-inducing conditions, normal breast epithelial cell lines are more prone to apoptosis than breast cancer cell lines. Therefore, abnormal apoptosis can be one of the causes of breast cancer. This is manifested in the imbalance between anti-apoptotic genes such as Bcl-2, Bcl-x, and pro-apoptotic gene Bax (10). In addition, experimental studies have shown that antiestrogen therapy can induce apoptosis, suggesting that apoptosis plays an important role in regulating tumor growth and that apoptosis may be related to the prognosis of BC. However, previous studies have focused on the association between one or more genes involved in apoptosis and BC, and did not systematically clarify the function and prognostic value of genes related to apoptosis in BC.

This study will provide novel prognostic markers for BC based on apoptosis-related genes through bioinformatics analysis. Briefly, we downloaded transcriptional data and clinical information from the TCGA database, and then constructed and verified the gene signature related to apoptosis. Finally, we analyzed the GSEA enrichment and explored the potential regulatory mechanism, which provides a new perspective for breast cancer research (11).

## Results

### Identification of Apoptosis-Related Differentially Expressed Genes (DEGs) in BC

**Figure 1:**
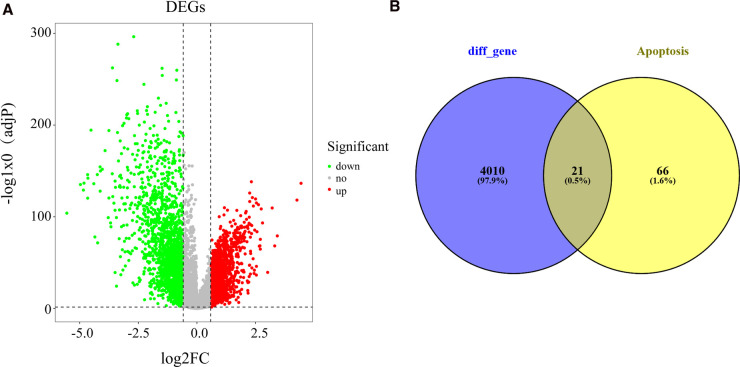
Recognition of differentially expressed apoptosis-related genes in BC and normal samples (**A**) Volcano plot exhibiting expression data of BC and normal samples in the TCGA database. The x-axis presents the mean differences between BC and normal samples. The y-axis presents the log-transformed adj. P-values. The red spots represent significantly upregulated genes, and the green spots represent significantly downregulated genes. (**B**) Venn plot showing the common genes shared by DEGs and apoptosis-related genes. BC, Breast cancer; DEGs, differentially expressed genes; TCGA, The Cancer Genome Atlas.

In view of screening criteria, we obtained 4,031 DEGs between BC samples and normal samples in the TCGA database, including 1937 up-regulated and 2094 down-regulated genes (Supplementary Tabl S2). The distribution of DEGs was displayed as Figure 1A. Subsequently, we used the Venn diagram to demonstrate the intersections between these DEGs and apoptosis-related genes. As shown in Figure 1B and Supplementary Table S3, a total of 21 apoptosis-related DEGs were identified.

### Construction of Apoptosis-Related Signature for Patients with BC

**Figure 2:**
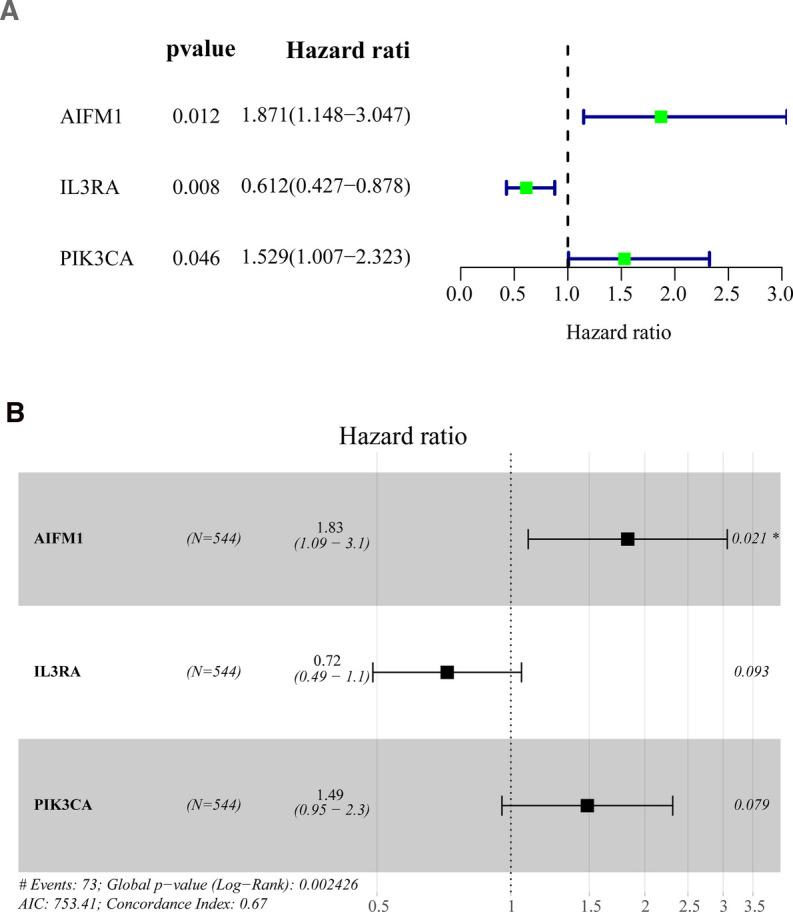
Identification of prognostic genes based on the differentially expressed apoptosis-related genes (**A**) Univariate Cox regression analysis was performed to identify the factors that were associated with the overall survival of BC patients in the TCGA database. (**B**) Multivariate Cox regression analysis to screen out the key variables most relevant to prognosis. BC, Breast cancer; OS, overall survival; TCGA, The Cancer Genome Atlas. (*p < 0.01).

To explore the prognostic value of apoptosis-related DEGs in BC, we first performed a univariate Cox regression analysis on the training set. The results showed that the 3 apoptosis-related DEGs were significantly related to the overall survival of BC (Figure 2A and Supplementary Table S4). Afterward, stepwise multivariate Cox regression analysis was used to establish a prognostic model for BC, which was composed of *IL3RA*, *AIFM1*, and *PIK3CA* (Figure 2B). Among them, *AIFM1* and *PIK3CA* were considered as risk genes (HR > 1), while *IL3RA* was considered as a protective gene (HR < 1). The risk score is calculated as follows: 0.6049*expression level of *AIFM1* + −0.3297* expression level of *IL3RA* + 0.3959* expression level of *PIK3CA*.

### Validation of the Apoptosis-Related Gene Signature in BC

**Figure 3:**
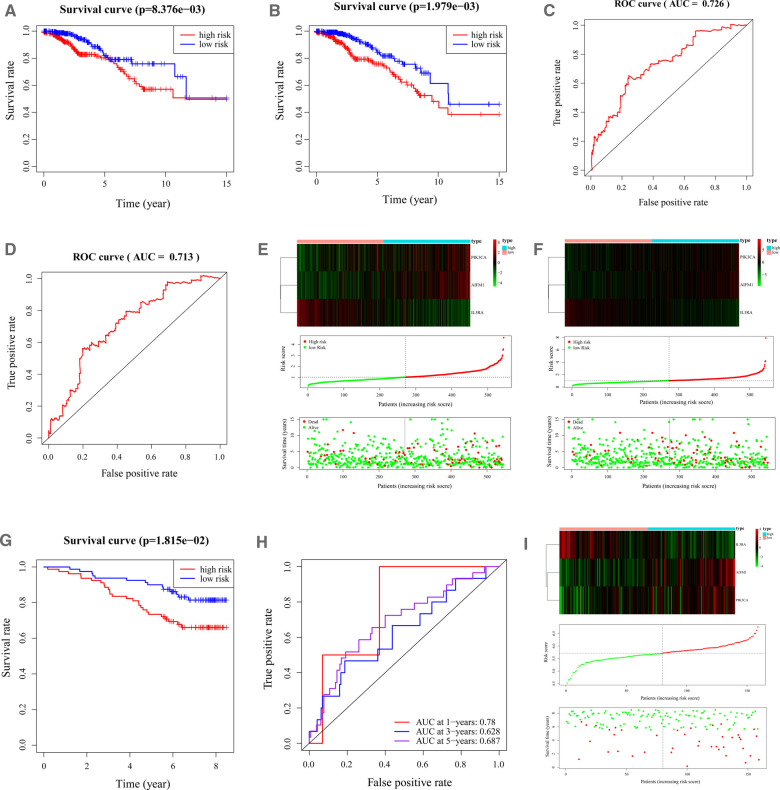
Characteristics of the 3-apoptosis-related gene-based signature. (**A,B**). Kaplan–Meier survival curves for the low-risk and high-risk groups in the training (**A**) and validation (**B**) sets. Overall survival time is recorded in years. (**C, D**). ROC curves of 5-year survival prediction in the training (**C**) and validation (**D**) sets. (**E**). Heatmap of the three apoptosis-related genes expression in BC patients (top). The risk score distribution of BC patients in the TCGA-training database (middle). Survival status and duration of patients (bottom). (**F**). The heatmap of mRNA expression and risk score of the three-gene signature in the validation set of the TCGA cohort. BC, Breast cancer; ROC, receiver operating characteristic; TCGA, The Cancer Genome Atlas.

The BC samples in both the training and validation sets were categorized into high-low risk groups depending on the median risk score in the corresponding cohort, respectively. Survival analysis demonstrated that there was a remarkable difference in survival rate between high- and low-risk groups in the training and validation sets (*p* < 0.01, Figures 3A, B). As shown in Figures 3C,D, the area under the curve (AUC) of the ROC curve was 0.726 (training set) and 0.713 (validation set), demonstrating that apoptosis-related gene signature performed well as a predictor of prognosis in BC. In addition, we had also drawn the risk plot of the training set (Figure 3E) and validation set (Figure 3F), including risk scores for BC patients, the survival status of the BC patients, and the expression levels of the three gene signatures. The above results indicated that the prognostic signature based on three apoptosis-related genes had convincing predictive performance and stability. Moreover, consistent results were found in the external validation set GSE1456 (Figures 3G–I). In addition, we also found that Age, T, and Fustat differ significantly between the high-risk group and the low-risk group (Supplementary Figure S1).

### Independent Prediction Capacity of the Apoptosis-Related Gene Signature

To determine whether apoptosis-related gene markers are independent prognostic factors for BC, we performed a univariate and multivariate Cox analysis in the training set. According to the results of the univariate Cox analysis, it was revealed that that the age (HR = 1.0433, *p* < 0.00001), T (HR = 1.6989, *p* = 0.0005), N (HR = 1.6649, *p* = 0.0002), M (HR = 19.9195, *p* < 0.00001), stage (HR = 2.4367, *p* < 0.00001), and risk score (HR = 1.9976, *p* = 0.0002) were associated with the overall survival of BC patients. Ultimately, age (HR = 1.0491, *p* < 0.00001), M (HR = 15.7822, *p* = 0.0005), N (HR = 1.6466, *p* = 0.0348), and risk score (HR = 1.9868, *p* = 0.0005) were independent parameters associated with patient overall survival as further confirmed by multivariate Cox analysis. The specific results are shown in Tables 1, 2.

**Table 1:** Univariable Cox regression analysis.

Id	HR	HR.95L	HR.95H	*p*-Value
Age	1.043325	1.02254	1.064532	3.61E-05
Race	0.90973	0.698911	1.18414	0.481821
M	19.91949	7.001549	56.67117	2.05E-08
N	1.664874	1.269851	2.182779	0.000225
Stage	2.436679	1.677736	3.538938	2.90E-06
T	1.698875	1.258666	2.293045	0.000533
RiskScore	1.997624	1.395869	2.858794	0.000155

**Table 2:** Multivariable Cox regression analysis.

Id	HR	HR.95L	HR.95H	*p*-Value
Age	1.049078	1.0275	1.071108	6.23E-06
Race	0.863768	0.650614	1.146756	0.311117
M	15.78223	3.333363	74.72299	0.000506
N	1.646628	1.036167	2.616743	0.034833
Stage	0.937947	0.395253	2.225773	0.884477
T	1.569311	0.977603	2.519158	0.062019
RiskScore	1.98684	1.347129	2.93033	0.000534

### Construction of a Nomogram for Clinical Practice

**Figure 4:**
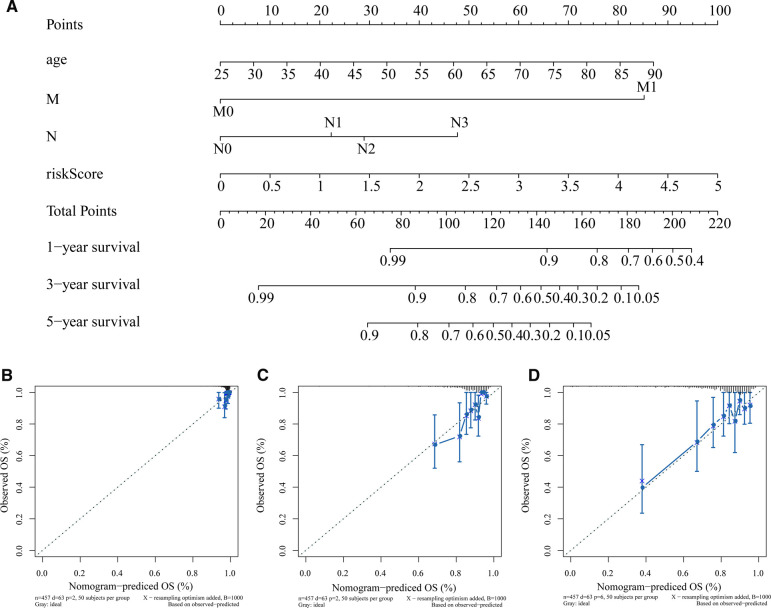
Construction and validation of nomogram. (**A**) Nomogram constructed based on age, M, N, and risk score as predictive factors to predict 1-, 3-, 5-year survival probability. For each patient, four lines are drawn upward to determine the points received from the three predictors in the nomogram. The sum of these points is located on the “Total Points” axis. Then a line is drawn downward to determine the possibility of 1-, 3-, and 5-year overall survival of BC. **(B–D)** Calibration curves for the survival probability at 1 (**B**), 3 (**C**), and 5 (**D**) years. The Y-axis represents actual survival, and the X-axis represents nomogram-predicted survival. BC, Breast cancer.

In the training set, we used 4 independent prognostic factors (including age, M, N, and risk score) to construct a nomogram model to predict BC 1-, 3-, and 5-year overall survival (Figure 4A). Figures 4B–D show the calibration curve of the nomogram and the ideal model 1-, 3-, and 5-year survival rates. The results indicated that the nomogram was more conducive to predict the long-term survival of BC patients.

### GSEA Analysis for Phenotype Differences between the High- and Low-Risk Groups

**Figure 5:**
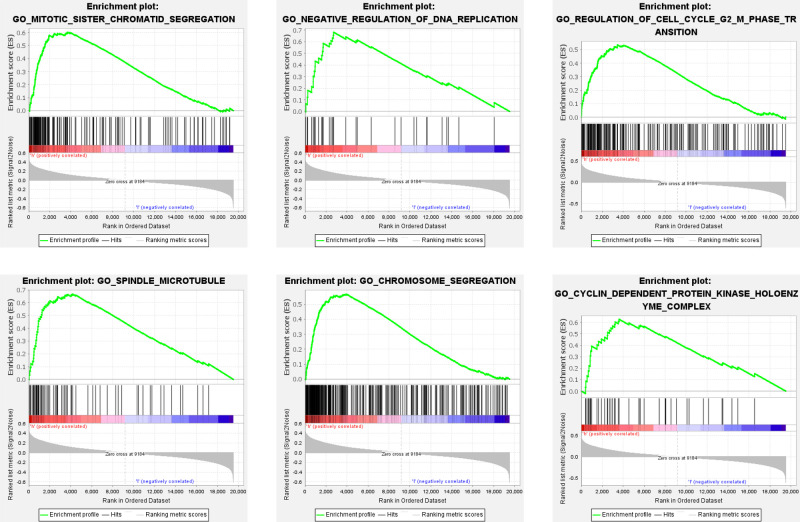
GSEA analysis between high- and low-risk groups. Some cell cycle-related pathways were gathered in the high-risk group: “MITOTIC_SISTER_CHROMATID_SEGREGATION,” “NEGATIVE_REGULATION_OF_DNA_REPLICATION,” “REGULATION_OF_CELL_CYCLE_G2_M_PHASE_TRANSITION,” “SPINDLE_MICROTUBULE,” “CHROMOSOME_SEGREGATION,” “CYCLIN_DEPENDENT_PROTEIN_KINASE_HOLOENZYME_COMPLEX.” GSEA, Gene set enrichment analysis; NES, normalized enrichment score.

To investigate the underlying molecular mechanisms of the apoptosis-related gene signature, we performed a GSEA enrichment analysis of all genes between high- and low-risk patients in the training set. As shown in Figure 5 and Supplementary Table S4, multiple cell cycle associated biological processes such as “CYCLIN DEPENDENT PROTEIN KINASE HOLOENZYME COMPLEX,” “NEGATIVE REGULATION OF DNA REPLICATION,” “CHROMOSOME SEGREGATION,” “REGULATION OF CELL CYCLE G2 M PHASE TRANSITION,” and so on, were substantially enrolled in the high-risk patients. The aforesaid results indicated that BC patients with high-risk scores could be closely concerned with the cell cycle phase.

## Discussion

BC is a very heterogeneous cancer. The latest statistics indicate that it has become the most prevalent cancer in women, which is posing a serious threat to the health of women worldwide. In recent years, significant progress has been made in the treatment of BC, but it is still very important to consider the diagnostic and prognostic value of genomics in personal diagnosis (12), especially in the detection of early diagnosis markers for BC.

The process of apoptosis can be roughly divided into the following stages: receiving apoptosis signal → interaction between apoptosis regulating molecules → activation of proteolytic enzyme (Caspase) → entering a continuous reaction process (13). Due to the different initiation stages of apoptosis, the signaling pathway of apoptosis can be divided into three main pathways, namely mitochondrial pathway, endoplasmic reticulum pathway, and death receptor pathway. The mitochondrial pathway consists of BH3 domain-containing Bcl-2 family members (Bid, Bad, Bim, Harikari, Noxa, etc.) and additional Bcl-2 family members (Bax subfamily members, Bax, Bak, etc.). The Bax subfamily members bound to the outer mitochondrial membrane or present in the cytoplasm interact, release cytochrome C, and combine with caspase-9 to form apoptotic bodies (14). The activated caspase-9 can activate other caspases such as caspase-3 and caspase-7, thereby inducing apoptosis. The endoplasmic reticulum pathway, which is caused by endoplasmic reticulum dysfunction, rather than triggered by apoptotic signals targeting cell membranes or mitochondria. The endoplasmic reticulum is the main site of intracellular protein synthesis and the main reservoir of Ca^2+^ (15). Disruption of endoplasmic reticulum Ca^2+^ balance or excessive accumulation of endoplasmic reticulum proteins are key steps that induce the expression of Caspase-12 located at the endoplasmic reticulum membrane and the translocation of cytoplasmic Caspase-7 to the endoplasmic reticulum surface. Caspase-7 can activate Caspase-12, and the activated Caspase-12 can further cut Caspase-3 to induce apoptosis (16). The death receptor pathway is initiated by various external factors and then transmits apoptosis signals through different signaling systems, causing apoptosis. There are five known death receptors, TFR-1 (also known as CD120a or p55), Fas (CD95 or Apo1), DR3 (death receptor 3, also known as Apo3, WSL-1, TRAMP, LARD), DR4, and DR5 (Apo2, TRAIL-R2, TRICK2, KILLER) (17). The corresponding ligands of the first three receptors are TNF, FasL (CD95L), Apo-3L (DR3L), and the latter two are Apo-2L (TRAIL). For example, the ligand FasL can first induce Fas trimerization. After the trimeric Fas and FasL are combined, the death domains of the three Fas molecules are clustered together, attracting another molecule with the same death domain in the cytoplasm (18). The protein FADD forms an apoptosis-inducing complex on the cell membrane, thereby activating caspase8, which in turn causes the subsequent cascade reaction, and the cell undergoes apoptosis (19, 20).

Determining the best surface markers for diagnostic and therapeutic purposes is a well-known challenge in the development of clinically relevant targeted BC therapies. Although most of the quantitative proteomics techniques used to identify protein markers are far from being used in daily clinical practice, more and more patient cohorts and clinical information are providing expression data. The results and findings of this study can provide the basis and important reference for the clinical diagnosis and treatment of BC. At the same time, the relevant findings and conclusions need further mechanism exploration and molecular verification in the future; Three prognostic genes, *IL3RA*, *AIFM1,* and *PIK3CA*. *IL3RA* and *PIK3CA* have been reported to be closely related to the incidence and development of BC, which is consistent with our results (21, 22). However, there are few studies on *AIFM1* in BC, only found to be related to TNBC cell apoptosis (23). Among them, *IL3RA*, also known as cell surface antigen CD123, is the interleukin (IL)—3 receptor on the surface of dendritic cells α Chain. The Tesla team studied 79 patients with acute myeloid leukemia (AML), 25 patients with acute B lymphocytic leukemia (ALL-B), and 7 patients with acute T lymphocytic leukemia (ALL-T). There is a significant correlation between the level of *IL3RA* expression and the number of blasts at the time of leukemia diagnosis. The level of expression of *IL3RA* correlates significantly with the number of leukemia blasts at the time of diagnosis. The complete remission rate and survival time of patients with elevated *IL3RA* levels are lower than those of patients with normal *IL3RA* levels (24). *IL3RA* is commonly expressed in acute myeloid leukemia (AML) and classic Hodgkin lymphoma (HL) (25), In order to provide an opportunity for the use of *IL3RA* directed antibody drug conjugates (ADC) in the treatment of AML and HL, on December 21, 2018, The US Food and Drug Administration (FDA) has approved Tagraxofusp Erzs for the treatment of plasmacytoid dendritic cell tumors in adults and children of 2 years and older (26). Tatiana lopatina found *IL3RA* Inhibition of metastatic spread of triple-negative breast cancer by extracellular vesicle reprogramming (21). Although there is not much research on solid tumors, the future prospects are worth looking forward to. The research of *AIFM1* mainly focuses on deafness (27). It was later discovered that it plays a role in the coding of apoptosis triggers. As a conservative redox switch, it measures the metabolic state of mitochondria and turns it into a binary life/death solution (28). The product of this gene induces mitochondria to release cytochrome c and caspase-9, which are pro-apoptotic proteins (29, 30). In the study of liver cancer, it was found that the full-length apoptosis-inducing factor mitochondrial-associated 1 (*AIFM1*) (∼67 kDa) was cleaved at its N-terminus to produce truncated *AIFM1* (∼57 kDa), which was not dependent on caspase Enzymes induce apoptosis, overexpression of full-length *AIFM1* can induce caspase-dependent apoptosis and inhibit cell growth of liver cancer cells, our data reveal the potential role of rAd-*AIFM1* in HCC gene therapy (31). Studies have shown that *PIK3CA* is a prognostic factor for oral cancer, and studies have also shown that *PIK3CA* is involved in the metabolic mechanism of breast cancer (32, 33). Inhibition of *PIK3CA* can cause tumor regression in nude mice (34). In primary and metastatic breast cancer, up to 40% of *PIK3CA* mutations are positive for estrogen receptor (ER) and negative for human epidermal growth factor (HER2). HER2 is overexpressed in 20%–30% of breast cancers. HER1, HER2, HER3, and HER4 are membrane receptor tyrosine kinases involved in HER signal transduction, which can be linked via various ligands and lead to the activation of phosphatidylinositol-3 kinase / protein kinase B(PI3K/PKB) (35). The current study found that *IL3RA* has considerable significance in the prognostic risk model of leukemia, while *PIK3CA* in breast cancer. After targeted treatment, it was found that patients with *PIK3CA* mutation HR+/Her2-mBC showed a poor prognosis and showed a poor prognosis for chemotherapy (22, 36, 37). Unfortunately, the survival analysis of *AIFM1* in tumor has not been found yet.

Our research has certain limits. Although our study has the advantage of using a large number of cohorts from the TCGA database to construct and verify apoptosis prediction models, this study still uses a retrospective innovation. Therefore, we need a prospective cohort to validate our model. In addition, further functional studies are needed to explore the molecular functions of the two established apoptotic genes in the course of BC. According to our understanding, an apoptotic prognostic model of BC patients was identified and verified for the first time in our study, which contains two apoptotic genes (*IL3RA* and *AIFM1*) and can be used as an independent prognostic marker for BC patients. In addition, this model can provide new clinical applications for BC, taking into account apoptosis goals and treatments related to apoptosis.

## Conclusion

In this study, the significance of apoptosis-related genes in prognosis was analyzed based on the data of breast cancer samples in TCGA. Firstly, 21 differential apoptosis-related genes were screened. Three prognostic-related biomarkers (*IL3RA*, *AIFM1*, *PIK3CA*) were obtained by Cox analysis, which were used to construct the risk model. And the validity of the risk model was verified by the TCGA validation set and external validation set GSE1456. Independent prognostic analysis showed that age, M, N, and riskScore were independent prognostic factors for breast cancer patients (*p* < 0.05). GSEA enrichment analysis was performed on all genes in the samples of high and low expression groups in the training set. The high-risk group was significantly enriched to 462 gene sets (*p* < 0.01), and the low-risk group was significantly enriched to 181 gene sets (*p* < 0.01). The results and findings of this study can provide a theoretical basis for the clinical diagnosis and treatment of breast cancer, and the influence mechanism of these three genes on diseases could be further explored in the future.

## Material and Methods

### Data Processing

We collected transcriptome data and related clinical information from 113 normal humans and 1,091 BC specimens from the Cancer Genome Atlas Database (TCGA) database portal for 26 years from 1988 to 2013 year (https://portal.gdc.cancer.gov/). And the clinical information of these 1091 BRCA patients in the TCGA database can be seen in Table 3. In addition, GSE1456, including 159 tumor samples, was used as an external validation set, which was downloaded from Gene Expression Omnibus (GEO) database. Furthermore, 87 apoptosis-related genes were recognized within the KEGG_APOPTOSIS gene set from the Molecular Signatures Database (MSigDB, https://www.gsea-msigdb.org/gsea/msigdb). See Supplementary Table S1 for a list of apoptosis-related genes.

**Table 3:** The clinical information of these 1091 BRCA patients in the TCGA database.

Characteristics	No.
Total cases	1091
Age (<50)	296
Age (≥50)	795
LIVING	943
DECEASED	151
clinical_stage (Stage I)	182
clinical_stage (Stage II)	618
clinical_stage (Stage III)	247
clinical_stage (Stage IV)	20
clinical_stage (Stage X)	24
Subtype (HER2+)	37
Subtype (Luminal A)	369
Subtype (Luminal B)	100
Subtype (TNBC)	116
Subtype (NA)	473
new_tumor_event_after_initial_treatment (NO)	296
new_tumor_event_after_initial_treatment (YES)	795
pathologic_M(M0)	907
pathologic_M(M1)	22
pathologic_M(MX)	162
pathologic_N(N0)	513
pathologic_N(N1)	361
pathologic_N(N2)	120
pathologic_N(N3)	77
pathologic_N(NX)	20
pathologic_T(T1)	280
pathologic_T(T2)	632
pathologic_T(T3)	138
pathologic_T(T4)	38
pathologic_T(TX)	3

### Identification of Apoptosis-Related DEGs in BC

To obtain apoptosis genes associated with breast cancer, the R package Linear Models for Microarray Data (Limma) was executed for differential expression analysis to screened differentially expressed genes (DEGs) between normal and BC samples at first. With adj. *p*-value < 0.05 and |log2 fold change (FC)| ≥ 0.585. Then the expression of DEGs was visualized by a volcano plot. Apoptosis-related DEGs were then identified by assessing the overlap between DEGs and the abovementioned list of 87 apoptosis-related genes.

### Construction and Evaluation of Apoptosis-Related Gene Signature for BC

A total of 1,089 BC patients from TCGA were randomly assigned and equally divided into training set (*n* = 544) and verification set (*n* = 545). By R package survival, univariate Cox analysis was implemented in the training set based on the identified differentially expressed apoptosis-related genes to recognize the variables linked to OS in BC patients. Overall survival refers to the total period from the start of the sample being confirmed as a BC patient until the death/missing visit/end of the study for that sample (this study was measured in years). All overall survival information involved in this study was obtained from the TCGA-BC database. Further, multivariate Cox analysis was utilized to determine the best variables for constructing prognostic signature. The present study proposed to assess the predictive validity of genetic prognostic signature using a risk score system. The risk score of each patient was calculated according to the gene expression and the prognostic coefficient obtained by multivariate COX regression. The formula for calculating the risk score is shown below:

$$\begin{array}{rl} & \mathrm{risk}\;\mathrm{score}=\mathrm{expression}\;\mathrm{level}\;\mathrm{of}\;\mathrm{Gen}e_{1}\times \beta_{1}\\ & +\mathrm{expression}\;\mathrm{level}\;\mathrm{of}\;\mathrm{Gen}e_{2}\times \beta_{2\;+\;}\ldots \\ & +\mathrm{expression}\;\mathrm{level}\;\mathrm{of}\;\mathrm{Gen}e_{n}\times \beta_{n} \end{array}$$

Then the BC samples were divided into high-risk groups and low-risk groups according to the average risk scores of their respective cohorts. Kaplan–Meier (KM) analysis and logarithmic test were used to analyze the survival of the two groups of patients. Then we draw the receptor performance curve (ROC) and calculate the AUC to determine the specificity and sensitivity of the genetic markers used to predict the prognosis of BC patients. Using R software to view the risk distribution, survival status, and expression of the three prognostic genes of BC patients in the training and validation set. GSE1456 verified the reliability of the model as an external validation set.

### Independent Prognostic Analysis and Construction of a Nomogram

We included risk scores and multiple clinical characteristics (age, race, M, T, N, stage) in univariate and multivariate Cox regression analyses to identify factors that independently predicted overall survival in BC patients. Only variables with *p* < 0.05 in both univariate and multivariate Cox analyses were deemed to be independent prognostic factors for BC. A nomogram with identified independent prognostic parameters was then generated using the “rms” and “nomogramEx” R package. We drew corresponding calibration curves of 1, 3, and 5 years with the “regplot” R package to further verify the accuracy of the nomogram.

### Gene Set Enrichment Analysis (GSEA)

With GSEA (version 4.1.0), we analyzed the differential pathways between high- and low-risk groups to provide a theoretical basis for revealing the underlying molecular mechanisms of the risk scoring system. *p* < 0.01 was considered to be extremely enriched. We selected “c2.cp.kegg.v7.2.symbols.gmt” and “c5.all.v7.2.symbols.gmt Gene ontology” as the reference gene set.

### Statistical Analysis

All statistical analyzes were performed using the R software. The statistical significance of the variables between the two groups was determined by the chi-squared test. Test. Unless otherwise stated, *p* < 0.05 was considered to be statistically significant.

## Data Availability

The datasets presented in this study can be found in online repositories. The names of the repository/repositories and accession number(s) can be found in the article/Supplementary Material.
