# Supplementary material for: A Signature of Three Apoptosis-Related Genes Predicts Overall Survival in Breast Cancer
Source: Front Surg. 2022 May 17;9:863035. doi: 10.3389/fsurg.2022.863035 (PMC9235836; doi:10.3389/fsurg.2022.863035)
Supplement: Supplementary file 1 [file Data_Sheet1_v1.docx]

**Supplementary Material：**


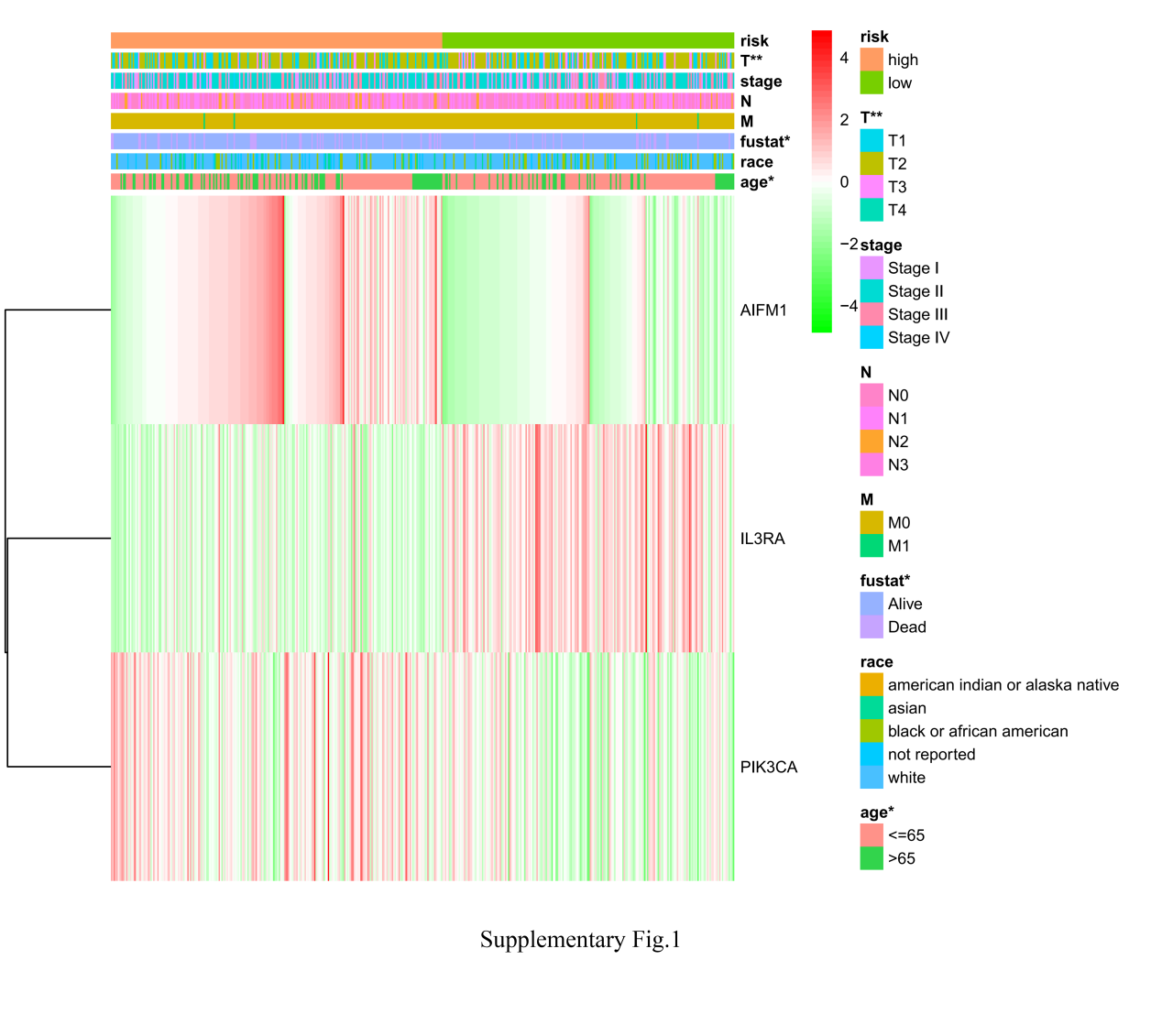


Supplementary Fig.1

Supplementary Table 1

| KEGG_APOPTOSIS |
| --- |
| > Apoptosis |
| AIFM1 |
| AKT1 |
| AKT2 |
| AKT3 |
| APAF1 |
| ATM |
| BAD |
| BAX |
| BCL2 |
| BCL2L1 |
| BID |
| BIRC2 |
| BIRC3 |
| CAPN1 |
| CAPN2 |
| CASP10 |
| CASP3 |
| CASP6 |
| CASP7 |
| CASP8 |
| CASP9 |
| CFLAR |
| CHP1 |
| CHP2 |
| CHUK |
| CSF2RB |
| CYCS |
| DFFA |
| DFFB |
| ENDOD1 |
| ENDOG |
| EXOG |
| FADD |
| FAS |
| FASLG |
| IKBKB |
| IKBKG |
| IL1A |
| IL1B |
| IL1R1 |
| IL1RAP |
| IL3 |
| IL3RA |
| IRAK1 |
| IRAK2 |
| IRAK3 |
| IRAK4 |
| MAP3K14 |
| MYD88 |
| NFKB1 |
| NFKBIA |
| NGF |
| NTRK1 |
| PIK3CA |
| PIK3CB |
| PIK3CD |
| PIK3CG |
| PIK3R1 |
| PIK3R2 |
| PIK3R3 |
| PIK3R5 |
| PPP3CA |
| PPP3CB |
| PPP3CC |
| PPP3R1 |
| PPP3R2 |
| PRKACA |
| PRKACB |
| PRKACG |
| PRKAR1A |
| PRKAR1B |
| PRKAR2A |
| PRKAR2B |
| PRKX |
| RELA |
| RIPK1 |
| TNF |
| TNFRSF10A |
| TNFRSF10B |
| TNFRSF10C |
| TNFRSF10D |
| TNFRSF1A |
| TNFSF10 |
| TP53 |
| TRADD |
| TRAF2 |
| XIAP |

Supplementary Table 2

| id | logFC | AveExpr | t | P.Value | adj.P.Val | B |
| --- | --- | --- | --- | --- | --- | --- |
| CA4 | -2.696112954 | 0.476505899 | -50.16476815 | 3.83E-297 | 7.49E-293 | 670.229556 |
| CD300LG | -3.370299729 | 0.792438937 | -49.00869313 | 5.83E-289 | 5.70E-285 | 651.4369398 |
| SCARA5 | -3.602012566 | 0.934034533 | -45.41812502 | 3.79E-263 | 2.47E-259 | 592.139903 |
| ARHGAP20 | -1.486787229 | 0.566314017 | -45.35502796 | 1.09E-262 | 5.33E-259 | 591.0859858 |
| C1QTNF9 | -0.857909131 | 0.197172304 | -45.06239774 | 1.47E-260 | 5.75E-257 | 586.1930842 |
| ABCA10 | -1.479368492 | 0.426987451 | -44.28216349 | 7.30E-255 | 2.38E-251 | 573.1073002 |
| TSLP | -0.876347819 | 0.225437077 | -43.6117487 | 5.98E-250 | 1.67E-246 | 561.8184828 |
| LYVE1 | -3.410759842 | 1.102662387 | -43.52391607 | 2.64E-249 | 6.45E-246 | 560.3365311 |
| GLYAT | -2.261236741 | 0.370282498 | -42.96578184 | 3.37E-245 | 7.32E-242 | 550.9037978 |
| HSD17B13 | -1.643653276 | 0.295043688 | -40.92845684 | 4.03E-230 | 7.88E-227 | 516.2582165 |
| ASPA | -1.311504935 | 0.393634747 | -40.17438751 | 1.66E-224 | 2.95E-221 | 503.3583287 |
| FXYD1 | -1.552665358 | 0.485463436 | -39.87853655 | 2.67E-222 | 4.35E-219 | 498.2871617 |
| ABCA9 | -2.113306596 | 0.75107338 | -39.67729898 | 8.49E-221 | 1.28E-217 | 494.8347071 |
| ACVR1C | -2.150540259 | 0.476595237 | -39.11460262 | 1.37E-216 | 1.91E-213 | 485.168598 |
| TMEM132C | -2.54262371 | 0.659436999 | -39.0796402 | 2.49E-216 | 3.25E-213 | 484.5674286 |
| MYOM1 | -1.782113434 | 0.649709372 | -38.86100367 | 1.08E-214 | 1.32E-211 | 480.8065693 |
| NKAPL | -0.896243908 | 0.346500972 | -38.83275049 | 1.76E-214 | 2.02E-211 | 480.3203938 |
| ADAMTS5 | -2.530144576 | 1.437772633 | -38.68303539 | 2.33E-213 | 2.53E-210 | 477.7434499 |
| PAMR1 | -2.957644217 | 1.46287364 | -38.65045483 | 4.08E-213 | 4.20E-210 | 477.182515 |
| SLC2A4 | -2.107352335 | 0.622466056 | -38.56100701 | 1.91E-212 | 1.87E-209 | 475.6422375 |
| BTNL9 | -2.991433795 | 1.27306443 | -38.49283738 | 6.19E-212 | 5.76E-209 | 474.4681076 |
| HEPACAM | -1.256717541 | 0.204950886 | -38.38965464 | 3.68E-211 | 3.27E-208 | 472.6905051 |
| ITIH5 | -3.007405294 | 1.36911171 | -38.02784599 | 1.90E-208 | 1.62E-205 | 466.4535364 |
| TNXB | -2.90607385 | 1.130363499 | -37.92701676 | 1.09E-207 | 8.85E-205 | 464.7143942 |
| HIF3A | -1.208678363 | 0.270319324 | -37.56231187 | 5.96E-205 | 4.66E-202 | 458.4203706 |
| SLC17A7 | -0.771731133 | 0.248780393 | -37.52858544 | 1.07E-204 | 8.03E-202 | 457.8380639 |
| PDE2A | -2.241642317 | 1.22976291 | -37.4368609 | 5.22E-204 | 3.78E-201 | 456.2541728 |
| GPAM | -3.110164023 | 2.07144176 | -37.25675761 | 1.18E-202 | 8.23E-200 | 453.1432871 |
| ABCA8 | -2.457992732 | 0.856084867 | -36.99755354 | 1.05E-200 | 7.07E-198 | 448.664183 |
| AQP7 | -3.171358706 | 0.87266496 | -36.91629229 | 4.28E-200 | 2.79E-197 | 447.2595301 |
| RDH5 | -1.834218899 | 0.534103827 | -36.89518496 | 6.17E-200 | 3.89E-197 | 446.8946434 |
| KLB | -1.997430025 | 0.534022292 | -36.50982291 | 4.90E-197 | 2.99E-194 | 440.23058 |
| LVRN | -1.363373195 | 0.252986938 | -36.5010923 | 5.70E-197 | 3.38E-194 | 440.0795554 |
| LEP | -4.517699838 | 1.130375866 | -36.26977387 | 3.15E-195 | 1.81E-192 | 436.0774703 |
| CAVIN2 | -3.754184662 | 1.902806333 | -36.20858395 | 9.09E-195 | 5.07E-192 | 435.0186055 |
| ANGPT4 | -0.717004934 | 0.181974313 | -36.01903266 | 2.43E-193 | 1.32E-190 | 431.7380039 |
| KCNIP2 | -3.388767706 | 1.210468956 | -35.92195185 | 1.31E-192 | 6.92E-190 | 430.0575352 |
| CPA1 | -0.666666681 | 0.117126045 | -35.86001456 | 3.83E-192 | 1.97E-189 | 428.985312 |
| DMD | -2.170601736 | 0.978990028 | -35.68299055 | 8.27E-191 | 4.14E-188 | 425.9204218 |
| ATOH8 | -1.692981959 | 0.593667447 | -35.54082001 | 9.74E-190 | 4.76E-187 | 423.4586257 |
| MAP1LC3C | -1.855559391 | 0.611252792 | -35.4971769 | 2.08E-189 | 9.90E-187 | 422.7028565 |
| NPR1 | -2.662647951 | 1.661553496 | -35.47126728 | 3.26E-189 | 1.50E-186 | 422.2541676 |
| TMEM252 | -0.58737864 | 0.151582931 | -35.47063368 | 3.29E-189 | 1.50E-186 | 422.243195 |
| CCDC178 | -0.770317805 | 0.230180351 | -35.34414588 | 2.96E-188 | 1.31E-185 | 420.0526355 |
| ALDH1L1 | -1.655169063 | 0.366633529 | -34.96018865 | 2.31E-185 | 1.01E-182 | 413.4022447 |
| GIPC2 | -1.399745139 | 0.617394976 | -34.78679324 | 4.69E-184 | 1.99E-181 | 410.3986382 |
| LRRN4CL | -2.196293429 | 0.987408266 | -34.75858392 | 7.66E-184 | 3.18E-181 | 409.9099792 |
| MME | -3.272435522 | 1.909471391 | -34.71271662 | 1.70E-183 | 6.91E-181 | 409.1154345 |
| FAM13A | -1.753623336 | 1.386842095 | -34.67485052 | 3.28E-183 | 1.31E-180 | 408.4594898 |
| CCL14 | -1.967034626 | 0.710869507 | -34.60131046 | 1.17E-182 | 4.59E-180 | 407.1855715 |
| ALDH1A2 | -1.285039016 | 0.467472362 | -34.58962923 | 1.44E-182 | 5.51E-180 | 406.9832201 |
| SMYD1 | -1.944527428 | 0.390375976 | -34.42978516 | 2.30E-181 | 8.66E-179 | 404.2142914 |
| GPR146 | -1.16815916 | 0.54958478 | -34.42364709 | 2.56E-181 | 9.45E-179 | 404.1079645 |
| TMEM220 | -1.529066912 | 1.032442185 | -34.4074003 | 3.40E-181 | 1.23E-178 | 403.8265297 |
| ACSM5 | -1.661345262 | 0.637326416 | -34.32866368 | 1.33E-180 | 4.73E-178 | 402.462628 |
| MAMDC2 | -2.548901316 | 1.33548649 | -34.30578639 | 1.98E-180 | 6.92E-178 | 402.0663447 |
| ATP1A2 | -2.627282728 | 0.765967757 | -34.30238839 | 2.10E-180 | 7.21E-178 | 402.0074843 |
| NIPSNAP3B | -1.60973506 | 0.988465274 | -34.19966071 | 1.25E-179 | 4.21E-177 | 400.2280562 |
| TNMD | -2.312104499 | 0.594963593 | -34.03511014 | 2.17E-178 | 7.20E-176 | 397.3778871 |
| CHL1 | -2.026245878 | 0.808435363 | -33.88221673 | 3.09E-177 | 1.01E-174 | 394.7298396 |
| IGSF10 | -1.687416706 | 0.593799455 | -33.84869709 | 5.52E-177 | 1.77E-174 | 394.1493264 |
| MRAP | -1.967565448 | 0.464074454 | -33.77858349 | 1.86E-176 | 5.87E-174 | 392.9350998 |
| LEPR | -2.028434548 | 1.099924755 | -33.74679401 | 3.24E-176 | 1.00E-173 | 392.3845901 |
| SLC19A3 | -3.101538516 | 0.947924766 | -33.71111314 | 6.01E-176 | 1.83E-173 | 391.7667075 |
| ADRB2 | -1.777719123 | 0.915845411 | -33.65170347 | 1.68E-175 | 5.06E-173 | 390.7379543 |
| HSPB7 | -2.950901831 | 1.088949701 | -33.54529154 | 1.07E-174 | 3.16E-172 | 388.8954288 |
| NAALAD2 | -0.745088293 | 0.279467311 | -33.43663222 | 7.03E-174 | 2.05E-171 | 387.0141802 |
| KLHL29 | -1.301853725 | 0.589437393 | -33.35158371 | 3.07E-173 | 8.83E-171 | 385.5418593 |
| ADH1C | -2.500410496 | 0.858014231 | -33.33947999 | 3.79E-173 | 1.07E-170 | 385.3323364 |
| FHL1 | -3.917729811 | 2.820847296 | -33.30543018 | 6.84E-173 | 1.91E-170 | 384.7429287 |
| LRRC2 | -0.758659385 | 0.233354518 | -33.20988586 | 3.59E-172 | 9.75E-170 | 383.0891675 |
| MAB21L1 | -1.384973049 | 0.589420949 | -33.20982545 | 3.59E-172 | 9.75E-170 | 383.088122 |
| EBF1 | -2.257450086 | 1.599767559 | -33.14208579 | 1.16E-171 | 3.11E-169 | 381.9157465 |
| ABCA6 | -1.319613812 | 0.608899639 | -33.13541132 | 1.30E-171 | 3.45E-169 | 381.8002365 |
| C14orf180 | -2.067544907 | 0.441712218 | -33.11554747 | 1.84E-171 | 4.80E-169 | 381.456474 |
| CRHBP | -1.102276823 | 0.320473562 | -33.10760687 | 2.11E-171 | 5.43E-169 | 381.319057 |
| COL6A6 | -1.396125058 | 0.450665347 | -33.10196353 | 2.33E-171 | 5.91E-169 | 381.2213964 |
| MYOC | -2.301425714 | 0.337946336 | -33.00448127 | 1.26E-170 | 3.12E-168 | 379.534542 |
| ANKRD53 | -0.981897097 | 0.453613887 | -32.87435918 | 1.21E-169 | 2.91E-167 | 377.283255 |
| CAV1 | -3.218812662 | 4.316138765 | -32.86873961 | 1.33E-169 | 3.17E-167 | 377.1860389 |
| GPIHBP1 | -2.7446934 | 1.334834165 | -32.76531501 | 7.98E-169 | 1.88E-166 | 375.3969981 |
| ADRA1A | -0.741032373 | 0.128107565 | -32.67037413 | 4.13E-168 | 9.62E-166 | 373.7549822 |
| CAV2 | -2.549230711 | 2.408614012 | -32.65479477 | 5.41E-168 | 1.24E-165 | 373.4855611 |
| ABCB5 | -0.587021672 | 0.124812843 | -32.62683796 | 8.79E-168 | 2.00E-165 | 373.0021098 |
| PRRG3 | -0.970271637 | 0.288666024 | -32.59389229 | 1.56E-167 | 3.49E-165 | 372.4324188 |
| KLF15 | -1.986779278 | 0.869377343 | -32.54620419 | 3.55E-167 | 7.89E-165 | 371.6078659 |
| FREM1 | -1.338709052 | 0.514600645 | -32.44656042 | 2.00E-166 | 4.38E-164 | 369.8852147 |
| ANGPTL7 | -1.792355592 | 0.372733339 | -32.43398049 | 2.48E-166 | 5.39E-164 | 369.6677556 |
| HPSE2 | -1.367636868 | 0.356883506 | -32.40047367 | 4.43E-166 | 9.52E-164 | 369.0885775 |
| GDF10 | -2.020157756 | 0.649232002 | -32.28806066 | 3.11E-165 | 6.60E-163 | 367.1457697 |
| SLC16A7 | -1.56876871 | 0.685654983 | -32.28377775 | 3.34E-165 | 7.03E-163 | 367.0717584 |
| KLHL31 | -1.491518771 | 0.551419641 | -32.23749684 | 7.45E-165 | 1.55E-162 | 366.2720394 |
| PCK1 | -2.312277887 | 0.529836175 | -32.21852916 | 1.04E-164 | 2.13E-162 | 365.9443077 |
| STARD9 | -0.99119055 | 0.576066503 | -32.18399743 | 1.88E-164 | 3.83E-162 | 365.3476893 |
| SLC7A10 | -1.952519209 | 0.47292302 | -32.15317749 | 3.21E-164 | 6.46E-162 | 364.8152405 |
| SCN4A | -1.225378533 | 0.399698118 | -32.0967429 | 8.52E-164 | 1.70E-161 | 363.840368 |
| ABCD2 | -1.429379584 | 0.415834518 | -32.00205878 | 4.39E-163 | 8.66E-161 | 362.2050521 |
| TNS1 | -2.63834156 | 3.498093552 | -31.97680177 | 6.79E-163 | 1.33E-160 | 361.7688948 |
| BMX | -1.359833232 | 0.613912074 | -31.87566903 | 3.90E-162 | 7.56E-160 | 360.0227349 |
| ADH1A | -0.733515573 | 0.121694716 | -31.84903527 | 6.19E-162 | 1.19E-159 | 359.5629512 |
| SYNE3 | -1.647362887 | 0.923346298 | -31.83647858 | 7.69E-162 | 1.46E-159 | 359.3461939 |
| CLEC3B | -3.346754543 | 2.744712609 | -31.80808423 | 1.26E-161 | 2.36E-159 | 358.8560684 |
| SCN2B | -0.931442047 | 0.299650928 | -31.7943855 | 1.59E-161 | 2.96E-159 | 358.6196226 |
| MICU3 | -1.019917592 | 0.550542138 | -31.76328551 | 2.73E-161 | 5.03E-159 | 358.0828563 |
| ANGPTL1 | -2.11703087 | 0.99176159 | -31.6843236 | 1.07E-160 | 1.95E-158 | 356.7202281 |
| ADCYAP1R1 | -1.195182932 | 0.474809924 | -31.55634845 | 9.76E-160 | 1.77E-157 | 354.512432 |
| FAM13C | -0.955140716 | 0.494131242 | -31.49427194 | 2.85E-159 | 5.11E-157 | 353.4417993 |
| PPP1R12B | -1.704752616 | 1.824632519 | -31.43943139 | 7.36E-159 | 1.31E-156 | 352.4961311 |
| TMEM246 | -1.04667137 | 0.597532071 | -31.43743955 | 7.61E-159 | 1.34E-156 | 352.4617868 |
| CORO2B | -1.489158377 | 0.995339588 | -31.4095804 | 1.23E-158 | 2.15E-156 | 351.9814483 |
| PALMD | -2.410195316 | 2.08710589 | -31.24233003 | 2.21E-157 | 3.83E-155 | 349.0986568 |
| SIK2 | -1.929368719 | 2.587988073 | -31.03405959 | 8.06E-156 | 1.35E-153 | 345.5110497 |
| CLDN19 | -1.371432206 | 0.353973483 | -30.99266605 | 1.65E-155 | 2.73E-153 | 344.7983238 |
| CHRDL1 | -4.130807053 | 2.11289529 | -30.94174832 | 3.96E-155 | 6.50E-153 | 343.9217522 |
| ACACB | -2.583791628 | 1.685933266 | -30.93746455 | 4.26E-155 | 6.94E-153 | 343.8480126 |
| SCN4B | -2.108739952 | 1.373219518 | -30.89055796 | 9.58E-155 | 1.55E-152 | 343.0406503 |
| CXCL2 | -2.966472227 | 1.284375438 | -30.88365034 | 1.08E-154 | 1.73E-152 | 342.9217672 |
| SVEP1 | -2.465759853 | 1.519090054 | -30.87665402 | 1.22E-154 | 1.93E-152 | 342.8013606 |
| KL | -1.173717711 | 0.541702739 | -30.82178263 | 3.13E-154 | 4.94E-152 | 341.857133 |
| APOB | -0.744954615 | 0.103758977 | -30.80713434 | 4.03E-154 | 6.31E-152 | 341.605098 |
| EBF3 | -1.697207184 | 1.007267435 | -30.6219058 | 9.82E-153 | 1.51E-150 | 338.4193231 |
| HSPB6 | -4.035442142 | 2.508215477 | -30.59512101 | 1.56E-152 | 2.38E-150 | 337.9588395 |
| HCAR2 | -1.973493824 | 0.936033638 | -30.49961506 | 8.07E-152 | 1.22E-149 | 336.3173099 |
| BLOC1S1-RDH5 | -0.708613534 | 0.406577744 | -30.4975165 | 8.36E-152 | 1.26E-149 | 336.2812476 |
| SORBS1 | -2.939058919 | 2.60045464 | -30.47778822 | 1.17E-151 | 1.75E-149 | 335.9422468 |
| DMGDH | -0.902182135 | 0.409996044 | -30.40138776 | 4.38E-151 | 6.48E-149 | 334.6296841 |
| MYZAP | -2.01553958 | 1.126999904 | -30.38879882 | 5.44E-151 | 7.99E-149 | 334.4134464 |
| TRARG1 | -4.116894892 | 1.412085229 | -30.34986668 | 1.06E-150 | 1.55E-148 | 333.7447907 |
| PAK5 | -0.767052262 | 0.197786051 | -30.31285861 | 2.01E-150 | 2.91E-148 | 333.109285 |
| HLF | -2.302866675 | 1.162951925 | -30.16936172 | 2.37E-149 | 3.40E-147 | 330.6461253 |
| ITPRIPL1 | -1.276604667 | 0.643513109 | -30.16021811 | 2.77E-149 | 3.95E-147 | 330.4892268 |
| HOXA4 | -1.231589251 | 0.630796529 | -30.15246498 | 3.17E-149 | 4.49E-147 | 330.356193 |
| SPRY2 | -2.405273726 | 2.315777577 | -30.12944693 | 4.71E-149 | 6.62E-147 | 329.9612608 |
| ADAM33 | -1.863638015 | 0.913267558 | -30.12137004 | 5.41E-149 | 7.55E-147 | 329.8226914 |
| AL845331.2 | -1.185534109 | 0.206576354 | -30.09714437 | 8.20E-149 | 1.14E-146 | 329.4071001 |
| CDO1 | -2.812153022 | 1.508065646 | -30.04007899 | 2.19E-148 | 3.01E-146 | 328.4283282 |
| NMUR1 | -1.05148812 | 0.330330279 | -30.03180588 | 2.52E-148 | 3.44E-146 | 328.2864516 |
| DEFB132 | -1.787851367 | 0.446291175 | -29.94702879 | 1.08E-147 | 1.47E-145 | 326.8329187 |
| OXTR | -3.61108205 | 1.755968749 | -29.94076874 | 1.20E-147 | 1.62E-145 | 326.7256114 |
| CFL2 | -1.441526409 | 1.812899081 | -29.93846918 | 1.25E-147 | 1.68E-145 | 326.686194 |
| INSYN2B | -1.509893133 | 0.379434274 | -29.93338106 | 1.37E-147 | 1.82E-145 | 326.5989788 |
| PPARG | -2.486941172 | 1.737139796 | -29.91974982 | 1.73E-147 | 2.28E-145 | 326.3653369 |
| FRMD1 | -0.681694542 | 0.13263778 | -29.90985688 | 2.05E-147 | 2.68E-145 | 326.1957798 |
| ITGA7 | -2.673146944 | 1.721133737 | -29.84538233 | 6.19E-147 | 8.07E-145 | 325.0909386 |
| LPL | -4.249371551 | 2.994975797 | -29.81769529 | 9.96E-147 | 1.29E-144 | 324.6165991 |
| GPD1 | -4.662083884 | 1.988405006 | -29.81479739 | 1.05E-146 | 1.35E-144 | 324.5669553 |
| CAPN11 | -1.187076612 | 0.521654166 | -29.8093776 | 1.15E-146 | 1.47E-144 | 324.4741115 |
| FMO2 | -3.258732998 | 1.851785384 | -29.72832176 | 4.62E-146 | 5.86E-144 | 323.0858837 |
| SGK2 | -1.255033664 | 0.495747077 | -29.64250398 | 2.01E-145 | 2.54E-143 | 321.6167243 |
| LDB2 | -1.790778678 | 1.917574811 | -29.60967287 | 3.53E-145 | 4.43E-143 | 321.0548443 |
| AOC3 | -3.297085418 | 3.027841189 | -29.56618224 | 7.45E-145 | 9.27E-143 | 320.3106839 |
| SCN3A | -0.666488838 | 0.190415792 | -29.5193158 | 1.66E-144 | 2.06E-142 | 319.5089526 |
| VIT | -2.097328113 | 0.931528674 | -29.43241872 | 7.37E-144 | 9.06E-142 | 318.0229637 |
| TSHZ2 | -1.94983028 | 1.231081475 | -29.42544734 | 8.31E-144 | 1.01E-141 | 317.9037796 |
| GRIA4 | -1.023342017 | 0.243512035 | -29.38932641 | 1.54E-143 | 1.87E-141 | 317.2863225 |
| PCOLCE2 | -3.219204162 | 1.2375123 | -29.30364908 | 6.69E-143 | 8.07E-141 | 315.8222359 |
| CIDEC | -4.650254247 | 1.790622965 | -29.29181782 | 8.19E-143 | 9.82E-141 | 315.6201142 |
| AVPR2 | -1.367594769 | 0.608278711 | -29.26787517 | 1.23E-142 | 1.47E-140 | 315.2111268 |
| GNAL | -1.221363898 | 0.819249483 | -29.22371291 | 2.63E-142 | 3.11E-140 | 314.4568951 |
| LIFR | -2.352153285 | 1.993377082 | -29.17804459 | 5.74E-142 | 6.75E-140 | 313.6771437 |
| SYN2 | -1.466997836 | 0.603708984 | -29.15244036 | 8.89E-142 | 1.04E-139 | 313.2400619 |
| SPX | -2.061515243 | 0.475394567 | -29.02955665 | 7.27E-141 | 8.40E-139 | 311.14327 |
| IGFBP6 | -2.957281656 | 3.062547343 | -28.99808141 | 1.24E-140 | 1.43E-138 | 310.6064488 |
| RBMS3 | -1.652678833 | 1.333671179 | -28.91103354 | 5.50E-140 | 6.29E-138 | 309.1223524 |
| LGI4 | -1.248416938 | 0.762443003 | -28.89434342 | 7.32E-140 | 8.32E-138 | 308.8378899 |
| PDE1C | -1.237704044 | 0.440685662 | -28.89236742 | 7.57E-140 | 8.55E-138 | 308.8042134 |
| LIPE | -3.490982067 | 2.119312007 | -28.86861065 | 1.14E-139 | 1.28E-137 | 308.3993645 |
| PAFAH1B3 | 2.322029755 | 5.11911026 | 28.76829143 | 6.29E-139 | 7.03E-137 | 306.6904488 |
| LGALS12 | -2.886741159 | 1.00754885 | -28.74785417 | 8.92E-139 | 9.91E-137 | 306.342438 |
| PAK3 | -1.064179184 | 0.407551683 | -28.69771335 | 2.10E-138 | 2.32E-136 | 305.4888206 |
| GSN | -2.31442597 | 5.387568377 | -28.64122835 | 5.50E-138 | 6.04E-136 | 304.5275297 |
| DENND2A | -1.379625921 | 0.994856351 | -28.59763735 | 1.16E-137 | 1.26E-135 | 303.7859186 |
| ADH1B | -4.816715806 | 2.150193342 | -28.56548335 | 2.00E-137 | 2.17E-135 | 303.2390216 |
| MMP11 | 4.4404587 | 4.815191703 | 28.54170666 | 3.00E-137 | 3.24E-135 | 302.8346869 |
| CEP112 | -1.011035382 | 0.917444603 | -28.5321397 | 3.53E-137 | 3.79E-135 | 302.672014 |
| TMEM255A | -1.334730184 | 0.830713766 | -28.4877994 | 7.51E-137 | 8.02E-135 | 301.9182053 |
| RBP4 | -4.39884333 | 2.13056422 | -28.48141068 | 8.37E-137 | 8.89E-135 | 301.8096123 |
| GPBAR1 | -0.927346572 | 0.394790241 | -28.45830652 | 1.24E-136 | 1.31E-134 | 301.4169361 |
| HSPB2 | -1.511454927 | 0.857434982 | -28.43841801 | 1.74E-136 | 1.83E-134 | 301.078962 |
| TMEM100 | -2.062147221 | 0.828247983 | -28.42039821 | 2.37E-136 | 2.47E-134 | 300.7727836 |
| PLIN1 | -4.969609998 | 2.432035165 | -28.35981491 | 6.64E-136 | 6.90E-134 | 299.7436782 |
| C8orf88 | -1.313658645 | 0.834797774 | -28.26948544 | 3.09E-135 | 3.17E-133 | 298.2100927 |
| SAMD5 | -1.997333475 | 0.871147146 | -28.25532223 | 3.93E-135 | 4.02E-133 | 297.9697228 |
| DNASE1L3 | -1.286313559 | 0.536011372 | -28.22531059 | 6.54E-135 | 6.66E-133 | 297.4604624 |
| SHE | -1.369156622 | 1.067260111 | -28.20298478 | 9.56E-135 | 9.68E-133 | 297.0816918 |
| CIDEA | -3.286815145 | 1.08946315 | -28.19441669 | 1.11E-134 | 1.11E-132 | 296.9363453 |
| CLCA4 | -0.912036644 | 0.213266616 | -28.1063342 | 4.94E-134 | 4.93E-132 | 295.4426621 |
| PGM5 | -1.79721789 | 1.021308288 | -28.05595733 | 1.16E-133 | 1.15E-131 | 294.5888145 |
| B3GALT1 | -0.618460091 | 0.15591335 | -28.04259573 | 1.46E-133 | 1.44E-131 | 294.3623995 |
| LMOD1 | -2.67126421 | 2.775587966 | -28.03978349 | 1.53E-133 | 1.50E-131 | 294.3147483 |
| CES1 | -3.30506028 | 1.914815612 | -28.02703284 | 1.90E-133 | 1.86E-131 | 294.0987114 |
| AKR1C1 | -2.356503421 | 0.895688483 | -28.02255463 | 2.05E-133 | 1.99E-131 | 294.0228409 |
| FAM180B | -1.158343448 | 0.302306405 | -28.00285099 | 2.86E-133 | 2.77E-131 | 293.6890494 |
| C2orf40 | -3.123455653 | 1.292446879 | -27.96200704 | 5.73E-133 | 5.51E-131 | 292.9972854 |
| MYH11 | -3.263474222 | 2.162280319 | -27.96096682 | 5.83E-133 | 5.58E-131 | 292.9796702 |
| GALNT15 | -2.390769158 | 1.528657441 | -27.94457186 | 7.70E-133 | 7.34E-131 | 292.7020548 |
| RERGL | -2.207219738 | 0.854521056 | -27.94365083 | 7.82E-133 | 7.42E-131 | 292.6864601 |
| TIMP4 | -3.473128639 | 1.593715759 | -27.94278019 | 7.93E-133 | 7.49E-131 | 292.6717186 |
| F10 | -1.493013419 | 0.949509985 | -27.92642961 | 1.05E-132 | 9.84E-131 | 292.3948928 |
| CDC14B | -1.230957043 | 1.182675809 | -27.92514865 | 1.07E-132 | 1.00E-130 | 292.3732067 |
| PLXNA4 | -1.634887646 | 0.763077009 | -27.91453519 | 1.28E-132 | 1.19E-130 | 292.1935341 |
| FAM162B | -1.161839844 | 0.770903367 | -27.89277686 | 1.85E-132 | 1.72E-130 | 291.8252382 |
| GYG2 | -2.964093018 | 2.355492194 | -27.86258057 | 3.09E-132 | 2.85E-130 | 291.3142173 |
| ADH4 | -0.597940503 | 0.117821649 | -27.83673407 | 4.79E-132 | 4.40E-130 | 290.8769036 |
| MMRN1 | -2.328535166 | 1.043889507 | -27.79889144 | 9.10E-132 | 8.31E-130 | 290.2367772 |
| ANGPTL8 | -1.170829323 | 0.287179821 | -27.7173704 | 3.62E-131 | 3.29E-129 | 288.858452 |
| DACT2 | -0.844724759 | 0.230525675 | -27.61480575 | 2.06E-130 | 1.86E-128 | 287.125594 |
| PFKFB1 | -1.814286936 | 0.81724864 | -27.6122601 | 2.15E-130 | 1.92E-128 | 287.0826026 |
| PLIN4 | -4.840968656 | 2.580465313 | -27.53578789 | 7.83E-130 | 6.99E-128 | 285.7915416 |
| GPRASP1 | -1.463416295 | 1.099587967 | -27.45093658 | 3.29E-129 | 2.92E-127 | 284.3599651 |
| LRRC70 | -0.693454912 | 0.389189658 | -27.43359744 | 4.41E-129 | 3.90E-127 | 284.0675501 |
| ZBTB16 | -1.760271724 | 0.64718448 | -27.43310133 | 4.44E-129 | 3.91E-127 | 284.0591841 |
| CD36 | -4.126776557 | 2.709067967 | -27.37901513 | 1.11E-128 | 9.71E-127 | 283.1473267 |
| RNF150 | -1.873367672 | 1.063141552 | -27.35761312 | 1.59E-128 | 1.39E-126 | 282.7866176 |
| NR3C2 | -1.620584885 | 1.12308435 | -27.25421131 | 9.11E-128 | 7.84E-126 | 281.0448112 |
| MEOX2 | -2.27952417 | 1.423382918 | -27.24714208 | 1.03E-127 | 8.80E-126 | 280.9257862 |
| PYCR1 | 2.249669795 | 4.844197598 | 27.09952755 | 1.24E-126 | 1.06E-124 | 278.4420693 |
| TACR1 | -1.579642408 | 0.698076696 | -27.09144835 | 1.42E-126 | 1.20E-124 | 278.3062242 |
| HAS3 | -2.152761293 | 1.059275201 | -27.08574148 | 1.56E-126 | 1.32E-124 | 278.210274 |
| MLXIPL | -1.760696463 | 0.715336854 | -27.05850958 | 2.47E-126 | 2.08E-124 | 277.7524877 |
| SLC22A3 | -1.479145419 | 0.66599481 | -27.05726425 | 2.52E-126 | 2.11E-124 | 277.7315555 |
| MGARP | -0.761446597 | 0.351027529 | -26.98814388 | 8.07E-126 | 6.74E-124 | 276.5701113 |
| SPTBN1 | -1.769061568 | 4.167769638 | -26.96184889 | 1.26E-125 | 1.04E-123 | 276.1284615 |
| PQLC2L | -1.169131381 | 0.48269921 | -26.93461611 | 1.99E-125 | 1.64E-123 | 275.6711722 |
| HOXA5 | -2.153084819 | 1.430806484 | -26.91253364 | 2.88E-125 | 2.37E-123 | 275.3004498 |
| CACHD1 | -1.879196277 | 1.73245477 | -26.91022471 | 2.99E-125 | 2.46E-123 | 275.2616915 |
| TCEAL7 | -1.690020059 | 1.337593113 | -26.87863688 | 5.09E-125 | 4.16E-123 | 274.7315343 |
| LIMS2 | -1.647415902 | 1.710316878 | -26.86297009 | 6.63E-125 | 5.40E-123 | 274.4686467 |
| ANKRD29 | -1.505636607 | 0.852690879 | -26.85396142 | 7.71E-125 | 6.25E-123 | 274.3174991 |
| FGF2 | -2.245130626 | 1.225057065 | -26.7801801 | 2.66E-124 | 2.15E-122 | 273.0800727 |
| CD34 | -1.89769329 | 3.103071176 | -26.74350063 | 4.93E-124 | 3.95E-122 | 272.4652201 |
| MTURN | -2.133817054 | 2.910261208 | -26.71059035 | 8.58E-124 | 6.84E-122 | 271.9137317 |
| JAM2 | -1.830015757 | 1.944639433 | -26.69971357 | 1.03E-123 | 8.18E-122 | 271.7315038 |
| PID1 | -1.861766045 | 1.395700874 | -26.68598909 | 1.30E-123 | 1.03E-121 | 271.5015929 |
| FAM149A | -1.063616137 | 0.733254011 | -26.67142072 | 1.66E-123 | 1.30E-121 | 271.2575784 |
| OVCH2 | -0.955458344 | 0.264356793 | -26.6349183 | 3.05E-123 | 2.40E-121 | 270.6463267 |
| CXorf36 | -1.713279711 | 2.035230863 | -26.57446126 | 8.42E-123 | 6.58E-121 | 269.6344168 |
| SYNM | -3.590203868 | 2.60735454 | -26.57143572 | 8.86E-123 | 6.90E-121 | 269.5837919 |
| PEAR1 | -1.479471372 | 1.287622541 | -26.56837727 | 9.32E-123 | 7.23E-121 | 269.5326179 |
| P2RY12 | -1.500209859 | 0.805138984 | -26.54616995 | 1.35E-122 | 1.05E-120 | 269.1610906 |
| DST | -2.296225522 | 2.500817361 | -26.53322177 | 1.68E-122 | 1.29E-120 | 268.9445056 |
| GHR | -2.435767477 | 1.875755913 | -26.51885906 | 2.14E-122 | 1.64E-120 | 268.7042918 |
| PLPP3 | -1.902459833 | 3.694457119 | -26.51312168 | 2.35E-122 | 1.80E-120 | 268.6083445 |
| ACADL | -1.294354338 | 0.354731461 | -26.48987793 | 3.48E-122 | 2.64E-120 | 268.2196909 |
| NTRK2 | -2.968577902 | 1.680308769 | -26.44925559 | 6.86E-122 | 5.20E-120 | 267.5406684 |
| MOCS1 | -1.578056231 | 2.025824539 | -26.43208172 | 9.15E-122 | 6.90E-120 | 267.2536812 |
| ENPP2 | -2.604998298 | 2.391061688 | -26.42382775 | 1.05E-121 | 7.90E-120 | 267.1157691 |
| CKS2 | 2.364939576 | 5.35869613 | 26.4168661 | 1.18E-121 | 8.84E-120 | 266.9994588 |
| CPED1 | -1.935399759 | 1.326568821 | -26.40819594 | 1.36E-121 | 1.02E-119 | 266.8546152 |
| BHMT2 | -1.861577842 | 1.126538982 | -26.38246716 | 2.10E-121 | 1.56E-119 | 266.4248646 |
| PDE3B | -1.707823277 | 0.754947797 | -26.37627437 | 2.33E-121 | 1.72E-119 | 266.3214425 |
| CA3 | -2.456202791 | 0.929982246 | -26.3589011 | 3.11E-121 | 2.30E-119 | 266.0313361 |
| HBB | -3.753967488 | 2.538928406 | -26.35513357 | 3.32E-121 | 2.44E-119 | 265.968431 |
| PPP1R1A | -3.200602474 | 1.636651188 | -26.3537593 | 3.39E-121 | 2.48E-119 | 265.9454858 |
| RSPO3 | -1.795654817 | 1.117918272 | -26.34678011 | 3.81E-121 | 2.78E-119 | 265.8289646 |
| TAL1 | -0.886499932 | 0.614809363 | -26.34412123 | 3.99E-121 | 2.90E-119 | 265.7845754 |
| CAT | -1.728819451 | 4.771080861 | -26.33726024 | 4.47E-121 | 3.24E-119 | 265.6700389 |
| GNAI1 | -1.967267238 | 1.980256473 | -26.33202366 | 4.88E-121 | 3.52E-119 | 265.5826253 |
| ADIPOQ | -4.660281454 | 2.061010886 | -26.32980199 | 5.07E-121 | 3.64E-119 | 265.5455407 |
| SH3D19 | -1.759707985 | 3.160838535 | -26.27191813 | 1.33E-120 | 9.52E-119 | 264.579626 |
| FHL5 | -1.505246924 | 0.880240257 | -26.22568153 | 2.89E-120 | 2.05E-118 | 263.8084825 |
| NEK2 | 2.501228936 | 2.774082573 | 26.2125688 | 3.60E-120 | 2.55E-118 | 263.5898528 |
| RELN | -1.200276286 | 0.37602151 | -26.18874532 | 5.36E-120 | 3.78E-118 | 263.1927177 |
| FAM126A | -1.392003118 | 1.19792294 | -26.09292406 | 2.65E-119 | 1.86E-117 | 261.5963856 |
| EDNRB | -1.853960802 | 1.651915837 | -26.05775193 | 4.77E-119 | 3.32E-117 | 261.0108408 |
| COL10A1 | 4.266475302 | 4.199812111 | 26.04305769 | 6.09E-119 | 4.22E-117 | 260.7662763 |
| PDZD2 | -1.781782298 | 1.373851782 | -26.04057612 | 6.35E-119 | 4.39E-117 | 260.7249779 |
| CCDC69 | -2.363254753 | 2.423358754 | -26.01231459 | 1.02E-118 | 6.98E-117 | 260.2547256 |
| PREX2 | -1.300696814 | 0.87848424 | -26.00833789 | 1.09E-118 | 7.43E-117 | 260.1885675 |
| PRRT4 | -1.17484978 | 0.365274494 | -25.99779746 | 1.30E-118 | 8.83E-117 | 260.0132259 |
| CLDN11 | -1.970959841 | 1.226211557 | -25.9547059 | 2.66E-118 | 1.80E-116 | 259.2965989 |
| TMOD1 | -1.489376444 | 0.703838435 | -25.92225258 | 4.56E-118 | 3.09E-116 | 258.7571099 |
| KCNB1 | -1.277547021 | 0.42375093 | -25.90458245 | 6.13E-118 | 4.13E-116 | 258.4634498 |
| SNCA | -0.948603579 | 0.721681427 | -25.88187709 | 8.94E-118 | 6.00E-116 | 258.0861923 |
| ASF1B | 2.251888976 | 3.27933956 | 25.82537876 | 2.29E-117 | 1.53E-115 | 257.1478609 |
| SLC35G2 | -1.034914098 | 0.832902039 | -25.76653953 | 6.09E-117 | 4.06E-115 | 256.1712746 |
| SEMA3G | -2.486338765 | 2.490066081 | -25.7003359 | 1.83E-116 | 1.21E-114 | 255.0732244 |
| PRKN | -0.910463578 | 0.672600199 | -25.65138227 | 4.13E-116 | 2.73E-114 | 254.2618088 |
| PLSCR4 | -1.958049494 | 2.535398839 | -25.61650303 | 7.37E-116 | 4.83E-114 | 253.6839541 |
| UBE2T | 2.537537183 | 4.024170541 | 25.61119622 | 8.05E-116 | 5.26E-114 | 253.5960549 |
| GNG11 | -1.979810436 | 3.172557692 | -25.60430124 | 9.02E-116 | 5.88E-114 | 253.4818579 |
| MATN2 | -2.781110495 | 2.346888064 | -25.59407191 | 1.07E-115 | 6.94E-114 | 253.3124529 |
| PDK4 | -3.54114973 | 3.18237651 | -25.58016671 | 1.35E-115 | 8.71E-114 | 253.0822049 |
| ANGPT1 | -1.472656 | 0.731302474 | -25.55650821 | 1.99E-115 | 1.29E-113 | 252.6905425 |
| PHYHIP | -1.029825333 | 0.514386564 | -25.54749797 | 2.32E-115 | 1.49E-113 | 252.5414075 |
| FZD4 | -2.117682825 | 2.885011931 | -25.53431449 | 2.88E-115 | 1.85E-113 | 252.3232263 |
| AC007906.2 | -2.409277072 | 1.179173909 | -25.52164479 | 3.55E-115 | 2.27E-113 | 252.1135794 |
| PDE11A | -0.668108023 | 0.291939455 | -25.52142378 | 3.57E-115 | 2.27E-113 | 252.1099226 |
| INMT | -1.779786484 | 1.131239047 | -25.48794859 | 6.22E-115 | 3.93E-113 | 251.5561551 |
| SAA2-SAA4 | -3.054023626 | 1.289773203 | -25.48518006 | 6.51E-115 | 4.10E-113 | 251.5103659 |
| CNN1 | -3.118103772 | 2.839548425 | -25.46146217 | 9.64E-115 | 6.06E-113 | 251.1181533 |
| FAM107A | -2.439759971 | 1.265440043 | -25.45090931 | 1.15E-114 | 7.19E-113 | 250.9436803 |
| C2orf88 | -1.349939522 | 0.663684447 | -25.42032179 | 1.91E-114 | 1.19E-112 | 250.4380914 |
| CEP68 | -1.05181937 | 2.435914312 | -25.40067771 | 2.64E-114 | 1.64E-112 | 250.1134856 |
| PYGM | -1.093017332 | 0.363057446 | -25.35667229 | 5.47E-114 | 3.37E-112 | 249.3865988 |
| APCDD1 | -2.027459737 | 2.145674578 | -25.34792608 | 6.32E-114 | 3.88E-112 | 249.2421732 |
| TK1 | 2.624265568 | 4.654782124 | 25.34586196 | 6.54E-114 | 4.01E-112 | 249.2080906 |
| GPLD1 | -0.864866733 | 0.395152812 | -25.34208533 | 6.96E-114 | 4.25E-112 | 249.1457335 |
| MYMX | -1.461834555 | 0.634860751 | -25.33569783 | 7.74E-114 | 4.71E-112 | 249.0402741 |
| PLAC9 | -2.634803108 | 2.14309366 | -25.3235762 | 9.45E-114 | 5.74E-112 | 248.8401646 |
| HBA2 | -2.826577547 | 1.369234611 | -25.29236715 | 1.58E-113 | 9.55E-112 | 248.3250849 |
| RASSF9 | -1.123989343 | 0.690958804 | -25.25149994 | 3.11E-113 | 1.87E-111 | 247.6508982 |
| ST6GALNAC3 | -0.708228626 | 0.490178484 | -25.20948445 | 6.23E-113 | 3.73E-111 | 246.9581165 |
| CNTNAP3 | -0.630610811 | 0.24064901 | -25.1869844 | 9.04E-113 | 5.39E-111 | 246.5872654 |
| TSPAN7 | -2.289390407 | 2.012442432 | -25.17381335 | 1.12E-112 | 6.67E-111 | 246.3702243 |
| SRPX | -2.521297876 | 3.099806744 | -25.17355473 | 1.13E-112 | 6.68E-111 | 246.365963 |
| PPP1R14A | -2.218252958 | 1.597834673 | -25.15827926 | 1.45E-112 | 8.57E-111 | 246.1142886 |
| NUSAP1 | 2.406913977 | 3.62643515 | 25.13980368 | 1.97E-112 | 1.16E-110 | 245.8099535 |
| EBF2 | -1.046540566 | 0.685103197 | -25.13090876 | 2.28E-112 | 1.34E-110 | 245.6634585 |
| ANK2 | -1.280047488 | 0.874131775 | -25.11309813 | 3.06E-112 | 1.79E-110 | 245.3701745 |
| ACSS2 | -1.363312301 | 3.255890097 | -25.08781487 | 4.65E-112 | 2.71E-110 | 244.9539514 |
| USHBP1 | -0.864912228 | 0.632746496 | -25.07051616 | 6.18E-112 | 3.59E-110 | 244.6692485 |
| HOXA6 | -1.121103472 | 0.497203473 | -25.03649728 | 1.08E-111 | 6.28E-110 | 244.1095435 |
| ROBO4 | -1.484841807 | 2.008662306 | -24.98651247 | 2.47E-111 | 1.43E-109 | 243.2875881 |
| IGF1 | -0.974176812 | 0.493590287 | -24.96319827 | 3.62E-111 | 2.09E-109 | 242.9043841 |
| CREB5 | -1.100385704 | 0.707914415 | -24.96303172 | 3.63E-111 | 2.09E-109 | 242.901647 |
| DDR2 | -2.132247222 | 2.504080717 | -24.95681237 | 4.03E-111 | 2.31E-109 | 242.799442 |
| CASQ2 | -1.512729744 | 0.747377031 | -24.93814877 | 5.47E-111 | 3.13E-109 | 242.4927842 |
| EEPD1 | -1.29671309 | 1.750563421 | -24.8927541 | 1.16E-110 | 6.58E-109 | 241.7472178 |
| TPM3 | 1.171569325 | 5.810386674 | 24.88185942 | 1.38E-110 | 7.85E-109 | 241.568347 |
| KLHL13 | -1.945549263 | 1.089143294 | -24.87997018 | 1.43E-110 | 8.08E-109 | 241.5373316 |
| UBE2C | 3.209028807 | 4.198472477 | 24.84577863 | 2.50E-110 | 1.41E-108 | 240.976145 |
| TCEAL5 | -1.250713665 | 0.459742965 | -24.82686696 | 3.42E-110 | 1.92E-108 | 240.6658535 |
| CKMT2 | -1.217682143 | 0.706479398 | -24.82610459 | 3.46E-110 | 1.94E-108 | 240.6533467 |
| F8 | -1.276973136 | 1.606790261 | -24.81632489 | 4.06E-110 | 2.27E-108 | 240.4929185 |
| DSC1 | -0.630878701 | 0.207004703 | -24.78469317 | 6.83E-110 | 3.82E-108 | 239.9741652 |
| ANXA1 | -2.54780341 | 4.920491222 | -24.7783584 | 7.58E-110 | 4.22E-108 | 239.8703018 |
| IL33 | -2.861335012 | 2.001024846 | -24.75224925 | 1.16E-109 | 6.46E-108 | 239.4423138 |
| NMT2 | -1.478408949 | 2.026931205 | -24.72899139 | 1.71E-109 | 9.45E-108 | 239.061188 |
| TPX2 | 2.74247413 | 3.919356193 | 24.69878275 | 2.80E-109 | 1.55E-107 | 238.566334 |
| TGFBR2 | -2.259442258 | 4.498317703 | -24.64618183 | 6.64E-109 | 3.65E-107 | 237.7051387 |
| FOXO1 | -1.662006928 | 2.592045357 | -24.64070302 | 7.27E-109 | 3.98E-107 | 237.6154728 |
| DTX1 | -1.529697394 | 1.245114495 | -24.63253213 | 8.31E-109 | 4.54E-107 | 237.4817609 |
| SLIT3 | -2.092831025 | 2.186407879 | -24.62999405 | 8.66E-109 | 4.71E-107 | 237.4402294 |
| FGF1 | -1.85660066 | 1.861589442 | -24.60856808 | 1.23E-108 | 6.68E-107 | 237.0896864 |
| NNAT | -2.235575644 | 1.158070158 | -24.59354591 | 1.57E-108 | 8.52E-107 | 236.8439736 |
| CCDC3 | -2.246908543 | 2.566878576 | -24.5847126 | 1.82E-108 | 9.82E-107 | 236.6995133 |
| EGFLAM | -1.339923586 | 1.576445911 | -24.57959532 | 1.98E-108 | 1.07E-106 | 236.6158328 |
| GPX3 | -3.303314555 | 4.215531984 | -24.55083215 | 3.17E-108 | 1.70E-106 | 236.1455894 |
| MAOA | -3.051660268 | 2.122729123 | -24.54208774 | 3.66E-108 | 1.96E-106 | 236.0026651 |
| NACC1 | 1.303325602 | 4.434717455 | 24.51002087 | 6.19E-108 | 3.30E-106 | 235.4786875 |
| ODF3L1 | -0.749761191 | 0.364807031 | -24.50576033 | 6.63E-108 | 3.53E-106 | 235.4090868 |
| PI16 | -3.49361434 | 1.693395104 | -24.49681829 | 7.68E-108 | 4.08E-106 | 235.2630215 |
| GSTM5 | -1.802653806 | 0.720110097 | -24.49559772 | 7.83E-108 | 4.15E-106 | 235.2430854 |
| MYCT1 | -1.619315763 | 2.045656675 | -24.4878277 | 8.90E-108 | 4.70E-106 | 235.1161812 |
| SLC50A1 | 1.702390134 | 5.176123901 | 24.47812338 | 1.04E-107 | 5.49E-106 | 234.9577041 |
| AKAP12 | -2.232013222 | 2.246428914 | -24.43763404 | 2.02E-107 | 1.06E-105 | 234.2967162 |
| MRAS | -2.044607767 | 2.663141475 | -24.33018421 | 1.17E-106 | 6.14E-105 | 232.5443857 |
| ZWINT | 2.099966951 | 3.852074653 | 24.31855961 | 1.42E-106 | 7.40E-105 | 232.3549642 |
| ADGRD1 | -0.784872193 | 0.352855029 | -24.31069458 | 1.61E-106 | 8.39E-105 | 232.226822 |
| COL25A1 | -0.610112335 | 0.20242325 | -24.29652315 | 2.03E-106 | 1.05E-104 | 231.9959672 |
| RHOXF1 | -0.651768679 | 0.277192463 | -24.27919631 | 2.69E-106 | 1.39E-104 | 231.7137728 |
| LHFPL6 | -2.078991574 | 4.018554839 | -24.19611654 | 1.04E-105 | 5.37E-104 | 230.3616475 |
| PROS1 | -1.949685251 | 2.513852964 | -24.17665339 | 1.43E-105 | 7.36E-104 | 230.0451145 |
| KCNMB1 | -1.766125445 | 1.12094973 | -24.15661196 | 1.99E-105 | 1.02E-103 | 229.7192687 |
| AKR1C2 | -2.702383425 | 1.136599184 | -24.15083541 | 2.18E-105 | 1.11E-103 | 229.6253674 |
| NPY2R | -1.54163043 | 0.314157208 | -24.12707223 | 3.22E-105 | 1.64E-103 | 229.2391644 |
| CCNB1 | 2.076064448 | 4.036179706 | 24.07743019 | 7.22E-105 | 3.66E-103 | 228.4328001 |
| TLN2 | -1.331016556 | 1.794109802 | -24.05082573 | 1.11E-104 | 5.63E-103 | 228.0008866 |
| FABP4 | -5.549233988 | 4.014077096 | -24.04884767 | 1.15E-104 | 5.80E-103 | 227.9687803 |
| KANK1 | -1.615286648 | 2.183176898 | -24.01146107 | 2.11E-104 | 1.06E-102 | 227.3621227 |
| MEOX1 | -2.275678999 | 1.446724365 | -23.99598347 | 2.71E-104 | 1.36E-102 | 227.1110709 |
| WASF3 | -1.719081252 | 1.584806665 | -23.9833294 | 3.33E-104 | 1.67E-102 | 226.9058598 |
| RUNX1T1 | -0.834591553 | 0.604838442 | -23.93564649 | 7.24E-104 | 3.59E-102 | 226.1329293 |
| CSRNP3 | -0.804886575 | 0.370415216 | -23.93135757 | 7.76E-104 | 3.84E-102 | 226.0634335 |
| CLIC5 | -1.167474025 | 0.817364749 | -23.9096705 | 1.10E-103 | 5.45E-102 | 225.7120926 |
| CCDC50 | -1.123587791 | 3.051819707 | -23.87638667 | 1.89E-103 | 9.32E-102 | 225.1730988 |
| SH3BGRL2 | -1.870478264 | 2.30530634 | -23.8559508 | 2.64E-103 | 1.30E-101 | 224.8422956 |
| TMEM37 | -2.213456249 | 2.392665965 | -23.84567822 | 3.12E-103 | 1.53E-101 | 224.6760476 |
| PLPP1 | -1.706600606 | 3.959960454 | -23.83588928 | 3.65E-103 | 1.78E-101 | 224.5176505 |
| SYNPO2 | -2.23324434 | 2.075494046 | -23.82572469 | 4.31E-103 | 2.10E-101 | 224.3531994 |
| RNASE7 | -1.008967303 | 0.351022588 | -23.82273984 | 4.52E-103 | 2.20E-101 | 224.3049129 |
| SLC7A3 | -1.139209301 | 0.359197693 | -23.79036094 | 7.64E-103 | 3.71E-101 | 223.7812517 |
| SLC35A2 | 1.153900178 | 4.068070777 | 23.77798937 | 9.34E-103 | 4.52E-101 | 223.5812348 |
| LDLRAD2 | -0.645588058 | 0.477597096 | -23.74141831 | 1.69E-102 | 8.15E-101 | 222.990193 |
| PCLAF | 1.865801959 | 2.298985149 | 23.66246331 | 6.06E-102 | 2.90E-100 | 221.7152866 |
| CMA1 | -1.637964846 | 0.717166377 | -23.58686862 | 2.06E-101 | 9.83E-100 | 220.4960897 |
| ALDH1A1 | -2.527111549 | 3.04268996 | -23.53271029 | 4.93E-101 | 2.35E-99 | 219.6234995 |
| ITSN1 | -1.073276994 | 1.653376861 | -23.53149223 | 5.03E-101 | 2.39E-99 | 219.6038828 |
| TDRD10 | -0.765423623 | 0.423495363 | -23.51189988 | 6.90E-101 | 3.27E-99 | 219.2884021 |
| H2AFY | 0.950554298 | 3.893796774 | 23.49996055 | 8.37E-101 | 3.94E-99 | 219.0961997 |
| DMRT2 | -0.904746608 | 0.390435439 | -23.48756089 | 1.02E-100 | 4.80E-99 | 218.896625 |
| MYLK | -2.180682817 | 2.635750677 | -23.46996194 | 1.36E-100 | 6.36E-99 | 218.6134339 |
| MARC1 | -1.678694033 | 1.568159712 | -23.45867031 | 1.63E-100 | 7.62E-99 | 218.4317777 |
| SEMA3D | -1.234775346 | 0.681809979 | -23.45013847 | 1.87E-100 | 8.72E-99 | 218.2945416 |
| PCDHGB7 | -1.039093548 | 0.860116913 | -23.44577134 | 2.01E-100 | 9.33E-99 | 218.2243028 |
| CDK1 | 2.230934256 | 3.213116271 | 23.42395325 | 2.85E-100 | 1.32E-98 | 217.873464 |
| CLMP | -2.077309099 | 2.822183182 | -23.40549419 | 3.84E-100 | 1.78E-98 | 217.576734 |
| S100B | -3.433639058 | 2.085590352 | -23.36645675 | 7.20E-100 | 3.32E-98 | 216.9494939 |
| G0S2 | -3.783128461 | 3.384527519 | -23.35699562 | 8.38E-100 | 3.86E-98 | 216.7975347 |
| ABCB1 | -1.270710561 | 0.89334639 | -23.26988935 | 3.40E-99 | 1.56E-97 | 215.399573 |
| TTC28 | -1.123296217 | 1.566092964 | -23.26928726 | 3.44E-99 | 1.57E-97 | 215.389917 |
| GIMAP8 | -1.590822496 | 2.082542389 | -23.25659702 | 4.22E-99 | 1.92E-97 | 215.1864188 |
| KCNAB1 | -0.952197088 | 0.763513267 | -23.25296586 | 4.47E-99 | 2.04E-97 | 215.128198 |
| NDRG2 | -2.429831777 | 3.460883108 | -23.2436632 | 5.19E-99 | 2.36E-97 | 214.979058 |
| CLEC1A | -0.945376146 | 0.914045725 | -23.22591084 | 6.90E-99 | 3.13E-97 | 214.6945156 |
| CYP2U1 | -1.036332363 | 1.442952833 | -23.20968768 | 8.96E-99 | 4.05E-97 | 214.434556 |
| ACO1 | -1.413760892 | 2.949363088 | -23.20250852 | 1.01E-98 | 4.54E-97 | 214.3195393 |
| EMCN | -1.710361328 | 2.063695836 | -23.19118262 | 1.21E-98 | 5.43E-97 | 214.1381156 |
| SMIM10L2A | -1.276786332 | 0.741434316 | -23.1867842 | 1.29E-98 | 5.81E-97 | 214.0676688 |
| ERG | -1.293895791 | 1.631894947 | -23.11535167 | 4.07E-98 | 1.82E-96 | 212.9242919 |
| KIF4A | 2.113981176 | 2.417557567 | 23.09124079 | 5.99E-98 | 2.68E-96 | 212.5386692 |
| METTL7A | -1.79782333 | 4.197796804 | -23.09050755 | 6.06E-98 | 2.70E-96 | 212.5269444 |
| KLF4 | -2.329775268 | 3.060440485 | -23.08977379 | 6.13E-98 | 2.73E-96 | 212.5152115 |
| TMTC1 | -1.950241415 | 1.409206814 | -23.0267179 | 1.68E-97 | 7.46E-96 | 211.5074716 |
| GABARAPL1 | -1.41601707 | 3.549509559 | -23.00413015 | 2.42E-97 | 1.07E-95 | 211.1467406 |
| HOXA2 | -0.762606816 | 0.340266844 | -23.00213883 | 2.50E-97 | 1.10E-95 | 211.1149454 |
| ADGRF5 | -1.63882498 | 2.685524288 | -22.99412271 | 2.84E-97 | 1.25E-95 | 210.9869636 |
| CYYR1 | -1.897203545 | 2.725521522 | -22.99337094 | 2.87E-97 | 1.26E-95 | 210.9749622 |
| PELI2 | -1.288950712 | 1.227669578 | -22.98769509 | 3.15E-97 | 1.38E-95 | 210.8843557 |
| SPC25 | 1.566311635 | 2.083597015 | 22.93072237 | 7.82E-97 | 3.42E-95 | 209.9753546 |
| TGFBR3 | -2.751669613 | 2.51779575 | -22.91926712 | 9.40E-97 | 4.09E-95 | 209.792692 |
| PIK3C2G | -1.678233142 | 0.732912566 | -22.90886628 | 1.11E-96 | 4.82E-95 | 209.6268738 |
| THSD1 | -1.085387571 | 1.442882822 | -22.89811502 | 1.32E-96 | 5.71E-95 | 209.4555 |
| NRN1 | -2.103048172 | 2.598761548 | -22.89533793 | 1.38E-96 | 5.96E-95 | 209.4112385 |
| XPNPEP2 | -1.023619412 | 0.442575708 | -22.87999752 | 1.76E-96 | 7.59E-95 | 209.1667805 |
| CLDN5 | -2.224616193 | 1.876440988 | -22.84336817 | 3.16E-96 | 1.36E-94 | 208.5833315 |
| NATD1 | -1.373325175 | 2.235623371 | -22.81140175 | 5.26E-96 | 2.26E-94 | 208.0744571 |
| MCAM | -1.781026848 | 4.004638012 | -22.78484178 | 8.04E-96 | 3.44E-94 | 207.6518623 |
| RUNDC3B | -0.698973214 | 0.521442071 | -22.76084694 | 1.18E-95 | 5.04E-94 | 207.2702488 |
| ITM2A | -2.56589224 | 3.295600456 | -22.72782099 | 1.99E-95 | 8.49E-94 | 206.7452663 |
| AOC2 | -0.849255698 | 0.492672357 | -22.68748773 | 3.79E-95 | 1.61E-93 | 206.1045385 |
| FERMT2 | -1.599761867 | 2.791533697 | -22.68518306 | 3.93E-95 | 1.67E-93 | 206.0679406 |
| WDR86 | -1.272260023 | 0.840164484 | -22.68310212 | 4.06E-95 | 1.72E-93 | 206.0348967 |
| CDKN1C | -1.942579776 | 2.576684193 | -22.67776377 | 4.42E-95 | 1.87E-93 | 205.9501332 |
| POC1A | 1.419274865 | 2.523486667 | 22.65760545 | 6.10E-95 | 2.57E-93 | 205.6301268 |
| MMD | -1.929844044 | 2.821250716 | -22.64637743 | 7.29E-95 | 3.06E-93 | 205.4519353 |
| EZH1 | -1.169541288 | 2.721944032 | -22.64451006 | 7.51E-95 | 3.15E-93 | 205.422303 |
| EOGT | -1.1934261 | 2.29439318 | -22.63152506 | 9.23E-95 | 3.86E-93 | 205.2162789 |
| LMNB1 | 2.017098518 | 3.814204377 | 22.62833543 | 9.72E-95 | 4.06E-93 | 205.1656785 |
| MMRN2 | -1.742820193 | 2.903811554 | -22.61981226 | 1.11E-94 | 4.64E-93 | 205.0304807 |
| FOXN3 | -1.353601351 | 2.508722244 | -22.60776826 | 1.35E-94 | 5.58E-93 | 204.839469 |
| CLEC4G | -0.645099814 | 0.167848641 | -22.60586145 | 1.39E-94 | 5.74E-93 | 204.8092317 |
| TMEM88 | -1.516985866 | 1.536575584 | -22.60049041 | 1.51E-94 | 6.24E-93 | 204.7240658 |
| RNF186 | -0.904399244 | 0.236227551 | -22.59368714 | 1.69E-94 | 6.93E-93 | 204.6162014 |
| LTBP4 | -1.746717555 | 2.807409549 | -22.58208015 | 2.03E-94 | 8.30E-93 | 204.4322056 |
| EPAS1 | -1.833308816 | 4.445415343 | -22.57027426 | 2.44E-94 | 1.00E-92 | 204.2450958 |
| NR3C1 | -1.597218357 | 2.864014494 | -22.56659018 | 2.59E-94 | 1.06E-92 | 204.1867156 |
| TXNIP | -2.103619448 | 7.240543141 | -22.56302677 | 2.74E-94 | 1.12E-92 | 204.130251 |
| FOSB | -3.683427312 | 2.769900388 | -22.52370183 | 5.12E-94 | 2.08E-92 | 203.5073628 |
| TOP2A | 2.797773733 | 3.9963344 | 22.52255472 | 5.22E-94 | 2.11E-92 | 203.4891998 |
| MRGPRF | -1.618822157 | 1.540425272 | -22.49949233 | 7.52E-94 | 3.04E-92 | 203.1241153 |
| ANKDD1A | -0.741605257 | 0.888510238 | -22.48958078 | 8.80E-94 | 3.55E-92 | 202.9672592 |
| EPHB1 | -1.359681385 | 0.68026871 | -22.48757283 | 9.09E-94 | 3.66E-92 | 202.9354857 |
| PTTG1 | 2.22278654 | 3.18932689 | 22.47511417 | 1.11E-93 | 4.45E-92 | 202.7383672 |
| DNAJB4 | -1.281590073 | 2.629974141 | -22.45279488 | 1.58E-93 | 6.33E-92 | 202.3853473 |
| CCM2L | -1.095237498 | 1.249123956 | -22.44568157 | 1.77E-93 | 7.07E-92 | 202.2728675 |
| RBMS2 | -1.005256717 | 2.51037498 | -22.4425918 | 1.85E-93 | 7.41E-92 | 202.2240149 |
| ABCC9 | -1.265028132 | 1.055979184 | -22.42527082 | 2.44E-93 | 9.73E-92 | 201.9502019 |
| SOX7 | -1.317681536 | 1.338347138 | -22.39483264 | 3.95E-93 | 1.57E-91 | 201.4692401 |
| PTH1R | -1.202910074 | 0.797745261 | -22.38804371 | 4.40E-93 | 1.75E-91 | 201.3620029 |
| CCDC167 | 1.637756548 | 4.540215868 | 22.36684414 | 6.16E-93 | 2.44E-91 | 201.0272231 |
| TBX15 | -1.435472974 | 1.484843165 | -22.36182135 | 6.66E-93 | 2.64E-91 | 200.9479233 |
| ISM1 | -2.158663105 | 2.259025934 | -22.35849943 | 7.02E-93 | 2.77E-91 | 200.8954808 |
| AQP1 | -2.32901736 | 5.176083666 | -22.35828071 | 7.05E-93 | 2.78E-91 | 200.892028 |
| ROBO3 | -0.930179817 | 0.692636174 | -22.34295824 | 8.98E-93 | 3.53E-91 | 200.6501777 |
| SOX17 | -1.447830435 | 1.314034692 | -22.33779817 | 9.75E-93 | 3.83E-91 | 200.5687465 |
| AASS | -1.22155079 | 1.177841678 | -22.3239428 | 1.21E-92 | 4.75E-91 | 200.3501328 |
| ADCY4 | -1.229946168 | 1.224305729 | -22.30900645 | 1.54E-92 | 6.01E-91 | 200.114526 |
| PER1 | -1.978432252 | 2.60661181 | -22.30515758 | 1.63E-92 | 6.37E-91 | 200.0538242 |
| GPR17 | -0.613501955 | 0.237259119 | -22.27752562 | 2.53E-92 | 9.84E-91 | 199.6181595 |
| LILRB5 | -1.122220572 | 0.609944869 | -22.27642109 | 2.57E-92 | 9.99E-91 | 199.6007494 |
| BGN | 2.282938405 | 7.803761083 | 22.2674775 | 2.96E-92 | 1.15E-90 | 199.4597893 |
| NAT8L | -2.082706924 | 1.348971969 | -22.26195677 | 3.23E-92 | 1.25E-90 | 199.3727888 |
| NSG1 | -1.381858396 | 0.626613169 | -22.25909385 | 3.38E-92 | 1.31E-90 | 199.3276759 |
| MAP7D3 | -1.222075008 | 1.216788602 | -22.24564657 | 4.18E-92 | 1.61E-90 | 199.1158106 |
| AADAC | -1.15101776 | 0.263788258 | -22.23006224 | 5.35E-92 | 2.05E-90 | 198.8703424 |
| ARHGEF15 | -1.427714306 | 1.896757505 | -22.21130936 | 7.19E-92 | 2.76E-90 | 198.5750614 |
| KIT | -2.981681045 | 2.515983429 | -22.20020343 | 8.57E-92 | 3.28E-90 | 198.4002377 |
| ADH5 | -0.845574943 | 4.444524886 | -22.19103408 | 9.91E-92 | 3.78E-90 | 198.2559261 |
| NTF4 | -0.912493165 | 0.375995974 | -22.16446925 | 1.51E-91 | 5.74E-90 | 197.8379771 |
| NRG2 | -1.08986435 | 0.457293802 | -22.15893801 | 1.64E-91 | 6.25E-90 | 197.7509794 |
| GPC3 | -3.127374056 | 2.759748909 | -22.13139292 | 2.54E-91 | 9.63E-90 | 197.3178749 |
| IL11RA | -1.222565435 | 1.63218123 | -22.13068852 | 2.57E-91 | 9.72E-90 | 197.3068023 |
| RRM2 | 2.339913247 | 3.064703938 | 22.11665159 | 3.20E-91 | 1.21E-89 | 197.0861831 |
| ECM2 | -2.061395726 | 2.431662083 | -22.11270391 | 3.41E-91 | 1.28E-89 | 197.0241477 |
| GYPC | -1.736053298 | 2.792123259 | -22.10332965 | 3.95E-91 | 1.49E-89 | 196.8768559 |
| TMEM273 | -1.331447082 | 1.427384732 | -22.09915056 | 4.22E-91 | 1.58E-89 | 196.8112009 |
| RHOJ | -1.457585133 | 2.191426786 | -22.06721565 | 6.97E-91 | 2.61E-89 | 196.3096656 |
| ADHFE1 | -1.282585338 | 1.197986797 | -22.05912408 | 7.92E-91 | 2.96E-89 | 196.1826368 |
| KIFC1 | 2.164313697 | 3.078779875 | 22.05710459 | 8.17E-91 | 3.05E-89 | 196.1509361 |
| FAM49A | -1.374790087 | 1.793766364 | -22.03809783 | 1.10E-90 | 4.11E-89 | 195.8526399 |
| IQANK1 | 1.900989508 | 3.873092643 | 22.03563887 | 1.15E-90 | 4.26E-89 | 195.8140565 |
| SLC25A27 | -1.407046391 | 0.975086468 | -22.03188581 | 1.22E-90 | 4.52E-89 | 195.7551707 |
| DTL | 1.864541113 | 2.491391662 | 22.00723251 | 1.79E-90 | 6.64E-89 | 195.3684653 |
| RNASEH2A | 1.487843977 | 3.879256726 | 22.00652894 | 1.81E-90 | 6.70E-89 | 195.357432 |
| MAML2 | -1.828296404 | 2.151942275 | -21.98767421 | 2.44E-90 | 9.00E-89 | 195.0618097 |
| CD209 | -1.476964028 | 0.89490766 | -21.98051772 | 2.73E-90 | 1.01E-88 | 194.9496318 |
| RABIF | 1.015614426 | 3.396851731 | 21.97171824 | 3.13E-90 | 1.15E-88 | 194.8117214 |
| ANTXR2 | -1.394639068 | 2.273955648 | -21.94136633 | 5.04E-90 | 1.85E-88 | 194.3362097 |
| DPT | -3.217151643 | 4.098047871 | -21.92109368 | 6.93E-90 | 2.54E-88 | 194.0187624 |
| KIF20A | 2.118990236 | 2.650798627 | 21.91848312 | 7.22E-90 | 2.64E-88 | 193.977893 |
| CRYAB | -3.66683922 | 3.719656552 | -21.91645671 | 7.45E-90 | 2.72E-88 | 193.9461703 |
| MEIS2 | -0.95331111 | 0.826731548 | -21.85568533 | 1.93E-89 | 7.04E-88 | 192.995399 |
| FAM89A | -1.822334612 | 1.73392961 | -21.83322927 | 2.75E-89 | 9.97E-88 | 192.6443604 |
| IQGAP3 | 2.024806937 | 2.358454676 | 21.83285773 | 2.77E-89 | 1.00E-87 | 192.6385537 |
| UHRF1 | 1.735852381 | 2.072233669 | 21.82500501 | 3.13E-89 | 1.13E-87 | 192.5158357 |
| TF | -2.416788819 | 1.137226329 | -21.7855363 | 5.80E-89 | 2.09E-87 | 191.8993294 |
| ECSCR | -1.491781491 | 2.030505249 | -21.76389924 | 8.14E-89 | 2.93E-87 | 191.5615612 |
| PAQR4 | 1.906508857 | 3.287565824 | 21.75785543 | 8.95E-89 | 3.21E-87 | 191.4672395 |
| ADGRD2 | -0.893334514 | 0.174655454 | -21.74700528 | 1.06E-88 | 3.80E-87 | 191.297937 |
| ABCA5 | -1.127002771 | 1.29210396 | -21.74355361 | 1.12E-88 | 4.01E-87 | 191.2440859 |
| CDH23 | -0.721176537 | 0.485578044 | -21.74092809 | 1.17E-88 | 4.17E-87 | 191.2031264 |
| S1PR1 | -1.937502523 | 3.043275342 | -21.73611301 | 1.26E-88 | 4.48E-87 | 191.1280141 |
| CNRIP1 | -1.27561118 | 1.785090269 | -21.71883956 | 1.65E-88 | 5.86E-87 | 190.858619 |
| NECTIN3 | -0.922656089 | 0.749698445 | -21.68551419 | 2.77E-88 | 9.84E-87 | 190.3391436 |
| RECK | -1.425950896 | 1.855638667 | -21.67922988 | 3.06E-88 | 1.08E-86 | 190.2412229 |
| KLF9 | -1.614695186 | 3.392875809 | -21.66222542 | 3.99E-88 | 1.41E-86 | 189.9763256 |
| PTPRB | -1.382143221 | 1.603934421 | -21.64948815 | 4.87E-88 | 1.72E-86 | 189.7779626 |
| CENPF | 2.174548411 | 2.962401121 | 21.64580517 | 5.16E-88 | 1.82E-86 | 189.7206156 |
| HOXA3 | -1.096187751 | 0.750387772 | -21.61930534 | 7.80E-88 | 2.74E-86 | 189.3081173 |
| ARF1 | 0.995822145 | 7.632098704 | 21.61719424 | 8.06E-88 | 2.83E-86 | 189.2752653 |
| DCUN1D3 | -0.849708543 | 1.861969495 | -21.58735955 | 1.28E-87 | 4.49E-86 | 188.8111423 |
| PKMYT1 | 1.789177821 | 1.941606097 | 21.58305351 | 1.37E-87 | 4.79E-86 | 188.7441787 |
| HS3ST4 | -0.95185774 | 0.235797788 | -21.57890612 | 1.46E-87 | 5.10E-86 | 188.6796879 |
| PDGFD | -1.880644591 | 2.626553095 | -21.56756571 | 1.75E-87 | 6.08E-86 | 188.5033755 |
| SSPN | -1.585777192 | 2.418871454 | -21.56328653 | 1.87E-87 | 6.48E-86 | 188.4368565 |
| GNG2 | -1.449974089 | 1.912864837 | -21.52385204 | 3.45E-87 | 1.19E-85 | 187.8241307 |
| TRMT9B | -0.877278853 | 0.507761825 | -21.52052765 | 3.64E-87 | 1.26E-85 | 187.7724996 |
| CDKN3 | 1.740845467 | 2.379871403 | 21.51911271 | 3.72E-87 | 1.28E-85 | 187.7505254 |
| JPH2 | -1.495609065 | 0.899032659 | -21.49979703 | 5.02E-87 | 1.73E-85 | 187.4506124 |
| FAM83H | 1.704212957 | 4.535962372 | 21.4791458 | 6.92E-87 | 2.38E-85 | 187.130095 |
| NUDT7 | -0.958471851 | 1.15681551 | -21.47763149 | 7.09E-87 | 2.43E-85 | 187.1065975 |
| PTPN14 | -1.49561197 | 2.039859751 | -21.47629141 | 7.24E-87 | 2.48E-85 | 187.0858041 |
| THSD7B | -0.649378724 | 0.37233961 | -21.4121436 | 1.96E-86 | 6.70E-85 | 186.0911325 |
| DGAT2 | -2.4646214 | 2.205430732 | -21.38946622 | 2.79E-86 | 9.52E-85 | 185.7398167 |
| BIRC5 | 2.463960867 | 3.126292985 | 21.37884717 | 3.29E-86 | 1.12E-84 | 185.5753648 |
| AKR1C3 | -2.118735416 | 2.049328848 | -21.37670576 | 3.40E-86 | 1.15E-84 | 185.5422063 |
| CCT3 | 1.133575287 | 6.664840376 | 21.34676462 | 5.42E-86 | 1.83E-84 | 185.0787412 |
| TFPI | -1.516847253 | 1.40210381 | -21.33608145 | 6.39E-86 | 2.16E-84 | 184.9134447 |
| FFAR4 | -0.671972083 | 0.493448716 | -21.31885894 | 8.35E-86 | 2.80E-84 | 184.6470464 |
| TACC3 | 1.632124915 | 2.76449989 | 21.3128876 | 9.16E-86 | 3.07E-84 | 184.5547041 |
| FAM110D | -1.4539215 | 1.233922664 | -21.308801 | 9.76E-86 | 3.27E-84 | 184.4915147 |
| FLRT2 | -0.755897394 | 0.536136694 | -21.29850499 | 1.14E-85 | 3.82E-84 | 184.3323359 |
| C20orf194 | -0.974195767 | 1.93097913 | -21.2923484 | 1.26E-85 | 4.20E-84 | 184.2371701 |
| CCDC152 | -0.71358454 | 0.676881117 | -21.28390765 | 1.44E-85 | 4.78E-84 | 184.1067171 |
| AOX1 | -1.913767743 | 1.399516434 | -21.28160317 | 1.49E-85 | 4.94E-84 | 184.071105 |
| CAVIN1 | -1.889525002 | 6.061506781 | -21.24538477 | 2.61E-85 | 8.65E-84 | 183.5116374 |
| ADAMTS1 | -2.275636428 | 2.884324418 | -21.23654431 | 2.99E-85 | 9.90E-84 | 183.3751439 |
| NUP210 | 1.663817355 | 3.850625321 | 21.21902147 | 3.92E-85 | 1.29E-83 | 183.1046738 |
| RCBTB2 | -1.053315954 | 2.272454377 | -21.21894478 | 3.92E-85 | 1.29E-83 | 183.1034904 |
| ELMOD3 | -0.960459837 | 2.113586659 | -21.21346312 | 4.27E-85 | 1.41E-83 | 183.0189003 |
| ATP6V0B | 1.238700524 | 5.208326994 | 21.208461 | 4.62E-85 | 1.52E-83 | 182.9417189 |
| FAXDC2 | -2.060068704 | 1.969360903 | -21.20210846 | 5.09E-85 | 1.67E-83 | 182.843713 |
| FAT4 | -1.009645547 | 0.799921274 | -21.19573972 | 5.62E-85 | 1.84E-83 | 182.7454705 |
| CEP55 | 2.053828257 | 2.584772702 | 21.18517547 | 6.62E-85 | 2.16E-83 | 182.5825387 |
| HOXA7 | -1.0527314 | 0.526404723 | -21.17698917 | 7.51E-85 | 2.45E-83 | 182.4563074 |
| TMEM178A | -1.242801385 | 0.833628897 | -21.16326348 | 9.28E-85 | 3.02E-83 | 182.2447098 |
| PKD2 | -1.352557075 | 2.599117408 | -21.15935726 | 9.86E-85 | 3.21E-83 | 182.1845021 |
| PROX1 | -0.902278202 | 0.416082245 | -21.14393237 | 1.25E-84 | 4.06E-83 | 181.946804 |
| SOBP | -0.890746439 | 0.747508568 | -21.11493881 | 1.96E-84 | 6.35E-83 | 181.5002271 |
| SAMD4A | -1.083220718 | 1.235692862 | -21.11406473 | 1.99E-84 | 6.42E-83 | 181.4867683 |
| CDC20 | 2.561391734 | 3.660329916 | 21.11236907 | 2.04E-84 | 6.58E-83 | 181.4606598 |
| CHST9 | -0.911217476 | 0.352742046 | -21.10595204 | 2.25E-84 | 7.26E-83 | 181.3618643 |
| NOTCH4 | -1.169306247 | 1.843395472 | -21.09986195 | 2.47E-84 | 7.96E-83 | 181.2681148 |
| KCNJ16 | -0.958148135 | 0.222618222 | -21.08994046 | 2.88E-84 | 9.26E-83 | 181.1154124 |
| INHBA | 2.221451872 | 2.987911389 | 21.06081791 | 4.51E-84 | 1.45E-82 | 180.6673756 |
| EGFR | -2.337451095 | 1.969382646 | -21.05926399 | 4.62E-84 | 1.48E-82 | 180.6434773 |
| PPP4C | 1.213122468 | 5.384763368 | 21.05682884 | 4.80E-84 | 1.54E-82 | 180.6060279 |
| PDE7B | -0.78661772 | 0.706511754 | -21.05173251 | 5.19E-84 | 1.66E-82 | 180.5276593 |
| HSD17B6 | 1.284087331 | 1.550047968 | 21.01817497 | 8.71E-84 | 2.77E-82 | 180.0118488 |
| PBK | 2.029192026 | 2.502849464 | 21.00160582 | 1.12E-83 | 3.57E-82 | 179.7573059 |
| PLA2R1 | -1.191736124 | 1.605631131 | -20.95658343 | 2.25E-83 | 7.13E-82 | 179.0661207 |
| KIF11 | 1.75415348 | 2.86509301 | 20.95526859 | 2.29E-83 | 7.27E-82 | 179.0459456 |
| EFCAB1 | -0.649960644 | 0.237577554 | -20.94573549 | 2.66E-83 | 8.40E-82 | 178.8996857 |
| ESRP1 | 1.693438547 | 4.863891091 | 20.94319019 | 2.76E-83 | 8.72E-82 | 178.8606401 |
| CRB3 | 1.398853954 | 3.23233224 | 20.93763979 | 3.01E-83 | 9.47E-82 | 178.7755032 |
| ZNF366 | -0.843007132 | 0.701345261 | -20.93156468 | 3.30E-83 | 1.04E-81 | 178.6823297 |
| SYNE1 | -0.930979844 | 1.102481453 | -20.91863358 | 4.03E-83 | 1.26E-81 | 178.4840485 |
| GRK3 | -1.270472487 | 2.168966078 | -20.91516199 | 4.25E-83 | 1.33E-81 | 178.4308261 |
| JPT1 | 1.840233404 | 4.738475299 | 20.90812808 | 4.74E-83 | 1.48E-81 | 178.3230028 |
| NOVA2 | -0.618277908 | 0.636324073 | -20.90636206 | 4.87E-83 | 1.52E-81 | 178.2959339 |
| RAPGEF3 | -1.19757069 | 1.240701996 | -20.90170805 | 5.23E-83 | 1.63E-81 | 178.2246044 |
| NPR2 | -1.258524563 | 1.736732769 | -20.89414341 | 5.87E-83 | 1.83E-81 | 178.1086809 |
| RXRG | -0.6104491 | 0.217826439 | -20.89207063 | 6.06E-83 | 1.88E-81 | 178.0769203 |
| PLAGL1 | -1.378740164 | 1.321231529 | -20.89038245 | 6.22E-83 | 1.93E-81 | 178.0510539 |
| RUSC1 | 1.192531769 | 3.623876597 | 20.88384351 | 6.88E-83 | 2.13E-81 | 177.9508729 |
| TSTA3 | 1.565102883 | 4.77784337 | 20.88256492 | 7.02E-83 | 2.17E-81 | 177.9312858 |
| DPP3 | 1.28390008 | 3.954730587 | 20.87705394 | 7.64E-83 | 2.35E-81 | 177.8468676 |
| RHOQ | -1.203906521 | 3.580374998 | -20.85486027 | 1.07E-82 | 3.29E-81 | 177.5070064 |
| CX3CL1 | -2.663951411 | 3.27358363 | -20.84995953 | 1.16E-82 | 3.55E-81 | 177.4319821 |
| TP53AIP1 | -0.760566534 | 0.327674262 | -20.84531965 | 1.24E-82 | 3.80E-81 | 177.3609588 |
| VWF | -1.888650724 | 4.115931024 | -20.81850587 | 1.88E-82 | 5.73E-81 | 176.950662 |
| IQSEC3 | -0.621631401 | 0.314517397 | -20.81761214 | 1.90E-82 | 5.80E-81 | 176.9369908 |
| CTHRC1 | 2.32994927 | 5.27434658 | 20.81538284 | 1.97E-82 | 5.99E-81 | 176.9028904 |
| BMP2 | -1.201448652 | 0.698689387 | -20.80447235 | 2.33E-82 | 7.07E-81 | 176.7360239 |
| TWIST2 | -1.657805627 | 1.615326955 | -20.80047043 | 2.47E-82 | 7.50E-81 | 176.6748284 |
| C10orf90 | -0.769981788 | 0.235195886 | -20.80046166 | 2.47E-82 | 7.50E-81 | 176.6746943 |
| CBX7 | -1.42037423 | 2.263146959 | -20.79251946 | 2.79E-82 | 8.45E-81 | 176.5532621 |
| TNS2 | -1.516753696 | 3.440590601 | -20.78871078 | 2.96E-82 | 8.95E-81 | 176.4950371 |
| UGP2 | -1.06518666 | 4.322674143 | -20.78521356 | 3.13E-82 | 9.43E-81 | 176.4415779 |
| COX7A1 | -2.080074646 | 2.776206526 | -20.76893066 | 4.01E-82 | 1.21E-80 | 176.1927301 |
| CELF2 | -1.592191748 | 2.031824847 | -20.76430799 | 4.31E-82 | 1.29E-80 | 176.1220997 |
| EHBP1 | -1.016133167 | 2.594768434 | -20.76222355 | 4.45E-82 | 1.33E-80 | 176.0902537 |
| CSRNP1 | -1.65916774 | 3.734175476 | -20.74853083 | 5.48E-82 | 1.64E-80 | 175.8810941 |
| GPRASP2 | -1.152448502 | 1.942955451 | -20.73535748 | 6.71E-82 | 2.01E-80 | 175.6799295 |
| LARP6 | -1.233001527 | 1.475084655 | -20.73345157 | 6.91E-82 | 2.06E-80 | 175.6508303 |
| AURKA | 2.001300469 | 2.833181332 | 20.72961053 | 7.33E-82 | 2.19E-80 | 175.5921892 |
| BICDL1 | 1.585201194 | 2.173395322 | 20.72050276 | 8.42E-82 | 2.51E-80 | 175.4531619 |
| LURAP1 | -0.648431723 | 0.571107261 | -20.71983312 | 8.51E-82 | 2.53E-80 | 175.4429413 |
| CDCA8 | 1.986516475 | 2.91876484 | 20.7168498 | 8.91E-82 | 2.65E-80 | 175.3974088 |
| MSRB3 | -1.806561136 | 2.915266131 | -20.67177568 | 1.77E-81 | 5.26E-80 | 174.7098501 |
| PNMA8C | -0.707085986 | 0.211830187 | -20.66218352 | 2.06E-81 | 6.09E-80 | 174.5636234 |
| MT1M | -1.878619056 | 1.401612406 | -20.63432306 | 3.15E-81 | 9.30E-80 | 174.1390907 |
| SMIM10 | -1.194133811 | 1.665805918 | -20.61941281 | 3.95E-81 | 1.17E-79 | 173.9120031 |
| TYRO3 | -1.047837348 | 1.090340478 | -20.61755062 | 4.06E-81 | 1.20E-79 | 173.8836469 |
| TNNT3 | -1.473245752 | 0.59710573 | -20.61664113 | 4.12E-81 | 1.21E-79 | 173.8697983 |
| DOCK11 | -1.582794482 | 1.962403832 | -20.60790383 | 4.71E-81 | 1.38E-79 | 173.7367715 |
| TEK | -1.45629156 | 1.876600612 | -20.59000709 | 6.19E-81 | 1.82E-79 | 173.4643748 |
| HMMR | 1.597670597 | 1.992420101 | 20.58897307 | 6.29E-81 | 1.84E-79 | 173.4486399 |
| CDH13 | -1.021760562 | 1.464308718 | -20.58509734 | 6.67E-81 | 1.95E-79 | 173.3896659 |
| TMEM63B | 1.121818066 | 4.118585981 | 20.57234315 | 8.10E-81 | 2.37E-79 | 173.1956324 |
| TNFRSF10D | -1.191193762 | 0.975809587 | -20.56387549 | 9.22E-81 | 2.69E-79 | 173.0668431 |
| KIF22 | 1.261342267 | 4.025044814 | 20.54192648 | 1.29E-80 | 3.75E-79 | 172.7331268 |
| SAA2 | -2.762335729 | 1.640990617 | -20.53124775 | 1.52E-80 | 4.41E-79 | 172.5708276 |
| FAT2 | -1.533965797 | 0.795474997 | -20.52495383 | 1.67E-80 | 4.85E-79 | 172.4751892 |
| TRDN | -0.81087877 | 0.15207668 | -20.52476177 | 1.67E-80 | 4.85E-79 | 172.4722709 |
| PRKD1 | -1.007917354 | 1.163296163 | -20.52171437 | 1.75E-80 | 5.08E-79 | 172.4259699 |
| CADM3 | -1.515180982 | 0.81616954 | -20.52122889 | 1.77E-80 | 5.11E-79 | 172.418594 |
| RGL1 | -1.38972967 | 2.500097406 | -20.49800171 | 2.52E-80 | 7.25E-79 | 172.0658017 |
| PRC1 | 1.855255886 | 3.083279036 | 20.48023811 | 3.30E-80 | 9.49E-79 | 171.7961242 |
| BMP6 | -1.448606004 | 1.173695519 | -20.47883992 | 3.37E-80 | 9.68E-79 | 171.7749025 |
| CRTAP | -0.958615653 | 4.754080044 | -20.46035183 | 4.46E-80 | 1.28E-78 | 171.4943553 |
| RGN | -1.277195776 | 0.721773006 | -20.4418087 | 5.92E-80 | 1.70E-78 | 171.2130957 |
| ACSL1 | -2.223524306 | 4.085631361 | -20.43920932 | 6.15E-80 | 1.76E-78 | 171.1736787 |
| H2AFZ | 1.324341221 | 6.183144703 | 20.43561005 | 6.50E-80 | 1.86E-78 | 171.1191031 |
| PLK1 | 1.958394364 | 2.451554063 | 20.43251662 | 6.81E-80 | 1.94E-78 | 171.0722013 |
| DAAM2 | -1.376492961 | 1.70998541 | -20.43172896 | 6.90E-80 | 1.96E-78 | 171.0602596 |
| COMP | 3.428125231 | 4.334903636 | 20.43010241 | 7.07E-80 | 2.01E-78 | 171.0356 |
| CD302 | -1.27994022 | 1.654352102 | -20.42536234 | 7.60E-80 | 2.16E-78 | 170.9637432 |
| NDN | -1.756208735 | 2.949409591 | -20.42170178 | 8.03E-80 | 2.28E-78 | 170.9082564 |
| CENPM | 1.643393423 | 2.252302598 | 20.41030808 | 9.55E-80 | 2.70E-78 | 170.735582 |
| PLEKHH2 | -1.143780083 | 0.914291737 | -20.40619665 | 1.02E-79 | 2.88E-78 | 170.6732838 |
| MARCKSL1 | 1.576305645 | 7.563529846 | 20.39063112 | 1.29E-79 | 3.64E-78 | 170.4374828 |
| TP63 | -2.178401374 | 1.391965929 | -20.3785615 | 1.55E-79 | 4.36E-78 | 170.2547012 |
| CCNB2 | 1.980538066 | 2.838561509 | 20.33626857 | 2.94E-79 | 8.25E-78 | 169.6146354 |
| POLR3K | 1.266785404 | 3.738546736 | 20.29796882 | 5.25E-79 | 1.47E-77 | 169.0355625 |
| DNAJC18 | -0.707312902 | 1.236424108 | -20.28694417 | 6.21E-79 | 1.73E-77 | 168.8689743 |
| KANK3 | -1.127584322 | 1.029109968 | -20.27778962 | 7.13E-79 | 1.99E-77 | 168.7306779 |
| DIXDC1 | -1.30412634 | 2.302910189 | -20.26524764 | 8.62E-79 | 2.40E-77 | 168.5412578 |
| SAA1 | -4.351454681 | 4.674218507 | -20.25719581 | 9.74E-79 | 2.71E-77 | 168.4196823 |
| PTPN21 | -1.229149832 | 1.841986312 | -20.25505311 | 1.01E-78 | 2.79E-77 | 168.3873334 |
| AMOTL2 | -1.437522444 | 3.652011254 | -20.23848841 | 1.29E-78 | 3.58E-77 | 168.1373086 |
| HNRNPAB | 1.004674417 | 6.271018222 | 20.23098613 | 1.45E-78 | 4.00E-77 | 168.0241034 |
| FAM122A | -0.82691705 | 2.777933916 | -20.22579867 | 1.57E-78 | 4.33E-77 | 167.9458396 |
| CLIP4 | -1.498805077 | 1.604960727 | -20.22344329 | 1.62E-78 | 4.48E-77 | 167.9103071 |
| MAZ | 1.140407471 | 4.350907198 | 20.22306878 | 1.63E-78 | 4.50E-77 | 167.9046575 |
| CTXN1 | 2.279063622 | 3.817686397 | 20.21753811 | 1.78E-78 | 4.88E-77 | 167.821232 |
| SPC24 | 1.608378562 | 1.998485074 | 20.21716959 | 1.79E-78 | 4.90E-77 | 167.8156736 |
| TIMM17A | 0.999463755 | 4.432534556 | 20.20604188 | 2.11E-78 | 5.79E-77 | 167.6478573 |
| EHD2 | -1.757874494 | 4.671677818 | -20.19480341 | 2.50E-78 | 6.85E-77 | 167.4784167 |
| HJURP | 1.709654097 | 1.97917654 | 20.19301052 | 2.57E-78 | 7.03E-77 | 167.4513899 |
| MKI67 | 2.039319217 | 2.715337123 | 20.1824364 | 3.02E-78 | 8.24E-77 | 167.2920152 |
| ALX4 | -1.224811986 | 0.534341162 | -20.18200835 | 3.04E-78 | 8.28E-77 | 167.2855644 |
| RMI2 | 1.675462323 | 2.52428571 | 20.18110857 | 3.08E-78 | 8.38E-77 | 167.2720048 |
| NRG1 | -1.115929633 | 0.554105923 | -20.17918608 | 3.17E-78 | 8.62E-77 | 167.2430341 |
| LSM4 | 1.240244641 | 4.896974583 | 20.16687772 | 3.82E-78 | 1.04E-76 | 167.0575873 |
| SPRY1 | -1.890475468 | 3.336362229 | -20.1664665 | 3.84E-78 | 1.04E-76 | 167.0513924 |
| CRYBG3 | -1.391807721 | 1.278722784 | -20.16478435 | 3.94E-78 | 1.07E-76 | 167.0260526 |
| DLGAP5 | 1.784366969 | 2.179358253 | 20.15231626 | 4.76E-78 | 1.29E-76 | 166.8382656 |
| ARID5B | -1.524336914 | 3.334684958 | -20.1370262 | 5.99E-78 | 1.62E-76 | 166.6080544 |
| PTGFR | -1.214690681 | 0.631203794 | -20.13091095 | 6.57E-78 | 1.77E-76 | 166.5160057 |
| CCNF | 1.203301427 | 2.415004956 | 20.11339503 | 8.56E-78 | 2.31E-76 | 166.2524274 |
| CFL1 | 0.987042559 | 7.090281846 | 20.10218199 | 1.01E-77 | 2.73E-76 | 166.083754 |
| ANO6 | -1.274074321 | 3.793973325 | -20.08622573 | 1.29E-77 | 3.46E-76 | 165.8438108 |
| EDN3 | -1.729013524 | 0.612535282 | -20.06783884 | 1.70E-77 | 4.56E-76 | 165.5674345 |
| BUB1B | 1.639016389 | 2.14691213 | 20.06112564 | 1.88E-77 | 5.04E-76 | 165.4665586 |
| EPB41L2 | -1.524590778 | 2.853713116 | -20.05693691 | 2.01E-77 | 5.36E-76 | 165.4036252 |
| NUF2 | 1.896161051 | 2.193309917 | 20.05379205 | 2.10E-77 | 5.61E-76 | 165.3563797 |
| POU6F1 | -0.806929779 | 1.339628534 | -20.02839163 | 3.08E-77 | 8.19E-76 | 164.9749223 |
| EGR1 | -3.014319943 | 5.825594032 | -20.02549533 | 3.22E-77 | 8.54E-76 | 164.9314417 |
| OGN | -2.890500159 | 2.399906342 | -20.01233518 | 3.93E-77 | 1.04E-75 | 164.7339149 |
| KIF2C | 1.998103917 | 2.632961021 | 20.005232 | 4.37E-77 | 1.15E-75 | 164.627327 |
| CENPU | 1.677393274 | 2.918971914 | 19.97873114 | 6.51E-77 | 1.71E-75 | 164.2298311 |
| KCTD12 | -1.661657526 | 4.028552078 | -19.9679624 | 7.66E-77 | 2.01E-75 | 164.0683824 |
| CFD | -3.314919135 | 3.949328838 | -19.96690373 | 7.78E-77 | 2.04E-75 | 164.0525128 |
| SCN2A | -0.677578918 | 0.260009797 | -19.96298127 | 8.25E-77 | 2.16E-75 | 163.9937182 |
| SPINT2 | 1.683421263 | 6.493971082 | 19.95528677 | 9.26E-77 | 2.43E-75 | 163.8784006 |
| CDCA5 | 1.899822002 | 2.614107391 | 19.94006722 | 1.16E-76 | 3.05E-75 | 163.650371 |
| MEDAG | -1.919354003 | 2.019036521 | -19.93756062 | 1.21E-76 | 3.16E-75 | 163.6128237 |
| JADE1 | -0.954170627 | 2.352447512 | -19.91031324 | 1.82E-76 | 4.75E-75 | 163.2048292 |
| SOX12 | 1.448713612 | 3.445666411 | 19.90977509 | 1.83E-76 | 4.78E-75 | 163.196774 |
| SOD3 | -2.460771341 | 2.850006944 | -19.9018677 | 2.07E-76 | 5.38E-75 | 163.0784252 |
| EZH2 | 1.49046098 | 2.495923322 | 19.90137607 | 2.08E-76 | 5.41E-75 | 163.0710677 |
| LRRN3 | -1.172896043 | 0.630939781 | -19.8989351 | 2.16E-76 | 5.60E-75 | 163.0345393 |
| STAT5B | -1.184053451 | 3.723073961 | -19.88994008 | 2.47E-76 | 6.40E-75 | 162.8999509 |
| AP1M2 | 1.779146623 | 5.402447648 | 19.88836548 | 2.53E-76 | 6.55E-75 | 162.8763941 |
| VSIR | -1.526177621 | 2.737856585 | -19.87254052 | 3.21E-76 | 8.28E-75 | 162.6396964 |
| INKA2 | -0.6994087 | 0.780730947 | -19.85958616 | 3.90E-76 | 1.00E-74 | 162.4460058 |
| FN1 | 2.704254969 | 7.387862831 | 19.85794896 | 3.99E-76 | 1.03E-74 | 162.4215312 |
| FGFBP2 | -1.811051375 | 0.718552652 | -19.8568958 | 4.06E-76 | 1.04E-74 | 162.405788 |
| CNTFR | -2.416734606 | 1.473143695 | -19.85228952 | 4.35E-76 | 1.12E-74 | 162.3369362 |
| ARHGAP10 | -0.912679337 | 1.226699257 | -19.84345602 | 4.96E-76 | 1.27E-74 | 162.2049207 |
| CDC25C | 1.237079899 | 1.380238125 | 19.83647934 | 5.51E-76 | 1.41E-74 | 162.1006764 |
| LHX6 | -1.079429588 | 1.030514904 | -19.82374344 | 6.67E-76 | 1.71E-74 | 161.9104265 |
| DLC1 | -1.479315186 | 2.291331247 | -19.7968638 | 9.97E-76 | 2.54E-74 | 161.5091 |
| TK2 | -0.870131647 | 2.624049122 | -19.78552272 | 1.18E-75 | 3.01E-74 | 161.3398549 |
| HDGF | 1.118244746 | 6.6996038 | 19.7844551 | 1.20E-75 | 3.06E-74 | 161.3239251 |
| STBD1 | -0.838434522 | 0.970528928 | -19.77700466 | 1.34E-75 | 3.41E-74 | 161.2127706 |
| BUB1 | 1.705271635 | 2.09152536 | 19.75399799 | 1.89E-75 | 4.81E-74 | 160.8696645 |
| NAXE | 1.201373624 | 5.864932782 | 19.74965387 | 2.02E-75 | 5.12E-74 | 160.8049019 |
| PTBP1 | 0.642386235 | 5.3585936 | 19.74413129 | 2.19E-75 | 5.56E-74 | 160.7225813 |
| WDR34 | 1.435860094 | 4.944957516 | 19.73984103 | 2.34E-75 | 5.92E-74 | 160.6586381 |
| EMP1 | -1.798698263 | 4.48019943 | -19.70733308 | 3.80E-75 | 9.60E-74 | 160.1743606 |
| CDK5 | 1.063947713 | 2.827908448 | 19.70111376 | 4.17E-75 | 1.05E-73 | 160.0817567 |
| N4BP2L1 | -1.092800028 | 1.79737212 | -19.68648549 | 5.19E-75 | 1.31E-73 | 159.8640047 |
| PARP1 | 1.083759531 | 5.247468523 | 19.68397964 | 5.39E-75 | 1.36E-73 | 159.8267115 |
| FBLN5 | -1.911570162 | 3.351745198 | -19.65387473 | 8.45E-75 | 2.12E-73 | 159.378869 |
| BNIP2 | -0.824246323 | 3.067485316 | -19.65181349 | 8.71E-75 | 2.18E-73 | 159.3482188 |
| TOR3A | 0.912666767 | 4.214197939 | 19.63904414 | 1.05E-74 | 2.64E-73 | 159.1583776 |
| SYNPO | -1.463111581 | 3.135508792 | -19.63892169 | 1.06E-74 | 2.64E-73 | 159.1565574 |
| MPRIP | -0.872734121 | 3.058983273 | -19.63208407 | 1.17E-74 | 2.92E-73 | 159.0549293 |
| MELK | 2.016670693 | 2.444596924 | 19.61631017 | 1.48E-74 | 3.68E-73 | 158.82055 |
| CACNA2D1 | -1.196950975 | 1.239001743 | -19.60810196 | 1.67E-74 | 4.15E-73 | 158.6986252 |
| RGCC | -1.516313955 | 4.269909333 | -19.60489075 | 1.75E-74 | 4.35E-73 | 158.650933 |
| ANLN | 2.01074378 | 2.596704849 | 19.59679053 | 1.98E-74 | 4.90E-73 | 158.5306482 |
| ABLIM1 | -1.491811278 | 3.715341298 | -19.5963999 | 1.99E-74 | 4.93E-73 | 158.5248481 |
| JTB | 1.021174784 | 6.741542568 | 19.58995943 | 2.19E-74 | 5.42E-73 | 158.4292292 |
| TRIM11 | 0.990405268 | 2.497073243 | 19.58409072 | 2.39E-74 | 5.90E-73 | 158.3421129 |
| PLCXD3 | -0.625857852 | 0.256972095 | -19.58148522 | 2.48E-74 | 6.12E-73 | 158.3034407 |
| HADH | -1.073502194 | 4.678248418 | -19.5719011 | 2.86E-74 | 7.04E-73 | 158.1612111 |
| SLC20A1 | 0.988142211 | 3.844290303 | 19.55288412 | 3.80E-74 | 9.33E-73 | 157.879103 |
| GABRE | -1.612575135 | 0.835597 | -19.54102698 | 4.53E-74 | 1.11E-72 | 157.7032795 |
| HM13 | 0.901155729 | 4.278150322 | 19.53843365 | 4.71E-74 | 1.15E-72 | 157.6648317 |
| SPAG5 | 1.723319944 | 2.548077152 | 19.53842928 | 4.71E-74 | 1.15E-72 | 157.6647669 |
| KPNA2 | 1.797141159 | 5.254220336 | 19.53103432 | 5.26E-74 | 1.28E-72 | 157.5551464 |
| AP1S1 | 1.128458455 | 5.052395741 | 19.52957275 | 5.38E-74 | 1.31E-72 | 157.533483 |
| COL17A1 | -3.092705705 | 2.172443005 | -19.52916989 | 5.41E-74 | 1.32E-72 | 157.527512 |
| PCSK5 | -0.877778963 | 0.880931079 | -19.52842678 | 5.47E-74 | 1.33E-72 | 157.5164981 |
| TEF | -1.235018303 | 2.789927986 | -19.52698133 | 5.59E-74 | 1.36E-72 | 157.4950753 |
| C1QTNF7 | -1.005460424 | 0.539863929 | -19.52599391 | 5.67E-74 | 1.37E-72 | 157.4804412 |
| TEDC2 | 1.290458217 | 1.651237586 | 19.52178171 | 6.04E-74 | 1.46E-72 | 157.418019 |
| CXCL12 | -2.064838034 | 4.234927484 | -19.51443255 | 6.73E-74 | 1.63E-72 | 157.3091253 |
| SMAD9 | -0.844380464 | 0.899012744 | -19.50478964 | 7.77E-74 | 1.88E-72 | 157.1662772 |
| MGLL | -1.910118845 | 3.09874386 | -19.50192642 | 8.11E-74 | 1.96E-72 | 157.1238691 |
| DPYSL2 | -1.436122939 | 3.728447978 | -19.49760587 | 8.65E-74 | 2.08E-72 | 157.059882 |
| CDH5 | -1.454255513 | 3.085665533 | -19.48693714 | 1.01E-73 | 2.44E-72 | 156.9019104 |
| CRIM1 | -1.825938094 | 4.11091425 | -19.48463174 | 1.05E-73 | 2.52E-72 | 156.8677803 |
| RNF180 | -0.9363771 | 1.039930099 | -19.47222961 | 1.26E-73 | 3.03E-72 | 156.6842101 |
| FAM189A2 | -2.027101496 | 1.847829045 | -19.45829975 | 1.55E-73 | 3.72E-72 | 156.4780997 |
| PDLIM3 | -1.724647992 | 2.186492579 | -19.4568899 | 1.58E-73 | 3.79E-72 | 156.4572435 |
| FOXM1 | 2.133580938 | 2.795108283 | 19.45443924 | 1.64E-73 | 3.93E-72 | 156.420992 |
| DLK2 | -0.976738922 | 0.893117821 | -19.44607075 | 1.86E-73 | 4.44E-72 | 156.2972191 |
| NUAK2 | 1.257135299 | 1.927208884 | 19.44574247 | 1.87E-73 | 4.46E-72 | 156.2923642 |
| CCNA2 | 1.89949195 | 2.833419983 | 19.44069178 | 2.01E-73 | 4.80E-72 | 156.2176769 |
| JPT2 | 1.197245823 | 5.584549529 | 19.44013309 | 2.03E-73 | 4.83E-72 | 156.2094158 |
| PCDH18 | -1.572505903 | 2.106081908 | -19.43952554 | 2.05E-73 | 4.87E-72 | 156.2004324 |
| RBP7 | -2.303918945 | 3.544995692 | -19.43264921 | 2.27E-73 | 5.38E-72 | 156.0987676 |
| LLGL2 | 1.478795283 | 3.533739519 | 19.43058889 | 2.34E-73 | 5.54E-72 | 156.0683099 |
| RAB25 | 1.764646685 | 5.892720907 | 19.41068721 | 3.14E-73 | 7.44E-72 | 155.7741907 |
| SPHKAP | -0.627183232 | 0.147162249 | -19.3853721 | 4.57E-73 | 1.08E-71 | 155.4002961 |
| ZNF106 | -1.185690145 | 3.013066448 | -19.37704605 | 5.17E-73 | 1.22E-71 | 155.2773794 |
| MTMR10 | -0.868644242 | 2.128294531 | -19.37265483 | 5.52E-73 | 1.30E-71 | 155.2125633 |
| H2AFX | 1.605761209 | 4.381617101 | 19.37044255 | 5.70E-73 | 1.34E-71 | 155.1799121 |
| KIF23 | 1.49715171 | 2.154394521 | 19.36276286 | 6.39E-73 | 1.50E-71 | 155.0665822 |
| SLC10A6 | -0.825901749 | 0.545652233 | -19.3602866 | 6.62E-73 | 1.56E-71 | 155.0300447 |
| GFAP | -0.735223006 | 0.262658569 | -19.33806836 | 9.20E-73 | 2.16E-71 | 154.702323 |
| WISP1 | 1.837065958 | 2.197845288 | 19.33800734 | 9.21E-73 | 2.16E-71 | 154.7014232 |
| KAT2B | -1.127860029 | 2.01633751 | -19.32245935 | 1.16E-72 | 2.71E-71 | 154.4722063 |
| FEZ1 | -0.810743076 | 0.921051524 | -19.31991142 | 1.20E-72 | 2.81E-71 | 154.4346526 |
| SFRP1 | -4.234517162 | 3.752343161 | -19.26560174 | 2.68E-72 | 6.27E-71 | 153.634807 |
| PRIMA1 | -1.243505917 | 0.966680141 | -19.25346975 | 3.21E-72 | 7.49E-71 | 153.4562955 |
| ARL4A | -1.465399592 | 2.592379901 | -19.25076031 | 3.34E-72 | 7.79E-71 | 153.4164365 |
| TACC1 | -1.735624539 | 2.901350023 | -19.22658794 | 4.77E-72 | 1.11E-70 | 153.060965 |
| MMP28 | -1.27817044 | 1.287702118 | -19.2234454 | 5.00E-72 | 1.16E-70 | 153.0147692 |
| CLCN6 | -0.761378414 | 1.793576795 | -19.21240479 | 5.88E-72 | 1.37E-70 | 152.852502 |
| TROAP | 1.632528526 | 1.872952231 | 19.1812653 | 9.31E-72 | 2.16E-70 | 152.3951021 |
| ADGRL4 | -1.494283624 | 3.189666836 | -19.17864499 | 9.68E-72 | 2.24E-70 | 152.3566309 |
| MBOAT7 | 1.242847052 | 4.448946573 | 19.17003532 | 1.10E-71 | 2.54E-70 | 152.2302444 |
| AK5 | -2.079135276 | 1.344931442 | -19.16968122 | 1.10E-71 | 2.55E-70 | 152.2250469 |
| ARHGEF40 | -1.122060216 | 2.742778387 | -19.16808612 | 1.13E-71 | 2.61E-70 | 152.2016351 |
| ASB1 | -0.776169062 | 1.999795717 | -19.13679079 | 1.79E-71 | 4.12E-70 | 151.742511 |
| EIF2AK1 | 0.798103321 | 5.480436517 | 19.13332068 | 1.89E-71 | 4.34E-70 | 151.6916266 |
| RECQL4 | 1.727727753 | 2.590931799 | 19.13109133 | 1.95E-71 | 4.47E-70 | 151.658939 |
| HBA1 | -0.614617599 | 0.14545501 | -19.12873846 | 2.02E-71 | 4.63E-70 | 151.6244423 |
| SLC52A2 | 1.507852383 | 4.472863488 | 19.12249942 | 2.21E-71 | 5.07E-70 | 151.5329796 |
| ARAP3 | -0.982218332 | 1.791526937 | -19.09655451 | 3.24E-71 | 7.41E-70 | 151.1528044 |
| GINS1 | 1.472500723 | 2.249801564 | 19.09107688 | 3.51E-71 | 8.02E-70 | 151.072575 |
| LSR | 1.506349939 | 5.446761949 | 19.08938313 | 3.60E-71 | 8.21E-70 | 151.0477698 |
| DEPP1 | -2.193923681 | 4.496505869 | -19.0863399 | 3.76E-71 | 8.58E-70 | 151.0032037 |
| C2CD2 | -1.237332793 | 2.440472497 | -19.07335959 | 4.55E-71 | 1.04E-69 | 150.813159 |
| ALDH18A1 | 0.93331067 | 5.144818782 | 19.06653219 | 5.03E-71 | 1.14E-69 | 150.7132267 |
| ATP6AP1 | 1.102785982 | 5.809765677 | 19.06007084 | 5.53E-71 | 1.26E-69 | 150.61867 |
| SBDS | -0.764367415 | 5.605628939 | -19.05963902 | 5.57E-71 | 1.26E-69 | 150.6123512 |
| C1QTNF6 | 1.476910565 | 2.618867967 | 19.05901319 | 5.62E-71 | 1.27E-69 | 150.6031937 |
| KCTD5 | 0.903875981 | 3.692639698 | 19.05283198 | 6.15E-71 | 1.39E-69 | 150.5127554 |
| SEMA3A | -0.609342462 | 0.448301395 | -19.04932824 | 6.48E-71 | 1.47E-69 | 150.4614985 |
| SBK1 | 1.682351274 | 2.876516606 | 19.02383745 | 9.41E-71 | 2.13E-69 | 150.0887413 |
| ZC3H12C | -0.823864926 | 0.817992182 | -19.01180068 | 1.12E-70 | 2.53E-69 | 149.9128182 |
| KLF11 | -1.13127487 | 2.700945206 | -19.00644975 | 1.21E-70 | 2.74E-69 | 149.8346309 |
| TMEM8A | 1.136946272 | 3.965891102 | 19.0042737 | 1.25E-70 | 2.82E-69 | 149.8028382 |
| AIFM2 | -1.223047891 | 2.378037943 | -19.00050697 | 1.33E-70 | 2.98E-69 | 149.7478096 |
| TRIM59 | 0.863898241 | 1.333839556 | 18.99007631 | 1.54E-70 | 3.46E-69 | 149.5954576 |
| FGF7 | -1.312897406 | 1.248995708 | -18.97691414 | 1.87E-70 | 4.20E-69 | 149.403273 |
| PECAM1 | -1.532233109 | 4.460755238 | -18.97245964 | 2.00E-70 | 4.47E-69 | 149.3382479 |
| AFAP1L1 | -1.050320808 | 1.83617522 | -18.96923451 | 2.10E-70 | 4.69E-69 | 149.2911737 |
| STX11 | -1.306624649 | 1.525110463 | -18.96010714 | 2.39E-70 | 5.35E-69 | 149.1579736 |
| BOC | -1.56848673 | 1.653700674 | -18.94702383 | 2.90E-70 | 6.47E-69 | 148.9671028 |
| UBE2S | 1.667240353 | 2.830812228 | 18.93949926 | 3.24E-70 | 7.22E-69 | 148.8573601 |
| SLC9A9 | -1.06988749 | 1.432561623 | -18.93282312 | 3.57E-70 | 7.95E-69 | 148.760011 |
| EZR | 1.300132487 | 6.778048955 | 18.93030476 | 3.70E-70 | 8.23E-69 | 148.723294 |
| GTF2IRD1 | 0.861704915 | 2.721716132 | 18.92975585 | 3.73E-70 | 8.28E-69 | 148.7152913 |
| FEZ2 | -0.686542471 | 2.561053398 | -18.92219091 | 4.17E-70 | 9.24E-69 | 148.6050143 |
| TMED3 | 1.083911318 | 3.145534637 | 18.92123142 | 4.23E-70 | 9.36E-69 | 148.5910291 |
| CRY2 | -1.089696976 | 3.193566572 | -18.91678488 | 4.51E-70 | 9.98E-69 | 148.526223 |
| NCAPH | 1.598567383 | 2.221266562 | 18.90425984 | 5.42E-70 | 1.20E-68 | 148.3437211 |
| FGD4 | -0.880298525 | 1.367830367 | -18.89148354 | 6.53E-70 | 1.44E-68 | 148.1576256 |
| FBXO31 | -0.737540461 | 2.513545407 | -18.88437242 | 7.25E-70 | 1.60E-68 | 148.0540767 |
| TUBA1C | 1.22405506 | 4.280239247 | 18.88269575 | 7.43E-70 | 1.63E-68 | 148.0296649 |
| ADAMTSL4 | -1.286212748 | 1.342658608 | -18.87509865 | 8.30E-70 | 1.82E-68 | 147.9190682 |
| CCBE1 | -0.634140733 | 0.345417883 | -18.86149369 | 1.01E-69 | 2.22E-68 | 147.7210708 |
| FLAD1 | 1.042309938 | 3.943837644 | 18.85730992 | 1.08E-69 | 2.36E-68 | 147.6601986 |
| ABI3BP | -1.746671902 | 1.590826838 | -18.84908914 | 1.21E-69 | 2.66E-68 | 147.5406109 |
| FBXW7 | -0.710104237 | 1.701626295 | -18.83379384 | 1.52E-69 | 3.32E-68 | 147.3181857 |
| CBX3 | 0.892063475 | 5.678422132 | 18.80200379 | 2.41E-69 | 5.26E-68 | 146.8562071 |
| THRB | -1.360095174 | 1.994002513 | -18.79562717 | 2.65E-69 | 5.76E-68 | 146.7635922 |
| LRIG3 | -1.425866476 | 2.06097268 | -18.79540738 | 2.65E-69 | 5.78E-68 | 146.7604002 |
| RAD51 | 1.322287969 | 1.845942563 | 18.79254324 | 2.77E-69 | 6.02E-68 | 146.718807 |
| NEXN | -1.265171117 | 1.867702957 | -18.7895291 | 2.89E-69 | 6.28E-68 | 146.675039 |
| RNF187 | 0.926354903 | 5.786674642 | 18.77951098 | 3.35E-69 | 7.25E-68 | 146.5295948 |
| GJB2 | 2.688460314 | 3.309086466 | 18.76451667 | 4.16E-69 | 9.01E-68 | 146.3119848 |
| ARHGAP24 | -0.83359687 | 1.184081587 | -18.75667325 | 4.66E-69 | 1.01E-67 | 146.1981923 |
| COL11A1 | 3.317388235 | 3.44995316 | 18.74190624 | 5.78E-69 | 1.25E-67 | 145.984023 |
| IL17D | -0.593669608 | 0.544571773 | -18.7397657 | 5.97E-69 | 1.29E-67 | 145.9529859 |
| NDC80 | 1.643146933 | 2.067600438 | 18.72229394 | 7.69E-69 | 1.66E-67 | 145.699724 |
| LOXL4 | -1.793056599 | 1.374639859 | -18.72069842 | 7.87E-69 | 1.69E-67 | 145.6766027 |
| UTRN | -1.442532618 | 3.089891147 | -18.71892901 | 8.08E-69 | 1.73E-67 | 145.6509626 |
| PCNA | 1.253228548 | 6.178681574 | 18.71101051 | 9.06E-69 | 1.94E-67 | 145.536234 |
| PYGO2 | 0.846830383 | 4.779615909 | 18.70945159 | 9.27E-69 | 1.98E-67 | 145.5136505 |
| MYBL2 | 2.659075151 | 3.567831722 | 18.69969789 | 1.07E-68 | 2.28E-67 | 145.3723752 |
| PLEKHM3 | -0.717738918 | 0.68310877 | -18.68354972 | 1.35E-68 | 2.88E-67 | 145.1385698 |
| PRCC | 0.850409511 | 4.592878639 | 18.67154798 | 1.61E-68 | 3.43E-67 | 144.9648713 |
| EGR2 | -2.032758828 | 2.546991487 | -18.67082121 | 1.62E-68 | 3.46E-67 | 144.954355 |
| PPP2R1B | -1.043787853 | 2.680414841 | -18.66516928 | 1.76E-68 | 3.75E-67 | 144.872579 |
| ATP1B2 | -0.873889465 | 0.808206786 | -18.66309659 | 1.82E-68 | 3.87E-67 | 144.8425933 |
| LAGE3 | 1.601360635 | 4.642063762 | 18.65195049 | 2.14E-68 | 4.54E-67 | 144.6813735 |
| LCA5 | -0.879297672 | 1.268572214 | -18.64109883 | 2.50E-68 | 5.30E-67 | 144.5244636 |
| INPP1 | -0.982857539 | 1.980894125 | -18.62591805 | 3.12E-68 | 6.60E-67 | 144.3050412 |
| NCAPG | 1.554406165 | 1.957094409 | 18.6135691 | 3.73E-68 | 7.88E-67 | 144.1266227 |
| TRPC1 | -0.778736227 | 1.017064701 | -18.61325295 | 3.74E-68 | 7.91E-67 | 144.1220558 |
| ICAM2 | -1.090088671 | 1.655538434 | -18.60378947 | 4.30E-68 | 9.07E-67 | 143.9853722 |
| SAV1 | -0.950555009 | 3.251179188 | -18.57978447 | 6.08E-68 | 1.28E-66 | 143.638834 |
| SPARCL1 | -2.007163845 | 6.120097922 | -18.56439179 | 7.60E-68 | 1.60E-66 | 143.4167544 |
| RCC2 | 0.99239428 | 5.688803573 | 18.56265836 | 7.79E-68 | 1.63E-66 | 143.3917515 |
| RACGAP1 | 1.432496 | 3.28103887 | 18.55839879 | 8.29E-68 | 1.73E-66 | 143.3303173 |
| GRK5 | -0.896462445 | 1.60561854 | -18.55067456 | 9.27E-68 | 1.94E-66 | 143.2189335 |
| PLA2G4A | -1.582140181 | 1.386771368 | -18.54563347 | 9.97E-68 | 2.08E-66 | 143.1462545 |
| E2F1 | 1.798596713 | 2.924224172 | 18.54386341 | 1.02E-67 | 2.13E-66 | 143.1207376 |
| PSMC4 | 0.889862249 | 5.239269872 | 18.53103001 | 1.23E-67 | 2.57E-66 | 142.9357738 |
| SNRNP25 | 1.149698062 | 3.617394491 | 18.53061633 | 1.24E-67 | 2.58E-66 | 142.9298128 |
| CUTC | -0.759779466 | 2.974780717 | -18.52503375 | 1.34E-67 | 2.79E-66 | 142.8493759 |
| DCTPP1 | 1.134303718 | 4.975342219 | 18.52273329 | 1.39E-67 | 2.88E-66 | 142.8162336 |
| LAMTOR2 | 1.243727827 | 5.234484898 | 18.51298922 | 1.60E-67 | 3.32E-66 | 142.6758777 |
| PLPP4 | 1.948499134 | 1.947727099 | 18.50841094 | 1.71E-67 | 3.54E-66 | 142.6099454 |
| TLCD1 | 1.674095732 | 3.042219778 | 18.5019306 | 1.87E-67 | 3.88E-66 | 142.5166367 |
| CKAP2L | 1.318245167 | 1.500550227 | 18.48849981 | 2.28E-67 | 4.71E-66 | 142.3233081 |
| CCDC85A | -0.632923619 | 0.338381444 | -18.47874563 | 2.62E-67 | 5.41E-66 | 142.1829513 |
| CALCOCO1 | -1.029427842 | 3.266952483 | -18.46953395 | 2.99E-67 | 6.18E-66 | 142.0504386 |
| RFTN2 | -0.63817051 | 0.803269625 | -18.4572405 | 3.57E-67 | 7.35E-66 | 141.8736511 |
| GIMAP6 | -1.433184438 | 2.632254492 | -18.45620196 | 3.63E-67 | 7.46E-66 | 141.8587193 |
| VGLL3 | -1.18543112 | 1.207621636 | -18.44399125 | 4.33E-67 | 8.88E-66 | 141.6831922 |
| SKA3 | 1.45522788 | 1.818750281 | 18.40670125 | 7.41E-67 | 1.52E-65 | 141.1475565 |
| ID4 | -2.63546999 | 2.85227422 | -18.40469036 | 7.62E-67 | 1.56E-65 | 141.1186892 |
| PDP2 | -0.732372061 | 1.516403199 | -18.3944946 | 8.83E-67 | 1.81E-65 | 140.9723511 |
| FAM83D | 2.023003488 | 2.560577818 | 18.38902984 | 9.55E-67 | 1.95E-65 | 140.8939352 |
| PTCH1 | -0.687298526 | 0.625718914 | -18.36292103 | 1.39E-66 | 2.84E-65 | 140.5194699 |
| SLC29A4 | -1.366160953 | 1.161504838 | -18.35253818 | 1.61E-66 | 3.29E-65 | 140.370637 |
| OIP5 | 1.180044963 | 1.646504331 | 18.35069296 | 1.66E-66 | 3.38E-65 | 140.3441916 |
| PCNX1 | -0.91891506 | 2.190386085 | -18.33854384 | 1.97E-66 | 4.02E-65 | 140.1701101 |
| PPM1F | -0.869475938 | 2.140265955 | -18.3298207 | 2.24E-66 | 4.55E-65 | 140.0451584 |
| HYAL1 | -1.011366966 | 0.861140689 | -18.32568289 | 2.38E-66 | 4.82E-65 | 139.9858995 |
| MOB3C | -0.709603766 | 2.103941762 | -18.32138367 | 2.53E-66 | 5.12E-65 | 139.9243369 |
| SGCG | -1.549113529 | 0.563558606 | -18.31237471 | 2.88E-66 | 5.82E-65 | 139.7953599 |
| FANCI | 1.319970194 | 2.601292909 | 18.30078018 | 3.40E-66 | 6.87E-65 | 139.6294189 |
| CCL28 | -2.578927766 | 2.327471323 | -18.293205 | 3.79E-66 | 7.65E-65 | 139.5210348 |
| TINAGL1 | -1.89847029 | 2.873689989 | -18.28773775 | 4.10E-66 | 8.26E-65 | 139.4428265 |
| TIMELESS | 1.165799632 | 3.131715115 | 18.28409508 | 4.32E-66 | 8.70E-65 | 139.3907257 |
| CAMK1 | -0.770475347 | 1.265704794 | -18.28299954 | 4.38E-66 | 8.82E-65 | 139.3750574 |
| FEN1 | 1.311545212 | 3.912113653 | 18.27226583 | 5.11E-66 | 1.03E-64 | 139.2215737 |
| HMGB3 | 1.846420835 | 3.948910153 | 18.26317303 | 5.83E-66 | 1.17E-64 | 139.0915934 |
| OSR1 | -1.729025653 | 1.064124803 | -18.26219359 | 5.91E-66 | 1.19E-64 | 139.0775948 |
| SCAMP3 | 0.931732408 | 5.37310156 | 18.26104177 | 6.01E-66 | 1.20E-64 | 139.0611327 |
| ITGB1BP1 | -0.848636689 | 2.901993074 | -18.25695955 | 6.37E-66 | 1.28E-64 | 139.0027937 |
| PTPRM | -1.277587848 | 2.461211223 | -18.25550176 | 6.50E-66 | 1.30E-64 | 138.9819622 |
| SQLE | 1.872607032 | 4.172437507 | 18.25479139 | 6.57E-66 | 1.31E-64 | 138.9718115 |
| LMO2 | -1.071230074 | 2.422066086 | -18.2454107 | 7.52E-66 | 1.50E-64 | 138.8377892 |
| MAP3K20 | -1.218147953 | 2.69640157 | -18.24410423 | 7.66E-66 | 1.53E-64 | 138.8191267 |
| EIF4EBP2 | -0.881213088 | 4.825303798 | -18.24044756 | 8.07E-66 | 1.61E-64 | 138.7668964 |
| CCDC82 | -0.986397719 | 1.427451076 | -18.23486078 | 8.74E-66 | 1.74E-64 | 138.6871085 |
| SLC27A6 | -1.463036913 | 0.792650759 | -18.23199939 | 9.11E-66 | 1.81E-64 | 138.6462489 |
| P3H4 | 1.273184979 | 3.405635578 | 18.21671549 | 1.13E-65 | 2.25E-64 | 138.4280615 |
| EPDR1 | -1.477588501 | 2.227405288 | -18.21369034 | 1.18E-65 | 2.35E-64 | 138.3848879 |
| MANF | 0.995938946 | 4.86050015 | 18.20757193 | 1.29E-65 | 2.56E-64 | 138.2975809 |
| ADGRL3 | -0.676887962 | 0.295282271 | -18.20193079 | 1.40E-65 | 2.77E-64 | 138.2170993 |
| ZFP36 | -2.204770619 | 5.946574778 | -18.19641238 | 1.52E-65 | 3.00E-64 | 138.1383822 |
| LRRC15 | 2.703615495 | 3.760831158 | 18.18263147 | 1.85E-65 | 3.65E-64 | 137.9418645 |
| PPARA | -0.930633537 | 1.183523248 | -18.17982916 | 1.92E-65 | 3.79E-64 | 137.9019134 |
| IL17B | -1.324445029 | 0.628104848 | -18.17592333 | 2.03E-65 | 4.00E-64 | 137.846236 |
| RNF125 | -0.969786465 | 1.486868937 | -18.16483102 | 2.38E-65 | 4.68E-64 | 137.6881527 |
| MRPS34 | 1.305970041 | 5.865943993 | 18.11838832 | 4.63E-65 | 9.08E-64 | 137.0268662 |
| SNRPB | 1.158502565 | 6.902327036 | 18.11270164 | 5.02E-65 | 9.83E-64 | 136.9459613 |
| TRPC6 | -0.747984635 | 0.804548608 | -18.09315784 | 6.63E-65 | 1.30E-63 | 136.6680205 |
| IRX6 | -1.135963751 | 0.559051621 | -18.09279162 | 6.67E-65 | 1.30E-63 | 136.6628139 |
| ESYT1 | -0.901534793 | 4.954676939 | -18.08468911 | 7.48E-65 | 1.46E-63 | 136.5476362 |
| GREM2 | -0.892218057 | 0.512136365 | -18.08259735 | 7.71E-65 | 1.50E-63 | 136.5179065 |
| AMOTL1 | -1.455985612 | 2.416601714 | -18.0812845 | 7.85E-65 | 1.53E-63 | 136.4992483 |
| TPD52 | 1.691403072 | 4.727952735 | 18.07660406 | 8.40E-65 | 1.64E-63 | 136.4327361 |
| THRSP | -3.182342638 | 2.655951395 | -18.07496031 | 8.60E-65 | 1.67E-63 | 136.4093797 |
| ANGPTL2 | -1.782559881 | 4.143460929 | -18.06482885 | 9.93E-65 | 1.93E-63 | 136.2654462 |
| NME1 | 1.479670927 | 4.063459112 | 18.06281117 | 1.02E-64 | 1.98E-63 | 136.2367875 |
| STAT5A | -1.361391663 | 3.340549916 | -18.05529367 | 1.14E-64 | 2.20E-63 | 136.1300261 |
| VAMP3 | -0.702206523 | 5.163030933 | -18.04180231 | 1.38E-64 | 2.67E-63 | 135.9384898 |
| GABRD | 1.178699748 | 1.336328946 | 18.0379164 | 1.46E-64 | 2.81E-63 | 135.8833368 |
| BMPER | -0.824141472 | 0.358576864 | -18.03049458 | 1.62E-64 | 3.12E-63 | 135.7780176 |
| SH3GLB1 | -0.741022496 | 4.29346644 | -18.02813377 | 1.67E-64 | 3.23E-63 | 135.7445217 |
| SLC25A22 | 1.154170916 | 2.898285247 | 18.02565673 | 1.74E-64 | 3.34E-63 | 135.7093793 |
| GLDN | -0.950583207 | 0.551031492 | -18.01519016 | 2.01E-64 | 3.87E-63 | 135.5609187 |
| KCNC4 | -0.671987714 | 0.652212309 | -18.0126668 | 2.09E-64 | 4.01E-63 | 135.5251341 |
| HABP4 | -0.850688552 | 1.805287321 | -17.99193815 | 2.80E-64 | 5.38E-63 | 135.2312833 |
| RPL26L1 | 0.860877069 | 3.996952615 | 17.98349792 | 3.16E-64 | 6.06E-63 | 135.1116898 |
| COPG1 | 0.793164873 | 5.979289996 | 17.97109404 | 3.77E-64 | 7.22E-63 | 134.9359921 |
| KCNJ2 | -0.959380701 | 1.054470582 | -17.96640344 | 4.03E-64 | 7.71E-63 | 134.8695691 |
| TBL2 | 0.741952916 | 3.598866125 | 17.95753592 | 4.57E-64 | 8.73E-63 | 134.7440248 |
| ECT2 | 1.410213865 | 3.259805173 | 17.95549689 | 4.71E-64 | 8.97E-63 | 134.7151617 |
| C19orf12 | -0.934064173 | 2.557798521 | -17.95274711 | 4.89E-64 | 9.32E-63 | 134.6762407 |
| TRABD2B | -1.107688129 | 0.817832325 | -17.95022406 | 5.07E-64 | 9.65E-63 | 134.6405321 |
| EXO1 | 1.543225276 | 1.809654614 | 17.94832441 | 5.21E-64 | 9.90E-63 | 134.6136482 |
| SAMD1 | 0.993252101 | 4.464894203 | 17.94216313 | 5.69E-64 | 1.08E-62 | 134.526465 |
| KLHL21 | -1.135002175 | 3.084975749 | -17.9312778 | 6.64E-64 | 1.26E-62 | 134.3724779 |
| PTGER4 | -1.21666512 | 1.766764475 | -17.92899123 | 6.86E-64 | 1.30E-62 | 134.3401383 |
| AVPR1A | -0.956369953 | 1.06415924 | -17.91869935 | 7.93E-64 | 1.50E-62 | 134.1946068 |
| RAB8A | 0.680940985 | 3.38477678 | 17.91718805 | 8.11E-64 | 1.53E-62 | 134.1732404 |
| HSD11B1 | -1.603350417 | 1.37216531 | -17.91484805 | 8.38E-64 | 1.58E-62 | 134.1401603 |
| PPP1CA | 1.024758007 | 5.892803532 | 17.91342953 | 8.55E-64 | 1.61E-62 | 134.1201081 |
| TESC | -1.343803093 | 1.065645079 | -17.90218156 | 1.00E-63 | 1.89E-62 | 133.9611393 |
| ZNF677 | -0.846532587 | 0.819241372 | -17.90193348 | 1.01E-63 | 1.90E-62 | 133.9576338 |
| AURKB | 1.838481178 | 2.31531189 | 17.90016604 | 1.03E-63 | 1.94E-62 | 133.9326599 |
| RCAN1 | -1.869575812 | 3.09158812 | -17.89965039 | 1.04E-63 | 1.96E-62 | 133.925374 |
| SEMA6D | -1.2951225 | 0.977169706 | -17.89215746 | 1.16E-63 | 2.17E-62 | 133.8195163 |
| MFAP4 | -2.754819584 | 4.268032457 | -17.88544781 | 1.27E-63 | 2.39E-62 | 133.7247465 |
| PYCR3 | 1.365089726 | 3.213253146 | 17.87639996 | 1.45E-63 | 2.71E-62 | 133.5969834 |
| NR5A2 | -0.670892198 | 0.648787633 | -17.87631009 | 1.45E-63 | 2.71E-62 | 133.5957145 |
| CD200 | -1.197428731 | 2.388473555 | -17.85944749 | 1.84E-63 | 3.44E-62 | 133.3577017 |
| GTSE1 | 1.386990158 | 1.707959841 | 17.85545888 | 1.94E-63 | 3.64E-62 | 133.3014221 |
| RASIP1 | -1.238277344 | 1.933066523 | -17.85087727 | 2.07E-63 | 3.88E-62 | 133.2367843 |
| CFLAR | -0.848750749 | 2.416260913 | -17.83653428 | 2.54E-63 | 4.74E-62 | 133.0344943 |
| PPP1R16B | -1.393024213 | 1.416423839 | -17.83624338 | 2.55E-63 | 4.76E-62 | 133.0303925 |
| P3H2 | -1.241065273 | 1.268131045 | -17.82668805 | 2.92E-63 | 5.44E-62 | 132.8956802 |
| P2RY14 | -0.793544695 | 0.776413252 | -17.82377246 | 3.04E-63 | 5.67E-62 | 132.8545841 |
| HIST1H2BD | 2.286556803 | 4.954371868 | 17.80975017 | 3.71E-63 | 6.90E-62 | 132.6569909 |
| TANK | -0.893839278 | 3.186300231 | -17.80516552 | 3.96E-63 | 7.35E-62 | 132.5924066 |
| EPM2A | -0.601555439 | 1.221057754 | -17.79831624 | 4.36E-63 | 8.08E-62 | 132.4959382 |
| ACKR1 | -2.95167 | 2.898077426 | -17.7825448 | 5.45E-63 | 1.01E-61 | 132.2738884 |
| ZEB2 | -1.013346612 | 1.47906422 | -17.78235656 | 5.47E-63 | 1.01E-61 | 132.2712388 |
| STX12 | -0.680586812 | 4.092806098 | -17.78192126 | 5.50E-63 | 1.02E-61 | 132.2651117 |
| DAB2IP | -1.079372031 | 3.137399092 | -17.77296269 | 6.24E-63 | 1.15E-61 | 132.1390357 |
| MOB3B | -1.239392894 | 1.417414155 | -17.75567649 | 7.97E-63 | 1.47E-61 | 131.8958677 |
| ETS2 | -1.289752537 | 3.666322176 | -17.74866361 | 8.80E-63 | 1.62E-61 | 131.7972556 |
| QKI | -1.059421483 | 2.565086921 | -17.74280459 | 9.55E-63 | 1.76E-61 | 131.7148862 |
| DDX39A | 1.208938714 | 3.94511212 | 17.72532042 | 1.22E-62 | 2.25E-61 | 131.4691782 |
| TMEM206 | 0.959179619 | 2.40913835 | 17.72043855 | 1.31E-62 | 2.41E-61 | 131.4005978 |
| SPAAR | -1.164105226 | 0.791423529 | -17.71889451 | 1.34E-62 | 2.46E-61 | 131.3789093 |
| EFEMP1 | -2.32909468 | 4.355673748 | -17.7096986 | 1.52E-62 | 2.79E-61 | 131.249762 |
| HSPA12A | -1.080040762 | 1.4755395 | -17.70960104 | 1.53E-62 | 2.79E-61 | 131.2483921 |
| B4GALT3 | 1.038014894 | 4.284969826 | 17.70903333 | 1.54E-62 | 2.81E-61 | 131.2404205 |
| ISG20L2 | 0.779660701 | 3.899854851 | 17.70688881 | 1.59E-62 | 2.90E-61 | 131.2103091 |
| ESM1 | 1.479500136 | 1.937431439 | 17.70568673 | 1.61E-62 | 2.94E-61 | 131.1934317 |
| GBE1 | -1.059539991 | 2.833798428 | -17.70558299 | 1.61E-62 | 2.95E-61 | 131.1919751 |
| INTS7 | 0.959445065 | 3.229997496 | 17.70366182 | 1.66E-62 | 3.02E-61 | 131.1650028 |
| STRBP | 0.910451561 | 3.12209297 | 17.70326737 | 1.67E-62 | 3.04E-61 | 131.1594652 |
| ACTG2 | -3.066665562 | 2.736355946 | -17.70082626 | 1.73E-62 | 3.14E-61 | 131.1251961 |
| MRPL14 | 1.171949457 | 5.457961843 | 17.69719388 | 1.82E-62 | 3.30E-61 | 131.0742089 |
| PARVA | -0.898703473 | 3.781329107 | -17.69207166 | 1.95E-62 | 3.54E-61 | 131.0023191 |
| GRAMD1A | 0.924065618 | 3.986428487 | 17.68897701 | 2.04E-62 | 3.70E-61 | 130.9588921 |
| SMIM10L2B | -0.725418153 | 0.611330254 | -17.67639281 | 2.44E-62 | 4.41E-61 | 130.7823446 |
| SAA4 | -0.984637542 | 0.453613055 | -17.67599741 | 2.45E-62 | 4.43E-61 | 130.7767986 |
| LEPROT | -0.862332706 | 4.483932704 | -17.67184009 | 2.60E-62 | 4.69E-61 | 130.7184913 |
| DUSP6 | -1.622387428 | 3.635509894 | -17.65928783 | 3.10E-62 | 5.59E-61 | 130.542492 |
| DAP3 | 0.790184601 | 5.07443865 | 17.6514411 | 3.46E-62 | 6.23E-61 | 130.4325079 |
| ARHGAP39 | 1.179134684 | 2.510064357 | 17.65094258 | 3.48E-62 | 6.27E-61 | 130.4255213 |
| ALDH2 | -1.771771654 | 3.302427689 | -17.64690017 | 3.69E-62 | 6.63E-61 | 130.3688728 |
| APOLD1 | -1.528333542 | 2.41154031 | -17.64649929 | 3.71E-62 | 6.66E-61 | 130.3632554 |
| GRAMD2B | -1.249153834 | 2.595202495 | -17.61308374 | 5.93E-62 | 1.06E-60 | 129.8952837 |
| MAPK10 | -1.004449866 | 0.790236693 | -17.6127953 | 5.96E-62 | 1.07E-60 | 129.8912464 |
| FOS | -2.889839212 | 5.587843472 | -17.60373696 | 6.76E-62 | 1.21E-60 | 129.7644792 |
| PARD3B | -0.809664877 | 1.535692325 | -17.59322275 | 7.84E-62 | 1.40E-60 | 129.6173859 |
| NFIB | -2.030387271 | 3.265590026 | -17.56362182 | 1.19E-61 | 2.12E-60 | 129.2035492 |
| ARRDC3 | -1.382453638 | 4.214853242 | -17.56267027 | 1.20E-61 | 2.15E-60 | 129.1902529 |
| CHST7 | -0.694398293 | 1.207223482 | -17.55807065 | 1.28E-61 | 2.29E-60 | 129.1259868 |
| GPATCH11 | -0.743582202 | 2.262995038 | -17.54708886 | 1.50E-61 | 2.67E-60 | 128.9725891 |
| PDIA4 | 1.072320738 | 6.161134014 | 17.54414692 | 1.56E-61 | 2.78E-60 | 128.9315045 |
| ASPM | 1.466296309 | 1.657650808 | 17.54008631 | 1.65E-61 | 2.94E-60 | 128.8748044 |
| ALDOC | -1.952305563 | 2.490941315 | -17.52526166 | 2.03E-61 | 3.61E-60 | 128.6678672 |
| CBX4 | 1.396627835 | 4.084317105 | 17.52015977 | 2.18E-61 | 3.87E-60 | 128.596674 |
| TMBIM1 | -0.9944998 | 4.80085155 | -17.51634696 | 2.30E-61 | 4.08E-60 | 128.543477 |
| USP53 | -1.103108031 | 2.145023739 | -17.51427916 | 2.37E-61 | 4.20E-60 | 128.5146294 |
| PRR19 | 0.711847067 | 1.079436255 | 17.50470764 | 2.71E-61 | 4.80E-60 | 128.3811254 |
| PHLDB1 | -0.977122143 | 2.170401328 | -17.4945029 | 3.13E-61 | 5.53E-60 | 128.2388369 |
| MTHFD2 | 1.230447225 | 4.276874515 | 17.49219696 | 3.23E-61 | 5.70E-60 | 128.2066912 |
| CIAO2A | 0.762930186 | 3.986915307 | 17.48442464 | 3.60E-61 | 6.35E-60 | 128.0983603 |
| SHANK3 | -1.077450893 | 2.158043323 | -17.48246825 | 3.70E-61 | 6.52E-60 | 128.0710966 |
| RPUSD1 | 1.115206144 | 3.373578919 | 17.48201899 | 3.72E-61 | 6.55E-60 | 128.0648362 |
| MET | -1.799086863 | 2.054976186 | -17.47578195 | 4.06E-61 | 7.14E-60 | 127.9779319 |
| UNC5B | 1.462984563 | 3.337557945 | 17.4700752 | 4.40E-61 | 7.72E-60 | 127.8984326 |
| GPRIN1 | 1.216343885 | 1.530368411 | 17.46372787 | 4.81E-61 | 8.43E-60 | 127.8100278 |
| NR2F6 | 1.092774907 | 4.542607927 | 17.46173862 | 4.95E-61 | 8.66E-60 | 127.7823256 |
| TPRN | 1.414009372 | 2.821273075 | 17.44799126 | 5.99E-61 | 1.05E-59 | 127.5909326 |
| TLE4 | -0.995877 | 1.395719663 | -17.44249799 | 6.47E-61 | 1.13E-59 | 127.5144794 |
| ST6GALNAC6 | -0.952531592 | 3.418394858 | -17.44029918 | 6.67E-61 | 1.16E-59 | 127.4838814 |
| PENK | -0.999231407 | 0.301638901 | -17.43911411 | 6.78E-61 | 1.18E-59 | 127.4673912 |
| AHNAK | -1.589772818 | 6.162759207 | -17.42235411 | 8.57E-61 | 1.49E-59 | 127.2342478 |
| EIPR1 | 0.64810577 | 2.363630858 | 17.42088787 | 8.75E-61 | 1.52E-59 | 127.2138578 |
| KIF18B | 1.389238916 | 1.548633534 | 17.41389778 | 9.65E-61 | 1.68E-59 | 127.1166652 |
| RETSAT | -1.214521375 | 4.80544645 | -17.40739536 | 1.06E-60 | 1.83E-59 | 127.0262741 |
| PITX1 | 2.292578593 | 2.652603239 | 17.40659354 | 1.07E-60 | 1.85E-59 | 127.0151293 |
| GPR34 | -1.432592617 | 2.2542341 | -17.40503376 | 1.09E-60 | 1.89E-59 | 126.9934502 |
| F3 | -2.001325067 | 2.954557873 | -17.40290984 | 1.12E-60 | 1.95E-59 | 126.9639321 |
| FAM111B | 1.73651716 | 2.457292462 | 17.38196694 | 1.51E-60 | 2.61E-59 | 126.6729838 |
| GULP1 | -0.818417098 | 0.808930873 | -17.37724327 | 1.61E-60 | 2.78E-59 | 126.6073895 |
| LPCAT2 | -1.112042333 | 2.000297412 | -17.37507308 | 1.66E-60 | 2.86E-59 | 126.5772571 |
| NCALD | -1.697884374 | 2.079927165 | -17.37124665 | 1.75E-60 | 3.02E-59 | 126.5241339 |
| PTN | -2.822801933 | 3.219829242 | -17.36279297 | 1.97E-60 | 3.39E-59 | 126.4067943 |
| POLR2H | 0.887796474 | 4.022755072 | 17.35942502 | 2.06E-60 | 3.55E-59 | 126.3600557 |
| SDS | 1.386669525 | 1.995520591 | 17.35722039 | 2.13E-60 | 3.66E-59 | 126.329464 |
| TLR4 | -1.245072785 | 2.117475237 | -17.35040305 | 2.34E-60 | 4.02E-59 | 126.2348803 |
| CYP26B1 | -1.22023734 | 1.04844512 | -17.34716904 | 2.44E-60 | 4.20E-59 | 126.1900195 |
| GPM6B | -1.548294708 | 1.786108268 | -17.33671394 | 2.83E-60 | 4.86E-59 | 126.0450251 |
| AK3 | -0.886073828 | 3.794621187 | -17.33173604 | 3.03E-60 | 5.20E-59 | 125.9760086 |
| KRT8 | 2.05647792 | 7.495655692 | 17.32929077 | 3.14E-60 | 5.37E-59 | 125.9421102 |
| TRAF2 | 0.977814886 | 3.120992756 | 17.32733255 | 3.22E-60 | 5.51E-59 | 125.914966 |
| NAALADL1 | -0.587832477 | 0.73522599 | -17.32162994 | 3.49E-60 | 5.96E-59 | 125.8359283 |
| DPM2 | 0.889562776 | 4.148393396 | 17.3169935 | 3.72E-60 | 6.36E-59 | 125.7716791 |
| MRPL17 | 0.877477423 | 4.577106595 | 17.31505008 | 3.82E-60 | 6.52E-59 | 125.7447515 |
| DUSP1 | -2.242966573 | 5.939033718 | -17.30444295 | 4.43E-60 | 7.55E-59 | 125.5978126 |
| CARD6 | -0.972518112 | 1.58963669 | -17.30239509 | 4.56E-60 | 7.76E-59 | 125.5694502 |
| TMEM170B | -0.81195087 | 1.100279986 | -17.30085669 | 4.66E-60 | 7.92E-59 | 125.5481449 |
| CDNF | -0.739257229 | 1.060619177 | -17.2994426 | 4.75E-60 | 8.07E-59 | 125.5285623 |
| FKBP4 | 1.37295987 | 6.04384599 | 17.29573206 | 5.00E-60 | 8.49E-59 | 125.4771825 |
| NABP2 | 0.717059152 | 4.220692056 | 17.29412119 | 5.11E-60 | 8.67E-59 | 125.4548789 |
| KNOP1 | 0.7579655 | 2.350987771 | 17.28900984 | 5.49E-60 | 9.30E-59 | 125.384117 |
| ATP13A1 | 0.768669301 | 3.487174055 | 17.28604047 | 5.72E-60 | 9.69E-59 | 125.3430146 |
| VAMP8 | 1.086112101 | 6.900406721 | 17.28286544 | 5.98E-60 | 1.01E-58 | 125.29907 |
| KLF6 | -1.309487491 | 4.955585785 | -17.27907697 | 6.30E-60 | 1.07E-58 | 125.2466415 |
| COA6 | 1.05864104 | 4.109869022 | 17.27039291 | 7.11E-60 | 1.20E-58 | 125.126489 |
| TMEM47 | -1.725702418 | 2.815228464 | -17.26856529 | 7.29E-60 | 1.23E-58 | 125.1012067 |
| PLPP7 | -0.78172656 | 0.927066827 | -17.2674606 | 7.40E-60 | 1.25E-58 | 125.0859258 |
| RHPN1 | 1.684881427 | 3.202981016 | 17.25535471 | 8.76E-60 | 1.48E-58 | 124.9185069 |
| PAK4 | 1.113526589 | 3.750602819 | 17.24982632 | 9.46E-60 | 1.59E-58 | 124.8420752 |
| ATIC | 0.785506707 | 4.867335709 | 17.24634435 | 9.93E-60 | 1.67E-58 | 124.7939436 |
| RIMS3 | -0.962052293 | 0.948983839 | -17.24591218 | 9.99E-60 | 1.68E-58 | 124.7879701 |
| LYSMD4 | -0.611561871 | 1.035619839 | -17.24007992 | 1.08E-59 | 1.82E-58 | 124.7073644 |
| CCDC158 | -0.596468218 | 0.429216578 | -17.23219067 | 1.21E-59 | 2.03E-58 | 124.5983559 |
| PDE1B | -0.784524542 | 0.892691771 | -17.2230017 | 1.37E-59 | 2.30E-58 | 124.4714269 |
| ATF3 | -2.124041317 | 3.14814624 | -17.22054207 | 1.42E-59 | 2.38E-58 | 124.4374585 |
| HGH1 | 1.188189056 | 3.934903989 | 17.21536437 | 1.53E-59 | 2.55E-58 | 124.3659621 |
| RNPEP | 0.902830765 | 5.231026886 | 17.21233725 | 1.59E-59 | 2.66E-58 | 124.324168 |
| FBXL19 | 1.00316392 | 2.892889719 | 17.19657656 | 1.98E-59 | 3.31E-58 | 124.1066391 |
| CSRP1 | -1.245262578 | 4.920377178 | -17.19629139 | 1.99E-59 | 3.32E-58 | 124.1027042 |
| SHROOM4 | -0.817503101 | 1.193991991 | -17.1873567 | 2.25E-59 | 3.75E-58 | 123.9794425 |
| PGP | 1.016245645 | 2.989548964 | 17.18434814 | 2.34E-59 | 3.90E-58 | 123.9379456 |
| SNTB2 | -0.8034071 | 2.249421467 | -17.18064475 | 2.47E-59 | 4.11E-58 | 123.8868708 |
| TRIB3 | 1.387167781 | 3.625625958 | 17.17879894 | 2.53E-59 | 4.21E-58 | 123.8614172 |
| RGS2 | -2.174394774 | 3.63534417 | -17.17422625 | 2.70E-59 | 4.48E-58 | 123.7983671 |
| SLC12A8 | 1.018982953 | 2.220923999 | 17.17368817 | 2.72E-59 | 4.51E-58 | 123.7909484 |
| NAP1L2 | -1.123932983 | 0.95685643 | -17.16615144 | 3.02E-59 | 5.00E-58 | 123.687053 |
| ABCG2 | -0.906110004 | 0.905577334 | -17.16389563 | 3.11E-59 | 5.16E-58 | 123.6559615 |
| ZCCHC24 | -1.431502755 | 3.084813431 | -17.15926192 | 3.32E-59 | 5.49E-58 | 123.5921036 |
| IL6 | -1.665179696 | 1.004768357 | -17.15863187 | 3.35E-59 | 5.54E-58 | 123.5834216 |
| ARHGEF28 | -0.796661546 | 1.221403517 | -17.15360498 | 3.59E-59 | 5.93E-58 | 123.5141587 |
| PSENEN | 1.00617786 | 4.955745742 | 17.15070051 | 3.73E-59 | 6.17E-58 | 123.4741449 |
| PRELP | -2.163788815 | 3.076135733 | -17.13917185 | 4.38E-59 | 7.23E-58 | 123.3153602 |
| FER | -0.689557809 | 1.312039581 | -17.13762041 | 4.47E-59 | 7.38E-58 | 123.2939971 |
| ALDH1A3 | -1.614363358 | 1.875181746 | -17.13424086 | 4.69E-59 | 7.72E-58 | 123.2474651 |
| PIK3R1 | -1.541417816 | 3.732324204 | -17.13345529 | 4.74E-59 | 7.80E-58 | 123.2366498 |
| PRNP | -1.569047502 | 4.884142959 | -17.11621627 | 6.01E-59 | 9.89E-58 | 122.9993849 |
| ODF2 | 0.794918785 | 3.554733393 | 17.10705425 | 6.82E-59 | 1.12E-57 | 122.8733448 |
| FLVCR1 | 0.966542603 | 2.264267044 | 17.10255668 | 7.26E-59 | 1.19E-57 | 122.8114878 |
| TMEM132A | 1.750735759 | 3.489301387 | 17.10053901 | 7.47E-59 | 1.22E-57 | 122.7837411 |
| MCM2 | 1.417709459 | 3.583987229 | 17.09938685 | 7.59E-59 | 1.24E-57 | 122.7678976 |
| BBOX1 | -2.086819288 | 1.336810613 | -17.09312821 | 8.27E-59 | 1.35E-57 | 122.6818459 |
| CHPF | 1.27105723 | 5.174990666 | 17.08174853 | 9.68E-59 | 1.58E-57 | 122.5254327 |
| CDC45 | 1.466450213 | 1.928030781 | 17.08072395 | 9.81E-59 | 1.60E-57 | 122.5113529 |
| RHOU | -1.602709036 | 2.612112095 | -17.07966266 | 9.96E-59 | 1.62E-57 | 122.4967693 |
| FBXL6 | 1.255250412 | 2.934320149 | 17.06963182 | 1.14E-58 | 1.86E-57 | 122.3589588 |
| MT1X | -2.04108973 | 4.016666038 | -17.0652585 | 1.21E-58 | 1.98E-57 | 122.2988905 |
| FHOD3 | -1.011416219 | 1.158428655 | -17.05861336 | 1.33E-58 | 2.17E-57 | 122.2076365 |
| PTGIS | -1.709019968 | 2.303948869 | -17.05208195 | 1.46E-58 | 2.37E-57 | 122.1179654 |
| MPDZ | -1.036183435 | 2.161451562 | -17.04733106 | 1.55E-58 | 2.53E-57 | 122.0527525 |
| PNPLA2 | -1.422254414 | 5.03954235 | -17.02659504 | 2.07E-58 | 3.36E-57 | 121.7682505 |
| FAAP24 | 0.709735625 | 1.828911762 | 17.01791215 | 2.33E-58 | 3.78E-57 | 121.6491826 |
| NGFR | -1.982244371 | 1.893242512 | -17.00412004 | 2.82E-58 | 4.56E-57 | 121.4601286 |
| VLDLR | -1.262682403 | 1.65514045 | -17.00294332 | 2.86E-58 | 4.63E-57 | 121.4440031 |
| CDCA3 | 1.301798641 | 1.618835546 | 16.99992285 | 2.99E-58 | 4.83E-57 | 121.4026146 |
| SNRPE | 1.013654444 | 5.494171687 | 16.99841863 | 3.05E-58 | 4.92E-57 | 121.3820045 |
| LAMA3 | -1.876553471 | 2.017345859 | -16.98997793 | 3.42E-58 | 5.53E-57 | 121.2663744 |
| SNX33 | -0.686699362 | 2.900044972 | -16.96845578 | 4.60E-58 | 7.42E-57 | 120.9716993 |
| GIMAP1 | -1.00215335 | 1.326042127 | -16.96163656 | 5.05E-58 | 8.14E-57 | 120.8783804 |
| RITA1 | 0.741556705 | 3.537998661 | 16.96061948 | 5.13E-58 | 8.24E-57 | 120.864464 |
| FLT4 | -0.82413391 | 1.397849409 | -16.95870109 | 5.26E-58 | 8.46E-57 | 120.8382165 |
| IQCN | -0.853443309 | 0.752785024 | -16.94588337 | 6.28E-58 | 1.01E-56 | 120.6628907 |
| WIF1 | -2.162658792 | 0.8061332 | -16.94223339 | 6.60E-58 | 1.06E-56 | 120.6129798 |
| SLC25A39 | 1.03384923 | 5.312181465 | 16.93651492 | 7.14E-58 | 1.14E-56 | 120.534797 |
| KLF10 | -1.12380287 | 4.349001208 | -16.93182504 | 7.61E-58 | 1.22E-56 | 120.4706891 |
| TLCD2 | -0.973195539 | 1.504637804 | -16.92462915 | 8.40E-58 | 1.34E-56 | 120.3723467 |
| EVC2 | -0.705397807 | 0.871922195 | -16.92421424 | 8.45E-58 | 1.35E-56 | 120.3666772 |
| APOO | 0.928719594 | 3.839512359 | 16.92195507 | 8.71E-58 | 1.39E-56 | 120.3358082 |
| SMARCA4 | 0.854349146 | 4.012728293 | 16.9189221 | 9.08E-58 | 1.45E-56 | 120.2943699 |
| LMNB2 | 1.156269455 | 3.679772002 | 16.91872964 | 9.11E-58 | 1.45E-56 | 120.2917406 |
| PSMA5 | 0.755731103 | 4.117777901 | 16.91691444 | 9.34E-58 | 1.49E-56 | 120.2669426 |
| SKA1 | 1.371255704 | 1.609727311 | 16.91491622 | 9.60E-58 | 1.53E-56 | 120.2396462 |
| STXBP2 | 1.190531204 | 3.156456926 | 16.91130465 | 1.01E-57 | 1.60E-56 | 120.190316 |
| ERCC6L | 0.998906567 | 1.216557144 | 16.89181737 | 1.32E-57 | 2.09E-56 | 119.9242522 |
| EPCAM | 1.678168569 | 6.269882078 | 16.88699036 | 1.41E-57 | 2.23E-56 | 119.8583771 |
| NUDT16L1 | 1.132526928 | 4.105989591 | 16.8832682 | 1.48E-57 | 2.35E-56 | 119.8075882 |
| KLHL30 | -0.596692463 | 0.258913043 | -16.88090385 | 1.53E-57 | 2.42E-56 | 119.7753302 |
| SHCBP1 | 1.222976171 | 1.646311057 | 16.8685465 | 1.81E-57 | 2.86E-56 | 119.6067779 |
| ADGRA2 | -1.298830631 | 2.560112613 | -16.86478598 | 1.91E-57 | 3.01E-56 | 119.5555 |
| EIF3L | -1.123936796 | 5.172602854 | -16.85007059 | 2.33E-57 | 3.68E-56 | 119.3549115 |
| MEF2C | -0.967208209 | 1.870956419 | -16.84733857 | 2.42E-57 | 3.82E-56 | 119.3176825 |
| TOMM40 | 1.04309007 | 4.060197583 | 16.83442384 | 2.89E-57 | 4.55E-56 | 119.1417458 |
| MBNL1 | -0.891415751 | 3.84081476 | -16.82774362 | 3.17E-57 | 4.98E-56 | 119.0507742 |
| PIGR | -3.264114292 | 2.192794929 | -16.82727342 | 3.19E-57 | 5.01E-56 | 119.0443719 |
| CSF3 | -0.648702991 | 0.148128459 | -16.81463643 | 3.79E-57 | 5.95E-56 | 118.8723448 |
| MAD2L1 | 1.263466559 | 2.148154948 | 16.80836827 | 4.13E-57 | 6.48E-56 | 118.7870461 |
| CALR | 0.971449458 | 8.303669007 | 16.80759998 | 4.17E-57 | 6.54E-56 | 118.7765925 |
| TRIP13 | 1.49804334 | 2.377343809 | 16.80563642 | 4.28E-57 | 6.71E-56 | 118.7498766 |
| VAV2 | 0.921312035 | 3.512740171 | 16.80356119 | 4.41E-57 | 6.90E-56 | 118.7216437 |
| CACFD1 | 1.242101913 | 3.73802723 | 16.80287313 | 4.45E-57 | 6.96E-56 | 118.7122832 |
| LETMD1 | -0.746820996 | 3.122114078 | -16.79658993 | 4.84E-57 | 7.57E-56 | 118.626817 |
| BCL2L2 | -0.840485064 | 3.651540527 | -16.7868437 | 5.53E-57 | 8.65E-56 | 118.4942845 |
| MFAP2 | 1.765064609 | 3.539157514 | 16.78457727 | 5.71E-57 | 8.91E-56 | 118.4634718 |
| WBP4 | -0.626907565 | 2.714716614 | -16.76721857 | 7.23E-57 | 1.13E-55 | 118.2275605 |
| SKAP2 | -1.169551971 | 2.669900969 | -16.76163995 | 7.81E-57 | 1.22E-55 | 118.1517771 |
| ESPL1 | 1.127324394 | 1.435281616 | 16.76041284 | 7.94E-57 | 1.24E-55 | 118.1351094 |
| ACKR4 | -0.833964533 | 0.746226839 | -16.75653959 | 8.37E-57 | 1.30E-55 | 118.0825044 |
| NMRAL1 | 1.0111612 | 3.92684378 | 16.75464243 | 8.59E-57 | 1.34E-55 | 118.0567407 |
| PCCA | -0.896199101 | 2.11228378 | -16.7525584 | 8.83E-57 | 1.37E-55 | 118.0284413 |
| MCM4 | 1.438060712 | 4.148856279 | 16.74682138 | 9.55E-57 | 1.48E-55 | 117.9505487 |
| CLDN8 | -2.255669697 | 1.959764346 | -16.74565279 | 9.71E-57 | 1.51E-55 | 117.9346846 |
| PTPA | 0.873944635 | 5.32030308 | 16.73293585 | 1.15E-56 | 1.79E-55 | 117.7620908 |
| ADD3 | -1.475586664 | 3.315212394 | -16.72819492 | 1.23E-56 | 1.91E-55 | 117.697768 |
| NANS | 0.986777723 | 3.856670134 | 16.72374978 | 1.31E-56 | 2.02E-55 | 117.6374687 |
| FLNC | -1.185456891 | 1.243778625 | -16.71918594 | 1.39E-56 | 2.15E-55 | 117.5755694 |
| NVL | 1.014497551 | 3.307517683 | 16.71210212 | 1.53E-56 | 2.37E-55 | 117.4795127 |
| TMEM43 | -0.76153769 | 4.991037268 | -16.70149122 | 1.77E-56 | 2.73E-55 | 117.335676 |
| KNL1 | 0.941571884 | 1.130963011 | 16.69893422 | 1.83E-56 | 2.83E-55 | 117.3010231 |
| RAPGEF2 | -0.773480877 | 2.022608075 | -16.69213608 | 2.01E-56 | 3.10E-55 | 117.2089092 |
| JAM3 | -1.118436397 | 2.507555172 | -16.68985828 | 2.07E-56 | 3.19E-55 | 117.1780506 |
| TUFT1 | 1.226054276 | 3.888157752 | 16.68670053 | 2.17E-56 | 3.33E-55 | 117.1352751 |
| WDR62 | 0.905088362 | 1.110396828 | 16.68043692 | 2.36E-56 | 3.62E-55 | 117.0504421 |
| FAM110A | 1.258981586 | 3.557554568 | 16.67818687 | 2.43E-56 | 3.73E-55 | 117.0199727 |
| TBC1D4 | -1.201451323 | 3.007362669 | -16.67503957 | 2.54E-56 | 3.89E-55 | 116.9773573 |
| TMEM9 | 1.015018386 | 5.139035431 | 16.66987856 | 2.72E-56 | 4.17E-55 | 116.9074867 |
| TENM2 | -0.93804663 | 0.596493041 | -16.66219908 | 3.02E-56 | 4.62E-55 | 116.8035456 |
| TSEN54 | 1.033351961 | 3.49206398 | 16.65059647 | 3.54E-56 | 5.41E-55 | 116.6465619 |
| BAX | 0.996975764 | 3.960032059 | 16.63720732 | 4.24E-56 | 6.48E-55 | 116.4654913 |
| NRIP2 | -0.824121502 | 1.442645389 | -16.6223375 | 5.19E-56 | 7.93E-55 | 116.2645033 |
| APH1A | 0.804550753 | 6.377750055 | 16.61702926 | 5.58E-56 | 8.51E-55 | 116.1927819 |
| STOX2 | -0.679703885 | 0.547918746 | -16.61142 | 6.02E-56 | 9.18E-55 | 116.1170086 |
| HSD17B11 | -1.291649203 | 3.20669468 | -16.5963068 | 7.39E-56 | 1.13E-54 | 115.9129304 |
| STARD8 | -0.78320747 | 1.478702058 | -16.59517405 | 7.50E-56 | 1.14E-54 | 115.8976393 |
| NUP62 | 0.669706074 | 3.91239358 | 16.5942367 | 7.60E-56 | 1.16E-54 | 115.8849862 |
| RUVBL1 | 0.830895184 | 3.239181766 | 16.58541713 | 8.56E-56 | 1.30E-54 | 115.765956 |
| ALPK3 | -0.795630112 | 0.876805449 | -16.5839479 | 8.73E-56 | 1.33E-54 | 115.7461308 |
| LAMA2 | -1.595540799 | 2.10127681 | -16.57787125 | 9.48E-56 | 1.44E-54 | 115.6641472 |
| MAFF | -1.207094376 | 2.176141652 | -16.56901581 | 1.07E-55 | 1.62E-54 | 115.5447069 |
| SRP9 | 0.899952692 | 6.975937077 | 16.56674192 | 1.10E-55 | 1.67E-54 | 115.5140435 |
| BACH2 | -0.700157311 | 0.678915072 | -16.56401216 | 1.14E-55 | 1.73E-54 | 115.4772364 |
| PER2 | -1.043353013 | 2.712321116 | -16.55175761 | 1.35E-55 | 2.04E-54 | 115.3120472 |
| NECTIN2 | 1.229375483 | 5.760524792 | 16.55156983 | 1.35E-55 | 2.04E-54 | 115.3095165 |
| GALE | 1.233046014 | 3.182752473 | 16.55063696 | 1.37E-55 | 2.07E-54 | 115.2969447 |
| TIMM17B | 0.995987578 | 3.908396931 | 16.5491704 | 1.40E-55 | 2.11E-54 | 115.2771817 |
| GRASP | -1.110151717 | 1.685880882 | -16.53568363 | 1.68E-55 | 2.53E-54 | 115.0954891 |
| DLG3 | 0.908289357 | 3.46637331 | 16.53454445 | 1.70E-55 | 2.56E-54 | 115.0801464 |
| DCN | -2.187856431 | 5.330534788 | -16.53330884 | 1.73E-55 | 2.60E-54 | 115.0635057 |
| OPRPN | -2.2735888 | 0.865573 | -16.52929437 | 1.83E-55 | 2.75E-54 | 115.009446 |
| RFC2 | 0.86954452 | 4.071783969 | 16.52187981 | 2.02E-55 | 3.03E-54 | 114.9096216 |
| NECAB1 | -0.830935602 | 0.442056627 | -16.49933511 | 2.74E-55 | 4.11E-54 | 114.6062691 |
| PKM | 0.946973614 | 6.810793698 | 16.48837772 | 3.18E-55 | 4.76E-54 | 114.4589251 |
| LPAR6 | -1.041823685 | 2.964792018 | -16.47871986 | 3.62E-55 | 5.41E-54 | 114.3291071 |
| CENPA | 1.508142694 | 1.857083093 | 16.44649265 | 5.60E-55 | 8.35E-54 | 113.8962665 |
| C6 | -1.051157904 | 0.485797622 | -16.44596996 | 5.64E-55 | 8.40E-54 | 113.8892507 |
| PKDCC | -1.399080417 | 1.601817838 | -16.44136242 | 6.00E-55 | 8.93E-54 | 113.8274122 |
| COL1A1 | 2.395863352 | 8.723837779 | 16.44112503 | 6.02E-55 | 8.95E-54 | 113.8242265 |
| ARHGEF10 | -0.827091663 | 1.870829019 | -16.42962759 | 7.02E-55 | 1.04E-53 | 113.6699669 |
| AMPD1 | -0.666774687 | 0.30895671 | -16.42790413 | 7.19E-55 | 1.07E-53 | 113.6468494 |
| ITGA1 | -0.955624862 | 2.062440597 | -16.42365562 | 7.61E-55 | 1.13E-53 | 113.5898689 |
| IFFO2 | -1.320662692 | 2.385948842 | -16.42287385 | 7.69E-55 | 1.14E-53 | 113.5793849 |
| PRKAR2B | -1.867472409 | 3.606542893 | -16.40374875 | 9.95E-55 | 1.47E-53 | 113.3230044 |
| PFKFB3 | -1.670857889 | 4.388568572 | -16.40363954 | 9.96E-55 | 1.48E-53 | 113.3215409 |
| ARHGAP19 | -0.759098732 | 2.047461867 | -16.39581085 | 1.11E-54 | 1.64E-53 | 113.2166489 |
| COBLL1 | -1.013706953 | 1.735177127 | -16.38927117 | 1.21E-54 | 1.79E-53 | 113.1290519 |
| BCL6 | -1.165738842 | 3.485807855 | -16.38333503 | 1.31E-54 | 1.93E-53 | 113.0495584 |
| HMGN1 | 0.841186344 | 5.246963814 | 16.3767349 | 1.43E-54 | 2.11E-53 | 112.9611945 |
| SF3B4 | 0.880814559 | 5.681217066 | 16.36923751 | 1.58E-54 | 2.33E-53 | 112.8608453 |
| TBC1D10B | 0.659870041 | 3.637484286 | 16.36517654 | 1.67E-54 | 2.46E-53 | 112.8065033 |
| EVPL | 1.279700952 | 2.800619404 | 16.35980253 | 1.80E-54 | 2.64E-53 | 112.7346039 |
| IGSF9 | 1.461729892 | 2.896474716 | 16.35870172 | 1.82E-54 | 2.68E-53 | 112.7198779 |
| RASL10A | -0.704675681 | 0.551122278 | -16.35833465 | 1.83E-54 | 2.69E-53 | 112.7149675 |
| NIPSNAP1 | 0.945244866 | 4.781055082 | 16.34110788 | 2.31E-54 | 3.39E-53 | 112.4846033 |
| SEPT10 | -1.015227172 | 3.75950087 | -16.33946402 | 2.36E-54 | 3.46E-53 | 112.4626289 |
| TARS2 | 0.873686338 | 3.713737264 | 16.33273179 | 2.58E-54 | 3.78E-53 | 112.3726503 |
| SELENOP | -1.812329649 | 4.03773308 | -16.32608912 | 2.82E-54 | 4.13E-53 | 112.2838917 |
| CPM | -1.401166994 | 2.25025348 | -16.32433901 | 2.89E-54 | 4.23E-53 | 112.2605108 |
| ARPC5L | 0.794360882 | 3.812187892 | 16.32377518 | 2.91E-54 | 4.26E-53 | 112.2529785 |
| CCL23 | -0.616956552 | 0.394594152 | -16.32157278 | 3.00E-54 | 4.38E-53 | 112.223558 |
| DPYD | -1.199961201 | 2.296358065 | -16.32072263 | 3.04E-54 | 4.43E-53 | 112.212202 |
| KLHL36 | -0.777281517 | 2.368271148 | -16.31049043 | 3.48E-54 | 5.08E-53 | 112.0755543 |
| SELP | -1.608768237 | 1.59397179 | -16.2911414 | 4.51E-54 | 6.57E-53 | 111.8173034 |
| ABHD6 | -0.83335272 | 1.56572845 | -16.28928331 | 4.63E-54 | 6.73E-53 | 111.7925138 |
| ZNF502 | -0.935664049 | 1.238894419 | -16.28722725 | 4.76E-54 | 6.91E-53 | 111.7650851 |
| FOXO4 | -0.963983059 | 3.018529408 | -16.28384361 | 4.98E-54 | 7.22E-53 | 111.7199508 |
| PSMD14 | 0.686746731 | 3.29346522 | 16.28294763 | 5.04E-54 | 7.31E-53 | 111.7080003 |
| ZHX3 | -0.762280559 | 2.040364916 | -16.26929198 | 6.05E-54 | 8.77E-53 | 111.5259151 |
| C1orf43 | 0.744777992 | 6.870754327 | 16.26719272 | 6.22E-54 | 9.01E-53 | 111.4979321 |
| PDGFRA | -1.457624889 | 2.462099581 | -16.25795718 | 7.04E-54 | 1.02E-52 | 111.3748503 |
| ITGA9 | -1.131743809 | 1.817475519 | -16.25777376 | 7.05E-54 | 1.02E-52 | 111.3724063 |
| SLIT2 | -1.075466649 | 1.516765244 | -16.25515262 | 7.31E-54 | 1.06E-52 | 111.3374828 |
| ALG3 | 0.922518897 | 3.894430004 | 16.25308748 | 7.51E-54 | 1.08E-52 | 111.30997 |
| CEBPA | -1.437799245 | 2.793807861 | -16.24757758 | 8.08E-54 | 1.17E-52 | 111.236575 |
| IGIP | -0.851912406 | 2.252133727 | -16.24306069 | 8.59E-54 | 1.24E-52 | 111.1764194 |
| SGK1 | -1.279357754 | 2.677909747 | -16.22122687 | 1.15E-53 | 1.65E-52 | 110.8857892 |
| FNDC4 | -1.259022263 | 2.026838948 | -16.22021686 | 1.17E-53 | 1.68E-52 | 110.872351 |
| SLC37A1 | 0.900801145 | 2.591762598 | 16.2195907 | 1.18E-53 | 1.69E-52 | 110.8640202 |
| GPRC5B | -1.385928089 | 2.569721073 | -16.21588738 | 1.23E-53 | 1.77E-52 | 110.8147533 |
| DEPDC1B | 1.027672969 | 1.227676538 | 16.2137376 | 1.27E-53 | 1.82E-52 | 110.7861572 |
| TCP11L2 | -0.727336157 | 1.123806903 | -16.21248427 | 1.29E-53 | 1.85E-52 | 110.7694866 |
| P4HA3 | 1.160575303 | 1.564009417 | 16.21038603 | 1.33E-53 | 1.90E-52 | 110.7415798 |
| SLC12A4 | -0.844968612 | 2.513930951 | -16.19226829 | 1.69E-53 | 2.42E-52 | 110.5007078 |
| DES | -1.850416839 | 0.928184447 | -16.17754641 | 2.06E-53 | 2.94E-52 | 110.3051104 |
| B4GALT6 | -0.604348627 | 0.773245602 | -16.17644598 | 2.09E-53 | 2.99E-52 | 110.2904946 |
| YAP1 | -1.167798269 | 4.126904818 | -16.17570612 | 2.11E-53 | 3.01E-52 | 110.280668 |
| REEP4 | 1.100369646 | 3.564758603 | 16.16916648 | 2.30E-53 | 3.29E-52 | 110.1938243 |
| CLIC2 | -1.156052427 | 2.374153411 | -16.15607305 | 2.74E-53 | 3.91E-52 | 110.0200167 |
| MAF | -1.030439713 | 2.562603155 | -16.15561136 | 2.76E-53 | 3.93E-52 | 110.0138897 |
| TYMS | 1.466048082 | 3.223848657 | 16.15346101 | 2.84E-53 | 4.04E-52 | 109.9853542 |
| ACAA2 | -0.991212288 | 3.381410379 | -16.14270115 | 3.28E-53 | 4.66E-52 | 109.8426061 |
| CREBRF | -0.798374782 | 1.851848023 | -16.13774947 | 3.50E-53 | 4.97E-52 | 109.776934 |
| SGO1 | 1.048573215 | 1.190759028 | 16.13648355 | 3.56E-53 | 5.05E-52 | 109.7601468 |
| C1GALT1C1L | -0.77535204 | 0.814864143 | -16.12635693 | 4.07E-53 | 5.77E-52 | 109.6258888 |
| ROGDI | 1.231193274 | 3.482200769 | 16.1244432 | 4.18E-53 | 5.92E-52 | 109.6005229 |
| CCL21 | -2.904751751 | 2.92871039 | -16.12328075 | 4.24E-53 | 6.01E-52 | 109.5851158 |
| BLOC1S3 | 0.6499792 | 3.130017496 | 16.12132753 | 4.36E-53 | 6.16E-52 | 109.5592296 |
| CDT1 | 1.469219847 | 2.248955346 | 16.12002465 | 4.43E-53 | 6.26E-52 | 109.5419636 |
| LIMK1 | 0.829342447 | 3.107294638 | 16.11848745 | 4.52E-53 | 6.39E-52 | 109.5215933 |
| YDJC | 1.147721593 | 3.323956978 | 16.11416039 | 4.79E-53 | 6.76E-52 | 109.4642601 |
| NAT14 | 1.314410181 | 3.125129019 | 16.11057375 | 5.03E-53 | 7.09E-52 | 109.4167449 |
| TMEM245 | -0.884299785 | 3.293593939 | -16.10539599 | 5.38E-53 | 7.59E-52 | 109.3481628 |
| ARHGAP6 | -0.608526183 | 0.613886883 | -16.10422373 | 5.47E-53 | 7.70E-52 | 109.3326377 |
| SYNGR2 | 1.05805379 | 5.919047977 | 16.10353292 | 5.52E-53 | 7.77E-52 | 109.323489 |
| ACSM3 | -0.739934435 | 0.610141994 | -16.09951056 | 5.82E-53 | 8.19E-52 | 109.2702246 |
| GGCT | 1.198038349 | 5.457575287 | 16.09873803 | 5.88E-53 | 8.27E-52 | 109.2599957 |
| ARHGAP11A | 1.19985447 | 1.887810545 | 16.09723534 | 6.00E-53 | 8.43E-52 | 109.2400998 |
| PTCHD1 | -0.801368454 | 0.331173505 | -16.08781537 | 6.80E-53 | 9.54E-52 | 109.1154048 |
| PPP1R15A | -1.174272785 | 4.184069808 | -16.07735682 | 7.82E-53 | 1.10E-51 | 108.977017 |
| DBNDD1 | 1.414961547 | 3.011214193 | 16.07180465 | 8.41E-53 | 1.18E-51 | 108.9035741 |
| TEAD1 | -1.102720955 | 2.916939161 | -16.0700418 | 8.61E-53 | 1.21E-51 | 108.8802589 |
| ZNF48 | 0.803968686 | 2.626616707 | 16.06965673 | 8.66E-53 | 1.21E-51 | 108.8751663 |
| PTCH2 | -0.686502436 | 0.696384785 | -16.06315723 | 9.44E-53 | 1.32E-51 | 108.7892206 |
| DIAPH2 | -0.805781648 | 1.642401045 | -16.05598564 | 1.04E-52 | 1.45E-51 | 108.6944137 |
| DTYMK | 0.894232486 | 3.502497919 | 16.05563775 | 1.04E-52 | 1.45E-51 | 108.6898153 |
| PSRC1 | 1.112833538 | 2.107702134 | 16.0490951 | 1.14E-52 | 1.58E-51 | 108.603348 |
| APBA1 | -0.739342289 | 0.7297077 | -16.0484032 | 1.15E-52 | 1.60E-51 | 108.5942051 |
| ZNF672 | 0.756636311 | 3.7009856 | 16.04265775 | 1.24E-52 | 1.72E-51 | 108.5182945 |
| GINS2 | 1.392364761 | 2.547151728 | 16.03452093 | 1.38E-52 | 1.92E-51 | 108.4108183 |
| FADS3 | -1.011407291 | 1.978010241 | -16.03115611 | 1.44E-52 | 2.00E-51 | 108.366384 |
| SSX2IP | 0.855970695 | 2.172037584 | 16.02824847 | 1.50E-52 | 2.08E-51 | 108.3279919 |
| FARSA | 0.806272835 | 4.759349561 | 16.02208536 | 1.63E-52 | 2.26E-51 | 108.2466299 |
| TMED9 | 0.886628872 | 6.254537112 | 16.02121673 | 1.65E-52 | 2.28E-51 | 108.2351644 |
| SPINT1 | 1.272631713 | 5.775028339 | 16.02097667 | 1.65E-52 | 2.29E-51 | 108.2319958 |
| MTX1 | 0.677238119 | 2.799234233 | 16.01982616 | 1.68E-52 | 2.32E-51 | 108.2168102 |
| CIT | 0.866388953 | 1.346806253 | 16.01679696 | 1.75E-52 | 2.41E-51 | 108.1768314 |
| UBAP2L | 0.728230754 | 4.96149395 | 16.01548907 | 1.78E-52 | 2.45E-51 | 108.1595717 |
| MRGPRX3 | -0.6762213 | 0.274884484 | -16.01290508 | 1.84E-52 | 2.54E-51 | 108.1254744 |
| TONSL | 1.145987912 | 1.920129267 | 15.99806226 | 2.24E-52 | 3.08E-51 | 107.9296836 |
| NACAD | -0.696455141 | 0.680976433 | -15.99632414 | 2.29E-52 | 3.15E-51 | 107.9067638 |
| PKP3 | 1.29058393 | 4.086449492 | 15.99604625 | 2.30E-52 | 3.16E-51 | 107.9030995 |
| NMB | -1.497959298 | 2.453340309 | -15.99602697 | 2.30E-52 | 3.16E-51 | 107.9028453 |
| MSRA | -0.815035928 | 1.673747805 | -15.99506849 | 2.33E-52 | 3.20E-51 | 107.8902072 |
| GTF3C1 | 0.787884696 | 3.785196597 | 15.99217009 | 2.42E-52 | 3.32E-51 | 107.851993 |
| LIX1L | -0.999795961 | 2.863532859 | -15.98993963 | 2.49E-52 | 3.42E-51 | 107.8225882 |
| TMCO1 | 0.79303549 | 4.839530433 | 15.98783349 | 2.56E-52 | 3.51E-51 | 107.7948249 |
| CDCA4 | 0.957610066 | 3.05546984 | 15.98362897 | 2.71E-52 | 3.71E-51 | 107.7394078 |
| YIF1B | 0.920713009 | 3.514457438 | 15.98343656 | 2.72E-52 | 3.72E-51 | 107.736872 |
| RND3 | -1.615675774 | 3.614004643 | -15.96362587 | 3.53E-52 | 4.83E-51 | 107.475889 |
| PPP1R14B | 1.316488705 | 5.161648776 | 15.95128004 | 4.15E-52 | 5.67E-51 | 107.3133532 |
| SAPCD2 | 1.40762219 | 2.380071002 | 15.94953136 | 4.25E-52 | 5.80E-51 | 107.2903381 |
| TXNDC17 | 0.851795485 | 3.264172325 | 15.94874128 | 4.30E-52 | 5.86E-51 | 107.2799401 |
| UQCC2 | 0.912868138 | 3.03521619 | 15.94640948 | 4.43E-52 | 6.04E-51 | 107.2492538 |
| ADIPOR1 | 0.789171247 | 5.87588538 | 15.94305695 | 4.63E-52 | 6.31E-51 | 107.2051397 |
| SIRPA | -1.259153168 | 3.561966983 | -15.9423575 | 4.67E-52 | 6.36E-51 | 107.1959369 |
| NEIL3 | 0.896441413 | 0.976550538 | 15.93331119 | 5.27E-52 | 7.16E-51 | 107.0769354 |
| CAB39L | -1.238765734 | 2.926977009 | -15.93180634 | 5.37E-52 | 7.30E-51 | 107.0571438 |
| EGR3 | -2.107261517 | 2.388228565 | -15.93160898 | 5.39E-52 | 7.32E-51 | 107.0545482 |
| PHKG2 | 0.80201208 | 2.473114494 | 15.92670422 | 5.75E-52 | 7.80E-51 | 106.9900505 |
| OLR1 | 1.526703488 | 2.581050476 | 15.91725044 | 6.51E-52 | 8.83E-51 | 106.8657695 |
| ZBTB4 | -0.881354615 | 4.360521207 | -15.91509937 | 6.70E-52 | 9.07E-51 | 106.8374979 |
| STX6 | 0.692034283 | 3.583904066 | 15.9148868 | 6.72E-52 | 9.09E-51 | 106.8347042 |
| HAGHL | 1.28224551 | 1.483677733 | 15.90888857 | 7.27E-52 | 9.84E-51 | 106.7558829 |
| KLHDC1 | -0.711856574 | 1.079446056 | -15.90292779 | 7.87E-52 | 1.06E-50 | 106.677573 |
| ALG1 | 0.725586816 | 2.71512496 | 15.89797505 | 8.40E-52 | 1.13E-50 | 106.6125209 |
| KIF15 | 1.116421093 | 1.510396993 | 15.89203811 | 9.08E-52 | 1.23E-50 | 106.5345591 |
| MID1 | -1.21786343 | 1.209498798 | -15.88692107 | 9.72E-52 | 1.31E-50 | 106.4673791 |
| HRASLS5 | -2.058468126 | 1.755401283 | -15.88492115 | 9.98E-52 | 1.34E-50 | 106.4411266 |
| MEST | -1.645247701 | 3.32328874 | -15.8804577 | 1.06E-51 | 1.43E-50 | 106.3825439 |
| IRS2 | -1.510390191 | 2.313259872 | -15.87792037 | 1.09E-51 | 1.47E-50 | 106.3492461 |
| AIMP2 | 0.663728689 | 3.484636833 | 15.87495302 | 1.14E-51 | 1.53E-50 | 106.3103097 |
| SMS | 0.972933913 | 5.663122705 | 15.86698917 | 1.26E-51 | 1.70E-50 | 106.2058344 |
| NMNAT2 | -1.051331065 | 0.596637445 | -15.86525025 | 1.29E-51 | 1.74E-50 | 106.1830266 |
| LIG1 | 0.882097342 | 2.862036634 | 15.86134728 | 1.36E-51 | 1.83E-50 | 106.131841 |
| LRWD1 | 0.74988536 | 2.038083597 | 15.84466998 | 1.70E-51 | 2.27E-50 | 105.9132186 |
| VIM | -1.364485361 | 7.413359276 | -15.84399203 | 1.71E-51 | 2.29E-50 | 105.9043346 |
| RAD51AP1 | 1.316933477 | 2.223615836 | 15.82799662 | 2.11E-51 | 2.83E-50 | 105.6947985 |
| DNASE2 | 0.778271498 | 5.217333165 | 15.81937085 | 2.36E-51 | 3.16E-50 | 105.5818604 |
| FLI1 | -0.910289363 | 1.75570547 | -15.81618692 | 2.47E-51 | 3.30E-50 | 105.5401831 |
| OLFML1 | -1.13891266 | 2.191410587 | -15.81085961 | 2.64E-51 | 3.53E-50 | 105.4704614 |
| TCF7L2 | -1.119227955 | 2.865697856 | -15.80952611 | 2.69E-51 | 3.59E-50 | 105.4530115 |
| SOSTDC1 | -2.006141004 | 1.222031415 | -15.80945625 | 2.69E-51 | 3.59E-50 | 105.4520973 |
| TMOD2 | -0.829014505 | 1.2671329 | -15.80643754 | 2.80E-51 | 3.74E-50 | 105.4125988 |
| PDCL3 | 0.692007158 | 3.932856653 | 15.7992826 | 3.08E-51 | 4.10E-50 | 105.3189994 |
| HSPE1 | 0.982196308 | 5.410827135 | 15.79655376 | 3.19E-51 | 4.25E-50 | 105.2833085 |
| CLEC14A | -1.217541364 | 3.214957697 | -15.78445016 | 3.74E-51 | 4.97E-50 | 105.1250528 |
| TLN1 | -0.780667778 | 5.307582341 | -15.78347457 | 3.79E-51 | 5.03E-50 | 105.1123003 |
| ST3GAL3 | -0.665883567 | 1.758739272 | -15.78338498 | 3.79E-51 | 5.04E-50 | 105.1111293 |
| IL17RD | -1.344953999 | 2.029190068 | -15.77878656 | 4.03E-51 | 5.35E-50 | 105.051028 |
| LRP1 | -1.514277552 | 4.215851766 | -15.77819872 | 4.06E-51 | 5.38E-50 | 105.0433458 |
| CSTF2 | 0.69888594 | 3.316534029 | 15.77265309 | 4.37E-51 | 5.78E-50 | 104.9708816 |
| SASH1 | -1.353355148 | 2.883958255 | -15.76706247 | 4.70E-51 | 6.21E-50 | 104.8978465 |
| RBFOX2 | -0.774268458 | 3.370991503 | -15.76686892 | 4.71E-51 | 6.23E-50 | 104.8953183 |
| HSPA12B | -1.136207036 | 2.02673962 | -15.76564462 | 4.79E-51 | 6.32E-50 | 104.8793266 |
| SEMA6A | -1.201214838 | 1.436010143 | -15.76297192 | 4.96E-51 | 6.54E-50 | 104.8444188 |
| SOX5 | -0.688248248 | 0.479268981 | -15.76184763 | 5.03E-51 | 6.63E-50 | 104.8297359 |
| CENPE | 1.086750197 | 1.436952938 | 15.75451266 | 5.54E-51 | 7.29E-50 | 104.7339594 |
| SACS | -0.824110426 | 1.191297657 | -15.75425017 | 5.56E-51 | 7.31E-50 | 104.7305325 |
| KIRREL1 | -1.285699223 | 2.844109125 | -15.74330575 | 6.42E-51 | 8.43E-50 | 104.5876817 |
| SEC14L1 | -0.732944765 | 2.977405747 | -15.74019737 | 6.68E-51 | 8.78E-50 | 104.5471219 |
| PRSS8 | 1.722939646 | 5.800628974 | 15.73607152 | 7.06E-51 | 9.26E-50 | 104.4932937 |
| ARHGEF6 | -1.430419703 | 3.194048203 | -15.73387458 | 7.26E-51 | 9.52E-50 | 104.4646349 |
| DHDDS | -0.609706942 | 3.092746574 | -15.71804526 | 8.93E-51 | 1.17E-49 | 104.2582215 |
| KCTD14 | -1.456629701 | 1.171352289 | -15.71493994 | 9.31E-51 | 1.22E-49 | 104.2177445 |
| TMEM237 | -0.734276435 | 2.177390013 | -15.69934011 | 1.14E-50 | 1.49E-49 | 104.0144842 |
| ACTA2 | -1.729887348 | 6.444543868 | -15.69908003 | 1.15E-50 | 1.50E-49 | 104.0110966 |
| CILP2 | 1.508017552 | 1.816303486 | 15.69818132 | 1.16E-50 | 1.51E-49 | 103.9993909 |
| CCDC8 | -1.64876684 | 2.34361261 | -15.69755815 | 1.17E-50 | 1.53E-49 | 103.9912742 |
| RTKN | 0.908383608 | 3.378604189 | 15.69722172 | 1.17E-50 | 1.53E-49 | 103.9868925 |
| MYCBP2 | -1.056557953 | 2.595043327 | -15.69266479 | 1.25E-50 | 1.62E-49 | 103.9275477 |
| STXBP1 | -1.064879574 | 2.343645564 | -15.68262379 | 1.42E-50 | 1.85E-49 | 103.7968242 |
| SHMT1 | -0.834401606 | 2.440096474 | -15.68215129 | 1.43E-50 | 1.86E-49 | 103.7906741 |
| CDC6 | 1.564228509 | 2.205591553 | 15.6811885 | 1.45E-50 | 1.88E-49 | 103.7781426 |
| PIGU | 0.755375715 | 3.641966213 | 15.66562857 | 1.77E-50 | 2.30E-49 | 103.5756896 |
| OLFML2A | -1.095000676 | 2.903966119 | -15.6655491 | 1.78E-50 | 2.31E-49 | 103.574656 |
| PGAM5 | 0.775060856 | 3.775625842 | 15.65553034 | 2.02E-50 | 2.63E-49 | 103.4443707 |
| TTK | 1.35966133 | 1.822256523 | 15.649998 | 2.17E-50 | 2.82E-49 | 103.3724511 |
| ZFAND5 | -0.727755168 | 4.606448764 | -15.64036409 | 2.47E-50 | 3.20E-49 | 103.2472519 |
| TTC23 | -0.799858277 | 1.744013383 | -15.63459423 | 2.66E-50 | 3.44E-49 | 103.172293 |
| KPNA5 | -0.712448625 | 1.550686769 | -15.63450969 | 2.66E-50 | 3.45E-49 | 103.1711948 |
| CFI | -1.312091697 | 2.31345822 | -15.63388831 | 2.68E-50 | 3.47E-49 | 103.1631233 |
| PRXL2C | -0.728095859 | 2.45628594 | -15.6311123 | 2.78E-50 | 3.60E-49 | 103.1270665 |
| ANGPTL4 | -2.045056369 | 2.551836666 | -15.62881229 | 2.87E-50 | 3.70E-49 | 103.0971956 |
| OCIAD2 | 0.951451993 | 4.054101705 | 15.62804909 | 2.90E-50 | 3.74E-49 | 103.0872842 |
| ARHGAP31 | -0.960599391 | 2.286026775 | -15.62800617 | 2.90E-50 | 3.74E-49 | 103.0867269 |
| RAD54L | 1.146887257 | 1.472683449 | 15.62615625 | 2.97E-50 | 3.83E-49 | 103.0627043 |
| SUCLA2 | -0.76456541 | 3.578123244 | -15.62036693 | 3.20E-50 | 4.12E-49 | 102.9875379 |
| PFDN6 | 0.946330657 | 3.579813866 | 15.61965201 | 3.23E-50 | 4.16E-49 | 102.9782569 |
| ADAM8 | 1.358109567 | 2.219384001 | 15.61681089 | 3.35E-50 | 4.31E-49 | 102.9413766 |
| TIE1 | -1.0128838 | 2.278406526 | -15.61659121 | 3.36E-50 | 4.32E-49 | 102.9385252 |
| ZDHHC12 | 0.987218389 | 3.726911994 | 15.61603925 | 3.39E-50 | 4.35E-49 | 102.9313609 |
| POLE2 | 0.785966855 | 1.568338729 | 15.60951731 | 3.69E-50 | 4.73E-49 | 102.8467201 |
| ABHD12 | 0.942924471 | 4.58144918 | 15.60660122 | 3.83E-50 | 4.91E-49 | 102.808883 |
| CACNB3 | 1.019694515 | 2.683505003 | 15.59465472 | 4.48E-50 | 5.73E-49 | 102.653923 |
| TJP3 | 1.519547434 | 3.578649811 | 15.59300542 | 4.57E-50 | 5.85E-49 | 102.6325358 |
| MYL9 | -1.55284908 | 6.241799695 | -15.59219278 | 4.62E-50 | 5.91E-49 | 102.6219985 |
| HOXA9 | -1.23158293 | 0.859100451 | -15.58907734 | 4.81E-50 | 6.15E-49 | 102.5816049 |
| MRPL47 | 0.802001511 | 4.827245784 | 15.58737756 | 4.92E-50 | 6.28E-49 | 102.5595683 |
| KRT18 | 1.89595083 | 7.492963579 | 15.58529153 | 5.06E-50 | 6.45E-49 | 102.5325265 |
| CSN1S1 | -1.511012973 | 0.325989236 | -15.58306553 | 5.20E-50 | 6.63E-49 | 102.5036728 |
| ACVR2A | -0.712289226 | 1.73961646 | -15.58298671 | 5.21E-50 | 6.63E-49 | 102.5026512 |
| R3HDM4 | 0.799150378 | 4.764398414 | 15.58114461 | 5.34E-50 | 6.79E-49 | 102.478776 |
| COPE | 0.969794127 | 5.279536495 | 15.57922407 | 5.47E-50 | 6.96E-49 | 102.4538859 |
| TUBG1 | 0.951157931 | 4.218768455 | 15.57498363 | 5.78E-50 | 7.35E-49 | 102.3989375 |
| PIMREG | 1.261810085 | 1.548868594 | 15.56071726 | 6.96E-50 | 8.84E-49 | 102.2141442 |
| SPATS2 | 0.653185004 | 2.700054189 | 15.55746964 | 7.26E-50 | 9.22E-49 | 102.1720933 |
| RPN1 | 0.684986838 | 6.489286406 | 15.55181205 | 7.82E-50 | 9.91E-49 | 102.0988514 |
| APEX2 | 0.72306349 | 3.764817962 | 15.54694243 | 8.33E-50 | 1.05E-48 | 102.0358246 |
| CD276 | 0.827241052 | 4.48706821 | 15.54449511 | 8.60E-50 | 1.09E-48 | 102.0041542 |
| SLC16A3 | 1.377031715 | 2.21422067 | 15.54423647 | 8.63E-50 | 1.09E-48 | 102.0008074 |
| ZWILCH | 0.778950541 | 2.36952725 | 15.54057126 | 9.05E-50 | 1.14E-48 | 101.9533832 |
| VKORC1L1 | -0.765916906 | 3.925452459 | -15.53743692 | 9.42E-50 | 1.19E-48 | 101.9128339 |
| DPY30 | 0.703686977 | 3.922845795 | 15.52678885 | 1.08E-49 | 1.37E-48 | 101.7751193 |
| IFNGR1 | -0.936236777 | 5.070796241 | -15.51339205 | 1.29E-49 | 1.62E-48 | 101.6019439 |
| C3orf80 | 0.858691507 | 1.309697747 | 15.51307789 | 1.29E-49 | 1.63E-48 | 101.5978839 |
| IRF7 | 1.493339084 | 3.752001005 | 15.51131016 | 1.32E-49 | 1.67E-48 | 101.5750409 |
| ECHDC1 | -1.113961398 | 2.860839064 | -15.50556327 | 1.43E-49 | 1.79E-48 | 101.5007901 |
| PLD1 | -0.829358363 | 1.171612506 | -15.50027395 | 1.53E-49 | 1.92E-48 | 101.4324675 |
| SDC1 | 1.914005375 | 6.257942113 | 15.49123389 | 1.72E-49 | 2.15E-48 | 101.3157321 |
| PCYOX1 | -0.980312274 | 4.641477408 | -15.49065442 | 1.73E-49 | 2.17E-48 | 101.3082509 |
| KANK2 | -0.963156321 | 3.403949513 | -15.48202438 | 1.93E-49 | 2.42E-48 | 101.1968554 |
| BUB3 | 0.861630934 | 4.130633139 | 15.47584842 | 2.10E-49 | 2.62E-48 | 101.1171621 |
| CTIF | -0.9097471 | 2.659052443 | -15.47019435 | 2.25E-49 | 2.82E-48 | 101.0442219 |
| CTNNAL1 | -1.00428632 | 2.977928219 | -15.46769538 | 2.33E-49 | 2.91E-48 | 101.0119898 |
| PLAUR | 1.291441283 | 2.96214972 | 15.45709116 | 2.67E-49 | 3.34E-48 | 100.8752531 |
| MRC1 | -1.492226405 | 1.54717816 | -15.45493397 | 2.75E-49 | 3.43E-48 | 100.8474447 |
| LAMP5 | 1.805064576 | 2.529233028 | 15.45392085 | 2.78E-49 | 3.48E-48 | 100.8343854 |
| TSPAN17 | 0.741053698 | 3.282049382 | 15.44933449 | 2.95E-49 | 3.69E-48 | 100.7752738 |
| CERS2 | 1.131671262 | 5.985337373 | 15.44756584 | 3.02E-49 | 3.77E-48 | 100.7524816 |
| CHST3 | -1.333990581 | 2.147337355 | -15.44502963 | 3.12E-49 | 3.89E-48 | 100.719801 |
| U2AF2 | 0.687394378 | 5.285964844 | 15.43919012 | 3.37E-49 | 4.20E-48 | 100.6445692 |
| ATAD2 | 1.443458195 | 3.360068488 | 15.43471548 | 3.57E-49 | 4.44E-48 | 100.5869341 |
| KIF26B | 1.177026407 | 1.525229861 | 15.42979738 | 3.80E-49 | 4.73E-48 | 100.5236002 |
| CKAP4 | 0.897791623 | 5.328344331 | 15.3990974 | 5.66E-49 | 7.03E-48 | 100.1285589 |
| EPHA2 | -1.078249779 | 2.257984685 | -15.39013189 | 6.35E-49 | 7.88E-48 | 100.013292 |
| MDFIC | -1.028330944 | 2.909671228 | -15.38893211 | 6.45E-49 | 8.00E-48 | 99.99787021 |
| CKAP2 | 1.048100338 | 3.02846254 | 15.38879344 | 6.46E-49 | 8.01E-48 | 99.99608786 |
| APCDD1L | -1.389279292 | 0.96850084 | -15.38809835 | 6.52E-49 | 8.07E-48 | 99.98715364 |
| AP2S1 | 0.886525392 | 5.482741811 | 15.38748908 | 6.57E-49 | 8.13E-48 | 99.97932276 |
| TOR2A | 0.812265458 | 2.397909773 | 15.38343789 | 6.92E-49 | 8.56E-48 | 99.92725868 |
| PYROXD2 | -1.027188769 | 1.814358029 | -15.37718373 | 7.51E-49 | 9.28E-48 | 99.84690084 |
| CENPL | 0.737830525 | 1.628489768 | 15.36409817 | 8.89E-49 | 1.10E-47 | 99.67883951 |
| PFN1 | 0.846967014 | 7.933245209 | 15.36121879 | 9.22E-49 | 1.14E-47 | 99.6418717 |
| STON1 | -0.782118593 | 1.183724463 | -15.35013519 | 1.06E-48 | 1.31E-47 | 99.49961513 |
| MBNL2 | -1.205107923 | 3.897852916 | -15.34494041 | 1.14E-48 | 1.40E-47 | 99.43296454 |
| DEPDC1 | 1.216952485 | 1.425836451 | 15.34219746 | 1.18E-48 | 1.45E-47 | 99.39777788 |
| ADAMTS14 | 0.918982378 | 1.029648209 | 15.34117454 | 1.19E-48 | 1.47E-47 | 99.38465682 |
| STIP1 | 0.878437743 | 5.272071215 | 15.33786623 | 1.25E-48 | 1.53E-47 | 99.34222518 |
| ASNA1 | 0.702136368 | 5.503690126 | 15.33268022 | 1.33E-48 | 1.64E-47 | 99.2757228 |
| ROCK2 | -0.898661702 | 2.887102779 | -15.33007013 | 1.38E-48 | 1.69E-47 | 99.24225831 |
| MYDGF | 0.934866461 | 5.542009542 | 15.32583264 | 1.45E-48 | 1.78E-47 | 99.18793675 |
| NEDD9 | -1.322270316 | 2.673742207 | -15.31780328 | 1.61E-48 | 1.98E-47 | 99.08503383 |
| CENPX | 1.237490335 | 4.564205582 | 15.31554809 | 1.66E-48 | 2.03E-47 | 99.0561382 |
| CD3EAP | 0.618891807 | 1.618617877 | 15.31508751 | 1.67E-48 | 2.04E-47 | 99.05023719 |
| H3F3A | 0.828724775 | 4.123660243 | 15.30127255 | 2.00E-48 | 2.44E-47 | 98.87329303 |
| ACSL4 | -1.178120749 | 2.704022713 | -15.29123593 | 2.27E-48 | 2.78E-47 | 98.74480998 |
| SLC39A7 | 0.906911706 | 6.128224259 | 15.28987706 | 2.31E-48 | 2.82E-47 | 98.72741887 |
| KATNAL1 | -0.686309346 | 1.704350451 | -15.28258869 | 2.54E-48 | 3.10E-47 | 98.63415861 |
| DUSP22 | -0.691564506 | 2.749663418 | -15.28199209 | 2.56E-48 | 3.12E-47 | 98.62652591 |
| RIC3 | -0.890315325 | 0.588634756 | -15.2812793 | 2.58E-48 | 3.15E-47 | 98.61740716 |
| RCC1 | 0.982001238 | 3.81512282 | 15.27880814 | 2.66E-48 | 3.25E-47 | 98.58579533 |
| ANKRD35 | -1.033412459 | 0.963942481 | -15.27467523 | 2.81E-48 | 3.42E-47 | 98.53293375 |
| FAM69A | -0.925490341 | 2.493560091 | -15.27306823 | 2.87E-48 | 3.49E-47 | 98.51238215 |
| PARN | 0.631348636 | 4.297411631 | 15.2705491 | 2.96E-48 | 3.60E-47 | 98.48016839 |
| SPART | -1.016350414 | 3.018459973 | -15.26619953 | 3.13E-48 | 3.81E-47 | 98.42455621 |
| SNRK | -0.739628995 | 2.929044151 | -15.26598865 | 3.14E-48 | 3.82E-47 | 98.42186024 |
| C22orf39 | -0.591338236 | 2.120659108 | -15.25975246 | 3.40E-48 | 4.13E-47 | 98.34214564 |
| ZMYND19 | 0.838855447 | 3.260863105 | 15.25937005 | 3.42E-48 | 4.15E-47 | 98.33725822 |
| LPAR2 | 0.940592245 | 2.922765368 | 15.25713382 | 3.52E-48 | 4.27E-47 | 98.30867923 |
| EIF4E3 | -0.858438896 | 2.199593322 | -15.25491008 | 3.62E-48 | 4.39E-47 | 98.28026278 |
| CC2D2A | -0.670953701 | 1.505577913 | -15.24794113 | 3.96E-48 | 4.80E-47 | 98.19122682 |
| ZNF25 | -0.687864127 | 2.272950372 | -15.24557058 | 4.08E-48 | 4.94E-47 | 98.16094664 |
| YKT6 | 0.645407689 | 4.946843883 | 15.23661271 | 4.58E-48 | 5.53E-47 | 98.04655234 |
| SCN7A | -0.661789842 | 0.421502619 | -15.22405093 | 5.38E-48 | 6.50E-47 | 97.88621195 |
| KRT19 | 2.248684593 | 8.815240875 | 15.22259826 | 5.48E-48 | 6.62E-47 | 97.86767562 |
| COMMD5 | 0.846709398 | 3.522785639 | 15.22028616 | 5.65E-48 | 6.81E-47 | 97.83817524 |
| MRPS12 | 1.008787473 | 3.759339835 | 15.2196732 | 5.69E-48 | 6.86E-47 | 97.8303549 |
| EPN3 | 1.54690191 | 3.015765098 | 15.21259932 | 6.23E-48 | 7.49E-47 | 97.74011976 |
| C4BPA | -1.052554219 | 0.407371683 | -15.20858288 | 6.56E-48 | 7.88E-47 | 97.68889834 |
| PPL | -1.307099711 | 3.278809223 | -15.20483263 | 6.88E-48 | 8.26E-47 | 97.64107985 |
| POP7 | 0.923331967 | 4.764515415 | 15.20323103 | 7.02E-48 | 8.43E-47 | 97.62066061 |
| MRVI1 | -0.920970529 | 1.772897224 | -15.20048842 | 7.28E-48 | 8.72E-47 | 97.5856979 |
| MARS | 0.61197149 | 3.877154271 | 15.19783855 | 7.53E-48 | 9.02E-47 | 97.55192134 |
| PDGFA | -1.004434503 | 2.38851246 | -15.19115104 | 8.20E-48 | 9.82E-47 | 97.46669701 |
| PTGS2 | -1.351823898 | 0.794157129 | -15.18702274 | 8.64E-48 | 1.03E-46 | 97.41409936 |
| LYPLA2 | 0.826087789 | 4.878833214 | 15.18659613 | 8.69E-48 | 1.04E-46 | 97.40866458 |
| MISP | 2.239388987 | 3.173978048 | 15.18626185 | 8.73E-48 | 1.04E-46 | 97.4044061 |
| PDE1A | -0.592205267 | 0.760851098 | -15.18444674 | 8.93E-48 | 1.07E-46 | 97.38128411 |
| UACA | -0.792657615 | 2.94642633 | -15.18435976 | 8.94E-48 | 1.07E-46 | 97.38017623 |
| FAM43A | -0.931831349 | 2.01807675 | -15.17361951 | 1.03E-47 | 1.22E-46 | 97.2433991 |
| RHNO1 | 0.826230435 | 3.769691742 | 15.16384531 | 1.16E-47 | 1.39E-46 | 97.11898182 |
| A2M | -1.396325346 | 6.618462667 | -15.1501121 | 1.39E-47 | 1.65E-46 | 96.94426158 |
| LRRC59 | 1.068157935 | 5.275337016 | 15.14945402 | 1.40E-47 | 1.66E-46 | 96.93589185 |
| HPRT1 | 0.949594281 | 4.504191685 | 15.14669921 | 1.45E-47 | 1.72E-46 | 96.90085785 |
| SEMA5A | -1.310482212 | 2.03036004 | -15.14551607 | 1.47E-47 | 1.75E-46 | 96.88581271 |
| GIMAP7 | -1.496923925 | 3.216607589 | -15.13389184 | 1.71E-47 | 2.02E-46 | 96.7380384 |
| ABCB8 | 0.693148248 | 2.296675531 | 15.13257302 | 1.73E-47 | 2.06E-46 | 96.72127757 |
| BAK1 | 0.83388529 | 3.442785143 | 15.13051048 | 1.78E-47 | 2.11E-46 | 96.69506694 |
| RGS19 | 0.870567809 | 3.238389629 | 15.12931861 | 1.81E-47 | 2.14E-46 | 96.67992178 |
| BST1 | -0.752325797 | 1.431482953 | -15.120247 | 2.03E-47 | 2.40E-46 | 96.5646752 |
| SRSF9 | 0.637637656 | 4.407556204 | 15.1177272 | 2.10E-47 | 2.48E-46 | 96.53267176 |
| KIF14 | 1.086932655 | 1.193689704 | 15.11457874 | 2.18E-47 | 2.58E-46 | 96.49268898 |
| DNAJA3 | 0.670946186 | 3.510634243 | 15.1128514 | 2.23E-47 | 2.64E-46 | 96.47075562 |
| MAGI1 | -0.874581738 | 2.238817755 | -15.11069887 | 2.29E-47 | 2.71E-46 | 96.44342572 |
| ERH | 0.730341983 | 6.197839743 | 15.1081283 | 2.37E-47 | 2.80E-46 | 96.4107915 |
| DPP4 | -1.200527156 | 1.555686259 | -15.10620477 | 2.43E-47 | 2.86E-46 | 96.38637412 |
| MFSD12 | 0.835500794 | 3.318941538 | 15.10139162 | 2.58E-47 | 3.04E-46 | 96.32528515 |
| RUVBL2 | 0.887863118 | 4.587644074 | 15.10125739 | 2.59E-47 | 3.05E-46 | 96.32358169 |
| CBX8 | 0.861625728 | 1.846870293 | 15.10072538 | 2.60E-47 | 3.06E-46 | 96.31683028 |
| MECOM | -0.81325481 | 1.245069684 | -15.09778637 | 2.70E-47 | 3.18E-46 | 96.27953572 |
| MGRN1 | 0.703555087 | 3.329001126 | 15.09620461 | 2.76E-47 | 3.24E-46 | 96.25946605 |
| EMILIN2 | -1.023279291 | 1.77031834 | -15.0944232 | 2.82E-47 | 3.32E-46 | 96.23686484 |
| ARPC3 | 0.663533279 | 6.036796064 | 15.09314908 | 2.87E-47 | 3.37E-46 | 96.22070086 |
| MXRA7 | -0.986027325 | 2.881890333 | -15.08933788 | 3.01E-47 | 3.53E-46 | 96.17235616 |
| IER5L | 1.2298213 | 2.667980152 | 15.07912247 | 3.43E-47 | 4.02E-46 | 96.04281555 |
| BCL9 | 0.972009556 | 3.359656553 | 15.07819315 | 3.47E-47 | 4.07E-46 | 96.03103396 |
| CYB5R3 | -0.782085517 | 5.006410428 | -15.07633402 | 3.55E-47 | 4.16E-46 | 96.00746591 |
| FILIP1 | -0.621122534 | 0.753995718 | -15.0746504 | 3.63E-47 | 4.25E-46 | 95.98612459 |
| SEPHS2 | 1.030340885 | 5.67752052 | 15.06264 | 4.23E-47 | 4.95E-46 | 95.83392919 |
| STAC2 | -3.052947015 | 2.219028798 | -15.06178502 | 4.27E-47 | 5.00E-46 | 95.82309816 |
| ILF2 | 0.861076314 | 6.774239556 | 15.05917356 | 4.42E-47 | 5.17E-46 | 95.79001803 |
| KIF24 | 0.714277264 | 1.315736034 | 15.03911436 | 5.70E-47 | 6.66E-46 | 95.53605314 |
| BRINP1 | -1.156030818 | 0.663380265 | -15.03766109 | 5.81E-47 | 6.78E-46 | 95.51766268 |
| ACBD6 | 0.606413721 | 2.789228391 | 15.03402013 | 6.09E-47 | 7.10E-46 | 95.47159321 |
| CLDN7 | 1.400498803 | 5.192263714 | 15.03394876 | 6.09E-47 | 7.10E-46 | 95.47069017 |
| CYBC1 | 0.769052533 | 3.420852358 | 15.02901254 | 6.49E-47 | 7.55E-46 | 95.4082439 |
| STMN1 | 1.394219696 | 4.728780602 | 15.02154916 | 7.13E-47 | 8.29E-46 | 95.31385426 |
| PPP6R1 | 0.707122671 | 3.854107044 | 15.01725213 | 7.53E-47 | 8.75E-46 | 95.25952409 |
| CYTL1 | -0.940308519 | 1.023982224 | -15.00530601 | 8.76E-47 | 1.02E-45 | 95.10853722 |
| JUN | -1.520047787 | 5.756489912 | -15.00515494 | 8.78E-47 | 1.02E-45 | 95.10662835 |
| ACVRL1 | -0.876235666 | 2.497348169 | -15.00029343 | 9.34E-47 | 1.08E-45 | 95.04520771 |
| RASSF7 | 1.380846476 | 3.423790684 | 14.98600886 | 1.12E-46 | 1.30E-45 | 94.86481452 |
| COL4A3BP | -0.794382679 | 2.684077913 | -14.98551968 | 1.13E-46 | 1.30E-45 | 94.85863898 |
| ADAMTS9 | -0.957911047 | 1.355602239 | -14.98429586 | 1.14E-46 | 1.32E-45 | 94.84318975 |
| ISG15 | 2.538038656 | 5.69211096 | 14.98351606 | 1.16E-46 | 1.34E-45 | 94.83334612 |
| AMY2B | -0.642433313 | 0.70458252 | -14.96773113 | 1.41E-46 | 1.63E-45 | 94.63416524 |
| DONSON | 0.91648038 | 2.431871383 | 14.96678922 | 1.43E-46 | 1.65E-45 | 94.62228439 |
| FIBP | 0.695679647 | 4.171912123 | 14.96529458 | 1.46E-46 | 1.68E-45 | 94.60343268 |
| HNRNPF | 0.687958141 | 6.559046578 | 14.95951938 | 1.57E-46 | 1.81E-45 | 94.53060275 |
| FIGN | -0.711014442 | 0.517952545 | -14.95924018 | 1.57E-46 | 1.81E-45 | 94.52708236 |
| ARFGAP1 | 0.915167113 | 3.797681443 | 14.957519 | 1.61E-46 | 1.85E-45 | 94.50538098 |
| SMIM3 | -1.092174053 | 3.327087679 | -14.94859045 | 1.80E-46 | 2.07E-45 | 94.39283323 |
| FAM124A | -0.587323236 | 0.60733856 | -14.94312114 | 1.93E-46 | 2.22E-45 | 94.32391328 |
| SH3RF2 | -0.868665355 | 0.864631122 | -14.94252211 | 1.94E-46 | 2.23E-45 | 94.31636572 |
| ZFP36L2 | -1.325737926 | 5.7872729 | -14.93969673 | 2.01E-46 | 2.31E-45 | 94.28077027 |
| FANCF | 0.744681207 | 2.874490446 | 14.93896898 | 2.03E-46 | 2.33E-45 | 94.27160251 |
| MYEOV | -1.546454464 | 0.901400964 | -14.93022152 | 2.27E-46 | 2.60E-45 | 94.16143099 |
| GPAT3 | -1.00192172 | 1.140187124 | -14.9205965 | 2.56E-46 | 2.94E-45 | 94.04025795 |
| RNPS1 | 0.646066709 | 4.039026633 | 14.91574048 | 2.73E-46 | 3.12E-45 | 93.97914409 |
| MIS18A | 0.850715407 | 3.342798461 | 14.91329797 | 2.81E-46 | 3.22E-45 | 93.94840993 |
| AKAP7 | -0.628339429 | 1.369267918 | -14.91273622 | 2.83E-46 | 3.24E-45 | 93.94134185 |
| TBCB | 0.885121381 | 4.244695017 | 14.90862965 | 2.98E-46 | 3.41E-45 | 93.88967779 |
| REV3L | -0.824208502 | 1.798738338 | -14.8954962 | 3.52E-46 | 4.02E-45 | 93.72451375 |
| CYB561 | 1.135061261 | 5.073958008 | 14.88327729 | 4.11E-46 | 4.69E-45 | 93.57094078 |
| KLF8 | -0.807535752 | 1.244575025 | -14.88322382 | 4.11E-46 | 4.69E-45 | 93.57026893 |
| SDF2L1 | 1.255387079 | 3.957382608 | 14.87797528 | 4.39E-46 | 5.01E-45 | 93.50432971 |
| PFKFB4 | 0.693113442 | 1.554083506 | 14.8653298 | 5.15E-46 | 5.87E-45 | 93.34552581 |
| KIF18A | 0.965878224 | 1.336740762 | 14.86078662 | 5.46E-46 | 6.21E-45 | 93.28849451 |
| PRX | -0.79675407 | 1.18286549 | -14.84936808 | 6.30E-46 | 7.17E-45 | 93.14520893 |
| CDC42EP2 | -0.887574782 | 2.746688891 | -14.8464517 | 6.54E-46 | 7.43E-45 | 93.10862498 |
| VPS72 | 0.762665644 | 3.719086263 | 14.84607807 | 6.57E-46 | 7.46E-45 | 93.10393831 |
| GFER | 0.919804611 | 3.099072059 | 14.83967718 | 7.12E-46 | 8.09E-45 | 93.02366223 |
| SAC3D1 | 1.069465616 | 2.899312475 | 14.83749385 | 7.32E-46 | 8.31E-45 | 92.99628577 |
| ORMDL2 | 0.731791317 | 3.58808815 | 14.83681226 | 7.38E-46 | 8.37E-45 | 92.98773997 |
| ARRDC4 | -1.050284581 | 2.793022036 | -14.8313737 | 7.91E-46 | 8.96E-45 | 92.9195607 |
| ESCO2 | 0.716560317 | 0.856318935 | 14.82399542 | 8.67E-46 | 9.83E-45 | 92.82709216 |
| LAMA4 | -1.158749958 | 3.272870837 | -14.81229613 | 1.01E-45 | 1.14E-44 | 92.68053554 |
| PRR36 | 1.526859576 | 2.849800237 | 14.81122449 | 1.02E-45 | 1.15E-44 | 92.6671152 |
| TMEM54 | 1.113712933 | 4.994411972 | 14.80828942 | 1.06E-45 | 1.19E-44 | 92.6303621 |
| PPM1G | 0.704252251 | 5.085087694 | 14.80271726 | 1.13E-45 | 1.28E-44 | 92.56060102 |
| SNRPG | 0.803866674 | 4.403172277 | 14.80219979 | 1.14E-45 | 1.29E-44 | 92.55412345 |
| ZC3H3 | 0.825476435 | 3.316546476 | 14.79233191 | 1.29E-45 | 1.46E-44 | 92.43062934 |
| PPP1R37 | 0.939616002 | 3.055384158 | 14.78581478 | 1.40E-45 | 1.58E-44 | 92.34910032 |
| SLC39A1 | 0.712426129 | 5.68163804 | 14.78298354 | 1.45E-45 | 1.64E-44 | 92.31368934 |
| MTFR2 | 0.962410018 | 1.194134419 | 14.78295299 | 1.45E-45 | 1.64E-44 | 92.3133073 |
| VAMP2 | -0.951373074 | 4.452580103 | -14.7825 | 1.46E-45 | 1.64E-44 | 92.30764198 |
| SLC12A6 | -0.701141704 | 1.719341675 | -14.77963079 | 1.52E-45 | 1.70E-44 | 92.27176178 |
| PRICKLE2 | -0.96489738 | 1.899173693 | -14.77301165 | 1.65E-45 | 1.85E-44 | 92.18900605 |
| TMEM79 | 1.096236119 | 3.083968597 | 14.76289315 | 1.87E-45 | 2.10E-44 | 92.06254919 |
| MND1 | 1.164281767 | 1.563944751 | 14.75936301 | 1.96E-45 | 2.19E-44 | 92.01844503 |
| STIL | 0.989121003 | 1.587582884 | 14.75773511 | 2.00E-45 | 2.23E-44 | 91.99810925 |
| SIDT2 | -0.759584491 | 2.983742002 | -14.7576153 | 2.00E-45 | 2.24E-44 | 91.99661254 |
| CACYBP | 0.793159773 | 4.314689731 | 14.75330976 | 2.11E-45 | 2.36E-44 | 91.94283529 |
| GMPPA | 0.667417819 | 3.266071715 | 14.74605841 | 2.31E-45 | 2.58E-44 | 91.85228855 |
| TYMP | 1.547553125 | 4.548536244 | 14.74565965 | 2.32E-45 | 2.59E-44 | 91.84731019 |
| SMIM29 | 0.933409693 | 3.589204772 | 14.73581429 | 2.63E-45 | 2.93E-44 | 91.72442408 |
| CHPF2 | 0.730308812 | 4.172330596 | 14.7314878 | 2.77E-45 | 3.09E-44 | 91.67044048 |
| HIST1H2BK | 1.716583803 | 5.937002257 | 14.72462209 | 3.02E-45 | 3.37E-44 | 91.58479647 |
| PDIA3 | 0.781620139 | 7.134290127 | 14.72400992 | 3.05E-45 | 3.39E-44 | 91.57716161 |
| MAPKAPK2 | 0.946543179 | 5.818135481 | 14.72309845 | 3.08E-45 | 3.43E-44 | 91.56579419 |
| MGAT3 | -1.024087477 | 0.965375746 | -14.72075213 | 3.17E-45 | 3.53E-44 | 91.53653421 |
| CCL24 | -0.705853864 | 0.28641731 | -14.71943331 | 3.23E-45 | 3.58E-44 | 91.52008918 |
| ADM2 | 1.165155171 | 1.915714576 | 14.71741732 | 3.31E-45 | 3.67E-44 | 91.49495281 |
| ESAM | -1.014853697 | 3.476333368 | -14.71098858 | 3.59E-45 | 3.98E-44 | 91.41481206 |
| TTYH3 | 1.096480956 | 4.009062899 | 14.70661704 | 3.79E-45 | 4.20E-44 | 91.36033019 |
| LSM14B | 0.740248924 | 4.181053055 | 14.7031171 | 3.96E-45 | 4.38E-44 | 91.31671908 |
| ZNF687 | 0.767150789 | 3.424507252 | 14.70246476 | 3.99E-45 | 4.42E-44 | 91.30859137 |
| ARHGAP26 | -0.893659528 | 1.406229838 | -14.69951057 | 4.14E-45 | 4.58E-44 | 91.27178742 |
| PDXDC1 | 0.821600712 | 4.63380792 | 14.69402045 | 4.43E-45 | 4.90E-44 | 91.20340378 |
| EPS8 | -1.079563927 | 3.287060983 | -14.68455063 | 4.99E-45 | 5.51E-44 | 91.08549183 |
| TRAF7 | 0.722470975 | 4.602764917 | 14.681197 | 5.21E-45 | 5.74E-44 | 91.04374735 |
| AVPI1 | -0.986414684 | 2.886531763 | -14.67239488 | 5.81E-45 | 6.41E-44 | 90.93421386 |
| ESD | -0.6570922 | 4.580285916 | -14.67010924 | 5.98E-45 | 6.58E-44 | 90.90577889 |
| SAE1 | 0.647248987 | 4.978488964 | 14.66760032 | 6.17E-45 | 6.79E-44 | 90.87456973 |
| VSTM4 | -0.958890948 | 1.652873865 | -14.66626775 | 6.27E-45 | 6.90E-44 | 90.85799508 |
| MMP13 | 2.344248909 | 2.290231889 | 14.66528292 | 6.35E-45 | 6.98E-44 | 90.84574622 |
| ZNF727 | -0.678194697 | 0.497177879 | -14.65960177 | 6.82E-45 | 7.49E-44 | 90.77509832 |
| THOC6 | 0.9309644 | 4.053919739 | 14.65865345 | 6.90E-45 | 7.58E-44 | 90.76330734 |
| RPS6KA3 | -1.046120149 | 2.41568501 | -14.65711298 | 7.04E-45 | 7.72E-44 | 90.74415495 |
| WLS | -1.622004141 | 3.372468924 | -14.65551732 | 7.18E-45 | 7.87E-44 | 90.72431788 |
| MRGBP | 0.794343835 | 3.103769897 | 14.65293661 | 7.41E-45 | 8.12E-44 | 90.69223794 |
| F12 | 1.275403151 | 1.698449312 | 14.65274296 | 7.43E-45 | 8.13E-44 | 90.68983086 |
| RASL12 | -1.06959634 | 2.005124161 | -14.6525621 | 7.45E-45 | 8.15E-44 | 90.68758281 |
| FANCD2 | 0.839704613 | 1.83458423 | 14.64890934 | 7.79E-45 | 8.52E-44 | 90.64218421 |
| CALB2 | -2.214765244 | 2.246617056 | -14.6480128 | 7.88E-45 | 8.62E-44 | 90.63104265 |
| GEMIN7 | 0.651650232 | 3.108563262 | 14.63738523 | 9.00E-45 | 9.83E-44 | 90.49900751 |
| ADGRL2 | -1.268040592 | 2.353875926 | -14.62045288 | 1.11E-44 | 1.21E-43 | 90.28878086 |
| WASF2 | -0.794089398 | 5.011780337 | -14.61857578 | 1.14E-44 | 1.24E-43 | 90.26548582 |
| FAM3D | -1.551509202 | 0.791764593 | -14.61383878 | 1.21E-44 | 1.32E-43 | 90.20670847 |
| CD248 | -1.369178047 | 4.182303282 | -14.61076843 | 1.25E-44 | 1.37E-43 | 90.16861814 |
| UST | -1.025928732 | 1.523980453 | -14.61074302 | 1.25E-44 | 1.37E-43 | 90.16830298 |
| RMI1 | 0.857348673 | 2.68006409 | 14.60603356 | 1.33E-44 | 1.45E-43 | 90.10988906 |
| HES6 | 1.431483665 | 1.994084294 | 14.60514405 | 1.35E-44 | 1.46E-43 | 90.09885757 |
| GPR75 | -0.609611874 | 0.718582508 | -14.60466791 | 1.35E-44 | 1.47E-43 | 90.09295274 |
| KDR | -1.027506608 | 2.935703914 | -14.60429022 | 1.36E-44 | 1.48E-43 | 90.08826882 |
| RBBP8NL | 0.971507157 | 1.880127157 | 14.59637131 | 1.50E-44 | 1.63E-43 | 89.99008439 |
| PSMG3 | 0.946932823 | 3.910204002 | 14.59390962 | 1.55E-44 | 1.68E-43 | 89.95957007 |
| CANT1 | 1.098114793 | 5.050550541 | 14.58875587 | 1.65E-44 | 1.79E-43 | 89.89569766 |
| RAB11FIP2 | -0.643284071 | 2.0532446 | -14.58621261 | 1.70E-44 | 1.84E-43 | 89.8641838 |
| MCTP1 | -0.650355786 | 0.967632535 | -14.57306405 | 2.01E-44 | 2.17E-43 | 89.70131986 |
| ADPRM | -0.592480605 | 2.258808487 | -14.5699053 | 2.09E-44 | 2.26E-43 | 89.66220946 |
| C7 | -2.021193568 | 1.780672796 | -14.56832437 | 2.13E-44 | 2.30E-43 | 89.64263716 |
| B4GALNT4 | 1.427109846 | 2.870007896 | 14.56753921 | 2.15E-44 | 2.32E-43 | 89.63291726 |
| RASGRF2 | -0.796191606 | 1.176982826 | -14.56668216 | 2.17E-44 | 2.35E-43 | 89.62230788 |
| SGCB | -0.975566387 | 3.372883512 | -14.56610142 | 2.19E-44 | 2.36E-43 | 89.61511908 |
| HMGA1 | 1.321275617 | 5.562043368 | 14.56327839 | 2.27E-44 | 2.44E-43 | 89.58017695 |
| YIF1A | 0.829491116 | 4.72194644 | 14.55925553 | 2.38E-44 | 2.57E-43 | 89.53039182 |
| MCRIP2 | 1.087650011 | 2.527687905 | 14.55655706 | 2.46E-44 | 2.65E-43 | 89.49700229 |
| PPM1L | -0.717459372 | 1.277456963 | -14.54856426 | 2.72E-44 | 2.93E-43 | 89.39812857 |
| SLC13A2 | -1.55595293 | 0.98721645 | -14.54730157 | 2.76E-44 | 2.97E-43 | 89.38251203 |
| FAM102A | 1.042255657 | 4.928506817 | 14.54595433 | 2.81E-44 | 3.02E-43 | 89.36585104 |
| CCND2 | -1.089335688 | 2.578734073 | -14.54305556 | 2.91E-44 | 3.13E-43 | 89.33000616 |
| CENPK | 0.716152679 | 1.104418986 | 14.53679424 | 3.15E-44 | 3.38E-43 | 89.25259845 |
| PLK4 | 0.908627211 | 1.679992947 | 14.53080603 | 3.39E-44 | 3.64E-43 | 89.17858918 |
| MRPL3 | 0.612295831 | 5.193127033 | 14.5248296 | 3.65E-44 | 3.92E-43 | 89.10474675 |
| TIPARP | -1.088479685 | 3.127742194 | -14.52433301 | 3.68E-44 | 3.94E-43 | 89.098612 |
| KIF26A | -0.71806792 | 0.860264597 | -14.5213053 | 3.82E-44 | 4.09E-43 | 89.06121189 |
| LGR4 | -1.36161034 | 2.450709789 | -14.51870663 | 3.94E-44 | 4.22E-43 | 89.02911583 |
| NR4A1 | -1.851197744 | 2.871048825 | -14.51860245 | 3.95E-44 | 4.22E-43 | 89.02782922 |
| PRG4 | -1.428589524 | 0.927070547 | -14.51413623 | 4.17E-44 | 4.46E-43 | 88.97267682 |
| NOV | -1.338465774 | 1.881795425 | -14.51133726 | 4.32E-44 | 4.61E-43 | 88.93811903 |
| IDH2 | 1.211595429 | 6.053950639 | 14.50953436 | 4.42E-44 | 4.72E-43 | 88.91586174 |
| TLL1 | -0.711934451 | 0.717936041 | -14.50307297 | 4.79E-44 | 5.10E-43 | 88.83611036 |
| CLCN2 | 0.716120578 | 1.658095097 | 14.48942918 | 5.67E-44 | 6.04E-43 | 88.66779019 |
| PGM1 | -1.008346626 | 4.06425749 | -14.48370148 | 6.09E-44 | 6.48E-43 | 88.59716206 |
| PNRC1 | -0.913491062 | 4.72774046 | -14.4812488 | 6.27E-44 | 6.68E-43 | 88.56692404 |
| SIPA1L3 | 0.895545371 | 3.221314858 | 14.48018266 | 6.36E-44 | 6.76E-43 | 88.55378124 |
| BPNT1 | 0.739371729 | 4.084484656 | 14.48012227 | 6.36E-44 | 6.76E-43 | 88.55303685 |
| EBNA1BP2 | 0.699685363 | 4.224465432 | 14.47941842 | 6.42E-44 | 6.82E-43 | 88.54436055 |
| RAB5IF | 0.827731384 | 3.706286626 | 14.47310091 | 6.94E-44 | 7.37E-43 | 88.46649839 |
| RRP12 | 0.661077422 | 2.768046982 | 14.47259193 | 6.98E-44 | 7.41E-43 | 88.46022633 |
| FAM184A | -0.620462676 | 0.688301763 | -14.47239444 | 7.00E-44 | 7.43E-43 | 88.45779278 |
| CYR61 | -1.806200213 | 5.976260238 | -14.47082197 | 7.14E-44 | 7.57E-43 | 88.4384168 |
| LATS2 | -0.821555547 | 2.418064626 | -14.46599527 | 7.58E-44 | 8.03E-43 | 88.37895136 |
| SRPRB | 0.694428321 | 4.448831887 | 14.4634584 | 7.82E-44 | 8.28E-43 | 88.34770242 |
| LRRC8C | -0.831201961 | 1.53022546 | -14.46207162 | 7.96E-44 | 8.42E-43 | 88.33062182 |
| UBQLN4 | 0.813625284 | 4.345012205 | 14.45883844 | 8.28E-44 | 8.76E-43 | 88.29080414 |
| RAB26 | 1.452220833 | 1.899392947 | 14.45865584 | 8.30E-44 | 8.77E-43 | 88.28855544 |
| SEC13 | 0.63915913 | 4.396389573 | 14.45617019 | 8.56E-44 | 9.04E-43 | 88.25794838 |
| ABHD11 | 1.15468679 | 4.452080848 | 14.43494568 | 1.11E-43 | 1.17E-42 | 87.99675077 |
| CENPO | 0.710545372 | 1.715303772 | 14.43382868 | 1.13E-43 | 1.19E-42 | 87.98301203 |
| LPCAT1 | 0.987408503 | 3.856297785 | 14.42852476 | 1.20E-43 | 1.27E-42 | 87.91778557 |
| ECHDC3 | -1.196633693 | 3.20008805 | -14.42837346 | 1.21E-43 | 1.27E-42 | 87.91592508 |
| LMBRD1 | -0.74343838 | 4.571878936 | -14.42774319 | 1.22E-43 | 1.28E-42 | 87.90817539 |
| TRAIP | 0.666329663 | 1.510354756 | 14.42521799 | 1.25E-43 | 1.32E-42 | 87.87712808 |
| NDUFS2 | 0.708284748 | 5.320466863 | 14.42337678 | 1.28E-43 | 1.35E-42 | 87.85449299 |
| PSME2 | 1.040480111 | 5.098196705 | 14.41845672 | 1.36E-43 | 1.43E-42 | 87.79401746 |
| RILP | -0.829091386 | 1.679029538 | -14.41581871 | 1.41E-43 | 1.48E-42 | 87.76159794 |
| PDRG1 | 0.933874641 | 3.917807046 | 14.40995118 | 1.52E-43 | 1.59E-42 | 87.6895048 |
| GAS1 | -1.503048039 | 3.246056796 | -14.40688893 | 1.57E-43 | 1.65E-42 | 87.6518878 |
| OSBPL1A | -0.943560194 | 3.227245268 | -14.40217544 | 1.67E-43 | 1.75E-42 | 87.5939978 |
| MRPL13 | 0.928702731 | 3.471603527 | 14.4004345 | 1.70E-43 | 1.79E-42 | 87.57261939 |
| HIST1H4I | 1.422289673 | 3.308496894 | 14.3993358 | 1.73E-43 | 1.81E-42 | 87.55912848 |
| GPR68 | 1.0389534 | 2.08985288 | 14.39131531 | 1.91E-43 | 2.00E-42 | 87.46066698 |
| RBPMS2 | -0.977118324 | 1.4456203 | -14.38990481 | 1.94E-43 | 2.03E-42 | 87.44335538 |
| RBMS1 | -0.883190216 | 2.559262352 | -14.38628687 | 2.03E-43 | 2.12E-42 | 87.39895649 |
| NAB1 | -0.918064428 | 3.229280977 | -14.37976341 | 2.20E-43 | 2.30E-42 | 87.31892123 |
| HSD17B7 | 0.799459638 | 2.078695843 | 14.37900235 | 2.22E-43 | 2.32E-42 | 87.30958561 |
| SMG7 | 0.628112102 | 4.138552784 | 14.36556851 | 2.62E-43 | 2.73E-42 | 87.14485509 |
| RNASE4 | -0.736743941 | 1.042615248 | -14.36339322 | 2.69E-43 | 2.81E-42 | 87.1181912 |
| MED25 | 0.751693346 | 3.292327162 | 14.36004697 | 2.80E-43 | 2.92E-42 | 87.07717962 |
| CREB3L4 | 1.50529197 | 5.11869149 | 14.35648531 | 2.93E-43 | 3.05E-42 | 87.03353535 |
| NDNF | -1.02225966 | 0.883609373 | -14.35585815 | 2.95E-43 | 3.08E-42 | 87.02585104 |
| LRP4 | -0.77730753 | 0.796734184 | -14.35232851 | 3.08E-43 | 3.21E-42 | 86.982608 |
| DSN1 | 0.856691809 | 3.37053743 | 14.35101384 | 3.13E-43 | 3.26E-42 | 86.9665034 |
| DDX41 | 0.61267733 | 4.013062999 | 14.34842138 | 3.24E-43 | 3.37E-42 | 86.93474904 |
| ZNF559 | -0.686236767 | 1.731956977 | -14.34575129 | 3.34E-43 | 3.47E-42 | 86.90204808 |
| CYGB | -1.130338615 | 2.494698497 | -14.33817629 | 3.67E-43 | 3.81E-42 | 86.80929931 |
| MXI1 | -1.00049223 | 3.49118462 | -14.33702391 | 3.72E-43 | 3.86E-42 | 86.79519252 |
| PDZD11 | 0.675340028 | 4.899181529 | 14.33602391 | 3.77E-43 | 3.91E-42 | 86.78295184 |
| H6PD | -0.804639799 | 3.625059888 | -14.33415801 | 3.86E-43 | 3.99E-42 | 86.76011331 |
| ORAI2 | 0.720613842 | 2.413735386 | 14.33301578 | 3.91E-43 | 4.05E-42 | 86.74613365 |
| LAMB3 | -2.063482901 | 3.346332864 | -14.31836216 | 4.68E-43 | 4.84E-42 | 86.566858 |
| HMOX2 | 0.667320419 | 3.870156616 | 14.31561144 | 4.84E-43 | 5.01E-42 | 86.53321957 |
| COL14A1 | -1.982554494 | 3.301200508 | -14.31284408 | 5.01E-43 | 5.18E-42 | 86.4993823 |
| EXOSC4 | 1.187487391 | 3.964018455 | 14.30656225 | 5.41E-43 | 5.59E-42 | 86.42258974 |
| KCTD13 | 0.751951189 | 2.400685582 | 14.30544447 | 5.49E-43 | 5.66E-42 | 86.40892794 |
| MOGS | 0.66339124 | 4.456754311 | 14.30202964 | 5.72E-43 | 5.90E-42 | 86.36719551 |
| ALPL | -1.591313509 | 2.598095758 | -14.29744206 | 6.06E-43 | 6.23E-42 | 86.31114211 |
| PAICS | 0.853537388 | 4.520983969 | 14.28751245 | 6.84E-43 | 7.04E-42 | 86.18986078 |
| FGFBP1 | -1.445316218 | 0.636022024 | -14.28665829 | 6.91E-43 | 7.11E-42 | 86.17943071 |
| LRRC45 | 1.098375509 | 2.911221267 | 14.27827849 | 7.66E-43 | 7.87E-42 | 86.07712977 |
| GARS | 0.750487067 | 4.86026372 | 14.26820299 | 8.67E-43 | 8.90E-42 | 85.95418417 |
| CENPI | 0.812121964 | 1.148626576 | 14.26773039 | 8.72E-43 | 8.95E-42 | 85.94841872 |
| PSMD4 | 0.75684444 | 5.809846158 | 14.26694053 | 8.80E-43 | 9.03E-42 | 85.93878335 |
| PHKG1 | -0.744781479 | 0.713530982 | -14.26583244 | 8.93E-43 | 9.15E-42 | 85.92526663 |
| ACOT7 | 1.01751666 | 2.876706739 | 14.26565037 | 8.95E-43 | 9.17E-42 | 85.9230457 |
| EBP | 0.935620656 | 4.04705273 | 14.2640467 | 9.12E-43 | 9.34E-42 | 85.9034852 |
| RRAGD | -1.116064102 | 1.909256588 | -14.24646941 | 1.13E-42 | 1.16E-41 | 85.68919137 |
| ARL6IP1 | 0.880481177 | 6.406255144 | 14.23794612 | 1.26E-42 | 1.28E-41 | 85.58534713 |
| MEN1 | 0.659562548 | 3.642371458 | 14.23771378 | 1.26E-42 | 1.29E-41 | 85.58251701 |
| RPS6KA2 | -0.904735325 | 2.509574266 | -14.23712629 | 1.27E-42 | 1.29E-41 | 85.57536105 |
| APOD | -3.156767327 | 5.957953724 | -14.2355986 | 1.29E-42 | 1.32E-41 | 85.55675367 |
| MICAL2 | 1.057813886 | 2.704938673 | 14.23253059 | 1.34E-42 | 1.37E-41 | 85.51938948 |
| METTL26 | 1.013967949 | 4.505024525 | 14.22407712 | 1.49E-42 | 1.51E-41 | 85.41646756 |
| AXIN1 | 0.722454552 | 2.893027399 | 14.21760943 | 1.61E-42 | 1.64E-41 | 85.33775195 |
| CXCL10 | 2.449422201 | 4.205712724 | 14.2163762 | 1.64E-42 | 1.66E-41 | 85.32274579 |
| CHD9 | -0.798969835 | 2.05775087 | -14.20712039 | 1.83E-42 | 1.86E-41 | 85.21014857 |
| EIF4A3 | 0.742099473 | 4.516470977 | 14.20244983 | 1.94E-42 | 1.97E-41 | 85.15335079 |
| IRX1 | -2.055826441 | 1.798061714 | -14.20068949 | 1.98E-42 | 2.01E-41 | 85.13194711 |
| FNDC10 | 1.278384983 | 2.95442492 | 14.1971297 | 2.07E-42 | 2.10E-41 | 85.08867005 |
| MCM10 | 1.210916438 | 1.47989693 | 14.19473495 | 2.13E-42 | 2.16E-41 | 85.05956092 |
| C19orf48 | 1.036571265 | 3.985413023 | 14.19406186 | 2.15E-42 | 2.18E-41 | 85.05137983 |
| JAZF1 | -0.724034052 | 2.267598973 | -14.19364238 | 2.16E-42 | 2.19E-41 | 85.04628153 |
| ZDHHC16 | 0.662471661 | 3.84808619 | 14.18822137 | 2.31E-42 | 2.33E-41 | 84.98040355 |
| NRP1 | -1.01786995 | 3.261315049 | -14.18657992 | 2.35E-42 | 2.38E-41 | 84.96045957 |
| TESMIN | 1.283531061 | 2.513282064 | 14.18638956 | 2.36E-42 | 2.38E-41 | 84.95814679 |
| ATP5IF1 | 0.873823313 | 4.975807049 | 14.18569833 | 2.38E-42 | 2.40E-41 | 84.94974883 |
| ELOB | 0.989802631 | 6.796478783 | 14.18390609 | 2.43E-42 | 2.45E-41 | 84.92797561 |
| SMIM22 | 1.983103532 | 4.199493146 | 14.17872824 | 2.59E-42 | 2.61E-41 | 84.86508311 |
| SCNM1 | 0.762455841 | 3.314430968 | 14.17820159 | 2.61E-42 | 2.63E-41 | 84.85868708 |
| E2F8 | 0.996978042 | 1.139234193 | 14.16393491 | 3.10E-42 | 3.12E-41 | 84.68548669 |
| ACKR3 | -1.434574761 | 3.628821141 | -14.15995979 | 3.26E-42 | 3.28E-41 | 84.63725012 |
| RCAN2 | -0.875990487 | 2.106963962 | -14.15504086 | 3.46E-42 | 3.48E-41 | 84.57757412 |
| HPS5 | -0.646197704 | 2.586021599 | -14.15387108 | 3.51E-42 | 3.53E-41 | 84.56338467 |
| FTO | -0.921235754 | 2.658929668 | -14.14786781 | 3.78E-42 | 3.79E-41 | 84.49057785 |
| CLSPN | 0.932229477 | 1.125743044 | 14.14262293 | 4.02E-42 | 4.04E-41 | 84.42698691 |
| KIAA0355 | -0.672116516 | 2.592911076 | -14.14238421 | 4.04E-42 | 4.05E-41 | 84.42409288 |
| GPRC5A | 1.912921422 | 4.719332402 | 14.1330739 | 4.52E-42 | 4.53E-41 | 84.31125383 |
| UFC1 | 0.733717618 | 5.952367892 | 14.13050199 | 4.66E-42 | 4.68E-41 | 84.28009213 |
| DNA2 | 0.808854049 | 1.459844078 | 14.12815109 | 4.80E-42 | 4.81E-41 | 84.25161188 |
| ANKRD33B | -0.585505433 | 0.605381743 | -14.12507323 | 4.98E-42 | 4.99E-41 | 84.21432976 |
| KLF2 | -1.423328121 | 3.832453928 | -14.12485777 | 5.00E-42 | 5.00E-41 | 84.21172007 |
| SERPING1 | -1.370824389 | 6.182329439 | -14.12442442 | 5.02E-42 | 5.02E-41 | 84.20647154 |
| MLST8 | 0.729123363 | 3.568674001 | 14.12264712 | 5.13E-42 | 5.13E-41 | 84.18494641 |
| EMC9 | 0.924382478 | 3.044178621 | 14.12091606 | 5.24E-42 | 5.24E-41 | 84.16398331 |
| KNSTRN | 0.83910417 | 2.479070478 | 14.11785052 | 5.44E-42 | 5.43E-41 | 84.126864 |
| PLXNA3 | 0.820595809 | 2.716147988 | 14.11641066 | 5.54E-42 | 5.53E-41 | 84.10943137 |
| P4HB | 0.869227655 | 7.387226208 | 14.11629165 | 5.55E-42 | 5.53E-41 | 84.10799058 |
| DCAF13 | 0.884907903 | 2.958303689 | 14.11401699 | 5.70E-42 | 5.68E-41 | 84.08045366 |
| PRKCA | -0.754160914 | 0.863743849 | -14.11123794 | 5.90E-42 | 5.88E-41 | 84.04681483 |
| RNF157 | -0.911718362 | 0.913987931 | -14.10702602 | 6.21E-42 | 6.18E-41 | 83.99584116 |
| ARRDC1 | 0.979663118 | 3.91254119 | 14.09793947 | 6.93E-42 | 6.89E-41 | 83.8859105 |
| PTPN6 | 0.821744097 | 3.422225816 | 14.09578804 | 7.12E-42 | 7.07E-41 | 83.8598895 |
| PTPRZ1 | -0.966673041 | 0.450099367 | -14.09194104 | 7.46E-42 | 7.40E-41 | 83.81336821 |
| CHAF1B | 0.904113096 | 2.244728903 | 14.08461032 | 8.15E-42 | 8.09E-41 | 83.72474381 |
| HELLS | 0.775262453 | 1.357781983 | 14.08308197 | 8.31E-42 | 8.23E-41 | 83.70627113 |
| MITF | -0.828755216 | 1.589435222 | -14.07872642 | 8.76E-42 | 8.68E-41 | 83.65363469 |
| VOPP1 | 0.796324462 | 3.373436313 | 14.0710396 | 9.61E-42 | 9.52E-41 | 83.56076862 |
| CD99L2 | -0.825961244 | 3.252844499 | -14.06984762 | 9.75E-42 | 9.65E-41 | 83.54637128 |
| SATB1 | -0.942512975 | 1.399907754 | -14.0691431 | 9.84E-42 | 9.73E-41 | 83.53786219 |
| TMEM140 | -0.795300079 | 3.49329913 | -14.06826987 | 9.94E-42 | 9.83E-41 | 83.52731583 |
| ATXN1L | -0.764192953 | 2.830153196 | -14.06305529 | 1.06E-41 | 1.05E-40 | 83.46434695 |
| ABCA1 | -0.976661082 | 2.241577327 | -14.05965957 | 1.10E-41 | 1.09E-40 | 83.42335088 |
| SLC27A4 | 0.870165824 | 3.946467673 | 14.05812136 | 1.12E-41 | 1.11E-40 | 83.40478249 |
| ACTL6A | 0.756262773 | 4.248959264 | 14.05510809 | 1.17E-41 | 1.15E-40 | 83.36841244 |
| SNRPC | 0.738163026 | 5.753371253 | 14.05326311 | 1.19E-41 | 1.17E-40 | 83.34614645 |
| EME1 | 0.898781996 | 1.180271129 | 14.04919622 | 1.25E-41 | 1.23E-40 | 83.29707266 |
| C22orf23 | -0.614869285 | 0.810391481 | -14.04694746 | 1.29E-41 | 1.27E-40 | 83.26994216 |
| BIN1 | -1.060687499 | 1.885366267 | -14.04579559 | 1.31E-41 | 1.28E-40 | 83.25604639 |
| ITM2B | -0.777176987 | 5.458827644 | -14.04078833 | 1.39E-41 | 1.36E-40 | 83.19565003 |
| PRDX1 | 0.878307585 | 7.752272352 | 14.03672232 | 1.46E-41 | 1.43E-40 | 83.14661824 |
| NCAM1 | -0.763925636 | 0.458924354 | -14.03435983 | 1.50E-41 | 1.47E-40 | 83.11813376 |
| PAGR1 | 0.957098231 | 2.716214843 | 14.03153007 | 1.55E-41 | 1.52E-40 | 83.08401991 |
| COL5A1 | 1.711456061 | 4.971772779 | 14.02865423 | 1.61E-41 | 1.58E-40 | 83.04935571 |
| TSC22D1 | -0.815092059 | 4.61277465 | -14.02829301 | 1.61E-41 | 1.58E-40 | 83.04500209 |
| PELI1 | -1.085783896 | 3.013879924 | -14.02468912 | 1.69E-41 | 1.65E-40 | 83.0015702 |
| OSTC | 0.606593659 | 5.768480149 | 14.02175615 | 1.75E-41 | 1.71E-40 | 82.96622979 |
| FOLR2 | -1.549514836 | 2.722672387 | -14.0207247 | 1.77E-41 | 1.73E-40 | 82.95380276 |
| MFSD3 | 1.25351573 | 3.75977011 | 14.0197267 | 1.79E-41 | 1.75E-40 | 82.94177934 |
| NLRP1 | -0.746010369 | 1.260029342 | -14.01686472 | 1.85E-41 | 1.81E-40 | 82.90730306 |
| C1orf35 | 0.856022544 | 2.58698347 | 14.01398702 | 1.92E-41 | 1.87E-40 | 82.87264246 |
| MAL2 | 1.398666154 | 6.149056923 | 14.0139762 | 1.92E-41 | 1.87E-40 | 82.87251208 |
| CTSG | -1.291676127 | 1.092343928 | -14.00732253 | 2.08E-41 | 2.03E-40 | 82.79239128 |
| GFOD1 | -0.751121076 | 1.304613519 | -14.00135422 | 2.24E-41 | 2.18E-40 | 82.72054662 |
| PLTP | -1.362514297 | 4.602561197 | -13.99631135 | 2.38E-41 | 2.32E-40 | 82.65985925 |
| FKBPL | 0.751156697 | 2.999338693 | 13.99356643 | 2.46E-41 | 2.39E-40 | 82.62683269 |
| CHTF18 | 1.039108521 | 1.993462716 | 13.99156122 | 2.52E-41 | 2.45E-40 | 82.60270921 |
| TRIM2 | -1.381972731 | 2.17607603 | -13.99076344 | 2.54E-41 | 2.47E-40 | 82.59311229 |
| ETFDH | -0.657357674 | 2.599520352 | -13.99075113 | 2.54E-41 | 2.47E-40 | 82.59296412 |
| LAMC1 | -1.114193961 | 4.775794799 | -13.98895007 | 2.60E-41 | 2.53E-40 | 82.57129965 |
| GNG7 | -1.041851235 | 1.802593193 | -13.98601472 | 2.69E-41 | 2.62E-40 | 82.5359954 |
| TMEM141 | 1.067172849 | 5.153508002 | 13.98302542 | 2.79E-41 | 2.71E-40 | 82.50004784 |
| C1orf112 | 0.698695704 | 1.612408385 | 13.97732612 | 2.99E-41 | 2.90E-40 | 82.43152663 |
| F11R | 0.812090161 | 5.043557083 | 13.96779334 | 3.36E-41 | 3.25E-40 | 82.31696146 |
| POLQ | 0.671332552 | 0.823067964 | 13.96144999 | 3.62E-41 | 3.51E-40 | 82.24075813 |
| INTS6L | -0.747637925 | 1.122212793 | -13.96014508 | 3.68E-41 | 3.57E-40 | 82.22508517 |
| C21orf58 | 0.769720374 | 1.373873788 | 13.95719429 | 3.82E-41 | 3.69E-40 | 82.18964789 |
| ANKRD65 | -1.099976609 | 1.715304881 | -13.95661752 | 3.84E-41 | 3.72E-40 | 82.18272191 |
| ALKAL2 | -0.907761928 | 0.364053078 | -13.95476594 | 3.93E-41 | 3.80E-40 | 82.16048887 |
| GRHL2 | 1.002025502 | 3.942219098 | 13.95441721 | 3.95E-41 | 3.81E-40 | 82.15630172 |
| TSEN34 | 0.754806156 | 4.094251994 | 13.95305139 | 4.01E-41 | 3.87E-40 | 82.13990321 |
| ZNF367 | 0.929101361 | 1.817248427 | 13.95147061 | 4.09E-41 | 3.94E-40 | 82.12092523 |
| OGFRL1 | -1.18955656 | 3.282983528 | -13.950006 | 4.16E-41 | 4.01E-40 | 82.10334336 |
| PCDHB4 | -0.84209475 | 1.10170973 | -13.94951346 | 4.19E-41 | 4.03E-40 | 82.09743094 |
| PXDC1 | -1.190714045 | 3.573957992 | -13.94743957 | 4.29E-41 | 4.13E-40 | 82.07253791 |
| ECE2 | 0.848496195 | 1.410315553 | 13.93909137 | 4.75E-41 | 4.57E-40 | 81.97236064 |
| RELT | 0.636250711 | 1.129759711 | 13.93481978 | 5.00E-41 | 4.80E-40 | 81.92111893 |
| MYO9A | -0.665477552 | 1.720089922 | -13.9346688 | 5.01E-41 | 4.81E-40 | 81.91930801 |
| KCNJ8 | -1.074612407 | 2.305306637 | -13.93461963 | 5.01E-41 | 4.81E-40 | 81.91871825 |
| SNCG | -2.252897588 | 3.429520077 | -13.92036517 | 5.95E-41 | 5.70E-40 | 81.74780653 |
| TMEM147 | 0.793566556 | 5.240089302 | 13.9181329 | 6.11E-41 | 5.85E-40 | 81.72105294 |
| PDZRN3 | -1.144611897 | 2.448086505 | -13.91463524 | 6.37E-41 | 6.10E-40 | 81.67913991 |
| MAP1B | -1.207762927 | 2.456103153 | -13.90920931 | 6.80E-41 | 6.51E-40 | 81.61413529 |
| EDN1 | -1.537122522 | 2.500581808 | -13.90133497 | 7.48E-41 | 7.15E-40 | 81.51983028 |
| TPPP | -1.082506282 | 1.298544048 | -13.89688098 | 7.89E-41 | 7.54E-40 | 81.4665054 |
| BVES | -0.62736156 | 0.705660679 | -13.89487702 | 8.09E-41 | 7.71E-40 | 81.44251717 |
| ZNF423 | -0.776856027 | 1.075518197 | -13.89265199 | 8.31E-41 | 7.92E-40 | 81.41588565 |
| SLC9A3R1 | 1.645220262 | 6.746178198 | 13.88833782 | 8.75E-41 | 8.34E-40 | 81.36425789 |
| CNIH2 | 1.164635058 | 1.379909449 | 13.87453187 | 1.03E-40 | 9.83E-40 | 81.19911959 |
| COX6C | 1.975651881 | 6.636701795 | 13.87412341 | 1.04E-40 | 9.87E-40 | 81.1942356 |
| C1QTNF1 | -1.237055686 | 2.715885473 | -13.87279316 | 1.05E-40 | 1.00E-39 | 81.17833059 |
| GDPD5 | -0.958296727 | 1.534020285 | -13.87024042 | 1.09E-40 | 1.03E-39 | 81.14781204 |
| ATP6V0C | 0.87949389 | 4.585880706 | 13.86032764 | 1.23E-40 | 1.16E-39 | 81.02934135 |
| SCARF1 | -0.659632726 | 1.496308809 | -13.85952544 | 1.24E-40 | 1.17E-39 | 81.0197567 |
| TMEM71 | -0.907542888 | 0.806285593 | -13.85241037 | 1.35E-40 | 1.28E-39 | 80.93476371 |
| SMYD3 | 0.775865539 | 1.484266733 | 13.84523698 | 1.47E-40 | 1.39E-39 | 80.84910598 |
| PRUNE2 | -0.759977279 | 0.688009908 | -13.8441367 | 1.49E-40 | 1.41E-39 | 80.83597037 |
| SHMT2 | 0.796278556 | 4.188014994 | 13.84375421 | 1.49E-40 | 1.41E-39 | 80.83140426 |
| AUNIP | 0.874382936 | 1.288582948 | 13.84324676 | 1.50E-40 | 1.42E-39 | 80.82534639 |
| BCAS4 | 1.159944036 | 2.105877143 | 13.84180444 | 1.53E-40 | 1.45E-39 | 80.80812938 |
| CPNE8 | -0.779664585 | 1.275455987 | -13.83462874 | 1.67E-40 | 1.57E-39 | 80.72249166 |
| YIPF2 | 0.719253102 | 4.354174541 | 13.83336502 | 1.69E-40 | 1.60E-39 | 80.70741325 |
| GSPT2 | -1.109032033 | 1.835151409 | -13.83275533 | 1.71E-40 | 1.61E-39 | 80.7001389 |
| NAP1L5 | -0.77481729 | 1.746084415 | -13.83241046 | 1.71E-40 | 1.61E-39 | 80.6960243 |
| CPXM2 | -1.375638922 | 2.692325656 | -13.83030065 | 1.76E-40 | 1.66E-39 | 80.67085412 |
| TFDP2 | -0.612463048 | 2.476751951 | -13.82821362 | 1.80E-40 | 1.70E-39 | 80.64595834 |
| HIST1H3H | 2.008338571 | 2.344447787 | 13.82444944 | 1.88E-40 | 1.77E-39 | 80.60106301 |
| NFAT5 | -0.926923486 | 1.813671783 | -13.82422233 | 1.89E-40 | 1.78E-39 | 80.59835458 |
| SERINC2 | 1.397375928 | 5.460474608 | 13.82055196 | 1.97E-40 | 1.86E-39 | 80.55458726 |
| GMIP | 0.749231726 | 2.810237779 | 13.81878701 | 2.02E-40 | 1.90E-39 | 80.53354418 |
| SOCS5 | -0.706450842 | 3.009238605 | -13.81675163 | 2.07E-40 | 1.94E-39 | 80.50927919 |
| NR4A3 | -1.095128408 | 0.924891859 | -13.81613003 | 2.08E-40 | 1.96E-39 | 80.50186918 |
| EFNB1 | -0.90062714 | 3.149327133 | -13.81565052 | 2.09E-40 | 1.96E-39 | 80.4961532 |
| FRMD3 | -0.980192254 | 1.09694376 | -13.81147865 | 2.20E-40 | 2.06E-39 | 80.44642884 |
| ALYREF | 1.057637315 | 5.056971787 | 13.81065291 | 2.22E-40 | 2.08E-39 | 80.43658816 |
| ITIH2 | -0.846724363 | 0.345201251 | -13.80280515 | 2.44E-40 | 2.28E-39 | 80.34308454 |
| PHLDB2 | -1.03225647 | 1.85306225 | -13.80145212 | 2.48E-40 | 2.32E-39 | 80.32696748 |
| COPB2 | 0.657618853 | 5.005531625 | 13.79764138 | 2.60E-40 | 2.43E-39 | 80.28158087 |
| CFH | -1.197778266 | 2.740096858 | -13.78857369 | 2.90E-40 | 2.70E-39 | 80.17361954 |
| SLC39A11 | 1.133336948 | 3.943897805 | 13.78588873 | 2.99E-40 | 2.79E-39 | 80.14166194 |
| CENPS | 0.594637456 | 1.607996192 | 13.78494607 | 3.02E-40 | 2.82E-39 | 80.13044301 |
| ZDBF2 | -0.70352538 | 0.745892352 | -13.78210985 | 3.13E-40 | 2.92E-39 | 80.09669159 |
| BRI3BP | 0.802444225 | 2.649383296 | 13.7803721 | 3.19E-40 | 2.98E-39 | 80.07601462 |
| MICAL3 | -0.593640422 | 0.896142917 | -13.77419223 | 3.44E-40 | 3.20E-39 | 80.00249749 |
| TBX18 | -0.661672508 | 0.806295392 | -13.77323999 | 3.48E-40 | 3.24E-39 | 79.99117161 |
| TMEM125 | 1.106038832 | 3.481819489 | 13.77102882 | 3.57E-40 | 3.32E-39 | 79.96487414 |
| RANGAP1 | 0.845278426 | 4.575190307 | 13.7700414 | 3.61E-40 | 3.36E-39 | 79.95313175 |
| BLCAP | -0.599314779 | 3.567207511 | -13.760321 | 4.06E-40 | 3.77E-39 | 79.83756971 |
| GSTM2 | -1.100286644 | 1.436225142 | -13.75727783 | 4.21E-40 | 3.91E-39 | 79.80140278 |
| KLC2 | 0.804231835 | 2.983466295 | 13.75644642 | 4.25E-40 | 3.94E-39 | 79.79152281 |
| S100P | 3.017448967 | 3.586980848 | 13.75575383 | 4.29E-40 | 3.98E-39 | 79.78329283 |
| PSMB4 | 0.791936055 | 6.739054936 | 13.75409396 | 4.37E-40 | 4.05E-39 | 79.76356991 |
| MRPL24 | 0.933961137 | 5.539369212 | 13.74499526 | 4.88E-40 | 4.52E-39 | 79.65548836 |
| RGMA | -1.094393511 | 1.063323287 | -13.7419709 | 5.06E-40 | 4.68E-39 | 79.6195742 |
| COPA | 0.743112777 | 6.126756731 | 13.74121685 | 5.10E-40 | 4.72E-39 | 79.61062076 |
| TPI1 | 0.855627221 | 7.090121512 | 13.73623819 | 5.41E-40 | 5.00E-39 | 79.55151418 |
| CHMP4B | 0.636572159 | 6.869312471 | 13.73439592 | 5.53E-40 | 5.11E-39 | 79.52964674 |
| MPV17L2 | 0.664961232 | 3.003463186 | 13.73287685 | 5.64E-40 | 5.20E-39 | 79.51161723 |
| CDCA2 | 1.135974275 | 1.453431355 | 13.7280094 | 5.97E-40 | 5.51E-39 | 79.45385633 |
| TUBB6 | -1.190043221 | 3.738918696 | -13.72715236 | 6.03E-40 | 5.57E-39 | 79.4436876 |
| CLN6 | 0.666213317 | 3.623155281 | 13.72514548 | 6.18E-40 | 5.70E-39 | 79.41987789 |
| RFX5 | 0.746780085 | 3.859940332 | 13.72402988 | 6.26E-40 | 5.77E-39 | 79.40664345 |
| BUD23 | 0.65366957 | 3.498591097 | 13.72397556 | 6.27E-40 | 5.77E-39 | 79.40599901 |
| SKI | -0.698171453 | 4.081590703 | -13.7234719 | 6.30E-40 | 5.81E-39 | 79.40002433 |
| PRR11 | 1.344570915 | 2.068945518 | 13.7229715 | 6.34E-40 | 5.84E-39 | 79.39408848 |
| DNMT1 | 0.821606235 | 3.470434211 | 13.72270782 | 6.36E-40 | 5.85E-39 | 79.39096069 |
| KRT14 | -3.491237502 | 4.108547955 | -13.71991594 | 6.58E-40 | 6.05E-39 | 79.3578462 |
| ADAR | 0.820668543 | 6.055709289 | 13.71710599 | 6.80E-40 | 6.25E-39 | 79.32452226 |
| DNAJB11 | 0.672827729 | 3.700186927 | 13.71554096 | 6.93E-40 | 6.37E-39 | 79.30596435 |
| FLYWCH2 | 0.997764363 | 4.355827179 | 13.71539201 | 6.94E-40 | 6.38E-39 | 79.30419827 |
| STARD10 | 1.768228199 | 5.196744864 | 13.70732583 | 7.64E-40 | 7.02E-39 | 79.20857576 |
| NDUFAB1 | 0.673334783 | 5.095946792 | 13.70464889 | 7.89E-40 | 7.24E-39 | 79.1768504 |
| TCEAL2 | -0.962400132 | 0.552493153 | -13.70369959 | 7.98E-40 | 7.32E-39 | 79.16560104 |
| PEX11B | 0.680983131 | 4.858279873 | 13.69894524 | 8.45E-40 | 7.74E-39 | 79.10926951 |
| NDE1 | 0.679530304 | 2.562453882 | 13.68699094 | 9.74E-40 | 8.92E-39 | 78.96769318 |
| SOCS2 | -1.286819925 | 1.683940714 | -13.68642017 | 9.80E-40 | 8.98E-39 | 78.96093568 |
| PTK6 | 1.380398131 | 2.668955688 | 13.68405502 | 1.01E-39 | 9.23E-39 | 78.9329364 |
| LRCH1 | -0.64916696 | 2.223763371 | -13.68385269 | 1.01E-39 | 9.24E-39 | 78.93054134 |
| ABLIM3 | -1.468123503 | 2.385329004 | -13.68370456 | 1.01E-39 | 9.26E-39 | 78.92878792 |
| DYNLT1 | 0.753228955 | 4.870052563 | 13.67369967 | 1.14E-39 | 1.04E-38 | 78.81038886 |
| LCAT | -0.740420204 | 1.345954986 | -13.65747953 | 1.38E-39 | 1.26E-38 | 78.61857214 |
| DOC2B | -1.142960201 | 1.43567322 | -13.65566844 | 1.41E-39 | 1.29E-38 | 78.59716482 |
| ANXA3 | -1.561105822 | 2.346538316 | -13.65186817 | 1.48E-39 | 1.35E-38 | 78.55225185 |
| TAPBP | 0.814287921 | 5.724696698 | 13.65083033 | 1.50E-39 | 1.36E-38 | 78.53998793 |
| RAN | 0.637070852 | 5.606554645 | 13.64988128 | 1.51E-39 | 1.38E-38 | 78.52877383 |
| TAGLN | -1.542227399 | 6.063696143 | -13.64901158 | 1.53E-39 | 1.39E-38 | 78.51849777 |
| PLEK2 | 0.973221396 | 2.421105394 | 13.64748707 | 1.56E-39 | 1.42E-38 | 78.50048584 |
| PRR14 | 0.669996781 | 3.644620598 | 13.6470958 | 1.56E-39 | 1.42E-38 | 78.4958633 |
| THRA | -0.934097486 | 3.041566081 | -13.64674974 | 1.57E-39 | 1.43E-38 | 78.4917749 |
| PUSL1 | 0.830833201 | 2.448740771 | 13.64590335 | 1.59E-39 | 1.44E-38 | 78.481776 |
| LONRF1 | -0.732232571 | 2.151398574 | -13.64506664 | 1.60E-39 | 1.46E-38 | 78.47189181 |
| SEC61G | 0.867575 | 4.82281077 | 13.64478651 | 1.61E-39 | 1.46E-38 | 78.46858274 |
| RRAS2 | -0.991741171 | 2.297146536 | -13.64429567 | 1.62E-39 | 1.47E-38 | 78.46278474 |
| CHCHD1 | 0.764665299 | 4.400840064 | 13.64293777 | 1.64E-39 | 1.49E-38 | 78.44674531 |
| FILIP1L | -1.170805007 | 2.881429434 | -13.64147888 | 1.67E-39 | 1.52E-38 | 78.42951427 |
| TIMM23 | 0.602692176 | 5.536722092 | 13.63406717 | 1.83E-39 | 1.66E-38 | 78.3419952 |
| RAE1 | 0.705483224 | 3.227726153 | 13.63151266 | 1.88E-39 | 1.71E-38 | 78.31183898 |
| COQ8A | -0.863064201 | 3.085822235 | -13.62757813 | 1.97E-39 | 1.79E-38 | 78.26539978 |
| HNMT | -0.909601272 | 3.278552222 | -13.62683841 | 1.99E-39 | 1.80E-38 | 78.25666993 |
| PDE8B | -1.002119515 | 1.258042352 | -13.62243324 | 2.10E-39 | 1.90E-38 | 78.20468937 |
| CCDC80 | -1.670933203 | 3.862309057 | -13.62151704 | 2.12E-39 | 1.92E-38 | 78.19387989 |
| NHP2 | 0.7807473 | 5.141153828 | 13.60997235 | 2.43E-39 | 2.20E-38 | 78.05771885 |
| ZDHHC2 | -1.01076985 | 1.728441234 | -13.60085363 | 2.71E-39 | 2.44E-38 | 77.95023023 |
| CSK | 0.723790211 | 3.899021154 | 13.59670253 | 2.85E-39 | 2.56E-38 | 77.90131576 |
| TCTEX1D2 | 0.799875846 | 2.324996435 | 13.59639523 | 2.86E-39 | 2.57E-38 | 77.89769521 |
| WDR76 | 0.808627071 | 2.097756182 | 13.59563517 | 2.88E-39 | 2.60E-38 | 77.88874036 |
| NSUN5 | 0.735943004 | 3.114517205 | 13.59088433 | 3.05E-39 | 2.74E-38 | 77.83277556 |
| CREBL2 | -0.762032643 | 4.352749177 | -13.58587388 | 3.23E-39 | 2.91E-38 | 77.77376822 |
| SHKBP1 | 0.722480787 | 3.806329383 | 13.58514405 | 3.26E-39 | 2.93E-38 | 77.76517445 |
| ADPRH | -0.630087083 | 1.955924958 | -13.58003431 | 3.47E-39 | 3.11E-38 | 77.70501642 |
| PRAF2 | 0.846666071 | 4.143076331 | 13.57833639 | 3.54E-39 | 3.17E-38 | 77.68503024 |
| BHLHE41 | -1.399626019 | 3.452059073 | -13.5770408 | 3.59E-39 | 3.22E-38 | 77.66978099 |
| SGO2 | 0.727910446 | 1.598852151 | 13.57661684 | 3.61E-39 | 3.24E-38 | 77.66479118 |
| ACADS | -1.210796042 | 2.857891125 | -13.57324752 | 3.76E-39 | 3.37E-38 | 77.62514003 |
| SAMD10 | 0.847751301 | 2.43056973 | 13.5698093 | 3.91E-39 | 3.50E-38 | 77.58468533 |
| VPS37C | 0.729453366 | 3.826347978 | 13.56787721 | 4.00E-39 | 3.58E-38 | 77.56195539 |
| ETV5 | -1.004818607 | 2.13812414 | -13.56707878 | 4.04E-39 | 3.61E-38 | 77.55256303 |
| SRRT | 0.620460382 | 4.590757484 | 13.56663355 | 4.06E-39 | 3.63E-38 | 77.54732577 |
| ADAM19 | 0.969963263 | 1.835525071 | 13.56394391 | 4.19E-39 | 3.75E-38 | 77.5156897 |
| FBXO46 | 0.704413597 | 3.222403676 | 13.56037579 | 4.37E-39 | 3.91E-38 | 77.4737279 |
| CRTAC1 | -1.061634727 | 0.706958329 | -13.5595483 | 4.42E-39 | 3.94E-38 | 77.46399764 |
| TOMM34 | 0.735012187 | 4.542139952 | 13.55613099 | 4.60E-39 | 4.10E-38 | 77.42381882 |
| N4BP3 | 1.184651695 | 2.53987648 | 13.55539659 | 4.64E-39 | 4.14E-38 | 77.41518518 |
| E2F2 | 0.970006663 | 1.304717636 | 13.54565508 | 5.21E-39 | 4.64E-38 | 77.30069537 |
| PARPBP | 0.675463141 | 1.250326509 | 13.5392587 | 5.61E-39 | 5.00E-38 | 77.22555309 |
| DBF4 | 0.73887138 | 2.017735245 | 13.53914211 | 5.62E-39 | 5.01E-38 | 77.22418369 |
| C16orf91 | 0.813281135 | 3.693034604 | 13.53696256 | 5.77E-39 | 5.13E-38 | 77.19858533 |
| S100A14 | 2.436996837 | 6.486213185 | 13.53279691 | 6.06E-39 | 5.39E-38 | 77.14966888 |
| USP5 | 0.68334891 | 4.886140166 | 13.52603949 | 6.56E-39 | 5.84E-38 | 77.07034141 |
| MATN3 | 1.650914043 | 2.63129012 | 13.52319754 | 6.79E-39 | 6.03E-38 | 77.03698763 |
| ATG4D | 0.69786307 | 2.944461798 | 13.52308132 | 6.80E-39 | 6.03E-38 | 77.0356237 |
| PRRG1 | -0.760144148 | 1.311903106 | -13.51998861 | 7.05E-39 | 6.26E-38 | 76.99933302 |
| RNFT2 | 0.662784174 | 0.938405206 | 13.51813399 | 7.20E-39 | 6.39E-38 | 76.97757331 |
| ABHD17C | 1.044600144 | 3.08083733 | 13.51567746 | 7.42E-39 | 6.57E-38 | 76.94875501 |
| SULT1C4 | -0.764257709 | 0.736978293 | -13.51233213 | 7.71E-39 | 6.83E-38 | 76.90951606 |
| MGAM2 | -0.876926724 | 0.349401335 | -13.50803285 | 8.12E-39 | 7.19E-38 | 76.85909829 |
| EHHADH | -0.784271217 | 1.766810597 | -13.50721978 | 8.19E-39 | 7.25E-38 | 76.84956472 |
| BBS2 | -0.610558956 | 2.430576135 | -13.50552288 | 8.36E-39 | 7.40E-38 | 76.82966931 |
| HSD17B10 | 0.786605654 | 6.070419332 | 13.50161443 | 8.75E-39 | 7.74E-38 | 76.78385124 |
| TUBB4B | 0.852852126 | 6.999213433 | 13.49987511 | 8.94E-39 | 7.90E-38 | 76.76346474 |
| MIF | 1.4374176 | 4.760790596 | 13.48531181 | 1.06E-38 | 9.37E-38 | 76.59284429 |
| LMAN2 | 0.785662006 | 6.143240871 | 13.48525278 | 1.06E-38 | 9.37E-38 | 76.59215309 |
| CABLES2 | 0.826264501 | 2.444400402 | 13.48226624 | 1.10E-38 | 9.70E-38 | 76.55718033 |
| POLA2 | 0.638320917 | 2.371325231 | 13.48013027 | 1.13E-38 | 9.93E-38 | 76.53217129 |
| TRIM29 | -1.992765826 | 2.082338503 | -13.47738886 | 1.16E-38 | 1.03E-37 | 76.50007785 |
| ADM | -1.481159467 | 2.118015468 | -13.47310814 | 1.22E-38 | 1.08E-37 | 76.44997341 |
| ALDOA | 1.005096251 | 7.736925034 | 13.46766861 | 1.31E-38 | 1.15E-37 | 76.38632236 |
| OTUB1 | 0.618212483 | 4.131936462 | 13.46395849 | 1.36E-38 | 1.20E-37 | 76.34291906 |
| NT5E | -1.087058558 | 2.295803499 | -13.46064861 | 1.42E-38 | 1.25E-37 | 76.30420543 |
| TFB2M | 0.699162915 | 3.868179293 | 13.45214101 | 1.57E-38 | 1.38E-37 | 76.20472951 |
| KDM5B | 0.836841889 | 3.896001028 | 13.44718299 | 1.66E-38 | 1.46E-37 | 76.1467789 |
| FZD5 | -0.670969768 | 1.124749962 | -13.44705257 | 1.66E-38 | 1.46E-37 | 76.1452547 |
| ADSS | 0.758238734 | 4.783693181 | 13.44223945 | 1.76E-38 | 1.54E-37 | 76.08901309 |
| CTTNBP2 | -0.774654543 | 0.653583759 | -13.43187087 | 1.99E-38 | 1.74E-37 | 75.96790622 |
| FAM173A | 1.190807575 | 2.7038317 | 13.43088694 | 2.01E-38 | 1.76E-37 | 75.9564173 |
| PI15 | -2.26612931 | 1.681866828 | -13.42055523 | 2.27E-38 | 1.99E-37 | 75.83581633 |
| TNFSF4 | 0.864590411 | 1.393664457 | 13.42047725 | 2.27E-38 | 1.99E-37 | 75.83490628 |
| RAB40C | 0.829262292 | 3.278771155 | 13.4178139 | 2.35E-38 | 2.05E-37 | 75.80382856 |
| BRMS1 | 0.844762137 | 4.299645449 | 13.41749734 | 2.35E-38 | 2.06E-37 | 75.800135 |
| FAM172A | -0.684176719 | 2.667355379 | -13.41185398 | 2.52E-38 | 2.20E-37 | 75.73430068 |
| CCDC137 | 0.882579562 | 3.261549691 | 13.40909303 | 2.60E-38 | 2.27E-37 | 75.70209947 |
| SFXN1 | 0.758396858 | 2.72034859 | 13.40618034 | 2.69E-38 | 2.35E-37 | 75.66813381 |
| ATP6V1F | 0.721397271 | 6.722313367 | 13.4028568 | 2.80E-38 | 2.44E-37 | 75.62938371 |
| KIFC2 | 1.231060223 | 2.28347849 | 13.40227057 | 2.81E-38 | 2.45E-37 | 75.62254949 |
| IFI6 | 2.280771254 | 7.566125944 | 13.39904971 | 2.92E-38 | 2.55E-37 | 75.58500466 |
| ORC6 | 0.944530017 | 1.271320349 | 13.39834245 | 2.95E-38 | 2.57E-37 | 75.57676115 |
| RRP36 | 0.587981686 | 4.381101419 | 13.39591569 | 3.03E-38 | 2.64E-37 | 75.54847842 |
| CSDE1 | -0.647409845 | 6.863365796 | -13.39525469 | 3.06E-38 | 2.66E-37 | 75.54077543 |
| RASD1 | -1.881895361 | 3.24633388 | -13.39167944 | 3.19E-38 | 2.77E-37 | 75.49911612 |
| GGPS1 | 0.677206924 | 4.246599494 | 13.39103002 | 3.21E-38 | 2.79E-37 | 75.49154988 |
| IL4I1 | 1.364995027 | 1.955745953 | 13.39044663 | 3.23E-38 | 2.81E-37 | 75.48475309 |
| CASP3 | 0.591031327 | 3.847948246 | 13.37788243 | 3.75E-38 | 3.25E-37 | 75.33842843 |
| UBE2M | 0.722461823 | 5.008432696 | 13.37425188 | 3.91E-38 | 3.39E-37 | 75.29616538 |
| FXYD6 | -0.983605755 | 1.89625557 | -13.36744606 | 4.23E-38 | 3.67E-37 | 75.2169621 |
| BANF1 | 0.774841268 | 6.117101381 | 13.35985078 | 4.63E-38 | 4.01E-37 | 75.12860669 |
| SCT | 1.063343144 | 1.111651106 | 13.35623535 | 4.83E-38 | 4.18E-37 | 75.08656175 |
| CCNE2 | 0.945226764 | 1.337362862 | 13.35474099 | 4.91E-38 | 4.25E-37 | 75.06918583 |
| FRMD4A | -0.674011338 | 1.629988451 | -13.3528754 | 5.02E-38 | 4.34E-37 | 75.04749542 |
| RABEP2 | 0.959611922 | 2.940187484 | 13.34920831 | 5.24E-38 | 4.53E-37 | 75.00486629 |
| CAMSAP3 | 1.013718951 | 3.076173438 | 13.34512032 | 5.50E-38 | 4.75E-37 | 74.9573544 |
| FMNL2 | -1.073846389 | 2.540438923 | -13.34416769 | 5.56E-38 | 4.80E-37 | 74.94628427 |
| HSPA5 | 0.707850785 | 7.548677964 | 13.34129485 | 5.75E-38 | 4.96E-37 | 74.91290348 |
| DENND5A | -0.641011458 | 3.243519189 | -13.33748731 | 6.01E-38 | 5.18E-37 | 74.86867031 |
| PRPF19 | 0.649307986 | 5.986568636 | 13.33586924 | 6.12E-38 | 5.28E-37 | 74.8498755 |
| ZNF662 | -0.88582874 | 1.205353192 | -13.33332977 | 6.31E-38 | 5.43E-37 | 74.82038164 |
| VPS36 | -0.665046682 | 3.295452937 | -13.33007159 | 6.55E-38 | 5.64E-37 | 74.78254672 |
| KDF1 | 0.896495242 | 3.628674001 | 13.32344179 | 7.08E-38 | 6.09E-37 | 74.70558074 |
| ATP6V1G1 | 0.734188328 | 6.756272209 | 13.32250354 | 7.16E-38 | 6.15E-37 | 74.69469078 |
| THOC3 | 0.620824057 | 1.895227408 | 13.32088129 | 7.29E-38 | 6.27E-37 | 74.67586323 |
| ARL8A | 0.618780588 | 5.281287421 | 13.31531008 | 7.78E-38 | 6.68E-37 | 74.61121763 |
| GIPC1 | 0.902737356 | 4.458846263 | 13.30454055 | 8.83E-38 | 7.58E-37 | 74.4863104 |
| AAGAB | 0.70563748 | 4.380809112 | 13.30434669 | 8.85E-38 | 7.59E-37 | 74.48406266 |
| NREP | 0.937193099 | 2.945124425 | 13.3033757 | 8.95E-38 | 7.67E-37 | 74.47280472 |
| POGK | 0.711419537 | 3.868344266 | 13.30295588 | 8.99E-38 | 7.71E-37 | 74.4679374 |
| MAP3K3 | -0.621467734 | 2.910080188 | -13.29903818 | 9.41E-38 | 8.06E-37 | 74.42252178 |
| SSR2 | 0.695726931 | 5.644436326 | 13.292767 | 1.01E-37 | 8.67E-37 | 74.34984423 |
| DAXX | 0.587575869 | 4.670657623 | 13.29125168 | 1.03E-37 | 8.82E-37 | 74.33228686 |
| CHAF1A | 0.784891382 | 2.682031993 | 13.28583584 | 1.10E-37 | 9.39E-37 | 74.26954774 |
| TXN | 0.840193159 | 7.084419999 | 13.28485158 | 1.11E-37 | 9.50E-37 | 74.25814781 |
| ELOC | 0.760896344 | 3.473684022 | 13.28328177 | 1.13E-37 | 9.67E-37 | 74.23996716 |
| RNF145 | -1.115979006 | 3.292349891 | -13.28241507 | 1.14E-37 | 9.76E-37 | 74.2299302 |
| RIBC2 | 0.834728618 | 1.450235705 | 13.28114225 | 1.16E-37 | 9.90E-37 | 74.21519096 |
| MAN2A2 | -0.708996303 | 2.489606377 | -13.28101157 | 1.16E-37 | 9.91E-37 | 74.21367778 |
| ANAPC11 | 0.957074695 | 3.929334203 | 13.27716134 | 1.21E-37 | 1.04E-36 | 74.16909891 |
| DHRS3 | -1.056348946 | 3.997501247 | -13.27168421 | 1.29E-37 | 1.10E-36 | 74.10570007 |
| ORC1 | 0.957603764 | 1.418034396 | 13.26943319 | 1.33E-37 | 1.13E-36 | 74.07964971 |
| TMCC3 | -0.831598383 | 1.529030828 | -13.26389489 | 1.42E-37 | 1.21E-36 | 74.01557061 |
| NFKBIE | 0.897112225 | 3.025509775 | 13.2638651 | 1.42E-37 | 1.21E-36 | 74.01522601 |
| CYP39A1 | -1.042763478 | 0.978902788 | -13.2578995 | 1.52E-37 | 1.29E-36 | 73.94622548 |
| KRT5 | -3.244727619 | 3.909696731 | -13.25106604 | 1.65E-37 | 1.40E-36 | 73.86721529 |
| XRCC2 | 0.766864024 | 1.257066794 | 13.24983285 | 1.67E-37 | 1.42E-36 | 73.85296002 |
| HIST1H3D | 1.632842167 | 1.961371426 | 13.24394044 | 1.79E-37 | 1.52E-36 | 73.78485965 |
| CTDSP2 | -0.682304816 | 5.4907748 | -13.24168762 | 1.83E-37 | 1.56E-36 | 73.75882908 |
| ZBED3 | -0.693225361 | 2.073858129 | -13.23989828 | 1.87E-37 | 1.59E-36 | 73.73815618 |
| RFC4 | 0.876164096 | 2.960145795 | 13.23944339 | 1.88E-37 | 1.60E-36 | 73.73290099 |
| DNAJC9 | 0.655322238 | 2.610562462 | 13.23806792 | 1.91E-37 | 1.62E-36 | 73.71701154 |
| BLM | 0.777896444 | 1.225228886 | 13.23804166 | 1.91E-37 | 1.62E-36 | 73.7167082 |
| PTX3 | -1.529907035 | 1.136555388 | -13.23684451 | 1.94E-37 | 1.64E-36 | 73.7028797 |
| SAP30L | -0.605171654 | 3.049387295 | -13.23650024 | 1.95E-37 | 1.65E-36 | 73.69890316 |
| RNF217 | -0.651922687 | 1.158737099 | -13.2347013 | 1.99E-37 | 1.68E-36 | 73.67812539 |
| XG | -0.98740937 | 1.365915489 | -13.23103662 | 2.08E-37 | 1.75E-36 | 73.63580496 |
| MORF4L2 | 0.751551472 | 6.859988028 | 13.22493645 | 2.23E-37 | 1.88E-36 | 73.56537844 |
| ARPC1B | 1.014292623 | 4.958849714 | 13.21984614 | 2.36E-37 | 1.99E-36 | 73.50662924 |
| COLGALT1 | 0.695419332 | 4.725930886 | 13.2193133 | 2.38E-37 | 2.01E-36 | 73.5004805 |
| ADAMTS18 | -0.676049281 | 0.436366035 | -13.21414815 | 2.53E-37 | 2.13E-36 | 73.44088654 |
| OVOL2 | 0.881400132 | 2.782756571 | 13.2133889 | 2.55E-37 | 2.15E-36 | 73.43212808 |
| STYXL1 | 0.720021544 | 4.130488537 | 13.21093295 | 2.62E-37 | 2.21E-36 | 73.40379938 |
| PIGQ | 0.956327802 | 3.458506859 | 13.21033408 | 2.64E-37 | 2.22E-36 | 73.3968922 |
| CCL11 | 0.903928616 | 0.925969235 | 13.20935896 | 2.67E-37 | 2.25E-36 | 73.38564594 |
| MYADM | -0.953733328 | 5.832475134 | -13.20808769 | 2.71E-37 | 2.28E-36 | 73.37098499 |
| ABRACL | 1.126468794 | 4.838788125 | 13.20767203 | 2.72E-37 | 2.29E-36 | 73.36619164 |
| BCL3 | 0.945883914 | 4.244983281 | 13.20555514 | 2.79E-37 | 2.35E-36 | 73.34178164 |
| C7orf43 | 0.67340841 | 2.701680536 | 13.20525869 | 2.80E-37 | 2.35E-36 | 73.33836346 |
| MGAT4B | 0.649396176 | 4.14007911 | 13.20198235 | 2.91E-37 | 2.44E-36 | 73.30059 |
| NRSN2 | 0.843362681 | 4.193101417 | 13.2017257 | 2.92E-37 | 2.45E-36 | 73.29763137 |
| RTKN2 | 0.626255484 | 0.916378768 | 13.19511901 | 3.15E-37 | 2.64E-36 | 73.2214842 |
| NAA20 | 0.669366962 | 4.95477579 | 13.19148654 | 3.29E-37 | 2.75E-36 | 73.17962937 |
| CST1 | 2.534840847 | 2.432516585 | 13.1829871 | 3.63E-37 | 3.03E-36 | 73.08172885 |
| CDC7 | 0.982798634 | 1.966870897 | 13.1763817 | 3.91E-37 | 3.27E-36 | 73.00567741 |
| C1orf115 | -1.469459483 | 3.708542438 | -13.16876054 | 4.27E-37 | 3.57E-36 | 72.9179664 |
| CLEC5A | 0.968666559 | 1.121873132 | 13.16870811 | 4.28E-37 | 3.58E-36 | 72.91736307 |
| DPCD | 0.822565328 | 3.472135896 | 13.16854265 | 4.29E-37 | 3.58E-36 | 72.91545927 |
| CAPZA1 | 0.586025485 | 5.411730476 | 13.16672549 | 4.38E-37 | 3.66E-36 | 72.89455159 |
| CIB1 | 0.921181173 | 6.271717629 | 13.16513698 | 4.46E-37 | 3.72E-36 | 72.87627653 |
| UBL5 | 0.727316052 | 5.709114318 | 13.1619845 | 4.62E-37 | 3.86E-36 | 72.84001356 |
| BDH2 | -0.802032881 | 2.9289256 | -13.16128562 | 4.66E-37 | 3.89E-36 | 72.83197514 |
| GLRX2 | 0.687668465 | 2.954969203 | 13.16070966 | 4.69E-37 | 3.91E-36 | 72.82535085 |
| PIP4P2 | -0.833933314 | 1.943595493 | -13.16037932 | 4.71E-37 | 3.92E-36 | 72.82155168 |
| SESN1 | -0.86046825 | 2.763141447 | -13.15984346 | 4.74E-37 | 3.95E-36 | 72.8153888 |
| ADRA2A | -1.460967177 | 2.546049308 | -13.15890559 | 4.79E-37 | 3.99E-36 | 72.80460309 |
| SIX4 | 0.990597203 | 1.972285816 | 13.15764021 | 4.86E-37 | 4.04E-36 | 72.79005184 |
| CIP2A | 0.904169878 | 1.618896698 | 13.15763334 | 4.86E-37 | 4.04E-36 | 72.78997277 |
| ENTPD7 | 0.812642284 | 2.398494347 | 13.15370499 | 5.09E-37 | 4.23E-36 | 72.7448052 |
| PPIA | 0.614966241 | 6.365222396 | 13.14946637 | 5.34E-37 | 4.44E-36 | 72.69608163 |
| NES | -1.302738391 | 3.748674766 | -13.13905161 | 6.03E-37 | 5.00E-36 | 72.57641204 |
| LUZP1 | -0.6572822 | 2.449932884 | -13.13731405 | 6.15E-37 | 5.10E-36 | 72.55645379 |
| ACTG1 | 0.647138196 | 10.19362303 | 13.1368933 | 6.18E-37 | 5.12E-36 | 72.55162113 |
| FZD7 | -1.325216775 | 3.641674259 | -13.12936712 | 6.74E-37 | 5.58E-36 | 72.46519735 |
| HOXA10 | -0.87192984 | 0.968904388 | -13.10971664 | 8.46E-37 | 6.99E-36 | 72.23972396 |
| BCL2L12 | 0.898665148 | 2.858153145 | 13.10850424 | 8.58E-37 | 7.09E-36 | 72.2258209 |
| LPP | -0.844117255 | 2.883671926 | -13.1058115 | 8.85E-37 | 7.31E-36 | 72.19494585 |
| MFSD4A | -0.796394451 | 0.777938177 | -13.09990458 | 9.47E-37 | 7.82E-36 | 72.12723327 |
| RBPMS | -1.000327885 | 3.014433589 | -13.09610351 | 9.90E-37 | 8.16E-36 | 72.0836728 |
| PFDN2 | 1.008298626 | 5.909699075 | 13.09554066 | 9.96E-37 | 8.21E-36 | 72.07722327 |
| FCGR1A | 0.781523535 | 1.348020104 | 13.09391146 | 1.01E-36 | 8.36E-36 | 72.05855603 |
| SPINDOC | 0.770160578 | 3.063703798 | 13.09208384 | 1.04E-36 | 8.54E-36 | 72.03761735 |
| TRAF4 | 1.001016051 | 4.254926534 | 13.09159166 | 1.04E-36 | 8.58E-36 | 72.031979 |
| TLE3 | 0.805103289 | 3.51775111 | 13.08724098 | 1.10E-36 | 9.02E-36 | 71.9821444 |
| LDHD | -1.209221318 | 1.788885477 | -13.08632081 | 1.11E-36 | 9.11E-36 | 71.97160598 |
| SBF2 | -0.730602557 | 1.787577035 | -13.08539492 | 1.12E-36 | 9.20E-36 | 71.96100259 |
| RALY | 0.641328247 | 4.410138867 | 13.08048138 | 1.18E-36 | 9.72E-36 | 71.90474176 |
| SNX21 | -0.700423376 | 2.361937474 | -13.08004359 | 1.19E-36 | 9.77E-36 | 71.89972971 |
| NAV2 | -1.157878888 | 1.976862963 | -13.07619801 | 1.24E-36 | 1.02E-35 | 71.8557094 |
| HMG20B | 0.886214102 | 4.647873121 | 13.07053247 | 1.33E-36 | 1.09E-35 | 71.79087356 |
| TM4SF18 | -1.27348474 | 2.542126946 | -13.06925647 | 1.35E-36 | 1.10E-35 | 71.77627415 |
| ESRP2 | 0.951424453 | 3.327292247 | 13.06398614 | 1.43E-36 | 1.17E-35 | 71.7159844 |
| ENSA | 0.683413677 | 5.612496475 | 13.06331608 | 1.44E-36 | 1.18E-35 | 71.70832057 |
| BOP1 | 1.166526673 | 3.847140271 | 13.05880226 | 1.52E-36 | 1.24E-35 | 71.65670155 |
| GBA | 0.748254345 | 4.220010877 | 13.04671756 | 1.75E-36 | 1.43E-35 | 71.51856943 |
| DKK3 | -1.26851465 | 3.490878545 | -13.03766665 | 1.94E-36 | 1.58E-35 | 71.41517748 |
| SRMS | 1.227709123 | 1.878680397 | 13.03676557 | 1.96E-36 | 1.60E-35 | 71.40488709 |
| LCMT1 | 0.669910076 | 4.090267188 | 13.03206089 | 2.07E-36 | 1.69E-35 | 71.35116788 |
| RPN2 | 0.730961723 | 7.297422066 | 13.03104926 | 2.09E-36 | 1.70E-35 | 71.33961877 |
| PC | -1.034626315 | 2.885103739 | -13.03067372 | 2.10E-36 | 1.71E-35 | 71.3353316 |
| C9orf116 | 1.184591966 | 2.229640806 | 13.02750544 | 2.18E-36 | 1.77E-35 | 71.29916651 |
| HOXC13 | 1.482103517 | 2.016690046 | 13.02642672 | 2.21E-36 | 1.79E-35 | 71.28685471 |
| SUN2 | -0.879624353 | 4.477180103 | -13.02635035 | 2.21E-36 | 1.80E-35 | 71.28598308 |
| TMEM14A | 0.748675373 | 4.891453707 | 13.02380478 | 2.27E-36 | 1.85E-35 | 71.25693286 |
| PCDH17 | 0.610459667 | 1.18850737 | 13.01895016 | 2.40E-36 | 1.95E-35 | 71.20154329 |
| C8orf76 | 0.657942008 | 2.083868075 | 13.0153473 | 2.51E-36 | 2.03E-35 | 71.16044593 |
| DIAPH3 | 0.820330323 | 1.164501778 | 13.01478403 | 2.52E-36 | 2.05E-35 | 71.15402165 |
| ETV1 | -0.762128191 | 1.241420281 | -13.01324784 | 2.57E-36 | 2.08E-35 | 71.13650173 |
| FBXO6 | 0.814104689 | 3.32277396 | 13.00144427 | 2.94E-36 | 2.38E-35 | 71.00193627 |
| NCOA7 | -1.205329058 | 2.711637022 | -13.00087754 | 2.96E-36 | 2.39E-35 | 70.99547764 |
| FOXO6 | 0.878453602 | 1.695529225 | 12.9936788 | 3.21E-36 | 2.60E-35 | 70.91345701 |
| DRD2 | -0.674769865 | 0.336339795 | -12.98934079 | 3.38E-36 | 2.73E-35 | 70.86404732 |
| NUBP2 | 0.873844589 | 3.471748873 | 12.98724787 | 3.46E-36 | 2.80E-35 | 70.8402136 |
| CTPS1 | 0.909246193 | 2.749358413 | 12.98667591 | 3.48E-36 | 2.81E-35 | 70.83370069 |
| MAP3K5 | -0.8984029 | 2.369716782 | -12.9856619 | 3.52E-36 | 2.84E-35 | 70.82215481 |
| SNRPD1 | 0.671663462 | 3.47404798 | 12.98556122 | 3.52E-36 | 2.85E-35 | 70.82100842 |
| FNIP2 | -0.742211708 | 1.996006679 | -12.98053171 | 3.73E-36 | 3.01E-35 | 70.76375076 |
| PPP1R35 | 0.981982597 | 3.586626294 | 12.97957536 | 3.77E-36 | 3.05E-35 | 70.75286523 |
| TLR3 | -0.77905801 | 1.439319551 | -12.97438041 | 4.01E-36 | 3.23E-35 | 70.69374504 |
| ZBTB47 | -0.698140396 | 2.567103397 | -12.96947222 | 4.24E-36 | 3.42E-35 | 70.63790459 |
| MRPL55 | 0.913409668 | 4.202462587 | 12.96195803 | 4.62E-36 | 3.72E-35 | 70.55244654 |
| SYNE4 | 1.069036233 | 2.909670064 | 12.95595394 | 4.95E-36 | 3.97E-35 | 70.48418964 |
| C11orf24 | 0.641567216 | 3.892292449 | 12.95382785 | 5.07E-36 | 4.07E-35 | 70.46002515 |
| METTL1 | 0.649839167 | 3.098139132 | 12.9501453 | 5.29E-36 | 4.24E-35 | 70.41817745 |
| EPB41L4B | -0.924966397 | 2.25134725 | -12.94857927 | 5.38E-36 | 4.32E-35 | 70.40038407 |
| PEAK1 | -0.62759101 | 1.663756305 | -12.94734106 | 5.46E-36 | 4.38E-35 | 70.38631668 |
| NCLN | 0.692779587 | 4.079266501 | 12.94656236 | 5.51E-36 | 4.41E-35 | 70.37747023 |
| ADGRA3 | -0.80427791 | 2.161795073 | -12.94605211 | 5.54E-36 | 4.44E-35 | 70.37167381 |
| SELENOH | 0.799419495 | 4.671771375 | 12.94371099 | 5.69E-36 | 4.56E-35 | 70.34508079 |
| CWF19L2 | -0.600803846 | 2.663612806 | -12.9367129 | 6.16E-36 | 4.93E-35 | 70.26561063 |
| ARHGAP21 | -0.828832761 | 2.975167113 | -12.93455183 | 6.32E-36 | 5.05E-35 | 70.2410761 |
| TNFRSF18 | 1.508364302 | 2.273946889 | 12.92809051 | 6.80E-36 | 5.44E-35 | 70.16773944 |
| MAGEF1 | 0.767289666 | 5.152273193 | 12.92726029 | 6.87E-36 | 5.49E-35 | 70.15831846 |
| SHB | 0.75706051 | 2.815863201 | 12.92642706 | 6.93E-36 | 5.54E-35 | 70.14886366 |
| CITED1 | -1.60255688 | 1.208088431 | -12.92584432 | 6.98E-36 | 5.57E-35 | 70.14225151 |
| RERE | -0.646891561 | 3.755792341 | -12.9214359 | 7.34E-36 | 5.85E-35 | 70.09223802 |
| ADAMTSL2 | 0.819903886 | 1.456114635 | 12.92077048 | 7.39E-36 | 5.90E-35 | 70.08468995 |
| EIF6 | 0.732463579 | 6.048452564 | 12.91968507 | 7.49E-36 | 5.96E-35 | 70.07237845 |
| SYNE2 | -0.804690826 | 2.463845484 | -12.91933963 | 7.52E-36 | 5.99E-35 | 70.06846033 |
| ITPA | 0.755468965 | 4.340398056 | 12.91831394 | 7.60E-36 | 6.05E-35 | 70.05682719 |
| ZDHHC17 | -0.598620552 | 2.21934135 | -12.91492332 | 7.90E-36 | 6.29E-35 | 70.01837642 |
| DMTN | -1.166872467 | 2.417939598 | -12.91246163 | 8.13E-36 | 6.46E-35 | 69.9904648 |
| SGCE | -1.386793893 | 3.507565465 | -12.91231408 | 8.14E-36 | 6.47E-35 | 69.98879193 |
| FAM189B | 0.808385304 | 3.454991392 | 12.91162696 | 8.21E-36 | 6.52E-35 | 69.98100189 |
| CPNE2 | -0.671602672 | 2.164315931 | -12.90875757 | 8.48E-36 | 6.73E-35 | 69.94847432 |
| SETD7 | -0.838486825 | 3.245474713 | -12.90703193 | 8.65E-36 | 6.86E-35 | 69.92891494 |
| PAXX | 1.060227015 | 4.206561145 | 12.90078518 | 9.29E-36 | 7.37E-35 | 69.85812736 |
| TMEM238 | 1.176740261 | 2.213225915 | 12.89796916 | 9.59E-36 | 7.60E-35 | 69.82622506 |
| KDELR2 | 0.634095344 | 6.304457229 | 12.89766643 | 9.62E-36 | 7.63E-35 | 69.82279573 |
| CREB3L1 | 1.60731171 | 3.696448541 | 12.89691076 | 9.71E-36 | 7.69E-35 | 69.81423592 |
| AJM1 | 0.699260205 | 1.032292411 | 12.88516209 | 1.11E-35 | 8.78E-35 | 69.68120176 |
| BARD1 | 0.711446728 | 1.906309776 | 12.88443247 | 1.12E-35 | 8.85E-35 | 69.67294299 |
| WDHD1 | 0.705990941 | 1.766559711 | 12.88336267 | 1.13E-35 | 8.96E-35 | 69.66083437 |
| SELENOI | 0.775079884 | 3.118757737 | 12.87464354 | 1.25E-35 | 9.89E-35 | 69.56217453 |
| CD9 | 0.899964071 | 6.802062455 | 12.86845337 | 1.34E-35 | 1.06E-34 | 69.49216135 |
| VDAC1 | 0.592024633 | 6.081390264 | 12.86800363 | 1.35E-35 | 1.07E-34 | 69.48707567 |
| SH3BP5L | 0.601391658 | 3.179216894 | 12.85532863 | 1.56E-35 | 1.23E-34 | 69.3438 |
| PLCH2 | -0.732929055 | 0.766393661 | -12.85446348 | 1.57E-35 | 1.24E-34 | 69.33402446 |
| PARM1 | -1.280293004 | 2.759572928 | -12.85398134 | 1.58E-35 | 1.25E-34 | 69.32857682 |
| TSPAN11 | -0.637108076 | 0.800251606 | -12.85020357 | 1.65E-35 | 1.30E-34 | 69.2858978 |
| CLN3 | 0.785049542 | 3.135336545 | 12.84943007 | 1.67E-35 | 1.31E-34 | 69.27716048 |
| GAPDH | 0.962380396 | 9.28841028 | 12.84926201 | 1.67E-35 | 1.32E-34 | 69.27526209 |
| MMP9 | 2.227464276 | 4.25635364 | 12.84820711 | 1.69E-35 | 1.33E-34 | 69.2633468 |
| CDKN2D | 0.891370331 | 2.842213606 | 12.84780903 | 1.70E-35 | 1.34E-34 | 69.25885067 |
| KRTCAP2 | 0.715564205 | 2.759513529 | 12.84495703 | 1.75E-35 | 1.38E-34 | 69.22664128 |
| KCTD9 | -0.792356345 | 2.938152466 | -12.84413812 | 1.77E-35 | 1.39E-34 | 69.21739384 |
| CAPN6 | -1.719339568 | 1.211059072 | -12.83519319 | 1.96E-35 | 1.54E-34 | 69.11641336 |
| FAHD1 | 0.672199531 | 3.550999632 | 12.83292291 | 2.01E-35 | 1.58E-34 | 69.09079242 |
| SIAH2 | 1.274652136 | 4.984755145 | 12.8313676 | 2.05E-35 | 1.61E-34 | 69.07324205 |
| ZNF471 | -0.733443097 | 1.068723028 | -12.82911038 | 2.10E-35 | 1.65E-34 | 69.04777424 |
| WDR24 | 0.715800497 | 2.435035599 | 12.82471958 | 2.21E-35 | 1.73E-34 | 68.99824334 |
| RHBDD3 | 0.790437911 | 2.973749583 | 12.82369953 | 2.23E-35 | 1.75E-34 | 68.98673838 |
| DHX34 | 0.633013711 | 2.639020351 | 12.82284486 | 2.25E-35 | 1.76E-34 | 68.97709917 |
| FOXN2 | -0.741688244 | 1.956236649 | -12.82159975 | 2.29E-35 | 1.79E-34 | 68.96305755 |
| MRPS23 | 0.724586433 | 3.166393398 | 12.82039401 | 2.32E-35 | 1.81E-34 | 68.94946073 |
| IGSF8 | 0.932760026 | 5.080926017 | 12.81804475 | 2.38E-35 | 1.86E-34 | 68.92297172 |
| EDA2R | -0.726831093 | 1.107990648 | -12.81287881 | 2.52E-35 | 1.97E-34 | 68.86473623 |
| CCNG1 | -0.778273387 | 4.79709028 | -12.81050531 | 2.59E-35 | 2.02E-34 | 68.83798587 |
| TMEM97 | 1.134971008 | 3.515170046 | 12.80829127 | 2.66E-35 | 2.08E-34 | 68.81303605 |
| NR2C2AP | 0.782606627 | 3.893687943 | 12.8030608 | 2.82E-35 | 2.20E-34 | 68.75410725 |
| NEU1 | 0.673050276 | 4.367575058 | 12.80071516 | 2.90E-35 | 2.26E-34 | 68.72768618 |
| NDUFAF6 | 0.790179066 | 2.379780008 | 12.7953714 | 3.08E-35 | 2.40E-34 | 68.66750841 |
| MAGED1 | 0.91870357 | 5.467713725 | 12.79520767 | 3.08E-35 | 2.40E-34 | 68.66566486 |
| CPAMD8 | -0.982851909 | 1.05985651 | -12.79046201 | 3.25E-35 | 2.53E-34 | 68.61223897 |
| SLC25A25 | -0.883987922 | 2.585449563 | -12.78952335 | 3.29E-35 | 2.56E-34 | 68.60167346 |
| NPR3 | -1.18894186 | 1.110628551 | -12.78861143 | 3.32E-35 | 2.59E-34 | 68.59140957 |
| DSEL | -0.748418555 | 0.956467114 | -12.78366217 | 3.51E-35 | 2.73E-34 | 68.53571369 |
| TCN2 | -0.9493919 | 3.069560048 | -12.78365001 | 3.51E-35 | 2.73E-34 | 68.53557686 |
| FOXP3 | 0.954536948 | 1.480648282 | 12.78321328 | 3.53E-35 | 2.74E-34 | 68.53066299 |
| MAPK13 | 0.782099535 | 3.823897996 | 12.77535577 | 3.86E-35 | 3.00E-34 | 68.44227567 |
| CAPN15 | 0.702437306 | 2.470776074 | 12.76726881 | 4.23E-35 | 3.28E-34 | 68.3513505 |
| MAP2K6 | -0.657131297 | 1.05700431 | -12.76217284 | 4.48E-35 | 3.48E-34 | 68.29407673 |
| HYLS1 | 0.659671439 | 2.062471617 | 12.75545904 | 4.83E-35 | 3.75E-34 | 68.21864681 |
| NSF | 0.721347455 | 3.659981119 | 12.75520502 | 4.85E-35 | 3.76E-34 | 68.21579345 |
| SSNA1 | 0.864796967 | 5.21680283 | 12.75383867 | 4.92E-35 | 3.82E-34 | 68.2004464 |
| METRN | 1.580008869 | 3.356723066 | 12.75370517 | 4.93E-35 | 3.82E-34 | 68.19894693 |
| ARHGEF4 | -0.61995448 | 0.630274351 | -12.75283468 | 4.98E-35 | 3.86E-34 | 68.18917016 |
| POSTN | 1.861833868 | 7.075671852 | 12.75187116 | 5.03E-35 | 3.90E-34 | 68.17834913 |
| PLP1 | -0.930732743 | 0.494589373 | -12.74639119 | 5.36E-35 | 4.14E-34 | 68.11681693 |
| LEF1 | 1.175712017 | 2.20832363 | 12.74631412 | 5.36E-35 | 4.15E-34 | 68.11595168 |
| MFGE8 | -1.472856075 | 4.256948933 | -12.74257404 | 5.59E-35 | 4.32E-34 | 68.07396776 |
| FGD5 | -0.96435528 | 2.155956994 | -12.7316454 | 6.33E-35 | 4.89E-34 | 67.95134298 |
| PGAP1 | -0.619413633 | 1.045867276 | -12.72952325 | 6.48E-35 | 5.00E-34 | 67.92754069 |
| LIMCH1 | -1.196156119 | 2.906062867 | -12.7206768 | 7.16E-35 | 5.53E-34 | 67.82835053 |
| DPP9 | 0.627549564 | 3.233513616 | 12.71926847 | 7.28E-35 | 5.61E-34 | 67.81256448 |
| IKBKE | 0.844503323 | 2.435177901 | 12.71875857 | 7.32E-35 | 5.64E-34 | 67.80684942 |
| FSTL1 | -1.252159423 | 5.282013937 | -12.71741735 | 7.43E-35 | 5.73E-34 | 67.79181735 |
| DRAP1 | 0.978690401 | 5.227311423 | 12.71428285 | 7.70E-35 | 5.93E-34 | 67.75669146 |
| CNPY3 | 0.717966475 | 5.38149408 | 12.71061675 | 8.02E-35 | 6.18E-34 | 67.71561674 |
| ZNF296 | 0.973120091 | 1.958429139 | 12.70031259 | 9.01E-35 | 6.94E-34 | 67.60021802 |
| ZNF462 | -0.924735156 | 1.310428458 | -12.69461287 | 9.61E-35 | 7.39E-34 | 67.53641622 |
| TSTD1 | 1.128411679 | 5.585596101 | 12.6937787 | 9.70E-35 | 7.45E-34 | 67.52708052 |
| CCNDBP1 | -0.639045896 | 2.811828222 | -12.69365812 | 9.71E-35 | 7.46E-34 | 67.52573104 |
| DOP1A | -0.61662113 | 1.741423679 | -12.69330776 | 9.75E-35 | 7.49E-34 | 67.52181015 |
| PACSIN1 | 0.692234182 | 0.841644362 | 12.69289408 | 9.79E-35 | 7.52E-34 | 67.51718065 |
| LOXL1 | 1.184948399 | 3.755262361 | 12.69105778 | 1.00E-34 | 7.67E-34 | 67.4966322 |
| ZFPM2 | -0.612458178 | 0.901451335 | -12.68812384 | 1.03E-34 | 7.93E-34 | 67.46380565 |
| USF1 | 0.654006837 | 4.682362254 | 12.68745355 | 1.04E-34 | 7.99E-34 | 67.45630687 |
| SIRT6 | 0.692151898 | 2.627786504 | 12.68573683 | 1.06E-34 | 8.14E-34 | 67.43710273 |
| GDI1 | 0.654649685 | 4.995346121 | 12.68447305 | 1.08E-34 | 8.25E-34 | 67.42296668 |
| SLC2A1 | 1.171504553 | 4.260920701 | 12.6811235 | 1.12E-34 | 8.56E-34 | 67.38550544 |
| ANKMY2 | -0.647178191 | 3.019133215 | -12.67870891 | 1.15E-34 | 8.80E-34 | 67.35850542 |
| FGL2 | -1.281328731 | 2.990916965 | -12.67081879 | 1.26E-34 | 9.61E-34 | 67.27030519 |
| BZW2 | 0.780081956 | 4.849449666 | 12.66837488 | 1.29E-34 | 9.87E-34 | 67.24299431 |
| TNFSF12 | -0.811200035 | 3.628959639 | -12.66413403 | 1.35E-34 | 1.03E-33 | 67.19561201 |
| CCNL1 | -0.686574906 | 3.165573427 | -12.66102148 | 1.40E-34 | 1.07E-33 | 67.16084379 |
| DOP1B | 0.912387541 | 3.172321715 | 12.66064091 | 1.41E-34 | 1.08E-33 | 67.1565931 |
| MLF2 | 0.687580555 | 5.779825341 | 12.66011515 | 1.42E-34 | 1.08E-33 | 67.15072095 |
| ATP2B4 | -0.95520567 | 4.071390372 | -12.65828254 | 1.45E-34 | 1.10E-33 | 67.13025422 |
| NOP56 | 0.628625745 | 4.407241759 | 12.65033581 | 1.58E-34 | 1.21E-33 | 67.04153081 |
| HIST1H4H | 1.990611121 | 3.390718848 | 12.64377753 | 1.70E-34 | 1.30E-33 | 66.96834128 |
| BMP5 | -0.84874724 | 0.396954868 | -12.64345689 | 1.71E-34 | 1.30E-33 | 66.96476373 |
| LRRC61 | 0.795312919 | 2.835599056 | 12.64207803 | 1.73E-34 | 1.32E-33 | 66.94937978 |
| SYNDIG1 | 1.173101759 | 1.490276389 | 12.63468927 | 1.88E-34 | 1.43E-33 | 66.86696549 |
| ZNF598 | 0.705727045 | 3.230331629 | 12.63241462 | 1.93E-34 | 1.47E-33 | 66.84160141 |
| FUS | 0.643034691 | 4.968644043 | 12.63232104 | 1.94E-34 | 1.47E-33 | 66.84055794 |
| PYM1 | 0.610967358 | 3.80065318 | 12.62599056 | 2.08E-34 | 1.58E-33 | 66.76998714 |
| RASA3 | -0.865074376 | 2.880165253 | -12.61796237 | 2.27E-34 | 1.73E-33 | 66.68052964 |
| ARHGAP42 | -0.659717998 | 1.118441836 | -12.61054174 | 2.47E-34 | 1.88E-33 | 66.59788083 |
| PSMB5 | 0.656124723 | 5.884443869 | 12.60884064 | 2.52E-34 | 1.91E-33 | 66.57893966 |
| COL4A6 | -0.931586217 | 0.684628999 | -12.60714541 | 2.57E-34 | 1.95E-33 | 66.56006593 |
| MEX3D | 0.72243505 | 3.258057436 | 12.60557648 | 2.61E-34 | 1.98E-33 | 66.54260003 |
| RNF38 | -0.659113655 | 3.271749235 | -12.60465527 | 2.64E-34 | 2.00E-33 | 66.53234549 |
| ADCY5 | -1.297868207 | 1.343624811 | -12.60371019 | 2.67E-34 | 2.02E-33 | 66.52182598 |
| ENOPH1 | 0.628965519 | 4.611956236 | 12.59590164 | 2.91E-34 | 2.20E-33 | 66.43493295 |
| SORD | 1.253925534 | 3.020936047 | 12.59512064 | 2.94E-34 | 2.22E-33 | 66.42624427 |
| STS | -0.956852223 | 2.289160246 | -12.59478097 | 2.95E-34 | 2.23E-33 | 66.4224656 |
| SVIL | -0.891373472 | 3.330975813 | -12.58967567 | 3.12E-34 | 2.36E-33 | 66.3656804 |
| SHTN1 | 0.783404323 | 3.420198431 | 12.57278245 | 3.77E-34 | 2.85E-33 | 66.17790639 |
| C11orf98 | 0.761058217 | 2.140205095 | 12.56249684 | 4.23E-34 | 3.19E-33 | 66.06367302 |
| CFAP45 | 1.027968501 | 1.395991526 | 12.56141195 | 4.28E-34 | 3.23E-33 | 66.05162829 |
| ZNF213 | 0.610222105 | 2.679809881 | 12.55967516 | 4.36E-34 | 3.29E-33 | 66.03234764 |
| SH3GL1 | 0.609836187 | 4.634553811 | 12.55795219 | 4.45E-34 | 3.35E-33 | 66.01322241 |
| BRIP1 | 0.787254778 | 1.150094784 | 12.55684725 | 4.50E-34 | 3.39E-33 | 66.0009585 |
| SMARCA2 | -0.768889051 | 4.481567052 | -12.5554342 | 4.58E-34 | 3.44E-33 | 65.98527597 |
| APBB1IP | -0.964466175 | 2.296135511 | -12.54885408 | 4.93E-34 | 3.71E-33 | 65.9122655 |
| VANGL1 | 0.707235585 | 2.669947083 | 12.54504526 | 5.14E-34 | 3.86E-33 | 65.87001772 |
| TSPYL2 | -0.746481413 | 2.70722944 | -12.54280268 | 5.27E-34 | 3.96E-33 | 65.84514745 |
| CHEK1 | 0.799826046 | 1.713529645 | 12.54213816 | 5.31E-34 | 3.99E-33 | 65.83777859 |
| PIP4K2C | 0.720370857 | 4.587644579 | 12.54138094 | 5.35E-34 | 4.02E-33 | 65.82938204 |
| GNB2 | 0.712009199 | 6.283509539 | 12.54061727 | 5.40E-34 | 4.05E-33 | 65.82091453 |
| SDSL | 0.928897283 | 2.871027393 | 12.53959407 | 5.46E-34 | 4.10E-33 | 65.8095698 |
| RPA3 | 0.597177234 | 2.973283851 | 12.53250569 | 5.91E-34 | 4.43E-33 | 65.73099735 |
| KLK5 | -2.467805065 | 2.078413737 | -12.53204478 | 5.94E-34 | 4.45E-33 | 65.72588951 |
| ERBB3 | 1.234423775 | 5.275104928 | 12.52915671 | 6.14E-34 | 4.59E-33 | 65.69388688 |
| EPHA4 | -0.855663938 | 1.266451849 | -12.52256564 | 6.60E-34 | 4.94E-33 | 65.62087266 |
| SCO2 | 0.792142389 | 3.670169756 | 12.51359239 | 7.30E-34 | 5.45E-33 | 65.52151668 |
| CENPH | 0.711490734 | 2.374929374 | 12.50908134 | 7.68E-34 | 5.73E-33 | 65.47158892 |
| SNCAIP | -0.595145319 | 0.621103904 | -12.50786281 | 7.78E-34 | 5.81E-33 | 65.45810481 |
| TRIM28 | 0.686120107 | 6.255918322 | 12.50275086 | 8.24E-34 | 6.15E-33 | 65.40154735 |
| AIFM1 | 0.589150842 | 4.18400046 | 12.5027255 | 8.24E-34 | 6.15E-33 | 65.40126683 |
| EMC10 | 0.713399905 | 3.742364371 | 12.50024568 | 8.47E-34 | 6.32E-33 | 65.37383714 |
| CFAP298 | 0.599423432 | 2.95516781 | 12.49040454 | 9.45E-34 | 7.04E-33 | 65.26502395 |
| CDC25A | 0.894714536 | 1.377991234 | 12.48946239 | 9.55E-34 | 7.11E-33 | 65.25461011 |
| AHCY | 0.760811094 | 5.642686281 | 12.48728859 | 9.78E-34 | 7.28E-33 | 65.23058473 |
| DAB2 | -0.93638428 | 3.410714553 | -12.4833947 | 1.02E-33 | 7.60E-33 | 65.18755659 |
| PYCR2 | 0.694689446 | 4.294568413 | 12.48022545 | 1.06E-33 | 7.87E-33 | 65.15254358 |
| TBL3 | 0.650959733 | 3.095289688 | 12.46813403 | 1.21E-33 | 9.00E-33 | 65.01902343 |
| HIPK3 | -0.768003012 | 3.955465968 | -12.46777684 | 1.22E-33 | 9.03E-33 | 65.01508066 |
| CETP | -0.661108862 | 0.803204228 | -12.46532996 | 1.25E-33 | 9.28E-33 | 64.98807358 |
| HIST1H2BH | 1.445247435 | 1.83984896 | 12.45962589 | 1.33E-33 | 9.87E-33 | 64.92513162 |
| LGR6 | -1.437838848 | 1.226269017 | -12.45872833 | 1.34E-33 | 9.96E-33 | 64.91522951 |
| SAYSD1 | 0.625292229 | 3.589043488 | 12.45795046 | 1.36E-33 | 1.00E-32 | 64.90664827 |
| TDRKH | 0.775041513 | 2.551203832 | 12.45461119 | 1.41E-33 | 1.04E-32 | 64.86981499 |
| MYRIP | -0.722631111 | 0.865331406 | -12.4537335 | 1.42E-33 | 1.05E-32 | 64.86013505 |
| KLF16 | 0.756684763 | 2.869144646 | 12.44912765 | 1.49E-33 | 1.11E-32 | 64.80934618 |
| IRAK3 | -0.925213108 | 1.131258767 | -12.44870664 | 1.50E-33 | 1.11E-32 | 64.80470446 |
| SNRPF | 0.720334444 | 3.880017331 | 12.44538388 | 1.56E-33 | 1.15E-32 | 64.76807423 |
| AK4 | -1.22915268 | 2.609297126 | -12.43798568 | 1.69E-33 | 1.25E-32 | 64.68654341 |
| MUC15 | -1.501283588 | 1.127849574 | -12.42943107 | 1.86E-33 | 1.37E-32 | 64.59231517 |
| MEA1 | 0.64932004 | 5.858122297 | 12.42349715 | 1.99E-33 | 1.46E-32 | 64.52698302 |
| SHARPIN | 0.823232256 | 4.587977163 | 12.42153742 | 2.03E-33 | 1.50E-32 | 64.50541181 |
| NR1D2 | -0.818233739 | 3.369413144 | -12.41904152 | 2.09E-33 | 1.54E-32 | 64.47794256 |
| ELOVL3 | -0.74083993 | 0.608273486 | -12.4165436 | 2.15E-33 | 1.58E-32 | 64.4504554 |
| ADRM1 | 0.862233517 | 5.295507432 | 12.41564114 | 2.17E-33 | 1.60E-32 | 64.44052578 |
| SMC4 | 1.034927666 | 2.8565998 | 12.41135598 | 2.27E-33 | 1.67E-32 | 64.39338439 |
| BTBD11 | -0.618511983 | 0.705002777 | -12.40696564 | 2.39E-33 | 1.76E-32 | 64.34509906 |
| CHCHD2 | 0.674332262 | 7.398141407 | 12.39676861 | 2.67E-33 | 1.96E-32 | 64.23300195 |
| SEPT9 | 0.690034165 | 4.749760751 | 12.3963084 | 2.68E-33 | 1.97E-32 | 64.22794443 |
| PLCL2 | -0.74599322 | 1.421782219 | -12.39470071 | 2.73E-33 | 2.01E-32 | 64.21027803 |
| CLIP3 | -0.836040136 | 2.542916634 | -12.38692309 | 2.98E-33 | 2.19E-32 | 64.12483665 |
| GTPBP3 | 0.644006649 | 2.431732575 | 12.38481201 | 3.05E-33 | 2.24E-32 | 64.10165254 |
| CPS1 | -0.603382863 | 0.532522056 | -12.38291307 | 3.11E-33 | 2.28E-32 | 64.0808007 |
| SIRT7 | 0.626021253 | 2.151791108 | 12.37658036 | 3.34E-33 | 2.45E-32 | 64.01128057 |
| LPIN1 | -0.89692133 | 1.948716371 | -12.37517623 | 3.39E-33 | 2.48E-32 | 63.99586979 |
| ARRB1 | -0.958502597 | 2.977160778 | -12.37263543 | 3.49E-33 | 2.55E-32 | 63.96798725 |
| EHMT2 | 0.598664862 | 3.716558056 | 12.37074544 | 3.56E-33 | 2.61E-32 | 63.94724949 |
| BYSL | 0.74918433 | 3.611666166 | 12.36710263 | 3.71E-33 | 2.71E-32 | 63.90728603 |
| FAAP100 | 0.679938351 | 3.282727855 | 12.36623663 | 3.74E-33 | 2.74E-32 | 63.89778687 |
| SRSF5 | -0.744318409 | 4.997616361 | -12.36017476 | 4.00E-33 | 2.93E-32 | 63.8313089 |
| TUBB | 0.716793614 | 7.881960075 | 12.34081947 | 4.95E-33 | 3.62E-32 | 63.61921644 |
| CAPG | 1.054857909 | 5.955220546 | 12.33605027 | 5.22E-33 | 3.81E-32 | 63.56699574 |
| PIGX | 0.628858683 | 3.370339148 | 12.33530476 | 5.26E-33 | 3.84E-32 | 63.55883412 |
| ASPSCR1 | 0.656286446 | 1.804653689 | 12.33511808 | 5.27E-33 | 3.85E-32 | 63.55679049 |
| PRXL2B | 0.834174482 | 3.875117065 | 12.33384112 | 5.35E-33 | 3.90E-32 | 63.54281188 |
| SLC25A30 | -0.593783014 | 1.919347274 | -12.3327943 | 5.41E-33 | 3.94E-32 | 63.53135329 |
| ZNF768 | 0.742389652 | 4.735515452 | 12.33014539 | 5.57E-33 | 4.06E-32 | 63.5023616 |
| COL5A2 | 1.570563212 | 5.663126804 | 12.32675143 | 5.78E-33 | 4.21E-32 | 63.46522257 |
| SPAG1 | 0.811999744 | 2.085100793 | 12.32430812 | 5.94E-33 | 4.32E-32 | 63.43849106 |
| PTPRG | -0.793325209 | 2.076592286 | -12.32392605 | 5.97E-33 | 4.34E-32 | 63.43431134 |
| DDR1 | 0.896107366 | 5.306288568 | 12.32092471 | 6.17E-33 | 4.48E-32 | 63.401481 |
| PNKD | 0.796261393 | 3.59497527 | 12.31566309 | 6.54E-33 | 4.75E-32 | 63.34394156 |
| PCDHGC3 | -0.897527173 | 1.949971206 | -12.31538335 | 6.56E-33 | 4.76E-32 | 63.34088287 |
| FRAT2 | 0.679796675 | 3.218582556 | 12.31101491 | 6.88E-33 | 4.99E-32 | 63.29312626 |
| MRPL51 | 0.678688737 | 5.767498367 | 12.31059294 | 6.91E-33 | 5.01E-32 | 63.28851397 |
| MYO19 | 0.638046773 | 1.964207076 | 12.30962122 | 6.98E-33 | 5.07E-32 | 63.27789295 |
| KLK7 | -1.841909782 | 1.308577447 | -12.30870904 | 7.06E-33 | 5.12E-32 | 63.26792335 |
| PRRT3 | 1.103265468 | 2.140805085 | 12.30839865 | 7.08E-33 | 5.13E-32 | 63.26453116 |
| MRPL9 | 0.628748871 | 4.883318384 | 12.30452874 | 7.39E-33 | 5.35E-32 | 63.2222425 |
| PDE9A | -0.940919358 | 1.290288461 | -12.30345059 | 7.48E-33 | 5.41E-32 | 63.21046278 |
| SOS1 | -0.650023773 | 3.013889712 | -12.29485389 | 8.22E-33 | 5.95E-32 | 63.11656526 |
| MAPK8IP2 | 1.408291455 | 2.169012037 | 12.28574564 | 9.08E-33 | 6.56E-32 | 63.01713597 |
| KMT2A | -0.676812852 | 2.345621657 | -12.28347942 | 9.31E-33 | 6.73E-32 | 62.99240584 |
| ARF3 | 0.591687211 | 5.721943707 | 12.28103825 | 9.56E-33 | 6.91E-32 | 62.96577065 |
| TCF3 | 0.74388009 | 3.828169382 | 12.27866176 | 9.82E-33 | 7.08E-32 | 62.93984504 |
| LPAR1 | -0.917460714 | 2.617461096 | -12.27411533 | 1.03E-32 | 7.44E-32 | 62.89025798 |
| ZNF707 | 0.662203429 | 1.799159706 | 12.26869335 | 1.10E-32 | 7.89E-32 | 62.83114018 |
| MAP3K8 | -0.944224416 | 2.110760124 | -12.26577727 | 1.13E-32 | 8.15E-32 | 62.7993535 |
| WDYHV1 | 0.66431785 | 2.996291236 | 12.25765206 | 1.24E-32 | 8.91E-32 | 62.71081579 |
| KLF7 | -0.784048305 | 2.145142481 | -12.24598285 | 1.40E-32 | 1.01E-31 | 62.58374003 |
| ISOC2 | 0.777121384 | 3.376825028 | 12.23702855 | 1.55E-32 | 1.12E-31 | 62.48629312 |
| ALS2CL | -0.666409399 | 1.142173616 | -12.23699563 | 1.55E-32 | 1.12E-31 | 62.48593494 |
| BAIAP2L1 | 0.840493196 | 3.13219643 | 12.2368793 | 1.55E-32 | 1.12E-31 | 62.48466933 |
| NSD2 | 0.636523542 | 2.407578761 | 12.23171861 | 1.64E-32 | 1.18E-31 | 62.42853291 |
| PRXL2A | -0.881929079 | 4.17524006 | -12.22955067 | 1.68E-32 | 1.21E-31 | 62.40495616 |
| PCGF5 | -0.656902134 | 2.822515525 | -12.22741837 | 1.72E-32 | 1.24E-31 | 62.38177022 |
| SLC35F3 | -0.936528703 | 0.759009394 | -12.22592783 | 1.75E-32 | 1.26E-31 | 62.36556441 |
| OAF | -0.833651953 | 3.382726853 | -12.22385005 | 1.79E-32 | 1.28E-31 | 62.34297642 |
| CCL13 | -1.275503619 | 1.141837662 | -12.22139132 | 1.84E-32 | 1.32E-31 | 62.31625098 |
| ZNF239 | 0.83348521 | 2.489635617 | 12.22051701 | 1.86E-32 | 1.33E-31 | 62.30674853 |
| TBRG4 | 0.59267441 | 3.452719313 | 12.21375391 | 2.00E-32 | 1.43E-31 | 62.23326195 |
| BEND5 | -0.721170269 | 1.250038026 | -12.19551735 | 2.44E-32 | 1.74E-31 | 62.03526467 |
| CIAO3 | 0.665918685 | 2.302782677 | 12.19305977 | 2.51E-32 | 1.79E-31 | 62.0086 |
| PSMB3 | 0.902257537 | 6.411692679 | 12.19212977 | 2.53E-32 | 1.81E-31 | 61.99851056 |
| NEK7 | -0.73104789 | 3.698174152 | -12.18883413 | 2.63E-32 | 1.87E-31 | 61.96276162 |
| PPDPF | 1.051027452 | 7.798465031 | 12.18710666 | 2.68E-32 | 1.91E-31 | 61.94402623 |
| TTLL12 | 1.040091151 | 4.28458317 | 12.1856068 | 2.72E-32 | 1.94E-31 | 61.92776099 |
| NDUFB11 | 0.705914617 | 5.773089064 | 12.18546349 | 2.72E-32 | 1.94E-31 | 61.92620699 |
| EFNB3 | -1.01989888 | 1.416796285 | -12.16748644 | 3.31E-32 | 2.36E-31 | 61.73137849 |
| ACCS | -0.684260411 | 1.556732444 | -12.1659322 | 3.37E-32 | 2.40E-31 | 61.71454481 |
| MPP6 | -0.63163036 | 0.794207436 | -12.16582447 | 3.37E-32 | 2.40E-31 | 61.71337811 |
| E2F7 | 0.665714457 | 0.779111597 | 12.16199831 | 3.52E-32 | 2.50E-31 | 61.67194497 |
| ABCC6 | -0.67062905 | 0.708642105 | -12.16082121 | 3.56E-32 | 2.53E-31 | 61.65920039 |
| WNT11 | -1.330713021 | 1.305729288 | -12.16032213 | 3.58E-32 | 2.55E-31 | 61.65379704 |
| ARMC6 | 0.609640314 | 3.192423839 | 12.15970837 | 3.61E-32 | 2.56E-31 | 61.64715238 |
| ZNF391 | -0.625981014 | 1.014619258 | -12.15769525 | 3.69E-32 | 2.62E-31 | 61.62535978 |
| ZNF354C | -0.631269861 | 1.229725268 | -12.15705489 | 3.71E-32 | 2.64E-31 | 61.61842829 |
| ST14 | 1.098253202 | 5.046604428 | 12.15187991 | 3.93E-32 | 2.79E-31 | 61.56242305 |
| SNX1 | -0.641235063 | 3.874532236 | -12.14863846 | 4.07E-32 | 2.89E-31 | 61.52735251 |
| THBD | -1.040360887 | 2.912296092 | -12.14602201 | 4.19E-32 | 2.97E-31 | 61.49904942 |
| MFNG | -0.854517401 | 2.078520697 | -12.14538506 | 4.22E-32 | 2.99E-31 | 61.49215995 |
| INTS8 | 0.651775834 | 2.961937026 | 12.13284015 | 4.83E-32 | 3.42E-31 | 61.35652888 |
| AKT3 | -1.03598503 | 2.179773964 | -12.12807462 | 5.09E-32 | 3.60E-31 | 61.30503442 |
| ZBTB12 | 0.785620057 | 2.437339499 | 12.12696636 | 5.15E-32 | 3.64E-31 | 61.2930613 |
| HSPBP1 | 0.827822493 | 4.11671551 | 12.12609799 | 5.20E-32 | 3.68E-31 | 61.28368038 |
| GPR137 | 0.674855144 | 3.052555392 | 12.12419398 | 5.31E-32 | 3.75E-31 | 61.26311342 |
| RNF39 | -1.112925901 | 1.795373573 | -12.1228892 | 5.39E-32 | 3.80E-31 | 61.24902074 |
| ITGA6 | -1.257937182 | 4.14873892 | -12.12145547 | 5.47E-32 | 3.86E-31 | 61.23353669 |
| PPIL1 | 0.655262691 | 4.218196926 | 12.1195135 | 5.59E-32 | 3.94E-31 | 61.21256596 |
| CEP85 | 0.688581037 | 2.354751959 | 12.11773522 | 5.70E-32 | 4.02E-31 | 61.19336529 |
| TUBB2B | -1.340377856 | 1.326492773 | -12.1096074 | 6.22E-32 | 4.39E-31 | 61.10563423 |
| CCT5 | 0.736140844 | 5.205930609 | 12.10830654 | 6.31E-32 | 4.45E-31 | 61.09159706 |
| HIST1H2AD | 1.41996926 | 1.638768596 | 12.1069331 | 6.41E-32 | 4.51E-31 | 61.07677815 |
| HASPIN | 0.625098683 | 0.915314146 | 12.10060105 | 6.86E-32 | 4.83E-31 | 61.00847419 |
| ALB | -1.860677443 | 0.944080539 | -12.09867662 | 7.01E-32 | 4.93E-31 | 60.98772084 |
| CORO2A | 1.05196882 | 3.152323113 | 12.09569053 | 7.24E-32 | 5.09E-31 | 60.95552365 |
| RASL11A | -1.03637181 | 2.100736897 | -12.09309074 | 7.45E-32 | 5.24E-31 | 60.92749659 |
| EIF4B | -0.639512104 | 6.00858154 | -12.09144619 | 7.58E-32 | 5.33E-31 | 60.90977004 |
| TAT | -2.418654426 | 1.412328423 | -12.09036465 | 7.67E-32 | 5.39E-31 | 60.89811314 |
| CCDC78 | 1.012488083 | 1.154018197 | 12.08750767 | 7.91E-32 | 5.56E-31 | 60.86732443 |
| MRPL58 | 0.715581594 | 4.485776707 | 12.08584287 | 8.06E-32 | 5.66E-31 | 60.84938611 |
| MARK1 | -0.836383902 | 1.20876564 | -12.07974703 | 8.61E-32 | 6.04E-31 | 60.78371943 |
| COA4 | 0.67502082 | 4.256181588 | 12.0786395 | 8.71E-32 | 6.11E-31 | 60.77179144 |
| NCDN | 0.776964039 | 3.416729917 | 12.07760333 | 8.81E-32 | 6.18E-31 | 60.76063287 |
| SH3BP5 | -0.784718293 | 1.976145734 | -12.07514942 | 9.05E-32 | 6.34E-31 | 60.73420934 |
| FKBP10 | 1.045968482 | 5.039187089 | 12.07248846 | 9.31E-32 | 6.52E-31 | 60.70556123 |
| MERTK | -0.704525908 | 1.729827178 | -12.07213235 | 9.35E-32 | 6.54E-31 | 60.70172766 |
| STX1A | 0.712883888 | 1.512603598 | 12.07015371 | 9.55E-32 | 6.68E-31 | 60.68042912 |
| FYN | -0.800446408 | 2.363134076 | -12.06979077 | 9.59E-32 | 6.71E-31 | 60.67652259 |
| PRR13 | 0.593971711 | 4.820012775 | 12.06902193 | 9.67E-32 | 6.76E-31 | 60.66824756 |
| B3GAT3 | 0.813408771 | 4.049795396 | 12.06883709 | 9.69E-32 | 6.77E-31 | 60.66625818 |
| BIK | 1.320721823 | 3.481580924 | 12.06655628 | 9.93E-32 | 6.94E-31 | 60.64171256 |
| HBEGF | -0.880025848 | 2.084475979 | -12.06584649 | 1.00E-31 | 6.99E-31 | 60.63407461 |
| CDKN2C | -0.900464394 | 2.659383937 | -12.06391196 | 1.02E-31 | 7.14E-31 | 60.61325933 |
| PHACTR2 | -0.681487584 | 1.895014005 | -12.05791275 | 1.09E-31 | 7.61E-31 | 60.54872532 |
| IGF2 | -1.701320047 | 3.583251605 | -12.05475884 | 1.13E-31 | 7.87E-31 | 60.51480858 |
| PSEN2 | 0.642471169 | 3.259458226 | 12.05179301 | 1.17E-31 | 8.12E-31 | 60.48292075 |
| EMILIN3 | -0.746001886 | 0.365404951 | -12.05033129 | 1.18E-31 | 8.25E-31 | 60.46720691 |
| DARS2 | 0.772727511 | 3.577602108 | 12.05021533 | 1.19E-31 | 8.25E-31 | 60.4659604 |
| KLHL2 | -0.752043686 | 2.491455065 | -12.0450543 | 1.25E-31 | 8.72E-31 | 60.41049069 |
| CCT6A | 0.641977274 | 5.826223393 | 12.04259483 | 1.29E-31 | 8.95E-31 | 60.38406342 |
| PINK1 | -0.614168801 | 2.525667084 | -12.04031149 | 1.32E-31 | 9.17E-31 | 60.3595324 |
| PURA | -0.586320687 | 2.153314132 | -12.03685536 | 1.37E-31 | 9.51E-31 | 60.32240853 |
| RARB | -0.864234366 | 1.560884374 | -12.02529611 | 1.55E-31 | 1.08E-30 | 60.19830604 |
| DDX49 | 0.67079201 | 4.146153198 | 12.01947056 | 1.65E-31 | 1.14E-30 | 60.13579723 |
| CLPP | 0.681310995 | 4.173855124 | 12.01543441 | 1.73E-31 | 1.19E-30 | 60.09250283 |
| FBXO45 | 0.612028468 | 2.67975309 | 12.01374166 | 1.76E-31 | 1.22E-30 | 60.07434865 |
| EEF1A1 | -0.672081551 | 9.62557458 | -12.01137395 | 1.80E-31 | 1.25E-30 | 60.04895914 |
| PMF1 | 0.702719059 | 3.494211224 | 12.01087523 | 1.81E-31 | 1.25E-30 | 60.04361183 |
| SLC23A2 | -0.667382526 | 2.456372984 | -12.00874565 | 1.86E-31 | 1.28E-30 | 60.02077996 |
| ARHGAP23 | -0.749049694 | 2.04580406 | -12.00822605 | 1.87E-31 | 1.29E-30 | 60.01520966 |
| CXCL11 | 1.700723704 | 2.155098248 | 12.00654643 | 1.90E-31 | 1.31E-30 | 59.99720483 |
| SLC35B1 | 0.709061849 | 3.342310174 | 12.00391161 | 1.96E-31 | 1.35E-30 | 59.9689645 |
| SLC25A19 | 0.599158079 | 1.779886754 | 12.00299998 | 1.97E-31 | 1.36E-30 | 59.95919479 |
| RTL5 | -0.825650885 | 1.570638561 | -12.00158852 | 2.00E-31 | 1.38E-30 | 59.94406948 |
| SERINC1 | -0.796700917 | 6.449638488 | -12.00077497 | 2.02E-31 | 1.39E-30 | 59.93535212 |
| MTFP1 | 0.643862668 | 1.445712739 | 11.99948253 | 2.05E-31 | 1.41E-30 | 59.92150423 |
| SULF1 | 1.398910509 | 4.47517852 | 11.99907478 | 2.06E-31 | 1.42E-30 | 59.91713565 |
| GNE | -0.791021138 | 2.72135647 | -11.99638834 | 2.12E-31 | 1.46E-30 | 59.88835637 |
| RAB11FIP4 | 0.770102516 | 1.998668592 | 11.99023662 | 2.27E-31 | 1.56E-30 | 59.82247313 |
| HYAL3 | 0.681212832 | 1.678543817 | 11.98999489 | 2.27E-31 | 1.56E-30 | 59.81988475 |
| RAB3A | 0.824676598 | 2.043464093 | 11.9858036 | 2.38E-31 | 1.63E-30 | 59.77501313 |
| NCAPG2 | 0.773076399 | 2.454460889 | 11.97467943 | 2.68E-31 | 1.84E-30 | 59.65597854 |
| FZD2 | 0.834421691 | 2.024690772 | 11.9713868 | 2.78E-31 | 1.91E-30 | 59.62076225 |
| RPS6KB2 | 0.704962984 | 2.912923217 | 11.96995936 | 2.82E-31 | 1.94E-30 | 59.60549752 |
| ZNF467 | 1.060761211 | 2.936667206 | 11.96945777 | 2.83E-31 | 1.95E-30 | 59.60013391 |
| YBX3 | -1.14991487 | 3.71215236 | -11.96920651 | 2.84E-31 | 1.95E-30 | 59.59744728 |
| KLF13 | -0.786551839 | 3.17282911 | -11.95391051 | 3.35E-31 | 2.30E-30 | 59.43397258 |
| MCM3 | 0.736555903 | 5.122495342 | 11.9531017 | 3.38E-31 | 2.32E-30 | 59.42533307 |
| RSPO1 | -0.89803378 | 0.644494401 | -11.95132229 | 3.45E-31 | 2.36E-30 | 59.40632749 |
| PDGFRL | -1.198199408 | 3.31322555 | -11.9443945 | 3.71E-31 | 2.54E-30 | 59.33235424 |
| CDS1 | 0.852527158 | 3.613143068 | 11.94199701 | 3.81E-31 | 2.61E-30 | 59.30676225 |
| BBC3 | 0.884640197 | 2.109274155 | 11.93940594 | 3.92E-31 | 2.68E-30 | 59.27910845 |
| TBC1D2B | -0.645186046 | 2.691369528 | -11.9390552 | 3.93E-31 | 2.69E-30 | 59.27536543 |
| SLC27A1 | -0.678822095 | 2.465171534 | -11.93576095 | 4.07E-31 | 2.79E-30 | 59.24021447 |
| FAM166B | -0.722135443 | 0.652658451 | -11.93536241 | 4.09E-31 | 2.80E-30 | 59.23596231 |
| PIGM | 0.614387387 | 3.167038031 | 11.92843106 | 4.41E-31 | 3.01E-30 | 59.16202887 |
| ARF5 | 0.683303426 | 6.15532725 | 11.92783331 | 4.43E-31 | 3.03E-30 | 59.15565452 |
| SLC7A5 | 1.697642638 | 3.903286747 | 11.92690026 | 4.48E-31 | 3.06E-30 | 59.14570517 |
| TSEN15 | 0.613153609 | 4.030726582 | 11.92647691 | 4.50E-31 | 3.07E-30 | 59.14119107 |
| ARHGEF16 | 0.808451593 | 2.309454958 | 11.92587863 | 4.53E-31 | 3.09E-30 | 59.13481189 |
| CASP6 | 0.652685447 | 3.853710538 | 11.92285322 | 4.68E-31 | 3.19E-30 | 59.10255728 |
| TUB | -0.839019332 | 1.451622832 | -11.91780869 | 4.94E-31 | 3.37E-30 | 59.04879077 |
| SYAP1 | 0.71508637 | 4.449126884 | 11.91288582 | 5.21E-31 | 3.55E-30 | 58.99633832 |
| PIK3IP1 | -0.78523718 | 3.614315721 | -11.90654876 | 5.57E-31 | 3.79E-30 | 58.9288429 |
| SNN | -0.699886265 | 3.83335936 | -11.90625219 | 5.59E-31 | 3.80E-30 | 58.92568487 |
| DEFB1 | -1.93813462 | 2.00549764 | -11.88855637 | 6.76E-31 | 4.59E-30 | 58.73736238 |
| CD93 | -0.956824337 | 3.847105132 | -11.8873395 | 6.85E-31 | 4.64E-30 | 58.72442031 |
| FAM50A | 0.79840394 | 4.641740026 | 11.88721545 | 6.86E-31 | 4.65E-30 | 58.72310101 |
| MBOAT2 | 1.037628899 | 3.168015724 | 11.88022853 | 7.39E-31 | 5.01E-30 | 58.64881234 |
| DNAJC1 | 1.102635254 | 4.829406526 | 11.87725075 | 7.63E-31 | 5.17E-30 | 58.6171614 |
| IL34 | -1.353701507 | 1.960431128 | -11.87515289 | 7.80E-31 | 5.28E-30 | 58.594867 |
| CYBRD1 | -1.563203479 | 5.45513075 | -11.87123904 | 8.14E-31 | 5.51E-30 | 58.55328197 |
| RAMP1 | 1.791621325 | 2.940333212 | 11.8653643 | 8.66E-31 | 5.85E-30 | 58.49088259 |
| MUC1 | 2.149960154 | 5.972051227 | 11.86327185 | 8.86E-31 | 5.98E-30 | 58.46866325 |
| FAP | 1.131723775 | 2.542893688 | 11.85897838 | 9.28E-31 | 6.26E-30 | 58.42308132 |
| CEMIP | 1.161716103 | 1.476932706 | 11.85865222 | 9.31E-31 | 6.28E-30 | 58.41961914 |
| C16orf89 | -1.842216385 | 1.98041516 | -11.85816665 | 9.36E-31 | 6.31E-30 | 58.41446495 |
| HIST1H2BC | 1.569217758 | 2.60966324 | 11.8573291 | 9.44E-31 | 6.37E-30 | 58.40557508 |
| COMTD1 | 1.067343797 | 2.523841905 | 11.85642412 | 9.53E-31 | 6.43E-30 | 58.39597005 |
| BICC1 | -0.896870844 | 1.935856668 | -11.84976462 | 1.02E-30 | 6.90E-30 | 58.32530685 |
| SORBS2 | -1.100378194 | 1.908097869 | -11.8497542 | 1.02E-30 | 6.90E-30 | 58.32519636 |
| COX8A | 0.749934567 | 7.612148609 | 11.84319999 | 1.10E-30 | 7.39E-30 | 58.25568102 |
| HSH2D | 1.39272745 | 2.235980281 | 11.84264309 | 1.10E-30 | 7.44E-30 | 58.24977584 |
| ATOX1 | 0.665216526 | 3.447204924 | 11.84150933 | 1.12E-30 | 7.52E-30 | 58.2377545 |
| MB | 1.343879446 | 3.312303644 | 11.83960061 | 1.14E-30 | 7.68E-30 | 58.21751822 |
| HIBADH | -0.621938916 | 4.665635073 | -11.83456788 | 1.20E-30 | 8.09E-30 | 58.16417363 |
| SLC1A3 | -0.891815246 | 1.829069056 | -11.83349008 | 1.22E-30 | 8.18E-30 | 58.1527517 |
| MCM6 | 0.794415131 | 3.956399435 | 11.83211208 | 1.24E-30 | 8.30E-30 | 58.13814976 |
| VIPR1 | -0.80253088 | 1.65386632 | -11.82808836 | 1.29E-30 | 8.66E-30 | 58.09552002 |
| KIAA1671 | -0.739110398 | 2.801381906 | -11.82581618 | 1.32E-30 | 8.87E-30 | 58.07145235 |
| LDHB | -1.553896312 | 5.478971016 | -11.81969622 | 1.41E-30 | 9.47E-30 | 58.00664563 |
| HOXC11 | 1.266778128 | 1.44722502 | 11.81929397 | 1.42E-30 | 9.50E-30 | 58.00238694 |
| RAB6B | -0.785204576 | 1.234089118 | -11.81777116 | 1.44E-30 | 9.66E-30 | 57.98626596 |
| OXLD1 | 0.877553355 | 3.619171857 | 11.81710501 | 1.45E-30 | 9.72E-30 | 57.97921435 |
| DENND1C | 0.67781166 | 2.225903975 | 11.81325175 | 1.51E-30 | 1.01E-29 | 57.93843136 |
| EVA1C | -0.916128575 | 2.118980014 | -11.81277274 | 1.52E-30 | 1.02E-29 | 57.93336217 |
| JMJD4 | 0.66269114 | 2.647363641 | 11.81244207 | 1.52E-30 | 1.02E-29 | 57.92986304 |
| MRPS26 | 0.809287649 | 5.207619163 | 11.80956704 | 1.57E-30 | 1.05E-29 | 57.89944212 |
| ABHD14B | -0.736418877 | 4.515208088 | -11.80539993 | 1.64E-30 | 1.10E-29 | 57.85536009 |
| PTMA | 0.647249235 | 7.717632453 | 11.79906087 | 1.76E-30 | 1.17E-29 | 57.78832561 |
| UQCC3 | 0.910038751 | 3.115476921 | 11.79804207 | 1.78E-30 | 1.19E-29 | 57.77755463 |
| IL1R1 | -1.15258107 | 3.386166346 | -11.79785593 | 1.78E-30 | 1.19E-29 | 57.77558682 |
| SLC39A4 | 1.060586187 | 2.164017642 | 11.79547547 | 1.83E-30 | 1.22E-29 | 57.7504233 |
| FXYD3 | 1.418927306 | 5.586790152 | 11.7892003 | 1.95E-30 | 1.30E-29 | 57.68410848 |
| CST2 | 1.456600692 | 1.487396072 | 11.78233149 | 2.10E-30 | 1.40E-29 | 57.61155226 |
| RASL11B | 0.937707887 | 1.796536634 | 11.78192354 | 2.11E-30 | 1.40E-29 | 57.60724417 |
| TFPT | 0.974227022 | 3.785565573 | 11.77522359 | 2.27E-30 | 1.51E-29 | 57.53650583 |
| NECAB3 | 1.005252688 | 3.223868991 | 11.77378087 | 2.30E-30 | 1.53E-29 | 57.52127773 |
| SSR4 | 0.799228181 | 5.18986097 | 11.77347262 | 2.31E-30 | 1.54E-29 | 57.51802434 |
| TMEM241 | 0.663396248 | 2.859029801 | 11.76981048 | 2.40E-30 | 1.60E-29 | 57.47937741 |
| ATP13A2 | 0.77251787 | 3.860673031 | 11.76695743 | 2.47E-30 | 1.64E-29 | 57.44927555 |
| NAA60 | 0.596101118 | 2.61718912 | 11.76049981 | 2.65E-30 | 1.76E-29 | 57.38116397 |
| TPMT | 0.613185685 | 3.555905249 | 11.75367762 | 2.85E-30 | 1.89E-29 | 57.30923938 |
| JMJD1C | -0.731385485 | 2.907278626 | -11.7530076 | 2.87E-30 | 1.90E-29 | 57.30217726 |
| FAM168B | -0.601161424 | 4.540154622 | -11.75176772 | 2.91E-30 | 1.93E-29 | 57.28910973 |
| PDE5A | -1.032202126 | 1.765385176 | -11.74735225 | 3.05E-30 | 2.02E-29 | 57.24258217 |
| RRS1 | 0.899796537 | 4.081857149 | 11.74543036 | 3.11E-30 | 2.06E-29 | 57.22233483 |
| KIAA1109 | -0.783243473 | 2.465216411 | -11.7429903 | 3.19E-30 | 2.11E-29 | 57.19663225 |
| TSPAN13 | 1.277891989 | 6.712042272 | 11.73975335 | 3.30E-30 | 2.18E-29 | 57.16254216 |
| AMT | -0.713868687 | 1.171379313 | -11.73796981 | 3.37E-30 | 2.23E-29 | 57.14376185 |
| PIP5K1B | -0.82781688 | 0.864971244 | -11.73538817 | 3.46E-30 | 2.29E-29 | 57.11658178 |
| IL6R | -0.811377589 | 1.791856363 | -11.73084617 | 3.63E-30 | 2.40E-29 | 57.06877423 |
| ANKRD22 | 1.114638726 | 1.972245514 | 11.73019167 | 3.65E-30 | 2.41E-29 | 57.06188634 |
| ADCK5 | 0.82230416 | 2.163180296 | 11.72328834 | 3.93E-30 | 2.60E-29 | 56.98925552 |
| PECR | -0.713081745 | 2.055637316 | -11.72103574 | 4.03E-30 | 2.66E-29 | 56.96556298 |
| EFNA1 | 0.928546547 | 5.52840739 | 11.71562775 | 4.26E-30 | 2.81E-29 | 56.90869722 |
| SLC6A14 | -1.471039286 | 1.007873008 | -11.71408535 | 4.33E-30 | 2.85E-29 | 56.89248254 |
| GOLGA8A | -1.012516428 | 1.523762947 | -11.7139078 | 4.34E-30 | 2.86E-29 | 56.8906161 |
| TRIM68 | -0.716534996 | 1.800606978 | -11.70853237 | 4.60E-30 | 3.02E-29 | 56.83412 |
| SLC25A20 | -0.658588471 | 3.343442881 | -11.7065754 | 4.69E-30 | 3.09E-29 | 56.81355726 |
| RAB13 | 0.677902397 | 5.370188811 | 11.7032736 | 4.86E-30 | 3.19E-29 | 56.77887003 |
| C19orf24 | 0.824607689 | 3.729030729 | 11.69873729 | 5.10E-30 | 3.35E-29 | 56.73122619 |
| SEPT4 | -0.658910344 | 1.506359196 | -11.69606585 | 5.25E-30 | 3.44E-29 | 56.70317551 |
| FUNDC1 | 0.613229001 | 3.942196953 | 11.6952563 | 5.29E-30 | 3.47E-29 | 56.69467617 |
| KRT15 | -2.456201167 | 3.364071793 | -11.69468316 | 5.32E-30 | 3.49E-29 | 56.68865901 |
| CETN2 | 0.616894962 | 5.285432917 | 11.69191158 | 5.48E-30 | 3.59E-29 | 56.65956504 |
| TNS4 | -1.655862842 | 2.45762771 | -11.68893239 | 5.66E-30 | 3.71E-29 | 56.62829783 |
| DDIAS | 0.6969416 | 1.237148011 | 11.68181011 | 6.10E-30 | 3.99E-29 | 56.55357372 |
| PRR15L | 1.423725936 | 4.665555733 | 11.66737737 | 7.10E-30 | 4.64E-29 | 56.40226236 |
| ROR1 | -0.651064772 | 0.914229939 | -11.66575205 | 7.23E-30 | 4.72E-29 | 56.38523194 |
| CDC42SE1 | 0.595375664 | 5.313182466 | 11.66533083 | 7.26E-30 | 4.74E-29 | 56.38081862 |
| MZT2A | 0.998327782 | 3.065047992 | 11.66499516 | 7.28E-30 | 4.75E-29 | 56.37730178 |
| FMOD | -1.589990167 | 4.95429411 | -11.66349074 | 7.40E-30 | 4.83E-29 | 56.3615407 |
| NDUFS6 | 0.876723327 | 4.979424842 | 11.66221068 | 7.50E-30 | 4.89E-29 | 56.34813148 |
| ERRFI1 | -1.065226435 | 4.169617521 | -11.65767127 | 7.87E-30 | 5.13E-29 | 56.30058834 |
| ACSL5 | -1.042227248 | 2.589994899 | -11.65388413 | 8.19E-30 | 5.34E-29 | 56.26093529 |
| PER3 | -0.834751971 | 2.231953611 | -11.64680054 | 8.82E-30 | 5.75E-29 | 56.18679467 |
| CDIP1 | -0.688799621 | 2.741281007 | -11.64659193 | 8.84E-30 | 5.76E-29 | 56.18461176 |
| SMKR1 | 1.00802757 | 1.798372358 | 11.64328591 | 9.16E-30 | 5.96E-29 | 56.15002195 |
| LIG3 | 0.594552378 | 2.217443183 | 11.64220464 | 9.26E-30 | 6.03E-29 | 56.13871068 |
| TAGLN2 | 0.816274584 | 8.056297986 | 11.63864063 | 9.62E-30 | 6.26E-29 | 56.10143305 |
| HAAO | -0.854464684 | 1.403584059 | -11.63819723 | 9.66E-30 | 6.28E-29 | 56.09679603 |
| NME4 | 0.839436885 | 4.947589882 | 11.63569935 | 9.92E-30 | 6.45E-29 | 56.07067577 |
| MRPL36 | 0.626680303 | 3.861213905 | 11.61523727 | 1.23E-29 | 7.99E-29 | 55.85687318 |
| GPATCH4 | 0.654467335 | 3.379017537 | 11.60770476 | 1.33E-29 | 8.64E-29 | 55.77824377 |
| 9-Mar | 0.593047628 | 2.46630317 | 11.6065046 | 1.35E-29 | 8.75E-29 | 55.76571939 |
| MANEAL | 1.042678822 | 2.668222821 | 11.60365069 | 1.39E-29 | 9.01E-29 | 55.73594146 |
| TNNT1 | 2.170018106 | 2.599585134 | 11.59910987 | 1.46E-29 | 9.44E-29 | 55.68857417 |
| INHBB | -1.33628407 | 3.370804722 | -11.5958829 | 1.51E-29 | 9.77E-29 | 55.65492125 |
| IBSP | 1.116558534 | 1.035028931 | 11.59580804 | 1.51E-29 | 9.77E-29 | 55.65414068 |
| NLGN4X | -0.77389586 | 0.76539803 | -11.59047275 | 1.60E-29 | 1.03E-28 | 55.59851748 |
| COX6A1 | 0.74315324 | 6.192835979 | 11.58963938 | 1.61E-29 | 1.04E-28 | 55.58983093 |
| ZNF521 | -1.02159658 | 2.218737804 | -11.58880652 | 1.62E-29 | 1.05E-28 | 55.58115037 |
| TRMT112 | 0.661093679 | 6.069597232 | 11.58125308 | 1.76E-29 | 1.14E-28 | 55.50244577 |
| PDAP1 | 0.597262987 | 5.213915208 | 11.57966085 | 1.79E-29 | 1.16E-28 | 55.4858605 |
| SOCS3 | -1.295694734 | 4.399947745 | -11.57963284 | 1.79E-29 | 1.16E-28 | 55.48556876 |
| MMP1 | 2.212275442 | 2.236161436 | 11.57671402 | 1.84E-29 | 1.19E-28 | 55.45516991 |
| TMUB1 | 0.808397007 | 3.897650516 | 11.57251861 | 1.93E-29 | 1.24E-28 | 55.41148638 |
| PRSS22 | 1.010077054 | 2.501316213 | 11.56985082 | 1.98E-29 | 1.28E-28 | 55.38371543 |
| PTEN | -0.635045506 | 3.346880539 | -11.56398895 | 2.11E-29 | 1.36E-28 | 55.32271283 |
| REM1 | -0.619177313 | 0.870744297 | -11.56239751 | 2.14E-29 | 1.38E-28 | 55.30615552 |
| RPL21 | -0.730710181 | 6.474849619 | -11.55915216 | 2.22E-29 | 1.43E-28 | 55.27239655 |
| SNRPA1 | 0.616368055 | 3.15310675 | 11.55828881 | 2.24E-29 | 1.44E-28 | 55.26341712 |
| VCL | -0.715784434 | 4.280195984 | -11.55380303 | 2.35E-29 | 1.51E-28 | 55.21677017 |
| AGPAT2 | -1.093093766 | 4.866845443 | -11.55340092 | 2.36E-29 | 1.51E-28 | 55.21258934 |
| PET100 | 0.751949692 | 3.315571039 | 11.54862366 | 2.48E-29 | 1.59E-28 | 55.16292868 |
| ILDR1 | 0.791732626 | 2.36417253 | 11.54827687 | 2.48E-29 | 1.60E-28 | 55.1593244 |
| ATP13A5 | -0.947315273 | 0.616625659 | -11.54619999 | 2.54E-29 | 1.63E-28 | 55.13774034 |
| HIST2H2BE | 1.462493993 | 4.563749307 | 11.54312009 | 2.62E-29 | 1.68E-28 | 55.10573822 |
| CUTA | 0.669431262 | 6.028875094 | 11.53881968 | 2.74E-29 | 1.76E-28 | 55.0610656 |
| FCER1A | -1.167826069 | 1.534943473 | -11.53740713 | 2.78E-29 | 1.78E-28 | 55.04639495 |
| FANCA | 0.645908901 | 1.018886178 | 11.53684181 | 2.80E-29 | 1.79E-28 | 55.04052399 |
| STAP2 | 0.917394785 | 3.691211121 | 11.53679202 | 2.80E-29 | 1.79E-28 | 55.04000687 |
| MXD3 | 0.690168398 | 1.280618584 | 11.53312985 | 2.91E-29 | 1.86E-28 | 55.00198014 |
| SNAPC2 | 0.775521308 | 3.767339385 | 11.53123801 | 2.97E-29 | 1.90E-28 | 54.98233973 |
| PNPLA7 | -0.719235098 | 1.244292061 | -11.52909893 | 3.04E-29 | 1.94E-28 | 54.9601357 |
| CARD19 | 0.791999161 | 3.216827879 | 11.52582239 | 3.14E-29 | 2.01E-28 | 54.92613093 |
| SMG5 | 0.631989276 | 4.69430056 | 11.52526812 | 3.16E-29 | 2.02E-28 | 54.92037935 |
| TIMP1 | 1.012714316 | 7.386253394 | 11.52484046 | 3.18E-29 | 2.03E-28 | 54.91594165 |
| EPHB6 | -0.950136658 | 1.125547736 | -11.52300484 | 3.24E-29 | 2.07E-28 | 54.89689585 |
| TGFB1I1 | -0.827491889 | 2.603582717 | -11.52088275 | 3.31E-29 | 2.11E-28 | 54.87488069 |
| KNTC1 | 0.630742646 | 1.844605425 | 11.51761041 | 3.42E-29 | 2.18E-28 | 54.84093893 |
| PEMT | -0.79215946 | 3.540289522 | -11.51654362 | 3.46E-29 | 2.21E-28 | 54.82987545 |
| ATL1 | -0.707740052 | 1.7586371 | -11.50986724 | 3.71E-29 | 2.37E-28 | 54.76065496 |
| DHTKD1 | 0.794227896 | 3.801116234 | 11.50420854 | 3.94E-29 | 2.51E-28 | 54.70201085 |
| ARHGDIA | 0.6547622 | 6.131787764 | 11.50083742 | 4.08E-29 | 2.60E-28 | 54.6670852 |
| TRABD | 0.820932229 | 3.56270888 | 11.50044913 | 4.10E-29 | 2.61E-28 | 54.66306297 |
| TWIST1 | -1.090325765 | 2.171590656 | -11.49591476 | 4.30E-29 | 2.73E-28 | 54.61609991 |
| MBNL3 | -0.680846277 | 1.177433707 | -11.4885641 | 4.64E-29 | 2.95E-28 | 54.53999973 |
| POLR2K | 0.710623678 | 5.095708012 | 11.486247 | 4.75E-29 | 3.02E-28 | 54.51601931 |
| ACBD3 | 0.629836334 | 4.828252578 | 11.48556233 | 4.79E-29 | 3.04E-28 | 54.50893415 |
| PALM | -1.134521508 | 2.845305343 | -11.48507439 | 4.81E-29 | 3.05E-28 | 54.50388501 |
| C19orf53 | 0.672690002 | 5.835070283 | 11.48430269 | 4.85E-29 | 3.08E-28 | 54.49589993 |
| HIST1H1C | 1.776408085 | 6.150663364 | 11.48401077 | 4.86E-29 | 3.08E-28 | 54.49287943 |
| MRAP2 | -0.772187656 | 0.750396452 | -11.48353798 | 4.89E-29 | 3.10E-28 | 54.48798769 |
| SLC29A3 | 0.797812976 | 3.147600783 | 11.48239617 | 4.95E-29 | 3.13E-28 | 54.47617435 |
| SHISA5 | 0.592342549 | 5.313673062 | 11.48168465 | 4.98E-29 | 3.16E-28 | 54.46881334 |
| MRPL12 | 1.127116253 | 3.852091319 | 11.47567511 | 5.31E-29 | 3.36E-28 | 54.40665646 |
| CRELD2 | 0.743123323 | 2.987213893 | 11.47553193 | 5.31E-29 | 3.36E-28 | 54.40517579 |
| SCRN2 | -0.929179864 | 3.232400475 | -11.46932665 | 5.67E-29 | 3.58E-28 | 54.34102246 |
| NKAIN1 | 2.176240807 | 2.697264672 | 11.46912662 | 5.68E-29 | 3.59E-28 | 54.33895494 |
| NUDT1 | 0.780787767 | 2.868592536 | 11.46571603 | 5.88E-29 | 3.72E-28 | 54.30370686 |
| RARRES2 | -1.341746024 | 4.272168382 | -11.46506716 | 5.92E-29 | 3.74E-28 | 54.29700175 |
| ACADVL | -0.73939513 | 5.330076451 | -11.46201523 | 6.12E-29 | 3.86E-28 | 54.26546895 |
| LSM7 | 0.811528514 | 4.053442946 | 11.46080034 | 6.19E-29 | 3.91E-28 | 54.25291854 |
| NECTIN4 | 1.116776329 | 4.355728476 | 11.45930938 | 6.29E-29 | 3.96E-28 | 54.23751752 |
| CSGALNACT1 | -0.71507944 | 1.66210399 | -11.45634351 | 6.49E-29 | 4.09E-28 | 54.20688617 |
| PHF10 | -0.614101723 | 3.815420163 | -11.45410211 | 6.64E-29 | 4.18E-28 | 54.18374135 |
| BNIP3L | -0.75053268 | 4.904967241 | -11.44878414 | 7.02E-29 | 4.42E-28 | 54.12884224 |
| LTBP2 | -1.151496915 | 4.251419554 | -11.44811888 | 7.07E-29 | 4.45E-28 | 54.12197593 |
| NOP2 | 0.699220949 | 3.367291157 | 11.44434589 | 7.35E-29 | 4.62E-28 | 54.08304032 |
| ATP5MF | 0.659292178 | 5.167886187 | 11.44225941 | 7.51E-29 | 4.72E-28 | 54.06151321 |
| PTGDS | -1.643751928 | 2.735480978 | -11.43749273 | 7.89E-29 | 4.95E-28 | 54.01234522 |
| FAM49B | 0.680764192 | 3.345857456 | 11.43736529 | 7.90E-29 | 4.96E-28 | 54.01103091 |
| EFNA4 | 1.064426012 | 4.036365452 | 11.43349332 | 8.23E-29 | 5.16E-28 | 53.9711042 |
| DOCK9 | -0.679386325 | 2.600230045 | -11.42717373 | 8.79E-29 | 5.51E-28 | 53.90596157 |
| SPP1 | 1.933201008 | 5.781157147 | 11.42701639 | 8.80E-29 | 5.51E-28 | 53.90434009 |
| TUFM | 0.597905069 | 6.54662062 | 11.41954012 | 9.51E-29 | 5.96E-28 | 53.8273127 |
| FANCG | 0.607276939 | 2.752173044 | 11.4172068 | 9.74E-29 | 6.10E-28 | 53.80328106 |
| CLK1 | -0.719395438 | 3.915078773 | -11.40763778 | 1.08E-28 | 6.73E-28 | 53.70476751 |
| OLFM4 | -2.010625899 | 1.393096167 | -11.40685744 | 1.08E-28 | 6.78E-28 | 53.69673682 |
| ZMAT3 | -0.604899341 | 2.240123837 | -11.40564229 | 1.10E-28 | 6.86E-28 | 53.68423225 |
| PLN | -0.948543287 | 1.498408813 | -11.40001348 | 1.16E-28 | 7.27E-28 | 53.62632258 |
| LRRC56 | 0.956252521 | 1.960938764 | 11.40001074 | 1.16E-28 | 7.27E-28 | 53.62629442 |
| VARS | 0.611642231 | 4.018080558 | 11.39828325 | 1.19E-28 | 7.40E-28 | 53.60852654 |
| FOLR1 | -1.816441173 | 1.661265349 | -11.39385704 | 1.24E-28 | 7.74E-28 | 53.56301098 |
| GEM | -1.2114749 | 3.387350318 | -11.39375505 | 1.24E-28 | 7.74E-28 | 53.56196236 |
| GABRP | -2.731284666 | 2.828038563 | -11.39296544 | 1.25E-28 | 7.80E-28 | 53.55384422 |
| RHOT2 | 0.782451325 | 3.825899194 | 11.39123326 | 1.28E-28 | 7.94E-28 | 53.53603695 |
| RFXANK | 0.708325249 | 4.054370927 | 11.39063629 | 1.28E-28 | 7.99E-28 | 53.52990041 |
| EPS8L1 | 1.052237454 | 2.752749478 | 11.3870041 | 1.33E-28 | 8.29E-28 | 53.49256913 |
| RNASE1 | -1.211929469 | 5.885643193 | -11.38451726 | 1.37E-28 | 8.50E-28 | 53.46701517 |
| GRTP1 | 0.790485003 | 2.81877984 | 11.38424007 | 1.37E-28 | 8.52E-28 | 53.46416711 |
| ZNF395 | -0.757030788 | 3.402775809 | -11.38140343 | 1.41E-28 | 8.77E-28 | 53.43502478 |
| FAM180A | -0.755511893 | 1.10920943 | -11.37781395 | 1.47E-28 | 9.10E-28 | 53.39815642 |
| FLT1 | -0.689948991 | 2.32254046 | -11.37735114 | 1.47E-28 | 9.14E-28 | 53.39340353 |
| ARID5A | -1.051824267 | 3.45367478 | -11.37672979 | 1.48E-28 | 9.20E-28 | 53.38702266 |
| RAB3D | 0.843468354 | 3.901153984 | 11.37132567 | 1.57E-28 | 9.72E-28 | 53.33153771 |
| RIPPLY3 | 0.88001568 | 1.030956398 | 11.37116278 | 1.57E-28 | 9.73E-28 | 53.3298656 |
| TRAPPC9 | 0.642914202 | 3.173181174 | 11.36820297 | 1.62E-28 | 1.00E-27 | 53.29948618 |
| FBXL5 | -0.664307239 | 4.743910564 | -11.3675681 | 1.63E-28 | 1.01E-27 | 53.29297072 |
| SERF2 | 0.730023755 | 5.795127322 | 11.36658675 | 1.65E-28 | 1.02E-27 | 53.28290004 |
| POLD1 | 0.700997954 | 2.501136609 | 11.36447848 | 1.68E-28 | 1.04E-27 | 53.26126709 |
| TRO | -0.66360449 | 1.218760348 | -11.36284181 | 1.71E-28 | 1.06E-27 | 53.24447551 |
| E4F1 | 0.708767177 | 2.558596088 | 11.36172441 | 1.73E-28 | 1.07E-27 | 53.23301253 |
| SLC19A1 | 0.597753486 | 1.750023207 | 11.36000262 | 1.76E-28 | 1.09E-27 | 53.21535114 |
| SLC28A3 | -1.247307429 | 1.118761705 | -11.35313113 | 1.89E-28 | 1.17E-27 | 53.1448878 |
| MPPED2 | -0.595419623 | 0.553274397 | -11.35273128 | 1.90E-28 | 1.17E-27 | 53.14078859 |
| GCNT4 | -0.633888295 | 0.718493174 | -11.3526668 | 1.90E-28 | 1.17E-27 | 53.14012757 |
| TCF7L1 | -1.241164659 | 2.791261408 | -11.35010943 | 1.95E-28 | 1.20E-27 | 53.11391277 |
| PDLIM4 | -1.116354529 | 2.600086783 | -11.34877916 | 1.98E-28 | 1.22E-27 | 53.10027855 |
| HIST1H2AM | 0.953233046 | 1.011277456 | 11.34823546 | 1.99E-28 | 1.23E-27 | 53.09470639 |
| KIF13A | -0.689109671 | 2.972921038 | -11.34658679 | 2.02E-28 | 1.25E-27 | 53.07781114 |
| SLC25A10 | 0.875373292 | 3.083391426 | 11.34302534 | 2.10E-28 | 1.29E-27 | 53.04132095 |
| ATG101 | 0.614921546 | 3.137291612 | 11.32669786 | 2.49E-28 | 1.53E-27 | 52.87414977 |
| ARTN | 0.940127665 | 1.119343384 | 11.32589061 | 2.51E-28 | 1.54E-27 | 52.86588969 |
| L3MBTL4 | -0.636383398 | 0.646379256 | -11.32543962 | 2.52E-28 | 1.55E-27 | 52.86127516 |
| TCF4 | -0.738690205 | 2.084828761 | -11.32080581 | 2.64E-28 | 1.62E-27 | 52.8138709 |
| ZBTB38 | -0.654723017 | 2.747884227 | -11.31340318 | 2.85E-28 | 1.75E-27 | 52.73817384 |
| CTSD | 1.117983138 | 8.009634468 | 11.30746139 | 3.03E-28 | 1.86E-27 | 52.67744389 |
| PYCARD | 1.351335929 | 4.096958079 | 11.30394709 | 3.14E-28 | 1.93E-27 | 52.6415369 |
| ALAD | -0.644248759 | 3.805861456 | -11.30069006 | 3.25E-28 | 1.99E-27 | 52.60826669 |
| CHST11 | 0.787272563 | 2.389555222 | 11.29005111 | 3.63E-28 | 2.22E-27 | 52.4996448 |
| PTGER3 | -1.310283734 | 1.952871986 | -11.28916128 | 3.66E-28 | 2.24E-27 | 52.49056352 |
| ZFHX4 | -0.609054409 | 0.900047893 | -11.28711693 | 3.74E-28 | 2.29E-27 | 52.46970192 |
| TELO2 | 0.815241626 | 3.412105548 | 11.28642697 | 3.76E-28 | 2.30E-27 | 52.46266182 |
| C2CD4B | -0.944935546 | 0.829166169 | -11.2861571 | 3.77E-28 | 2.31E-27 | 52.4599083 |
| NME3 | 1.161876461 | 4.865885105 | 11.28530265 | 3.81E-28 | 2.33E-27 | 52.45119052 |
| PODXL2 | 1.301182243 | 4.086163438 | 11.2848494 | 3.82E-28 | 2.34E-27 | 52.44656635 |
| EIF5A | 0.607718528 | 6.785251247 | 11.2822051 | 3.93E-28 | 2.40E-27 | 52.41959142 |
| BBS10 | -0.593735083 | 2.602987984 | -11.28214955 | 3.93E-28 | 2.40E-27 | 52.41902476 |
| CAPS | 1.296555804 | 2.939484488 | 11.28116215 | 3.97E-28 | 2.43E-27 | 52.40895351 |
| NOC2L | 0.661786808 | 4.09185063 | 11.27935592 | 4.05E-28 | 2.47E-27 | 52.39053213 |
| RTN4RL2 | 0.67569584 | 1.373851233 | 11.27555063 | 4.21E-28 | 2.57E-27 | 52.35173055 |
| ZNF608 | -0.855543678 | 1.566334463 | -11.27420053 | 4.27E-28 | 2.60E-27 | 52.3379665 |
| FAT1 | -1.236115956 | 3.183984884 | -11.27242761 | 4.35E-28 | 2.65E-27 | 52.31989383 |
| SAP30 | 0.791663762 | 3.187546069 | 11.27104442 | 4.41E-28 | 2.69E-27 | 52.30579559 |
| RPS25 | -0.69229786 | 8.058269315 | -11.26825524 | 4.54E-28 | 2.76E-27 | 52.27737099 |
| GAS7 | -0.949666842 | 2.365822034 | -11.26217054 | 4.83E-28 | 2.94E-27 | 52.21538137 |
| NTHL1 | 0.881059545 | 2.842764259 | 11.25675922 | 5.10E-28 | 3.10E-27 | 52.16027478 |
| APOBEC3B | 1.270737542 | 2.105998993 | 11.25403099 | 5.25E-28 | 3.19E-27 | 52.13249982 |
| PTGES2 | 0.646609022 | 3.667028887 | 11.24480121 | 5.77E-28 | 3.50E-27 | 52.03857548 |
| DCBLD2 | -0.647772003 | 2.080606704 | -11.23697112 | 6.25E-28 | 3.79E-27 | 51.9589436 |
| PROCR | -0.820283407 | 3.219169779 | -11.23364367 | 6.47E-28 | 3.92E-27 | 51.92511715 |
| PPM1J | 1.030136969 | 1.869841534 | 11.23334701 | 6.49E-28 | 3.93E-27 | 51.92210178 |
| PNP | 0.785553824 | 3.432198625 | 11.23294858 | 6.52E-28 | 3.94E-27 | 51.91805197 |
| GUCY1A1 | -1.076566982 | 2.660530014 | -11.23221918 | 6.56E-28 | 3.97E-27 | 51.91063846 |
| GOLT1A | 1.048097435 | 2.485387733 | 11.22340014 | 7.19E-28 | 4.34E-27 | 51.82103352 |
| PRR7 | 0.81494754 | 1.380234299 | 11.21464255 | 7.86E-28 | 4.74E-27 | 51.7321094 |
| CYSRT1 | 0.820645054 | 1.22870734 | 11.21256573 | 8.03E-28 | 4.84E-27 | 51.71102975 |
| STX19 | -0.813536363 | 0.726883752 | -11.21070646 | 8.18E-28 | 4.94E-27 | 51.69216088 |
| ARHGEF37 | -0.986091213 | 2.822147859 | -11.20600132 | 8.59E-28 | 5.18E-27 | 51.64442188 |
| CLPSL2 | 1.123371455 | 1.310596713 | 11.20555582 | 8.62E-28 | 5.20E-27 | 51.63990269 |
| SLC2A12 | -0.644416225 | 0.570172722 | -11.20353307 | 8.81E-28 | 5.30E-27 | 51.61938525 |
| ROMO1 | 0.943865858 | 5.685892488 | 11.20229081 | 8.92E-28 | 5.37E-27 | 51.60678606 |
| RBBP7 | 0.68750687 | 4.604063724 | 11.19352696 | 9.75E-28 | 5.87E-27 | 51.51793416 |
| HEY2 | -1.108775468 | 1.830048616 | -11.19286025 | 9.82E-28 | 5.91E-27 | 51.51117708 |
| SRSF8 | -0.612960449 | 3.761713018 | -11.19141678 | 9.97E-28 | 5.99E-27 | 51.49654864 |
| TEAD4 | 0.811708412 | 2.889914246 | 11.18951308 | 1.02E-27 | 6.10E-27 | 51.47725851 |
| JAK1 | -0.660182266 | 5.071222198 | -11.18697051 | 1.04E-27 | 6.26E-27 | 51.45149883 |
| KIAA1522 | 0.782939745 | 5.284652496 | 11.18692118 | 1.04E-27 | 6.26E-27 | 51.45099904 |
| PKP1 | -1.712721922 | 2.011816141 | -11.18531568 | 1.06E-27 | 6.36E-27 | 51.43473566 |
| MYO1C | -0.641521036 | 5.058525582 | -11.18158197 | 1.10E-27 | 6.61E-27 | 51.39692127 |
| ZFYVE9 | -0.716252446 | 2.602202551 | -11.18040987 | 1.12E-27 | 6.68E-27 | 51.38505259 |
| GPSM2 | 0.71413847 | 1.893893455 | 11.17489254 | 1.18E-27 | 7.07E-27 | 51.3291975 |
| SERPINF1 | -1.22067207 | 5.668631563 | -11.17328815 | 1.20E-27 | 7.18E-27 | 51.31295952 |
| HOXD9 | -0.785216549 | 1.811889988 | -11.15711526 | 1.41E-27 | 8.46E-27 | 51.1493801 |
| POLR2J | 0.700653688 | 4.494304886 | 11.15336921 | 1.47E-27 | 8.78E-27 | 51.11151845 |
| IMPDH1 | 0.733478942 | 4.422900005 | 11.15331464 | 1.47E-27 | 8.78E-27 | 51.11096701 |
| OLFML2B | 1.04363365 | 4.111137212 | 11.14931488 | 1.53E-27 | 9.14E-27 | 51.07055256 |
| RAB7B | -0.89635531 | 1.689252941 | -11.1426239 | 1.64E-27 | 9.78E-27 | 51.00297197 |
| PMVK | 0.817868718 | 5.546642082 | 11.14071353 | 1.67E-27 | 9.97E-27 | 50.98368279 |
| SAMD8 | -0.682655034 | 2.644392353 | -11.14042623 | 1.68E-27 | 1.00E-26 | 50.98078204 |
| PDK2 | -0.736713328 | 2.486973824 | -11.13471239 | 1.78E-27 | 1.06E-26 | 50.92310616 |
| TMEM184A | 0.826892791 | 1.810073368 | 11.13003325 | 1.86E-27 | 1.11E-26 | 50.87589243 |
| HPN | 1.551197498 | 3.20172895 | 11.12822033 | 1.90E-27 | 1.13E-26 | 50.85760401 |
| DSC3 | -1.520102851 | 1.495876367 | -11.12819101 | 1.90E-27 | 1.13E-26 | 50.85730823 |
| LRRC1 | 0.669088373 | 3.253221734 | 11.12600746 | 1.94E-27 | 1.16E-26 | 50.83528411 |
| CTF1 | -1.033693978 | 2.08980416 | -11.12219004 | 2.02E-27 | 1.20E-26 | 50.79678875 |
| NFIL3 | -1.041856007 | 3.483641486 | -11.12112779 | 2.04E-27 | 1.21E-26 | 50.78607871 |
| RHOD | 0.918787388 | 4.340103824 | 11.12022131 | 2.06E-27 | 1.22E-26 | 50.77693995 |
| EPRS | 0.665629386 | 5.443479444 | 11.1200244 | 2.06E-27 | 1.23E-26 | 50.77495489 |
| CYC1 | 0.79428212 | 5.785971395 | 11.11938038 | 2.08E-27 | 1.23E-26 | 50.76846254 |
| HRCT1 | -1.489423343 | 1.488555845 | -11.11835988 | 2.10E-27 | 1.25E-26 | 50.7581756 |
| NR1H3 | -0.677638392 | 2.70893014 | -11.11363595 | 2.20E-27 | 1.31E-26 | 50.71056704 |
| PIK3CA | -0.588022004 | 2.341816869 | -11.11263156 | 2.22E-27 | 1.32E-26 | 50.70044668 |
| PCDHB7 | -0.586057502 | 0.790676083 | -11.11188729 | 2.24E-27 | 1.33E-26 | 50.69294787 |
| ME3 | -0.861299486 | 1.956993451 | -11.10547814 | 2.39E-27 | 1.42E-26 | 50.62838988 |
| ARV1 | 0.634961257 | 3.934394224 | 11.09767333 | 2.59E-27 | 1.53E-26 | 50.54981473 |
| GUK1 | 0.749620969 | 5.093121551 | 11.09562511 | 2.64E-27 | 1.56E-26 | 50.52920161 |
| BEND7 | -0.735746618 | 1.046070129 | -11.09428438 | 2.68E-27 | 1.58E-26 | 50.51571032 |
| MFAP5 | -1.415119568 | 3.444549872 | -11.0913401 | 2.76E-27 | 1.63E-26 | 50.4860877 |
| CSE1L | 0.677605162 | 5.321745655 | 11.08968012 | 2.81E-27 | 1.66E-26 | 50.46938937 |
| RPS6KA1 | 0.673179842 | 3.343926608 | 11.08535142 | 2.93E-27 | 1.73E-26 | 50.42585503 |
| SWAP70 | -0.621263144 | 3.65979038 | -11.08083503 | 3.07E-27 | 1.81E-26 | 50.38044775 |
| AIF1L | -1.369155683 | 3.173265664 | -11.07164655 | 3.37E-27 | 1.98E-26 | 50.28811433 |
| PGK1 | 0.698276865 | 6.224757071 | 11.06727559 | 3.52E-27 | 2.07E-26 | 50.2442132 |
| CORO1B | 0.763479461 | 4.358985886 | 11.06618746 | 3.56E-27 | 2.09E-26 | 50.23328643 |
| RANBP1 | 0.626101968 | 3.695368917 | 11.06453435 | 3.62E-27 | 2.13E-26 | 50.21668791 |
| ELF5 | -1.933499813 | 1.90594021 | -11.06231896 | 3.70E-27 | 2.18E-26 | 50.19444685 |
| PM20D2 | -1.091561792 | 1.697320233 | -11.06145174 | 3.74E-27 | 2.19E-26 | 50.18574157 |
| ACSS3 | -0.809872619 | 1.295708617 | -11.05684254 | 3.91E-27 | 2.30E-26 | 50.13948279 |
| WDR90 | 0.749243285 | 2.125473835 | 11.05678183 | 3.92E-27 | 2.30E-26 | 50.13887355 |
| MST1R | 0.877798355 | 2.094336302 | 11.05157656 | 4.13E-27 | 2.42E-26 | 50.08665163 |
| JCHAIN | -2.542915537 | 5.529283989 | -11.05125521 | 4.14E-27 | 2.43E-26 | 50.08342841 |
| MRPL28 | 0.74041493 | 4.399556067 | 11.05085296 | 4.16E-27 | 2.44E-26 | 50.07939369 |
| OSCAR | 0.719750625 | 1.651289484 | 11.05061212 | 4.17E-27 | 2.44E-26 | 50.07697815 |
| TIGD5 | 0.639983681 | 1.881727809 | 11.04083836 | 4.60E-27 | 2.69E-26 | 49.97898401 |
| TSPAN2 | -0.784058267 | 1.646013294 | -11.04046621 | 4.62E-27 | 2.70E-26 | 49.97525414 |
| HIST1H2AI | 1.386126846 | 1.449425702 | 11.03705269 | 4.78E-27 | 2.80E-26 | 49.94104694 |
| FAM129A | -1.30252964 | 3.516309542 | -11.03410995 | 4.93E-27 | 2.88E-26 | 49.91156439 |
| RHBDD2 | 0.656896495 | 5.332916697 | 11.03163086 | 5.05E-27 | 2.95E-26 | 49.88673198 |
| CXorf40A | 0.642868883 | 2.555497262 | 11.02973906 | 5.15E-27 | 3.01E-26 | 49.8677854 |
| PPIB | 0.627902944 | 6.696719073 | 11.02948488 | 5.16E-27 | 3.01E-26 | 49.86523997 |
| RAC3 | 1.003177308 | 3.531619494 | 11.02944066 | 5.16E-27 | 3.01E-26 | 49.86479715 |
| PRSS12 | -0.819989219 | 0.627134347 | -11.02899306 | 5.19E-27 | 3.03E-26 | 49.86031487 |
| PTAR1 | -0.606784908 | 2.216395599 | -11.02785086 | 5.25E-27 | 3.06E-26 | 49.84887758 |
| HPD | -0.645624248 | 0.32463429 | -11.02747626 | 5.27E-27 | 3.07E-26 | 49.84512672 |
| GNG12 | -0.937322201 | 4.592912572 | -11.02502634 | 5.40E-27 | 3.15E-26 | 49.82059857 |
| DYNC2H1 | -0.679829838 | 1.355854895 | -11.02239215 | 5.54E-27 | 3.23E-26 | 49.79423046 |
| ZBTB44 | -0.602485284 | 2.747522444 | -11.0207871 | 5.64E-27 | 3.28E-26 | 49.77816653 |
| POLD2 | 0.609027859 | 4.728601919 | 11.01969141 | 5.70E-27 | 3.32E-26 | 49.7672016 |
| AMIGO2 | -1.46375138 | 3.275733627 | -11.01773013 | 5.81E-27 | 3.38E-26 | 49.74757656 |
| CMTM7 | -1.09709453 | 2.613455554 | -11.01737304 | 5.83E-27 | 3.39E-26 | 49.74400369 |
| AGTRAP | 0.687146183 | 4.384013164 | 11.01694599 | 5.86E-27 | 3.41E-26 | 49.73973104 |
| DLL4 | -0.621207625 | 1.942230222 | -11.01100866 | 6.22E-27 | 3.61E-26 | 49.6803416 |
| CKAP5 | 0.648037597 | 4.278443965 | 11.01011511 | 6.28E-27 | 3.64E-26 | 49.67140589 |
| GLO1 | 0.623410725 | 6.155262623 | 11.00844461 | 6.38E-27 | 3.70E-26 | 49.65470222 |
| CPE | -1.263628103 | 4.951845332 | -11.0050971 | 6.60E-27 | 3.83E-26 | 49.62123587 |
| SMPD2 | 0.631563605 | 3.024764909 | 11.00086479 | 6.89E-27 | 4.00E-26 | 49.57893572 |
| ID1 | -1.082758301 | 3.303509682 | -10.99783952 | 7.10E-27 | 4.12E-26 | 49.54870764 |
| S100A11 | 0.990194542 | 9.252706608 | 10.99340566 | 7.43E-27 | 4.30E-26 | 49.50441736 |
| FAM13B | -0.633411241 | 2.738809303 | -10.98857314 | 7.80E-27 | 4.52E-26 | 49.45616143 |
| TJP1 | -0.586550275 | 3.224538972 | -10.98593724 | 8.01E-27 | 4.64E-26 | 49.4298475 |
| C2orf15 | 0.588204473 | 1.792437926 | 10.9844531 | 8.13E-27 | 4.70E-26 | 49.41503379 |
| NFATC2 | -0.714542464 | 1.871807574 | -10.97150525 | 9.26E-27 | 5.35E-26 | 49.28586598 |
| TCF19 | 0.850882826 | 3.134327966 | 10.96385574 | 1.00E-26 | 5.78E-26 | 49.20961307 |
| CBLC | 1.190800493 | 3.409789975 | 10.96081227 | 1.03E-26 | 5.95E-26 | 49.17928673 |
| VWA5A | -0.925145186 | 2.57981031 | -10.95855658 | 1.05E-26 | 6.09E-26 | 49.1568147 |
| LAYN | -0.649216759 | 1.73656197 | -10.95764851 | 1.06E-26 | 6.14E-26 | 49.14776919 |
| PJA1 | -0.586212305 | 3.480141887 | -10.95628522 | 1.08E-26 | 6.22E-26 | 49.13419029 |
| COLCA2 | -0.58894435 | 0.609663198 | -10.95520078 | 1.09E-26 | 6.29E-26 | 49.12338992 |
| RHBDL1 | 0.969012884 | 1.382478232 | 10.95267396 | 1.12E-26 | 6.45E-26 | 49.0982275 |
| MOSPD3 | 0.633092193 | 3.514534469 | 10.94768603 | 1.18E-26 | 6.78E-26 | 49.04857098 |
| TIMM10 | 0.66770741 | 4.904787096 | 10.94658561 | 1.19E-26 | 6.85E-26 | 49.03761844 |
| SECISBP2L | -0.689137623 | 2.973327027 | -10.94413492 | 1.22E-26 | 7.01E-26 | 49.01322981 |
| SEMA7A | 0.69088463 | 1.582173804 | 10.94318382 | 1.23E-26 | 7.08E-26 | 49.00376592 |
| CDK2AP2 | 0.77850773 | 4.56501114 | 10.93936059 | 1.28E-26 | 7.35E-26 | 48.96572977 |
| COX17 | 0.667221995 | 3.753862144 | 10.93682042 | 1.31E-26 | 7.53E-26 | 48.9404644 |
| GIMAP4 | -0.923083785 | 3.73850911 | -10.93581478 | 1.32E-26 | 7.61E-26 | 48.93046327 |
| IL21R | 0.650189949 | 0.789477636 | 10.93405995 | 1.35E-26 | 7.74E-26 | 48.91301324 |
| GNG5 | 0.628026198 | 6.138805855 | 10.92245612 | 1.51E-26 | 8.68E-26 | 48.79768277 |
| HERC1 | -0.655786772 | 2.552843656 | -10.92070358 | 1.54E-26 | 8.82E-26 | 48.78027294 |
| GSTA1 | -1.452290716 | 0.894538696 | -10.9172927 | 1.60E-26 | 9.13E-26 | 48.74639577 |
| MYOM2 | -0.614809818 | 0.342988366 | -10.91681081 | 1.60E-26 | 9.17E-26 | 48.74161024 |
| ZNHIT2 | 0.865136505 | 3.152059117 | 10.9074224 | 1.76E-26 | 1.01E-25 | 48.64841172 |
| SLC4A2 | 0.598067565 | 3.619903885 | 10.90663358 | 1.77E-26 | 1.01E-25 | 48.64058418 |
| CDC42EP3 | -0.819650335 | 2.124979338 | -10.90124516 | 1.87E-26 | 1.07E-25 | 48.58712625 |
| PJA2 | -0.726574099 | 4.770356944 | -10.89832452 | 1.93E-26 | 1.10E-25 | 48.55816001 |
| HMGB2 | 0.890584127 | 5.310366086 | 10.89679554 | 1.96E-26 | 1.12E-25 | 48.54299849 |
| PLCG2 | -0.689035942 | 1.399267237 | -10.89527666 | 1.99E-26 | 1.14E-25 | 48.52793884 |
| LEO1 | 0.664720361 | 3.953377565 | 10.89382632 | 2.02E-26 | 1.15E-25 | 48.5135603 |
| CENPN | 0.721890154 | 1.798368169 | 10.89225557 | 2.05E-26 | 1.17E-25 | 48.49798991 |
| NFIX | -1.139181179 | 4.376959816 | -10.89163204 | 2.06E-26 | 1.18E-25 | 48.49180949 |
| SDK1 | -0.623505524 | 1.017889554 | -10.88515905 | 2.20E-26 | 1.25E-25 | 48.42766688 |
| HSDL2 | -0.754737324 | 4.119502578 | -10.8833652 | 2.24E-26 | 1.28E-25 | 48.40989662 |
| HIST1H2BG | 1.553273804 | 2.610780333 | 10.87244479 | 2.50E-26 | 1.42E-25 | 48.30176887 |
| RPL5 | -0.66035886 | 8.040191709 | -10.87141251 | 2.52E-26 | 1.43E-25 | 48.29155243 |
| AMIGO1 | -0.611002836 | 1.776118795 | -10.85914337 | 2.85E-26 | 1.62E-25 | 48.170186 |
| ARHGEF25 | -0.652662925 | 2.316791946 | -10.85812155 | 2.88E-26 | 1.64E-25 | 48.16008321 |
| KCNK6 | 1.029524395 | 3.097782953 | 10.85515536 | 2.97E-26 | 1.68E-25 | 48.13076083 |
| KRT17 | -2.665484275 | 4.366279013 | -10.85337203 | 3.02E-26 | 1.71E-25 | 48.1131348 |
| LMO3 | -0.754568221 | 0.608463983 | -10.85131112 | 3.08E-26 | 1.75E-25 | 48.09276824 |
| BOK | -0.922888867 | 3.855012535 | -10.85108367 | 3.09E-26 | 1.75E-25 | 48.09052065 |
| KIAA1211 | 0.694279918 | 0.86275733 | 10.8408528 | 3.42E-26 | 1.94E-25 | 47.98946462 |
| CCDC24 | 0.829278653 | 2.451374984 | 10.83182455 | 3.74E-26 | 2.12E-25 | 47.90035255 |
| PLIN2 | -0.976056965 | 3.519612793 | -10.82602443 | 3.97E-26 | 2.24E-25 | 47.84313549 |
| FBXO41 | 0.605847787 | 1.604659091 | 10.82414748 | 4.04E-26 | 2.28E-25 | 47.82462514 |
| TEDC1 | 0.631549136 | 1.38282298 | 10.82335749 | 4.07E-26 | 2.30E-25 | 47.81683517 |
| LAMC2 | -1.597241903 | 3.043092834 | -10.82332354 | 4.07E-26 | 2.30E-25 | 47.81650035 |
| HBP1 | -0.617498982 | 3.564166557 | -10.8200802 | 4.21E-26 | 2.38E-25 | 47.78452296 |
| PRKAA1 | -0.736515724 | 3.245534717 | -10.8191995 | 4.25E-26 | 2.40E-25 | 47.77584119 |
| CNTN1 | -0.926714095 | 1.303852467 | -10.81845627 | 4.28E-26 | 2.41E-25 | 47.76851502 |
| SCAF1 | 0.629861298 | 4.160953006 | 10.81809174 | 4.29E-26 | 2.42E-25 | 47.76492191 |
| RPL9 | -0.684435093 | 6.979009513 | -10.8180316 | 4.29E-26 | 2.42E-25 | 47.76432906 |
| ANKRD40 | -0.67380256 | 3.75085096 | -10.81615406 | 4.38E-26 | 2.47E-25 | 47.7458242 |
| MMP19 | -0.77493362 | 1.869728853 | -10.81479106 | 4.44E-26 | 2.50E-25 | 47.73239222 |
| OXCT1 | -0.906079071 | 3.193194139 | -10.81442875 | 4.45E-26 | 2.51E-25 | 47.72882192 |
| FBXO17 | -0.747715114 | 1.370160117 | -10.80396507 | 4.94E-26 | 2.78E-25 | 47.62575452 |
| NDUFB10 | 0.685498587 | 6.057459647 | 10.80296267 | 4.99E-26 | 2.81E-25 | 47.6158851 |
| SLC16A11 | -0.5856753 | 0.565261258 | -10.80240561 | 5.02E-26 | 2.82E-25 | 47.61040076 |
| CEP126 | -0.808986222 | 1.24752507 | -10.79945449 | 5.17E-26 | 2.91E-25 | 47.58135047 |
| VEGFC | -0.663760681 | 2.32321668 | -10.79564615 | 5.36E-26 | 3.01E-25 | 47.54387158 |
| SRPK1 | 0.687819856 | 3.478390714 | 10.79463135 | 5.42E-26 | 3.04E-25 | 47.53388642 |
| CALD1 | -0.921827992 | 4.837399014 | -10.79390031 | 5.46E-26 | 3.07E-25 | 47.52669389 |
| SEMA3F | 0.898615673 | 4.292360389 | 10.79028537 | 5.66E-26 | 3.18E-25 | 47.49113303 |
| TRNP1 | -0.915867486 | 2.055508346 | -10.78206576 | 6.14E-26 | 3.44E-25 | 47.41031164 |
| APOBEC3C | -0.955467012 | 3.32574628 | -10.77975306 | 6.28E-26 | 3.52E-25 | 47.38758061 |
| CNTD2 | 1.252642748 | 1.666696704 | 10.77811923 | 6.38E-26 | 3.58E-25 | 47.37152443 |
| DOCK1 | -0.689175643 | 3.565938997 | -10.77553372 | 6.55E-26 | 3.66E-25 | 47.34611989 |
| CX3CR1 | -1.112367789 | 1.749384136 | -10.77498746 | 6.58E-26 | 3.68E-25 | 47.34075313 |
| MPG | 0.701505374 | 4.010945433 | 10.77083747 | 6.86E-26 | 3.84E-25 | 47.29998867 |
| FOXS1 | 0.700658903 | 1.602145939 | 10.76379593 | 7.36E-26 | 4.11E-25 | 47.23085072 |
| UNC93B1 | 0.744764136 | 3.617270363 | 10.76298334 | 7.42E-26 | 4.14E-25 | 47.22287457 |
| FYCO1 | -0.680190245 | 3.120733924 | -10.75883303 | 7.73E-26 | 4.31E-25 | 47.18214445 |
| SOD2 | -0.929148025 | 3.896759535 | -10.75803961 | 7.79E-26 | 4.35E-25 | 47.17435945 |
| GPS1 | 0.602126965 | 4.083595266 | 10.75789365 | 7.80E-26 | 4.35E-25 | 47.17292731 |
| RABL6 | 0.616653595 | 3.341550027 | 10.75628019 | 7.92E-26 | 4.42E-25 | 47.15709782 |
| RASSF6 | -0.957139254 | 1.284776253 | -10.75338228 | 8.15E-26 | 4.55E-25 | 47.1286717 |
| EXOSC5 | 0.699958001 | 3.883074585 | 10.74227217 | 9.10E-26 | 5.07E-25 | 47.01974882 |
| TMEM208 | 0.657175658 | 4.142106041 | 10.74156973 | 9.16E-26 | 5.10E-25 | 47.0128653 |
| HLA-E | -0.873566602 | 7.439826225 | -10.73254496 | 1.00E-25 | 5.57E-25 | 46.92446017 |
| VPS28 | 0.747129992 | 4.985806342 | 10.73132401 | 1.01E-25 | 5.64E-25 | 46.91250466 |
| TIMM13 | 0.801373852 | 4.571067408 | 10.72730797 | 1.06E-25 | 5.86E-25 | 46.87318765 |
| SIGIRR | 0.890434036 | 3.574729182 | 10.72695633 | 1.06E-25 | 5.88E-25 | 46.86974565 |
| C3orf58 | -0.855731872 | 2.235863281 | -10.72573157 | 1.07E-25 | 5.95E-25 | 46.85775801 |
| MAD2L2 | 0.699011573 | 3.166953122 | 10.71188086 | 1.23E-25 | 6.81E-25 | 46.72226905 |
| BACE1 | -0.587750728 | 2.66071326 | -10.70926249 | 1.26E-25 | 6.99E-25 | 46.69667222 |
| VCAN | 1.335157758 | 4.390707902 | 10.70534287 | 1.31E-25 | 7.26E-25 | 46.65836411 |
| TICRR | 0.633786131 | 0.959152165 | 10.70311903 | 1.34E-25 | 7.42E-25 | 46.63663477 |
| WSB1 | -0.600407708 | 3.214175772 | -10.70190185 | 1.36E-25 | 7.50E-25 | 46.62474315 |
| HIST1H2BJ | 1.116914551 | 1.695089139 | 10.70070878 | 1.37E-25 | 7.59E-25 | 46.61308814 |
| SRC | 0.676427209 | 3.327468048 | 10.70008487 | 1.38E-25 | 7.63E-25 | 46.60699367 |
| OXGR1 | -0.661445835 | 0.455700622 | -10.69878911 | 1.40E-25 | 7.73E-25 | 46.5943373 |
| PLPPR2 | 0.697468463 | 3.696126307 | 10.69009173 | 1.52E-25 | 8.40E-25 | 46.50941856 |
| CXorf40B | 0.620612114 | 3.342807955 | 10.68966315 | 1.53E-25 | 8.43E-25 | 46.50523542 |
| NOC4L | 0.613132972 | 3.382542265 | 10.6861635 | 1.58E-25 | 8.72E-25 | 46.47108308 |
| C1orf122 | 0.723296904 | 3.944940924 | 10.68286553 | 1.64E-25 | 9.01E-25 | 46.43890731 |
| ANKRD13D | 0.660266711 | 2.718384872 | 10.67832763 | 1.71E-25 | 9.42E-25 | 46.39464788 |
| ITPR1 | -1.090583794 | 2.683090493 | -10.67650147 | 1.74E-25 | 9.58E-25 | 46.3768413 |
| ZMYND10 | 1.145168178 | 1.752499665 | 10.67375833 | 1.79E-25 | 9.84E-25 | 46.35009802 |
| ATAD3A | 0.763356505 | 2.827750589 | 10.67306053 | 1.80E-25 | 9.90E-25 | 46.34329595 |
| RGS10 | 0.798177131 | 4.876695877 | 10.66546828 | 1.94E-25 | 1.07E-24 | 46.26931158 |
| LRFN1 | 0.694453173 | 1.366613622 | 10.66403769 | 1.97E-25 | 1.08E-24 | 46.25537573 |
| ZEB1 | -0.812298692 | 2.278832722 | -10.66299456 | 1.99E-25 | 1.09E-24 | 46.24521524 |
| GRB2 | 0.607475743 | 5.537606051 | 10.65997702 | 2.05E-25 | 1.12E-24 | 46.21582792 |
| CASP10 | -0.591079976 | 1.263017535 | -10.64961428 | 2.27E-25 | 1.24E-24 | 46.11495919 |
| CNKSR1 | 0.720914461 | 2.951905554 | 10.6492448 | 2.28E-25 | 1.25E-24 | 46.11136425 |
| PSMA7 | 0.675602782 | 6.390729657 | 10.64559893 | 2.36E-25 | 1.29E-24 | 46.0758964 |
| FGFR1 | -1.094724567 | 3.375649385 | -10.64273852 | 2.43E-25 | 1.33E-24 | 46.04807682 |
| MZT2B | 1.09424991 | 4.834044584 | 10.63861907 | 2.53E-25 | 1.38E-24 | 46.00802295 |
| IRX5 | 0.963985116 | 4.099262383 | 10.63396366 | 2.64E-25 | 1.44E-24 | 45.96277325 |
| E2F5 | 0.629584535 | 1.741541334 | 10.62949642 | 2.76E-25 | 1.51E-24 | 45.91936801 |
| ELK3 | -0.638662606 | 3.845759692 | -10.6278184 | 2.81E-25 | 1.53E-24 | 45.90306775 |
| CNR1 | -0.587892016 | 0.426409577 | -10.62745744 | 2.82E-25 | 1.54E-24 | 45.8995616 |
| HIST1H2AC | 1.255672371 | 5.494751099 | 10.6205978 | 3.01E-25 | 1.64E-24 | 45.83295096 |
| TMC4 | 1.234203325 | 4.80462376 | 10.62016199 | 3.03E-25 | 1.65E-24 | 45.82872022 |
| CD59 | -0.809133026 | 6.447569979 | -10.61347542 | 3.23E-25 | 1.76E-24 | 45.76382668 |
| ERMP1 | 0.846479919 | 4.049487839 | 10.60123062 | 3.64E-25 | 1.98E-24 | 45.64507801 |
| DKC1 | 0.58856184 | 4.328398324 | 10.59263985 | 3.96E-25 | 2.15E-24 | 45.56183357 |
| FNDC1 | 1.31177513 | 3.045540394 | 10.58880082 | 4.11E-25 | 2.23E-24 | 45.52465146 |
| MAOB | -2.008875303 | 4.112616366 | -10.58290301 | 4.36E-25 | 2.36E-24 | 45.46755133 |
| SCRIB | 0.841492208 | 3.989514551 | 10.57906531 | 4.52E-25 | 2.45E-24 | 45.43041051 |
| SLC25A37 | -0.851959511 | 2.526216088 | -10.57902396 | 4.53E-25 | 2.45E-24 | 45.43001039 |
| DSCC1 | 0.843240975 | 2.12712558 | 10.57853288 | 4.55E-25 | 2.46E-24 | 45.4252586 |
| MUCL1 | -3.431865518 | 3.894862093 | -10.57661486 | 4.63E-25 | 2.51E-24 | 45.40670118 |
| PNKP | 0.606800941 | 2.939557727 | 10.5766057 | 4.63E-25 | 2.51E-24 | 45.40661256 |
| FDPS | 0.601825109 | 4.583005501 | 10.57496342 | 4.71E-25 | 2.55E-24 | 45.39072522 |
| FBXO32 | -1.106680865 | 4.084219009 | -10.57437634 | 4.74E-25 | 2.56E-24 | 45.38504631 |
| SLC9A6 | -0.642529352 | 1.78982257 | -10.57409368 | 4.75E-25 | 2.57E-24 | 45.38231218 |
| ERAP1 | -0.713714588 | 3.400295386 | -10.57139149 | 4.88E-25 | 2.63E-24 | 45.35617758 |
| C3 | -1.376856581 | 5.617292295 | -10.57118434 | 4.89E-25 | 2.64E-24 | 45.35417433 |
| BAIAP2 | 0.71663011 | 2.747014996 | 10.56592686 | 5.14E-25 | 2.78E-24 | 45.30334263 |
| STEAP4 | -1.517936851 | 2.264919829 | -10.55999302 | 5.45E-25 | 2.94E-24 | 45.24599673 |
| LGALS3 | -1.037975803 | 5.97890132 | -10.55761377 | 5.58E-25 | 3.01E-24 | 45.2230107 |
| PKN3 | -0.639231421 | 2.065577429 | -10.54888774 | 6.07E-25 | 3.27E-24 | 45.13874489 |
| MARVELD2 | 0.745060897 | 2.879832646 | 10.54795366 | 6.13E-25 | 3.30E-24 | 45.12972802 |
| HOMER3 | 0.879107762 | 3.099821657 | 10.5357655 | 6.90E-25 | 3.71E-24 | 45.01213449 |
| TPT1 | -0.618083932 | 8.258912517 | -10.53490232 | 6.96E-25 | 3.74E-24 | 45.00381064 |
| JRKL | -0.719869997 | 2.096940311 | -10.53169082 | 7.18E-25 | 3.86E-24 | 44.97284641 |
| BSPRY | 1.006168908 | 4.338509956 | 10.53041062 | 7.27E-25 | 3.91E-24 | 44.96050538 |
| PPAT | 0.607199575 | 2.423270535 | 10.52586133 | 7.60E-25 | 4.08E-24 | 44.91666052 |
| VWA1 | 0.868097975 | 4.201178389 | 10.51806294 | 8.19E-25 | 4.39E-24 | 44.84153827 |
| TFAP2A | 0.934699631 | 3.828433977 | 10.51615596 | 8.35E-25 | 4.47E-24 | 44.82317526 |
| SHROOM2 | 0.66306378 | 1.722117445 | 10.51485628 | 8.45E-25 | 4.53E-24 | 44.81066184 |
| RAB31 | 1.16908755 | 4.678726642 | 10.51003449 | 8.86E-25 | 4.74E-24 | 44.76424824 |
| FAM241B | 0.743035406 | 3.112346931 | 10.50189485 | 9.59E-25 | 5.13E-24 | 44.68593789 |
| MYBPC1 | -1.696331625 | 1.122313826 | -10.50079731 | 9.69E-25 | 5.18E-24 | 44.67538251 |
| DSE | -0.603352625 | 1.73930555 | -10.4994019 | 9.82E-25 | 5.25E-24 | 44.66196367 |
| SUCO | 0.719796504 | 3.86163654 | 10.49782669 | 9.97E-25 | 5.33E-24 | 44.64681755 |
| HIST1H2AE | 1.508789254 | 2.841696864 | 10.49704072 | 1.01E-24 | 5.37E-24 | 44.63926097 |
| PRDX4 | 0.700964545 | 5.397737442 | 10.49306429 | 1.04E-24 | 5.57E-24 | 44.60103738 |
| ANO9 | 0.781968632 | 1.743783195 | 10.49036058 | 1.07E-24 | 5.72E-24 | 44.57505473 |
| PUF60 | 0.716056513 | 4.94184664 | 10.48858195 | 1.09E-24 | 5.82E-24 | 44.55796513 |
| BOLA1 | 0.736750869 | 3.591130237 | 10.48787881 | 1.10E-24 | 5.85E-24 | 44.55120981 |
| LRRC46 | 0.827517085 | 1.348908044 | 10.48631642 | 1.12E-24 | 5.94E-24 | 44.53620068 |
| MMP3 | 1.387436702 | 2.296655131 | 10.48108086 | 1.17E-24 | 6.24E-24 | 44.48591891 |
| GOLM1 | 1.081117852 | 5.618601564 | 10.47324 | 1.27E-24 | 6.72E-24 | 44.41065528 |
| KPTN | 0.614682163 | 2.513548965 | 10.47273274 | 1.27E-24 | 6.75E-24 | 44.4057877 |
| NCMAP | -0.95178529 | 0.807977092 | -10.46646564 | 1.35E-24 | 7.17E-24 | 44.34566657 |
| EFNA3 | 1.144099614 | 2.746536058 | 10.45519548 | 1.51E-24 | 7.99E-24 | 44.23762585 |
| OVOL1 | 0.747301794 | 1.764960163 | 10.44933221 | 1.60E-24 | 8.45E-24 | 44.18145627 |
| SLC4A4 | -0.820771635 | 0.453641697 | -10.44927236 | 1.60E-24 | 8.45E-24 | 44.18088297 |
| MFSD10 | 0.726020951 | 4.512124427 | 10.44826458 | 1.61E-24 | 8.53E-24 | 44.17123127 |
| MYC | -1.190529039 | 4.904584889 | -10.44822156 | 1.61E-24 | 8.53E-24 | 44.17081931 |
| SSBP2 | -0.645184066 | 2.011664144 | -10.44641454 | 1.64E-24 | 8.67E-24 | 44.15351499 |
| SCGB3A1 | -1.959416127 | 1.775747237 | -10.42632131 | 1.99E-24 | 1.05E-23 | 43.9612679 |
| FBXW9 | 0.647768825 | 2.902547766 | 10.42559853 | 2.01E-24 | 1.06E-23 | 43.95435818 |
| ZNF205 | 0.689348064 | 2.982879372 | 10.42528131 | 2.01E-24 | 1.06E-23 | 43.9513258 |
| CDH11 | 1.043427394 | 3.132007001 | 10.42212358 | 2.07E-24 | 1.09E-23 | 43.92114394 |
| MCC | -0.779529408 | 2.085842457 | -10.42211245 | 2.07E-24 | 1.09E-23 | 43.92103756 |
| LYPLA1 | 0.782636551 | 4.320823552 | 10.41835667 | 2.15E-24 | 1.13E-23 | 43.88514953 |
| PROM2 | 0.978032209 | 4.272202551 | 10.41580707 | 2.20E-24 | 1.16E-23 | 43.86079316 |
| SGPL1 | 0.596755035 | 4.038714689 | 10.41347543 | 2.25E-24 | 1.19E-23 | 43.83852334 |
| PRR15 | 1.652178118 | 3.567237029 | 10.41078877 | 2.31E-24 | 1.22E-23 | 43.81286784 |
| CERCAM | 0.803898687 | 3.795050996 | 10.40480235 | 2.45E-24 | 1.29E-23 | 43.75572209 |
| ZNF334 | -0.634008961 | 0.735112854 | -10.40266236 | 2.50E-24 | 1.32E-23 | 43.73530062 |
| PHLDB3 | 0.682104306 | 1.994053906 | 10.40133796 | 2.53E-24 | 1.33E-23 | 43.72266389 |
| RPL34 | -0.670126538 | 6.846496445 | -10.40032515 | 2.56E-24 | 1.35E-23 | 43.71300111 |
| ZNF428 | 0.736379543 | 3.787358585 | 10.39798542 | 2.62E-24 | 1.38E-23 | 43.69068169 |
| KMT5C | 0.61145471 | 1.838472365 | 10.39693013 | 2.64E-24 | 1.39E-23 | 43.68061639 |
| CRABP2 | 1.697014855 | 8.102937568 | 10.39610151 | 2.67E-24 | 1.40E-23 | 43.67271365 |
| THOP1 | 0.617168717 | 2.511655285 | 10.39330681 | 2.74E-24 | 1.44E-23 | 43.64606369 |
| DTX3L | 0.686011652 | 4.249760435 | 10.39014487 | 2.82E-24 | 1.48E-23 | 43.61591905 |
| GINS4 | 0.741224464 | 1.266020816 | 10.38931038 | 2.85E-24 | 1.49E-23 | 43.60796464 |
| NYNRIN | -0.759732927 | 2.136245191 | -10.38847827 | 2.87E-24 | 1.50E-23 | 43.60003348 |
| ANPEP | -1.648175497 | 3.547424582 | -10.3883862 | 2.87E-24 | 1.51E-23 | 43.59915591 |
| DUS1L | 0.715123008 | 3.901614083 | 10.38747104 | 2.90E-24 | 1.52E-23 | 43.59043383 |
| PDGFC | -0.765343318 | 2.650631906 | -10.3861583 | 2.93E-24 | 1.54E-23 | 43.57792363 |
| PTOV1 | 0.625091793 | 4.317425106 | 10.38612474 | 2.93E-24 | 1.54E-23 | 43.57760381 |
| BASP1 | 0.986564857 | 3.960297288 | 10.36346843 | 3.65E-24 | 1.91E-23 | 43.36190173 |
| ATF5 | 0.645477479 | 4.104646276 | 10.36226361 | 3.69E-24 | 1.93E-23 | 43.3504421 |
| PCGF2 | 0.806507696 | 3.968194178 | 10.36091404 | 3.74E-24 | 1.95E-23 | 43.33760704 |
| PLA2G16 | -1.208898507 | 4.635764206 | -10.35931894 | 3.80E-24 | 1.98E-23 | 43.32243861 |
| WISP2 | -1.318917509 | 2.28640636 | -10.35558205 | 3.93E-24 | 2.05E-23 | 43.28691081 |
| BMF | 0.639764106 | 2.59154896 | 10.35269112 | 4.05E-24 | 2.11E-23 | 43.25943322 |
| CSF1 | -0.887686571 | 3.427744851 | -10.35264243 | 4.05E-24 | 2.11E-23 | 43.25897049 |
| LYNX1 | -1.020709418 | 1.663880576 | -10.35104893 | 4.11E-24 | 2.14E-23 | 43.24382742 |
| PLS3 | -0.882148206 | 3.798281676 | -10.34963945 | 4.17E-24 | 2.17E-23 | 43.23043475 |
| TTC38 | -0.586119289 | 3.02548489 | -10.34784225 | 4.24E-24 | 2.21E-23 | 43.21336026 |
| DNAAF3 | 0.691911199 | 0.923715048 | 10.34726085 | 4.26E-24 | 2.22E-23 | 43.20783709 |
| ZNF219 | -0.73189772 | 2.073557661 | -10.34701386 | 4.27E-24 | 2.22E-23 | 43.20549079 |
| PDPR | -0.593796163 | 2.436175106 | -10.34514503 | 4.35E-24 | 2.26E-23 | 43.18773954 |
| C4orf48 | 1.115786106 | 2.296132089 | 10.34375448 | 4.41E-24 | 2.29E-23 | 43.17453311 |
| RHBDF2 | 0.639013876 | 2.406880544 | 10.34318385 | 4.43E-24 | 2.30E-23 | 43.16911401 |
| HID1 | 1.16737979 | 4.355078229 | 10.34247404 | 4.46E-24 | 2.32E-23 | 43.16237364 |
| DEGS2 | 1.649503904 | 3.341107397 | 10.33918336 | 4.60E-24 | 2.39E-23 | 43.13113006 |
| SLC52A3 | 0.940431639 | 2.700897125 | 10.33887454 | 4.62E-24 | 2.40E-23 | 43.12819842 |
| PSPH | 0.686833211 | 2.72752304 | 10.33434696 | 4.82E-24 | 2.50E-23 | 43.08522568 |
| FBXL3 | -0.619081105 | 3.36101282 | -10.33337732 | 4.87E-24 | 2.52E-23 | 43.07602462 |
| WDR18 | 0.688318407 | 3.609194843 | 10.33183685 | 4.94E-24 | 2.56E-23 | 43.06140823 |
| GOT2 | 0.653703943 | 4.959904142 | 10.32993966 | 5.03E-24 | 2.61E-23 | 43.04340982 |
| TSPAN3 | -0.706886359 | 4.196537413 | -10.32908423 | 5.07E-24 | 2.62E-23 | 43.03529528 |
| DYNC1LI2 | -0.693044245 | 3.541036802 | -10.32308858 | 5.37E-24 | 2.78E-23 | 42.97843719 |
| FRY | -0.654603413 | 1.625850524 | -10.31944578 | 5.56E-24 | 2.88E-23 | 42.94390518 |
| SBNO2 | 0.625558253 | 3.74927962 | 10.31801489 | 5.64E-24 | 2.91E-23 | 42.93034383 |
| CYHR1 | 0.647415357 | 2.908975217 | 10.31626149 | 5.73E-24 | 2.96E-23 | 42.913728 |
| UNC119B | -0.69553376 | 3.06108266 | -10.3131355 | 5.91E-24 | 3.05E-23 | 42.88411091 |
| PMAIP1 | 1.119559759 | 2.720819662 | 10.31058102 | 6.05E-24 | 3.12E-23 | 42.85991419 |
| CNIH4 | 0.589598578 | 3.442033429 | 10.30938033 | 6.12E-24 | 3.16E-23 | 42.84854263 |
| AFAP1L2 | -0.903952996 | 2.330006536 | -10.30724615 | 6.25E-24 | 3.22E-23 | 42.82833288 |
| RBL2 | -0.685452849 | 3.539217565 | -10.30472542 | 6.40E-24 | 3.30E-23 | 42.80446715 |
| NR2F1 | -1.151832285 | 2.136296792 | -10.30324277 | 6.49E-24 | 3.35E-23 | 42.79043205 |
| ST3GAL4 | 0.719187544 | 2.995492866 | 10.2985107 | 6.80E-24 | 3.50E-23 | 42.7456486 |
| PPP1R9B | 0.609526697 | 4.299708143 | 10.29576204 | 6.98E-24 | 3.59E-23 | 42.71964361 |
| PAIP2B | -0.587948581 | 1.406501805 | -10.28948514 | 7.41E-24 | 3.81E-23 | 42.6602799 |
| TOMM7 | -0.592509575 | 6.041239726 | -10.28478929 | 7.75E-24 | 3.98E-23 | 42.61588883 |
| PTP4A3 | 0.797849136 | 3.445704958 | 10.28363655 | 7.83E-24 | 4.03E-23 | 42.6049943 |
| P4HA1 | 0.74654774 | 4.633052 | 10.27345146 | 8.63E-24 | 4.43E-23 | 42.50877948 |
| EGF | -0.945775378 | 1.187866442 | -10.27252974 | 8.71E-24 | 4.47E-23 | 42.50007624 |
| LRRC73 | 0.688524893 | 1.060520111 | 10.26528441 | 9.33E-24 | 4.78E-23 | 42.43168606 |
| ARHGAP36 | -1.074536505 | 0.469398221 | -10.26445697 | 9.40E-24 | 4.82E-23 | 42.42387828 |
| MIEN1 | 1.153840746 | 4.507298418 | 10.26085982 | 9.73E-24 | 4.98E-23 | 42.38994131 |
| UBALD1 | 0.606519283 | 2.811584788 | 10.25625314 | 1.02E-23 | 5.20E-23 | 42.3464947 |
| ACAT1 | -0.659801065 | 3.433881693 | -10.24740608 | 1.11E-23 | 5.65E-23 | 42.26310198 |
| HS6ST1 | 0.620915456 | 3.910769412 | 10.24466952 | 1.14E-23 | 5.80E-23 | 42.23731927 |
| KLK4 | 0.886746751 | 1.07971812 | 10.24375687 | 1.15E-23 | 5.85E-23 | 42.22872193 |
| MAP7 | 0.754877843 | 3.608308694 | 10.24298224 | 1.15E-23 | 5.89E-23 | 42.2214253 |
| ZFP36L1 | -0.809956479 | 6.22371794 | -10.23868536 | 1.20E-23 | 6.13E-23 | 42.18095927 |
| C7orf50 | 0.704174788 | 3.57397391 | 10.23751076 | 1.22E-23 | 6.19E-23 | 42.16989988 |
| MYH10 | -0.669832554 | 3.464792362 | -10.23482266 | 1.25E-23 | 6.35E-23 | 42.14459419 |
| FAM129B | 0.622118989 | 5.443791529 | 10.23383685 | 1.26E-23 | 6.41E-23 | 42.13531519 |
| COL1A2 | 1.443253912 | 8.163724334 | 10.23006846 | 1.30E-23 | 6.64E-23 | 42.09985209 |
| CYB561D2 | 0.629355251 | 2.903211738 | 10.22805747 | 1.33E-23 | 6.77E-23 | 42.08093178 |
| C1orf56 | 0.614997022 | 2.547709878 | 10.22473552 | 1.37E-23 | 6.98E-23 | 42.04968414 |
| SCPEP1 | -0.9175654 | 4.650793268 | -10.22411855 | 1.38E-23 | 7.02E-23 | 42.04388162 |
| GALNT16 | -0.87660503 | 1.459330692 | -10.21660573 | 1.48E-23 | 7.53E-23 | 41.97324801 |
| DLL1 | -0.690643757 | 1.580939355 | -10.21254319 | 1.54E-23 | 7.83E-23 | 41.93507123 |
| MICALL2 | 0.673785902 | 2.000056138 | 10.21194773 | 1.55E-23 | 7.87E-23 | 41.9294766 |
| FADD | 0.75864564 | 2.940550154 | 10.21179581 | 1.55E-23 | 7.88E-23 | 41.92804926 |
| SOX4 | 0.800690533 | 5.095406774 | 10.20588619 | 1.64E-23 | 8.33E-23 | 41.87254119 |
| FCGR3A | 1.060682191 | 4.390313059 | 10.19987747 | 1.74E-23 | 8.82E-23 | 41.81613 |
| FAM171A1 | -1.103441255 | 2.483089511 | -10.19949584 | 1.74E-23 | 8.85E-23 | 41.81254804 |
| MEX3A | 1.217889646 | 2.611855491 | 10.18981922 | 1.91E-23 | 9.69E-23 | 41.72176336 |
| PAM | -0.872207513 | 3.953675468 | -10.18663933 | 1.97E-23 | 9.98E-23 | 41.69194583 |
| HIST3H2BB | 0.743825602 | 0.87337561 | 10.18506858 | 2.00E-23 | 1.01E-22 | 41.67721999 |
| SYNGR3 | 0.635552016 | 0.726053398 | 10.170749 | 2.29E-23 | 1.16E-22 | 41.54306125 |
| PNISR | -0.596178712 | 3.110355591 | -10.16143043 | 2.50E-23 | 1.26E-22 | 41.45584184 |
| PARP9 | 0.767725152 | 3.603945635 | 10.1602037 | 2.53E-23 | 1.28E-22 | 41.44436492 |
| TRPM2 | 0.699904775 | 1.811439874 | 10.15697555 | 2.61E-23 | 1.32E-22 | 41.41416906 |
| SNAI2 | -0.927272396 | 3.24652703 | -10.1550216 | 2.65E-23 | 1.34E-22 | 41.39589586 |
| SPATA18 | -0.892391127 | 1.628626908 | -10.15400032 | 2.68E-23 | 1.35E-22 | 41.3863461 |
| OASL | 1.180239922 | 2.137715096 | 10.15057849 | 2.77E-23 | 1.40E-22 | 41.35435526 |
| CLEC10A | -0.949256015 | 1.249219945 | -10.14299244 | 2.97E-23 | 1.50E-22 | 41.28346528 |
| PLEKHA4 | -1.039820921 | 3.014603569 | -10.13922715 | 3.08E-23 | 1.55E-22 | 41.24829604 |
| ADRB1 | -0.669862678 | 0.435037703 | -10.13865217 | 3.10E-23 | 1.56E-22 | 41.24292648 |
| WDR54 | 0.669390019 | 2.437345618 | 10.13499472 | 3.21E-23 | 1.61E-22 | 41.20877667 |
| PARP12 | 0.647623347 | 3.025749319 | 10.12389924 | 3.56E-23 | 1.79E-22 | 41.1052411 |
| PI3 | -1.790507573 | 1.36396904 | -10.12271137 | 3.60E-23 | 1.81E-22 | 41.09416242 |
| MMP14 | 1.034659218 | 6.322545543 | 10.12109855 | 3.66E-23 | 1.83E-22 | 41.07912204 |
| TDO2 | 0.640585994 | 0.674531268 | 10.11830318 | 3.75E-23 | 1.88E-22 | 41.05305871 |
| PLAC1 | 0.612072707 | 0.59708606 | 10.11320456 | 3.94E-23 | 1.97E-22 | 41.00553609 |
| RIPK2 | 0.668818473 | 3.332831271 | 10.11192472 | 3.98E-23 | 2.00E-22 | 40.99361018 |
| THY1 | 0.897880103 | 4.472646074 | 10.08529754 | 5.12E-23 | 2.56E-22 | 40.74578117 |
| RAMP2 | -0.956421407 | 4.272496669 | -10.08190069 | 5.28E-23 | 2.64E-22 | 40.71420504 |
| SKA2 | 0.703592309 | 3.65864711 | 10.08136426 | 5.31E-23 | 2.65E-22 | 40.70921943 |
| TMX4 | -0.697551237 | 3.6571628 | -10.07625754 | 5.57E-23 | 2.78E-22 | 40.66176788 |
| NOP16 | 0.590226517 | 2.850423716 | 10.0737991 | 5.70E-23 | 2.85E-22 | 40.63893142 |
| LIMA1 | -0.781107844 | 4.435090854 | -10.07242235 | 5.78E-23 | 2.88E-22 | 40.62614475 |
| KREMEN2 | 0.820114651 | 0.872846018 | 10.07185927 | 5.81E-23 | 2.90E-22 | 40.62091561 |
| PTPRS | -0.786641309 | 3.113440786 | -10.06819828 | 6.01E-23 | 3.00E-22 | 40.58692276 |
| FDXR | 0.770194848 | 2.325990405 | 10.06702862 | 6.08E-23 | 3.03E-22 | 40.5760645 |
| SLC12A7 | 0.594088179 | 4.102013206 | 10.06098766 | 6.43E-23 | 3.20E-22 | 40.52000171 |
| ASPH | -0.975188748 | 3.727714996 | -10.06049532 | 6.46E-23 | 3.21E-22 | 40.51543389 |
| FHDC1 | 0.65777815 | 2.078439227 | 10.05220343 | 6.98E-23 | 3.47E-22 | 40.43853091 |
| CXCR4 | 1.001794919 | 5.009964122 | 10.05104988 | 7.06E-23 | 3.51E-22 | 40.42783654 |
| GPATCH2 | 0.603782302 | 2.22561012 | 10.04849893 | 7.23E-23 | 3.59E-22 | 40.40419084 |
| TTC39A | 1.18226002 | 3.425570319 | 10.04299626 | 7.61E-23 | 3.77E-22 | 40.35320187 |
| RAVER1 | 0.58597649 | 2.509196192 | 10.04253102 | 7.64E-23 | 3.79E-22 | 40.34889196 |
| ECI1 | 0.760287114 | 3.980794214 | 10.03805309 | 7.97E-23 | 3.95E-22 | 40.30741751 |
| SERPINB5 | -1.60876642 | 1.944488413 | -10.03697049 | 8.05E-23 | 3.99E-22 | 40.29739289 |
| NAA10 | 0.614630021 | 2.912541118 | 10.03608516 | 8.12E-23 | 4.02E-22 | 40.28919562 |
| MTERF3 | 0.639884156 | 3.764559673 | 10.03502799 | 8.20E-23 | 4.06E-22 | 40.279408 |
| SCAND1 | 0.894027458 | 4.132935523 | 10.03484172 | 8.21E-23 | 4.07E-22 | 40.27768355 |
| TMEM134 | 0.61457828 | 2.74313008 | 10.03413208 | 8.27E-23 | 4.09E-22 | 40.2711141 |
| SLAMF8 | 1.004094335 | 2.297042654 | 10.02938955 | 8.64E-23 | 4.27E-22 | 40.22722047 |
| JUP | 0.698727497 | 6.430624753 | 10.02845903 | 8.72E-23 | 4.31E-22 | 40.2186103 |
| NDUFS8 | 0.714993987 | 3.912301426 | 10.02843923 | 8.72E-23 | 4.31E-22 | 40.2184271 |
| PTRH2 | 0.639879622 | 2.639715044 | 10.0276765 | 8.78E-23 | 4.34E-22 | 40.21137 |
| ME1 | -1.168497202 | 2.480997045 | -10.01146184 | 1.02E-22 | 5.04E-22 | 40.06145248 |
| PLEKHG1 | -0.730589455 | 1.907000473 | -10.01118448 | 1.02E-22 | 5.05E-22 | 40.0588898 |
| PLEKHG5 | -0.605144243 | 1.725015132 | -10.00551444 | 1.08E-22 | 5.32E-22 | 40.00651546 |
| CLDN3 | 1.451232562 | 5.325088552 | 10.00038371 | 1.13E-22 | 5.58E-22 | 39.95914429 |
| POLD4 | 0.700683383 | 2.66757587 | 9.999470983 | 1.14E-22 | 5.63E-22 | 39.95071941 |
| BCHE | -0.837958964 | 0.550319542 | -9.998482623 | 1.15E-22 | 5.68E-22 | 39.94159712 |
| ETS1 | -0.840934847 | 3.873987009 | -9.993921623 | 1.20E-22 | 5.92E-22 | 39.89951024 |
| TPM2 | -1.118339464 | 4.624139635 | -9.987335188 | 1.28E-22 | 6.29E-22 | 39.83876224 |
| PWWP2B | 0.717203787 | 3.221238533 | 9.987229811 | 1.28E-22 | 6.29E-22 | 39.83779061 |
| COMMD3 | 0.613149639 | 3.353101256 | 9.982321319 | 1.34E-22 | 6.58E-22 | 39.79254107 |
| CELSR1 | 1.220871422 | 3.795689759 | 9.96705387 | 1.54E-22 | 7.57E-22 | 39.65191665 |
| VSIG2 | -1.300960125 | 1.476019144 | -9.964989943 | 1.57E-22 | 7.71E-22 | 39.63292036 |
| ZNF787 | 0.660066036 | 2.838218346 | 9.9559658 | 1.71E-22 | 8.37E-22 | 39.54990171 |
| BSG | 0.621451436 | 6.946768983 | 9.954423449 | 1.74E-22 | 8.49E-22 | 39.53571905 |
| P2RY13 | -0.680444135 | 1.307260229 | -9.951788931 | 1.78E-22 | 8.69E-22 | 39.5114977 |
| ARC | -0.797382857 | 0.723186388 | -9.940457411 | 1.98E-22 | 9.64E-22 | 39.40737942 |
| GRN | 0.618977117 | 6.649584585 | 9.937883679 | 2.03E-22 | 9.87E-22 | 39.38374502 |
| YWHAZ | 0.690321555 | 6.833560981 | 9.934757827 | 2.09E-22 | 1.02E-21 | 39.35504752 |
| GCAT | 0.781750389 | 2.931286318 | 9.934248618 | 2.10E-22 | 1.02E-21 | 39.35037335 |
| ALDH3A1 | -0.686210955 | 0.308843865 | -9.933164788 | 2.12E-22 | 1.03E-21 | 39.34042526 |
| MCCC1 | -0.63537646 | 3.044125727 | -9.931858725 | 2.14E-22 | 1.04E-21 | 39.32843859 |
| MSX1 | -0.616950339 | 1.412519738 | -9.930227951 | 2.17E-22 | 1.06E-21 | 39.3134737 |
| NOVA1 | -1.163594543 | 1.254809079 | -9.929779388 | 2.18E-22 | 1.06E-21 | 39.3093578 |
| DDRGK1 | 0.704478613 | 4.992947321 | 9.922874384 | 2.33E-22 | 1.13E-21 | 39.24601914 |
| SLC6A9 | 0.915145906 | 2.342177914 | 9.919210651 | 2.41E-22 | 1.17E-21 | 39.21242739 |
| TMEM106C | 0.659578109 | 4.634298925 | 9.913428863 | 2.54E-22 | 1.23E-21 | 39.15943723 |
| GBP2 | -0.898011057 | 4.020162988 | -9.912184291 | 2.57E-22 | 1.24E-21 | 39.14803413 |
| SRD5A3 | 0.692043961 | 3.40223677 | 9.908410573 | 2.66E-22 | 1.29E-21 | 39.11346578 |
| BCL6B | -0.59790192 | 2.165181131 | -9.905364241 | 2.74E-22 | 1.32E-21 | 39.08556864 |
| SPNS2 | -0.867574645 | 2.041670037 | -9.904242602 | 2.77E-22 | 1.34E-21 | 39.07529894 |
| CYTH3 | -0.665960979 | 2.971348844 | -9.894187644 | 3.04E-22 | 1.47E-21 | 38.98328006 |
| TPPP3 | -1.113113786 | 2.680278372 | -9.893845397 | 3.05E-22 | 1.47E-21 | 38.98014935 |
| CCNE1 | 1.009853124 | 1.518862595 | 9.891346963 | 3.12E-22 | 1.50E-21 | 38.95729769 |
| SLC34A2 | -1.778881811 | 1.973120643 | -9.887889485 | 3.22E-22 | 1.55E-21 | 38.92568231 |
| NDUFA13 | 0.681909573 | 3.374066067 | 9.881980006 | 3.40E-22 | 1.64E-21 | 38.87166743 |
| INCENP | 0.638678085 | 2.572495541 | 9.881931288 | 3.40E-22 | 1.64E-21 | 38.87122225 |
| RABAC1 | 0.74666559 | 5.198181513 | 9.873544414 | 3.67E-22 | 1.77E-21 | 38.79461048 |
| NDST1 | -0.612895392 | 4.295375232 | -9.872584884 | 3.71E-22 | 1.78E-21 | 38.78584896 |
| MISP3 | 0.946991293 | 2.479819897 | 9.872203126 | 3.72E-22 | 1.79E-21 | 38.78236331 |
| ETV7 | 0.880646588 | 1.611266955 | 9.872115303 | 3.72E-22 | 1.79E-21 | 38.78156146 |
| TGFB1 | 0.715822537 | 3.978290648 | 9.871107921 | 3.76E-22 | 1.81E-21 | 38.77236415 |
| DEF6 | 0.714778422 | 3.144754869 | 9.868570692 | 3.85E-22 | 1.85E-21 | 38.74920302 |
| FOXRED2 | 0.661098528 | 3.155374375 | 9.868360885 | 3.85E-22 | 1.85E-21 | 38.74728801 |
| UQCRQ | 0.738085786 | 6.002410623 | 9.867287627 | 3.89E-22 | 1.87E-21 | 38.73749246 |
| PARD6B | 1.27773794 | 2.849878478 | 9.866170268 | 3.93E-22 | 1.89E-21 | 38.72729537 |
| SEMA3C | -1.224223883 | 4.443453636 | -9.864765501 | 3.98E-22 | 1.91E-21 | 38.71447677 |
| INAFM2 | -0.59476172 | 3.682033668 | -9.858555767 | 4.22E-22 | 2.02E-21 | 38.65783105 |
| TUBB3 | 0.636979328 | 0.675798239 | 9.857145832 | 4.27E-22 | 2.05E-21 | 38.64497373 |
| RRAD | -0.802490406 | 0.963647644 | -9.853722235 | 4.41E-22 | 2.11E-21 | 38.61376016 |
| PKN1 | 0.658832577 | 4.78750861 | 9.852301061 | 4.47E-22 | 2.14E-21 | 38.60080576 |
| HIST1H2BO | 0.850517112 | 0.869108473 | 9.85106052 | 4.52E-22 | 2.16E-21 | 38.58949919 |
| S100A4 | -1.034690893 | 6.003994073 | -9.85021332 | 4.55E-22 | 2.18E-21 | 38.5817783 |
| ZG16B | 1.537857391 | 4.294242133 | 9.84993139 | 4.57E-22 | 2.18E-21 | 38.57920908 |
| STARD13 | -0.632712033 | 2.116376467 | -9.849461582 | 4.59E-22 | 2.19E-21 | 38.57492788 |
| PRDX2 | 0.600662027 | 6.554371522 | 9.848014257 | 4.65E-22 | 2.22E-21 | 38.56173997 |
| CFB | 1.442632292 | 2.907049408 | 9.829864096 | 5.49E-22 | 2.62E-21 | 38.39649722 |
| ARHGAP5 | -0.657286212 | 3.096505812 | -9.829355298 | 5.52E-22 | 2.63E-21 | 38.39186875 |
| SEC16A | 0.621639372 | 4.603022573 | 9.828267582 | 5.57E-22 | 2.65E-21 | 38.38197464 |
| RPL39L | 0.979783863 | 3.61634416 | 9.827700289 | 5.60E-22 | 2.67E-21 | 38.37681478 |
| PTPN11 | -0.587183267 | 4.396264247 | -9.82620682 | 5.68E-22 | 2.70E-21 | 38.36323204 |
| TMEM63C | 1.020269679 | 2.142661375 | 9.823993884 | 5.80E-22 | 2.76E-21 | 38.34310913 |
| VEGFB | -0.635609879 | 5.553431638 | -9.807392371 | 6.75E-22 | 3.21E-21 | 38.1922696 |
| RCSD1 | -0.736790533 | 1.929717014 | -9.806598023 | 6.80E-22 | 3.23E-21 | 38.18505769 |
| MRPL15 | 0.663046813 | 5.239794359 | 9.797092893 | 7.42E-22 | 3.52E-21 | 38.0987989 |
| SLAIN1 | -0.677399246 | 0.784862152 | -9.796964984 | 7.43E-22 | 3.52E-21 | 38.09763862 |
| OAS3 | 1.080399404 | 3.736595228 | 9.796827709 | 7.44E-22 | 3.52E-21 | 38.09639339 |
| NDUFB9 | 0.728304799 | 5.667173721 | 9.789020587 | 7.99E-22 | 3.78E-21 | 38.02559892 |
| PRKD3 | -0.697523531 | 2.247315922 | -9.779113669 | 8.75E-22 | 4.13E-21 | 37.9358329 |
| EPYC | 1.062139157 | 1.012044511 | 9.777716712 | 8.86E-22 | 4.19E-21 | 37.92318139 |
| STEAP1 | -0.990429908 | 2.749728927 | -9.777651407 | 8.86E-22 | 4.19E-21 | 37.92258999 |
| FBXL16 | 1.231431221 | 2.689595744 | 9.776390979 | 8.97E-22 | 4.24E-21 | 37.91117634 |
| TMEM213 | -0.707838376 | 0.40820017 | -9.768050345 | 9.68E-22 | 4.57E-21 | 37.83568035 |
| MDK | 1.170650676 | 5.819333769 | 9.766550611 | 9.81E-22 | 4.63E-21 | 37.8221112 |
| MSRB1 | 0.631094062 | 3.892218144 | 9.765783197 | 9.88E-22 | 4.66E-21 | 37.81516856 |
| MTUS1 | -0.775298192 | 3.089667055 | -9.763884048 | 1.01E-21 | 4.74E-21 | 37.79798932 |
| TCIRG1 | 0.789754414 | 3.796720929 | 9.758191368 | 1.06E-21 | 4.99E-21 | 37.74651182 |
| JAG2 | -0.636193938 | 2.638076935 | -9.757696226 | 1.06E-21 | 5.01E-21 | 37.74203559 |
| CISD3 | 0.821412697 | 3.743337384 | 9.757377948 | 1.07E-21 | 5.02E-21 | 37.73915835 |
| NFASC | -0.791216487 | 1.092737517 | -9.752079773 | 1.12E-21 | 5.27E-21 | 37.69127469 |
| RAB11FIP3 | 0.669675219 | 3.41530218 | 9.750747286 | 1.13E-21 | 5.33E-21 | 37.67923547 |
| NEGR1 | -0.635531042 | 0.963944993 | -9.749513672 | 1.15E-21 | 5.39E-21 | 37.66809083 |
| GSDMD | 0.820870105 | 3.792255302 | 9.739631692 | 1.25E-21 | 5.89E-21 | 37.57885916 |
| SPAG4 | 0.703330642 | 1.523026725 | 9.738034612 | 1.27E-21 | 5.98E-21 | 37.56444521 |
| MROH6 | 0.859658941 | 1.604715858 | 9.734777204 | 1.31E-21 | 6.15E-21 | 37.53505271 |
| FAM117A | -0.590446845 | 2.509056899 | -9.725914078 | 1.42E-21 | 6.67E-21 | 37.45512079 |
| HSPB1 | 1.219196451 | 7.779593019 | 9.722926283 | 1.46E-21 | 6.85E-21 | 37.42818944 |
| CXCL9 | 1.909632527 | 3.78953466 | 9.721994124 | 1.47E-21 | 6.90E-21 | 37.41978859 |
| GPCPD1 | -0.638254324 | 2.689244553 | -9.715704364 | 1.56E-21 | 7.30E-21 | 37.36312176 |
| PODXL | -0.774804421 | 4.189494234 | -9.714867175 | 1.57E-21 | 7.35E-21 | 37.35558158 |
| BLNK | 0.685451834 | 2.480517262 | 9.712583583 | 1.61E-21 | 7.50E-21 | 37.33501709 |
| CLSTN3 | 0.686158814 | 3.673352208 | 9.711817501 | 1.62E-21 | 7.55E-21 | 37.32811921 |
| LIMD2 | 0.882589938 | 2.04334668 | 9.70625136 | 1.70E-21 | 7.93E-21 | 37.27801501 |
| C15orf48 | 1.390151962 | 3.589473205 | 9.705122178 | 1.72E-21 | 8.01E-21 | 37.26785356 |
| HIST1H2BE | 0.593045945 | 0.645212811 | 9.704171389 | 1.73E-21 | 8.08E-21 | 37.25929824 |
| CBX2 | 1.146555618 | 1.829137527 | 9.701919774 | 1.77E-21 | 8.25E-21 | 37.23904076 |
| IER3 | 1.153553703 | 5.842116297 | 9.698594192 | 1.82E-21 | 8.49E-21 | 37.20912832 |
| BATF | 1.070680348 | 3.459960315 | 9.69574508 | 1.87E-21 | 8.71E-21 | 37.18350854 |
| SLC22A18 | 0.765493219 | 2.292954515 | 9.684082344 | 2.08E-21 | 9.67E-21 | 37.07870195 |
| LINGO1 | 1.14307546 | 1.923898058 | 9.683426769 | 2.09E-21 | 9.73E-21 | 37.07281386 |
| ITGB1 | -0.790370348 | 5.920912036 | -9.681552475 | 2.13E-21 | 9.89E-21 | 37.05598165 |
| SH3BP1 | 0.586716303 | 1.951059872 | 9.678355387 | 2.19E-21 | 1.02E-20 | 37.02727643 |
| ECHDC2 | -0.663496452 | 2.482314087 | -9.67709907 | 2.22E-21 | 1.03E-20 | 37.01599875 |
| RRP1 | 0.657779292 | 3.076549107 | 9.672758154 | 2.30E-21 | 1.07E-20 | 36.97704092 |
| SH2D3C | -0.5970932 | 2.142010457 | -9.667042327 | 2.43E-21 | 1.13E-20 | 36.92576669 |
| WWTR1 | -0.742255532 | 3.324223917 | -9.660623321 | 2.57E-21 | 1.19E-20 | 36.86821545 |
| MEF2A | -0.594001858 | 3.186343369 | -9.657172394 | 2.65E-21 | 1.23E-20 | 36.8372888 |
| EME2 | 0.704048268 | 1.642403978 | 9.655900386 | 2.69E-21 | 1.24E-20 | 36.82589166 |
| HAS1 | -0.602149062 | 0.43842261 | -9.654897041 | 2.71E-21 | 1.25E-20 | 36.81690263 |
| HSPH1 | 0.686581868 | 4.204532493 | 9.644599033 | 2.97E-21 | 1.37E-20 | 36.72468841 |
| HEG1 | -0.766332759 | 3.202122478 | -9.641369618 | 3.06E-21 | 1.41E-20 | 36.69578776 |
| COL3A1 | 1.410181485 | 8.717709366 | 9.638222158 | 3.15E-21 | 1.45E-20 | 36.6676285 |
| CTPS2 | 0.611249841 | 3.281915523 | 9.63557111 | 3.23E-21 | 1.49E-20 | 36.64391658 |
| PIGT | 0.647585349 | 6.502400756 | 9.634345827 | 3.26E-21 | 1.50E-20 | 36.6329591 |
| SEC23B | 0.591248638 | 4.721745046 | 9.630230403 | 3.39E-21 | 1.56E-20 | 36.59616437 |
| PLEKHF2 | 0.885550031 | 4.64719676 | 9.629526035 | 3.41E-21 | 1.57E-20 | 36.58986818 |
| MT-ND1 | -0.902833568 | 11.493194 | -9.621865794 | 3.65E-21 | 1.68E-20 | 36.52142048 |
| POLB | 0.690308866 | 3.178295134 | 9.613956682 | 3.92E-21 | 1.80E-20 | 36.450798 |
| BAALC | -0.721763754 | 0.790229691 | -9.608117441 | 4.14E-21 | 1.90E-20 | 36.39868986 |
| DAPK3 | 0.618568952 | 3.871371927 | 9.604864182 | 4.26E-21 | 1.96E-20 | 36.36967025 |
| CELSR3 | 0.657682486 | 0.888395973 | 9.601491221 | 4.39E-21 | 2.02E-20 | 36.33959177 |
| CRISPLD1 | -1.447858041 | 2.337844962 | -9.599927196 | 4.45E-21 | 2.04E-20 | 36.32564761 |
| CCDC9B | -0.761011399 | 1.588746784 | -9.599424002 | 4.47E-21 | 2.05E-20 | 36.32116177 |
| RSPH1 | 0.837057267 | 1.601987645 | 9.590135318 | 4.86E-21 | 2.23E-20 | 36.23839178 |
| AMMECR1 | 0.586075195 | 2.026445471 | 9.587441859 | 4.98E-21 | 2.28E-20 | 36.21440365 |
| WNT7B | 1.005013584 | 2.035692099 | 9.58661697 | 5.02E-21 | 2.30E-20 | 36.20705829 |
| CKS1B | 0.701631714 | 3.683661333 | 9.585714517 | 5.06E-21 | 2.31E-20 | 36.19902287 |
| IL3RA | -0.63584984 | 2.716972024 | -9.583546901 | 5.16E-21 | 2.36E-20 | 36.17972511 |
| DNMT3B | 0.637944142 | 1.40562982 | 9.578249751 | 5.41E-21 | 2.47E-20 | 36.13258164 |
| TRIM14 | 0.653048146 | 3.533749842 | 9.571198612 | 5.77E-21 | 2.63E-20 | 36.06986276 |
| FAAH2 | 0.77576031 | 2.954842742 | 9.569693814 | 5.84E-21 | 2.67E-20 | 36.05648292 |
| LY75 | -0.599488105 | 1.160825334 | -9.563967096 | 6.15E-21 | 2.80E-20 | 36.00558063 |
| SERPINH1 | 0.769436215 | 5.554140478 | 9.559700422 | 6.39E-21 | 2.91E-20 | 35.96767303 |
| DCDC2 | -0.948607425 | 1.145734541 | -9.557282133 | 6.53E-21 | 2.97E-20 | 35.946194 |
| PLPP2 | 0.82564241 | 3.539467431 | 9.556080496 | 6.60E-21 | 3.01E-20 | 35.93552291 |
| FAM89B | 0.592791669 | 4.033048357 | 9.55394437 | 6.73E-21 | 3.06E-20 | 35.91655596 |
| GRINA | 0.658810904 | 6.916002586 | 9.553777292 | 6.74E-21 | 3.07E-20 | 35.9150726 |
| KCNK15 | 1.290316051 | 2.210858066 | 9.551202558 | 6.90E-21 | 3.14E-20 | 35.89221637 |
| TMC6 | 0.66549068 | 2.619970846 | 9.546099292 | 7.22E-21 | 3.28E-20 | 35.84692969 |
| TMEM200B | -0.786989602 | 1.618844703 | -9.54557016 | 7.26E-21 | 3.30E-20 | 35.84223534 |
| AFF1 | -0.636014873 | 3.242724989 | -9.543435816 | 7.40E-21 | 3.36E-20 | 35.82330211 |
| HAUS1 | 0.592481245 | 3.389543887 | 9.542108468 | 7.49E-21 | 3.40E-20 | 35.81152937 |
| NOD2 | 0.623303472 | 1.294320495 | 9.539489667 | 7.66E-21 | 3.48E-20 | 35.7883064 |
| TNFRSF12A | 1.029714283 | 4.375893396 | 9.53934198 | 7.67E-21 | 3.48E-20 | 35.78699691 |
| KLHDC9 | 0.966876674 | 2.52276716 | 9.537474089 | 7.80E-21 | 3.54E-20 | 35.77043641 |
| CLDN4 | 0.993510644 | 5.585272176 | 9.535310169 | 7.96E-21 | 3.61E-20 | 35.75125484 |
| CITED2 | -0.845437681 | 5.048113783 | -9.535235656 | 7.96E-21 | 3.61E-20 | 35.7505944 |
| ALDH6A1 | -0.679027202 | 3.004572804 | -9.534491811 | 8.02E-21 | 3.63E-20 | 35.74400167 |
| RNF208 | 0.809824405 | 2.818876793 | 9.5329872 | 8.12E-21 | 3.68E-20 | 35.73066759 |
| EDN2 | 1.115035281 | 1.800787106 | 9.52767978 | 8.52E-21 | 3.86E-20 | 35.68364687 |
| PRPS2 | 0.633335565 | 4.302638485 | 9.525723532 | 8.67E-21 | 3.92E-20 | 35.6663213 |
| SC5D | -0.693576074 | 2.936172759 | -9.524385363 | 8.77E-21 | 3.97E-20 | 35.65447153 |
| CPNE7 | 1.021351884 | 1.531695103 | 9.521535563 | 9.00E-21 | 4.07E-20 | 35.62924073 |
| PLCB1 | -0.654041703 | 1.074715069 | -9.511812567 | 9.82E-21 | 4.43E-20 | 35.54320667 |
| RUNX2 | 0.669184588 | 1.664892112 | 9.511760426 | 9.82E-21 | 4.43E-20 | 35.5427455 |
| RNF40 | 0.593241109 | 3.737322803 | 9.506239489 | 1.03E-20 | 4.65E-20 | 35.4939273 |
| CPSF1 | 0.642611281 | 4.027136436 | 9.503630365 | 1.06E-20 | 4.76E-20 | 35.47086492 |
| ARNT2 | 1.170941807 | 2.699990248 | 9.501758194 | 1.07E-20 | 4.83E-20 | 35.45431992 |
| MT-CYB | -0.837207582 | 11.58210691 | -9.491666424 | 1.18E-20 | 5.28E-20 | 35.3651839 |
| JCAD | -0.656421886 | 2.493893155 | -9.487830751 | 1.22E-20 | 5.46E-20 | 35.33132651 |
| RAP2C | 0.686740466 | 3.769929735 | 9.486517293 | 1.23E-20 | 5.52E-20 | 35.31973536 |
| TMEM121 | 0.800319539 | 1.629195524 | 9.484594401 | 1.25E-20 | 5.62E-20 | 35.3027685 |
| OGFR | 0.648397355 | 3.463766485 | 9.481022631 | 1.29E-20 | 5.80E-20 | 35.27126042 |
| PDZD4 | -0.678056021 | 0.883204974 | -9.478803747 | 1.32E-20 | 5.91E-20 | 35.25169186 |
| NFKBIB | 0.619900294 | 3.116566416 | 9.476360709 | 1.35E-20 | 6.04E-20 | 35.23015103 |
| MAPK15 | 1.197995947 | 1.961700467 | 9.471502137 | 1.41E-20 | 6.30E-20 | 35.18732607 |
| DENND4C | -0.670357168 | 2.920562243 | -9.470374283 | 1.42E-20 | 6.36E-20 | 35.17738753 |
| PODN | -1.125413528 | 3.351524797 | -9.469865866 | 1.43E-20 | 6.39E-20 | 35.17290774 |
| CPQ | -0.670541939 | 3.74071759 | -9.466936871 | 1.47E-20 | 6.55E-20 | 35.14710365 |
| DSG1 | -0.879967573 | 0.448369185 | -9.464198367 | 1.50E-20 | 6.71E-20 | 35.12298399 |
| CYP7B1 | -0.762542469 | 1.665328407 | -9.463674402 | 1.51E-20 | 6.74E-20 | 35.11836979 |
| OPTN | -0.667121521 | 4.183344736 | -9.461146146 | 1.54E-20 | 6.89E-20 | 35.0961083 |
| SLC2A6 | 0.667941769 | 1.319098036 | 9.460982966 | 1.55E-20 | 6.90E-20 | 35.09467167 |
| CNTNAP2 | 1.2280919 | 1.377166903 | 9.459986583 | 1.56E-20 | 6.96E-20 | 35.08589998 |
| KLHL35 | 0.639853531 | 1.102567698 | 9.45755631 | 1.59E-20 | 7.10E-20 | 35.06450832 |
| VPS13C | -0.637514108 | 2.30099719 | -9.455011783 | 1.63E-20 | 7.26E-20 | 35.04211606 |
| HOXC10 | 1.590550073 | 3.312945236 | 9.454345037 | 1.64E-20 | 7.30E-20 | 35.03624945 |
| GALNT12 | -0.612454428 | 0.895742861 | -9.451797301 | 1.68E-20 | 7.47E-20 | 35.01383551 |
| PPP2R2C | 1.074274038 | 1.257599402 | 9.449579283 | 1.71E-20 | 7.61E-20 | 34.99432654 |
| MRPL23 | 0.633257838 | 2.972734391 | 9.447783804 | 1.74E-20 | 7.73E-20 | 34.97853697 |
| TSPAN6 | -0.809917109 | 3.435956486 | -9.447012827 | 1.75E-20 | 7.78E-20 | 34.97175774 |
| CH25H | -0.716985553 | 1.234211017 | -9.44616674 | 1.76E-20 | 7.83E-20 | 34.96431861 |
| PLA2G2A | -1.63239136 | 1.85830234 | -9.441894041 | 1.83E-20 | 8.13E-20 | 34.92676013 |
| RGS4 | 0.809027678 | 1.536582418 | 9.437173419 | 1.91E-20 | 8.48E-20 | 34.88528129 |
| ZBTB42 | 0.758310199 | 3.114162665 | 9.436477618 | 1.92E-20 | 8.53E-20 | 34.87916899 |
| SESN3 | -1.056762277 | 2.748226395 | -9.432926445 | 1.98E-20 | 8.80E-20 | 34.84797955 |
| RAI2 | -1.218015685 | 3.03052219 | -9.426684836 | 2.10E-20 | 9.29E-20 | 34.79318493 |
| FES | -0.608732606 | 2.005532092 | -9.426294202 | 2.10E-20 | 9.32E-20 | 34.78975663 |
| RGS5 | -1.018734864 | 3.297133355 | -9.422511235 | 2.18E-20 | 9.63E-20 | 34.75656265 |
| TRAPPC6A | 0.758048114 | 4.705117176 | 9.419494927 | 2.23E-20 | 9.88E-20 | 34.73010403 |
| SEC24D | 0.638778201 | 3.318805111 | 9.412990201 | 2.37E-20 | 1.05E-19 | 34.67307036 |
| GPR143 | 0.735244768 | 1.43114764 | 9.412638591 | 2.37E-20 | 1.05E-19 | 34.6699884 |
| MAGED2 | 1.035330269 | 7.625680578 | 9.40996083 | 2.43E-20 | 1.07E-19 | 34.64652034 |
| EEF1A2 | 2.169217974 | 3.974647018 | 9.40818419 | 2.47E-20 | 1.09E-19 | 34.63095292 |
| SMPDL3B | 0.81464962 | 2.693717466 | 9.406581362 | 2.51E-20 | 1.11E-19 | 34.61691068 |
| ARSI | 0.608406998 | 1.153345835 | 9.394884616 | 2.78E-20 | 1.22E-19 | 34.51449897 |
| TP53I11 | 0.674047392 | 4.598251746 | 9.392063629 | 2.85E-20 | 1.25E-19 | 34.48981607 |
| RGS14 | 0.656825756 | 2.426157141 | 9.389447258 | 2.92E-20 | 1.28E-19 | 34.46692923 |
| TFPI2 | -1.464914232 | 1.630370089 | -9.385888761 | 3.01E-20 | 1.32E-19 | 34.43580993 |
| ADAMDEC1 | 1.156437663 | 1.556300647 | 9.382523743 | 3.10E-20 | 1.36E-19 | 34.40639198 |
| DHCR7 | 0.899553355 | 4.187618682 | 9.37574949 | 3.29E-20 | 1.44E-19 | 34.34719716 |
| TAP1 | 1.026744854 | 4.491601067 | 9.371107471 | 3.43E-20 | 1.50E-19 | 34.30665556 |
| FGF10 | -1.385459977 | 1.327284342 | -9.370502219 | 3.45E-20 | 1.51E-19 | 34.3013708 |
| TSR3 | 0.644208072 | 4.956368397 | 9.368615913 | 3.50E-20 | 1.54E-19 | 34.28490241 |
| ARL15 | -0.64330456 | 2.876819341 | -9.366463443 | 3.57E-20 | 1.56E-19 | 34.26611377 |
| MARC2 | -0.727975153 | 2.964212355 | -9.361072286 | 3.75E-20 | 1.64E-19 | 34.21907141 |
| NUAK1 | -0.654408262 | 2.319636872 | -9.356545277 | 3.90E-20 | 1.70E-19 | 34.17958755 |
| SPEF1 | 0.733750259 | 1.026898522 | 9.355730309 | 3.93E-20 | 1.72E-19 | 34.17248128 |
| ARHGEF19 | 0.725311878 | 2.997816418 | 9.348978961 | 4.17E-20 | 1.82E-19 | 34.11363217 |
| KDELC2 | -0.597095296 | 3.197316656 | -9.346254295 | 4.27E-20 | 1.86E-19 | 34.08989263 |
| TSPAN18 | -0.620253157 | 2.178691445 | -9.344011465 | 4.35E-20 | 1.90E-19 | 34.07035572 |
| LRP5 | -0.731853326 | 3.854382814 | -9.34258264 | 4.41E-20 | 1.92E-19 | 34.05791158 |
| CLEC11A | 0.969823706 | 3.198297251 | 9.341641793 | 4.45E-20 | 1.94E-19 | 34.04971832 |
| HELZ2 | 0.731773 | 2.488233397 | 9.340562702 | 4.49E-20 | 1.95E-19 | 34.04032205 |
| TNFRSF21 | -0.935430192 | 3.670559073 | -9.339688293 | 4.52E-20 | 1.97E-19 | 34.03270875 |
| PPFIA3 | 0.652497204 | 1.569777297 | 9.338236909 | 4.58E-20 | 1.99E-19 | 34.02007321 |
| CAPN13 | 1.158502393 | 2.523940317 | 9.336812294 | 4.64E-20 | 2.02E-19 | 34.00767238 |
| TTYH1 | -1.054530417 | 0.803647879 | -9.335858483 | 4.68E-20 | 2.03E-19 | 33.99937066 |
| RND1 | 1.023275628 | 2.409789324 | 9.33415143 | 4.75E-20 | 2.06E-19 | 33.98451475 |
| PSME1 | 0.610379236 | 6.69574151 | 9.330329134 | 4.91E-20 | 2.13E-19 | 33.95125912 |
| ENAH | 0.6719595 | 4.155619889 | 9.326794203 | 5.07E-20 | 2.20E-19 | 33.92051416 |
| SULT1C3 | -0.916060785 | 0.215919663 | -9.318917121 | 5.43E-20 | 2.35E-19 | 33.85203974 |
| NTN4 | -1.508925668 | 4.339527866 | -9.318709397 | 5.44E-20 | 2.36E-19 | 33.8502347 |
| MT-ND5 | -0.933173579 | 10.10090475 | -9.313598065 | 5.69E-20 | 2.46E-19 | 33.80583021 |
| PSMD3 | 0.848604678 | 5.060601011 | 9.30972401 | 5.89E-20 | 2.54E-19 | 33.77218856 |
| SLC5A1 | -1.186624915 | 1.377509727 | -9.308483517 | 5.95E-20 | 2.57E-19 | 33.76141888 |
| HEYL | 0.664136928 | 2.343202104 | 9.306037207 | 6.08E-20 | 2.62E-19 | 33.74018422 |
| CEP131 | 0.688005392 | 2.505646721 | 9.304109342 | 6.18E-20 | 2.67E-19 | 33.72345321 |
| PAPLN | -0.614686218 | 1.47193773 | -9.299407702 | 6.44E-20 | 2.78E-19 | 33.68266253 |
| IRAK1 | 0.664654032 | 4.947662133 | 9.294296167 | 6.74E-20 | 2.90E-19 | 33.63833592 |
| MARCO | -1.215336544 | 1.238142052 | -9.29011708 | 6.99E-20 | 3.01E-19 | 33.60211107 |
| FMO1 | -0.763659573 | 1.484783701 | -9.284806844 | 7.32E-20 | 3.15E-19 | 33.55610162 |
| FGD3 | 1.194293142 | 2.833097684 | 9.274293895 | 8.03E-20 | 3.45E-19 | 33.46508155 |
| HIST2H2AC | 0.818384888 | 1.301502729 | 9.272941369 | 8.13E-20 | 3.49E-19 | 33.453378 |
| PLK3 | -0.585765376 | 2.523146515 | -9.272407644 | 8.17E-20 | 3.50E-19 | 33.44876004 |
| GNPNAT1 | 0.600594461 | 3.576457529 | 9.264198693 | 8.77E-20 | 3.76E-19 | 33.3777624 |
| ARFIP2 | 0.617415026 | 4.179203217 | 9.263568547 | 8.82E-20 | 3.78E-19 | 33.37231463 |
| AXL | -0.680348488 | 3.536058067 | -9.262294586 | 8.92E-20 | 3.82E-19 | 33.36130193 |
| MSR1 | 0.776524781 | 2.527967921 | 9.262234703 | 8.93E-20 | 3.82E-19 | 33.3607843 |
| PHPT1 | 0.692718443 | 5.011907278 | 9.260838161 | 9.03E-20 | 3.86E-19 | 33.34871352 |
| ESPN | 1.074214145 | 2.337768347 | 9.260657032 | 9.05E-20 | 3.87E-19 | 33.34714808 |
| AKAP11 | -0.60581061 | 2.90840245 | -9.259649352 | 9.13E-20 | 3.90E-19 | 33.33843949 |
| TLE2 | -0.790363089 | 3.097380504 | -9.258319722 | 9.24E-20 | 3.95E-19 | 33.32694979 |
| RAD21 | 0.766135715 | 5.910489101 | 9.254614441 | 9.54E-20 | 4.07E-19 | 33.29493901 |
| MPZ | -0.756241377 | 0.66396409 | -9.251744792 | 9.78E-20 | 4.17E-19 | 33.27015508 |
| TMPRSS2 | -0.937499313 | 1.774210489 | -9.244584638 | 1.04E-19 | 4.44E-19 | 33.20834493 |
| FBLN1 | -1.02776444 | 4.810091004 | -9.244107496 | 1.05E-19 | 4.45E-19 | 33.20422747 |
| ARHGAP29 | -0.753747797 | 2.715727923 | -9.239852965 | 1.09E-19 | 4.62E-19 | 33.16752146 |
| SLC38A5 | 0.727524453 | 1.416292471 | 9.232315682 | 1.16E-19 | 4.93E-19 | 33.10252947 |
| LRP6 | -0.641604959 | 2.608057719 | -9.232079289 | 1.16E-19 | 4.94E-19 | 33.10049186 |
| GPAA1 | 0.628372826 | 5.368248746 | 9.224493728 | 1.24E-19 | 5.27E-19 | 33.03513148 |
| PLCD3 | -0.726908189 | 2.363392461 | -9.214372434 | 1.36E-19 | 5.75E-19 | 32.94799479 |
| DOHH | 0.632693088 | 2.386671207 | 9.212134281 | 1.38E-19 | 5.86E-19 | 32.92873718 |
| GLT8D2 | -0.823219552 | 2.649013126 | -9.205071738 | 1.47E-19 | 6.21E-19 | 32.86799602 |
| SLC44A4 | 1.818072481 | 4.316595004 | 9.201364336 | 1.52E-19 | 6.41E-19 | 32.83612683 |
| CDKN2A | 1.077230477 | 1.742479441 | 9.193666177 | 1.62E-19 | 6.85E-19 | 32.76998829 |
| APOBR | 0.848246854 | 2.139200165 | 9.193636065 | 1.62E-19 | 6.85E-19 | 32.76972968 |
| E2F3 | 0.622426774 | 2.631747845 | 9.191365522 | 1.66E-19 | 6.98E-19 | 32.75023161 |
| UBE2L6 | 0.823490495 | 5.506182527 | 9.189120385 | 1.69E-19 | 7.12E-19 | 32.73095582 |
| APOC1 | 1.085204092 | 4.992978879 | 9.185526578 | 1.74E-19 | 7.34E-19 | 32.70010944 |
| RRAS | -0.687318362 | 4.884469438 | -9.179531398 | 1.83E-19 | 7.72E-19 | 32.64867492 |
| SETBP1 | -0.711340159 | 1.762033776 | -9.177030058 | 1.87E-19 | 7.89E-19 | 32.62722377 |
| GADD45GIP1 | 0.763278657 | 4.286605006 | 9.168596225 | 2.02E-19 | 8.48E-19 | 32.55493381 |
| CAPN9 | 0.98930926 | 1.489371878 | 9.157179445 | 2.23E-19 | 9.35E-19 | 32.45716781 |
| HYOU1 | 0.62080265 | 4.499443432 | 9.144211674 | 2.49E-19 | 1.04E-18 | 32.34624861 |
| HIST1H4K | 0.596151767 | 0.78459131 | 9.1426769 | 2.52E-19 | 1.06E-18 | 32.33313004 |
| CRIP1 | 0.829562509 | 1.827073447 | 9.136246483 | 2.67E-19 | 1.12E-18 | 32.27818652 |
| GAS6 | -0.870106778 | 3.956993155 | -9.134685079 | 2.70E-19 | 1.13E-18 | 32.26485046 |
| SH3KBP1 | -0.633722148 | 2.975464548 | -9.134133112 | 2.72E-19 | 1.14E-18 | 32.26013655 |
| LRFN4 | 0.843292636 | 2.691287424 | 9.132567556 | 2.75E-19 | 1.15E-18 | 32.24676772 |
| EFNB2 | -0.770982446 | 3.224027486 | -9.130171235 | 2.81E-19 | 1.18E-18 | 32.22630857 |
| DDIT4L | -0.587610895 | 0.747777821 | -9.12469447 | 2.95E-19 | 1.23E-18 | 32.17956696 |
| CMYA5 | -0.81769772 | 1.603168533 | -9.122489408 | 3.01E-19 | 1.26E-18 | 32.16075468 |
| SFXN5 | 0.679226371 | 1.756158599 | 9.112753376 | 3.27E-19 | 1.36E-18 | 32.07773997 |
| PARP10 | 0.864423937 | 3.681148905 | 9.108111439 | 3.40E-19 | 1.42E-18 | 32.03818747 |
| AURKAIP1 | 0.77607828 | 5.079176294 | 9.106544621 | 3.45E-19 | 1.44E-18 | 32.02484105 |
| GOLGA8B | -0.642967298 | 1.284790431 | -9.104639564 | 3.51E-19 | 1.46E-18 | 32.00861616 |
| GPX2 | -1.015082187 | 1.067703516 | -9.101368285 | 3.61E-19 | 1.50E-18 | 31.9807624 |
| SLC1A4 | 0.830114076 | 3.808300406 | 9.100147365 | 3.64E-19 | 1.52E-18 | 31.97036894 |
| PDLIM1 | -0.924454212 | 5.679771455 | -9.092810245 | 3.88E-19 | 1.61E-18 | 31.90793501 |
| HDAC11 | 0.754236372 | 3.086764162 | 9.089507394 | 3.99E-19 | 1.66E-18 | 31.87984431 |
| PTMS | 0.805631891 | 7.531834833 | 9.080757232 | 4.31E-19 | 1.79E-18 | 31.80546728 |
| TMPO | 0.58579495 | 3.658069915 | 9.071464156 | 4.66E-19 | 1.93E-18 | 31.72654381 |
| SLC16A2 | -0.814249826 | 2.864976333 | -9.067399611 | 4.83E-19 | 2.00E-18 | 31.69204693 |
| STAC | -0.615021726 | 0.635581703 | -9.06718331 | 4.84E-19 | 2.00E-18 | 31.69021151 |
| ADAM12 | 1.029928167 | 3.109845836 | 9.066587413 | 4.86E-19 | 2.01E-18 | 31.6851552 |
| MYL7 | -0.590505494 | 0.398570253 | -9.065299844 | 4.92E-19 | 2.03E-18 | 31.67423093 |
| C1R | -0.932580835 | 5.246905358 | -9.064445176 | 4.95E-19 | 2.05E-18 | 31.6669803 |
| CDC14A | -0.731775632 | 1.467007889 | -9.063113199 | 5.01E-19 | 2.07E-18 | 31.6556816 |
| MT-ND6 | -0.96545903 | 10.366409 | -9.062939855 | 5.02E-19 | 2.07E-18 | 31.65421129 |
| CGN | 0.917129216 | 3.585915668 | 9.056873598 | 5.29E-19 | 2.18E-18 | 31.6027725 |
| HIST1H2BF | 0.937284795 | 1.046230285 | 9.05682395 | 5.29E-19 | 2.18E-18 | 31.60235163 |
| PRRG2 | 0.672500511 | 2.925225721 | 9.048298609 | 5.69E-19 | 2.35E-18 | 31.53011216 |
| SLCO3A1 | -0.617810204 | 1.588519315 | -9.047141587 | 5.75E-19 | 2.37E-18 | 31.52031271 |
| TNFRSF1B | -0.792991549 | 3.418282578 | -9.045439561 | 5.83E-19 | 2.40E-18 | 31.50589931 |
| SLC16A4 | -0.787406391 | 1.996702702 | -9.044961391 | 5.85E-19 | 2.41E-18 | 31.50185041 |
| TDRP | -0.76738638 | 2.044701761 | -9.040166963 | 6.10E-19 | 2.51E-18 | 31.46126397 |
| KDELR3 | 0.862938572 | 4.154288908 | 9.039766769 | 6.12E-19 | 2.52E-18 | 31.45787705 |
| ZNF281 | 0.6497865 | 3.433410238 | 9.030012305 | 6.65E-19 | 2.73E-18 | 31.37536341 |
| RARA | 0.879191898 | 4.378344818 | 9.024395578 | 6.98E-19 | 2.86E-18 | 31.32788644 |
| TRPS1 | 1.038677609 | 5.159862127 | 9.022946313 | 7.07E-19 | 2.90E-18 | 31.31564031 |
| RET | 1.445480338 | 2.414440981 | 9.020440499 | 7.22E-19 | 2.96E-18 | 31.2944705 |
| TCF7 | -0.641856512 | 1.423976021 | -9.016156237 | 7.49E-19 | 3.07E-18 | 31.25828776 |
| SLC26A3 | -0.826948348 | 0.546216095 | -9.012421652 | 7.73E-19 | 3.16E-18 | 31.22675955 |
| SPR | 0.722382888 | 4.975344859 | 8.99893803 | 8.68E-19 | 3.55E-18 | 31.11302279 |
| BICDL2 | 0.807664203 | 2.628378774 | 8.996805559 | 8.84E-19 | 3.61E-18 | 31.09504863 |
| ALDH3A2 | -0.753914383 | 3.750464449 | -8.994939055 | 8.98E-19 | 3.67E-18 | 31.07931932 |
| FBLN2 | -1.12016235 | 4.720278814 | -8.991235337 | 9.27E-19 | 3.78E-18 | 31.04811596 |
| ITGAX | 0.641200266 | 1.86191512 | 8.980031144 | 1.02E-18 | 4.16E-18 | 30.95379044 |
| C1orf210 | 0.654880511 | 3.225573897 | 8.966087669 | 1.15E-18 | 4.67E-18 | 30.83654715 |
| TIMP2 | -0.799779488 | 6.10902908 | -8.965946245 | 1.15E-18 | 4.68E-18 | 30.8353588 |
| MLLT11 | 0.610973096 | 1.894026219 | 8.961168487 | 1.20E-18 | 4.87E-18 | 30.79522238 |
| ADCY6 | -0.606533962 | 3.370148798 | -8.960019773 | 1.21E-18 | 4.92E-18 | 30.78557519 |
| TENM3 | -0.634411737 | 1.075727634 | -8.958372455 | 1.23E-18 | 4.99E-18 | 30.77174248 |
| RAB17 | 0.788415814 | 2.590282645 | 8.956056781 | 1.25E-18 | 5.08E-18 | 30.75230128 |
| GAA | 0.620159891 | 4.887677863 | 8.950924999 | 1.31E-18 | 5.31E-18 | 30.70923316 |
| SCX | 0.635848102 | 0.902390495 | 8.94938534 | 1.32E-18 | 5.37E-18 | 30.6963159 |
| MTFR1 | 0.637127171 | 3.076154105 | 8.946586745 | 1.36E-18 | 5.50E-18 | 30.67284152 |
| ZNF692 | 0.659755878 | 2.973783018 | 8.940659959 | 1.42E-18 | 5.78E-18 | 30.62314937 |
| GPR87 | -0.789381794 | 0.851679736 | -8.937343706 | 1.47E-18 | 5.94E-18 | 30.59535736 |
| MT-ND3 | -0.827052657 | 11.49273624 | -8.93276649 | 1.52E-18 | 6.17E-18 | 30.55701263 |
| SYT13 | 1.711286964 | 2.000994673 | 8.932376827 | 1.53E-18 | 6.19E-18 | 30.5537491 |
| APOL3 | -0.742886522 | 2.783641653 | -8.926509549 | 1.61E-18 | 6.50E-18 | 30.50462416 |
| KLF5 | -1.104497628 | 2.617017142 | -8.916319667 | 1.75E-18 | 7.07E-18 | 30.41937454 |
| HDGFL2 | 0.609023022 | 3.654273081 | 8.910135874 | 1.85E-18 | 7.45E-18 | 30.36768186 |
| MRPL27 | 0.594372643 | 3.766623942 | 8.905120679 | 1.93E-18 | 7.76E-18 | 30.32578101 |
| CLPSL1 | 1.039902976 | 1.421770279 | 8.902151564 | 1.97E-18 | 7.96E-18 | 30.30098445 |
| OAS2 | 1.124055304 | 3.762844352 | 8.900955589 | 1.99E-18 | 8.04E-18 | 30.29099831 |
| GRAMD2A | -0.85289976 | 1.432930785 | -8.899515005 | 2.02E-18 | 8.13E-18 | 30.2789713 |
| RNASET2 | 0.598313389 | 2.869246291 | 8.890943101 | 2.17E-18 | 8.73E-18 | 30.20744229 |
| SDC3 | -0.601687985 | 4.093878248 | -8.888825329 | 2.21E-18 | 8.89E-18 | 30.18977967 |
| KDM4B | 0.809357533 | 3.191674571 | 8.88048858 | 2.37E-18 | 9.53E-18 | 30.12028541 |
| DSG3 | -1.303581727 | 1.32907969 | -8.880216556 | 2.38E-18 | 9.55E-18 | 30.11801882 |
| DNPH1 | 0.797715188 | 4.000993206 | 8.876479208 | 2.45E-18 | 9.84E-18 | 30.08688408 |
| BCAT2 | 0.623486122 | 3.182665846 | 8.875899194 | 2.46E-18 | 9.89E-18 | 30.08205318 |
| NEURL1 | 1.045655653 | 1.289044216 | 8.858506114 | 2.85E-18 | 1.14E-17 | 29.93731614 |
| CLGN | 1.233969871 | 2.092061464 | 8.85590497 | 2.92E-18 | 1.17E-17 | 29.91569205 |
| C6orf132 | 0.588235169 | 2.935544147 | 8.85579395 | 2.92E-18 | 1.17E-17 | 29.91476923 |
| F13A1 | -1.150512402 | 3.232655281 | -8.843343319 | 3.24E-18 | 1.29E-17 | 29.81134173 |
| CCDC88C | 0.590236784 | 2.56898638 | 8.84074839 | 3.31E-18 | 1.32E-17 | 29.78980171 |
| ATP5MC1 | 0.649153229 | 4.327006424 | 8.838467086 | 3.38E-18 | 1.34E-17 | 29.77086963 |
| CXCL14 | -1.94348213 | 5.67399938 | -8.832248531 | 3.56E-18 | 1.42E-17 | 29.71928485 |
| AHR | -0.7799839 | 3.803966481 | -8.83001606 | 3.63E-18 | 1.44E-17 | 29.70077362 |
| B3GNT5 | -0.671517842 | 0.996130711 | -8.827507363 | 3.70E-18 | 1.47E-17 | 29.67997687 |
| STAB1 | -0.659637854 | 2.527190275 | -8.827172194 | 3.71E-18 | 1.48E-17 | 29.67719876 |
| MS4A4A | -0.755198832 | 2.294911305 | -8.824476735 | 3.80E-18 | 1.51E-17 | 29.65486031 |
| ELMO3 | 0.7898978 | 3.47089715 | 8.81401081 | 4.15E-18 | 1.64E-17 | 29.56818141 |
| MSI2 | 0.652788653 | 2.683247415 | 8.798842135 | 4.71E-18 | 1.86E-17 | 29.44271471 |
| RRBP1 | 0.604486518 | 5.454395092 | 8.794253245 | 4.89E-18 | 1.93E-17 | 29.40479543 |
| JOSD2 | 0.728726939 | 3.318887818 | 8.791496216 | 5.01E-18 | 1.98E-17 | 29.3820217 |
| APBB1 | -0.62403106 | 2.440831112 | -8.790328236 | 5.06E-18 | 2.00E-17 | 29.37237579 |
| GPR160 | 0.924302759 | 3.284701059 | 8.781729569 | 5.43E-18 | 2.14E-17 | 29.3013973 |
| PHLDA2 | 0.981579847 | 2.875414687 | 8.77873119 | 5.57E-18 | 2.20E-17 | 29.27666127 |
| TINCR | 1.10281518 | 1.793075049 | 8.778653521 | 5.57E-18 | 2.20E-17 | 29.27602062 |
| CACNG4 | 1.594700679 | 3.812992118 | 8.777801504 | 5.61E-18 | 2.21E-17 | 29.26899306 |
| ADGRB2 | 0.954338116 | 1.947785211 | 8.770851453 | 5.95E-18 | 2.34E-17 | 29.21169043 |
| FUT2 | 0.768678425 | 1.630945588 | 8.761272291 | 6.45E-18 | 2.53E-17 | 29.13277643 |
| KLK8 | -0.906494157 | 0.689531807 | -8.761002831 | 6.46E-18 | 2.54E-17 | 29.13055769 |
| IRS1 | -0.895860545 | 2.84732652 | -8.753031637 | 6.90E-18 | 2.71E-17 | 29.0649499 |
| PMP22 | -0.823650428 | 5.475997835 | -8.745760962 | 7.33E-18 | 2.87E-17 | 29.00515365 |
| FGFR3 | 1.166235982 | 2.143021517 | 8.738728596 | 7.78E-18 | 3.04E-17 | 28.94735894 |
| MICB | 0.723225682 | 1.930204827 | 8.735117556 | 8.01E-18 | 3.13E-17 | 28.91769792 |
| GNAZ | -0.611284832 | 1.796846668 | -8.721696536 | 8.96E-18 | 3.50E-17 | 28.80755252 |
| ADAMTS4 | 0.716632747 | 2.036351439 | 8.720919799 | 9.02E-18 | 3.52E-17 | 28.80118246 |
| B4GALNT3 | 0.949592456 | 2.142493923 | 8.718911767 | 9.17E-18 | 3.58E-17 | 28.7847168 |
| RFTN1 | -0.665613763 | 3.071074215 | -8.716377374 | 9.36E-18 | 3.65E-17 | 28.7639398 |
| F2RL2 | 1.161960643 | 2.736325282 | 8.712050595 | 9.71E-18 | 3.78E-17 | 28.72848109 |
| CAMK2D | -0.669338676 | 2.666055159 | -8.708768772 | 9.98E-18 | 3.88E-17 | 28.70159633 |
| MGST1 | -1.063824457 | 4.247628035 | -8.698392823 | 1.09E-17 | 4.23E-17 | 28.61665506 |
| XDH | -0.86266283 | 1.015311486 | -8.69094464 | 1.16E-17 | 4.49E-17 | 28.55573658 |
| CDC25B | 0.775327022 | 3.661409139 | 8.689703153 | 1.17E-17 | 4.53E-17 | 28.54558698 |
| MYBL1 | 0.806768771 | 1.436325294 | 8.689320116 | 1.17E-17 | 4.55E-17 | 28.54245576 |
| TMEM160 | 0.683538903 | 2.690773013 | 8.68882521 | 1.18E-17 | 4.57E-17 | 28.53841024 |
| AFP | -0.693694699 | 0.435097804 | -8.681642661 | 1.25E-17 | 4.84E-17 | 28.47972059 |
| 8-Mar | -0.611145599 | 3.033282498 | -8.680464571 | 1.26E-17 | 4.88E-17 | 28.47009833 |
| ASB13 | 0.758441922 | 4.443034895 | 8.673343581 | 1.34E-17 | 5.18E-17 | 28.41196091 |
| SIX2 | 1.030141017 | 1.684422166 | 8.669409115 | 1.38E-17 | 5.34E-17 | 28.37985706 |
| TPBG | 0.713745852 | 3.463280401 | 8.665948778 | 1.42E-17 | 5.50E-17 | 28.35163256 |
| FABP5 | -0.983929432 | 2.562869272 | -8.665004539 | 1.43E-17 | 5.54E-17 | 28.34393253 |
| THEM6 | 0.711748332 | 4.10604867 | 8.664290726 | 1.44E-17 | 5.57E-17 | 28.33811206 |
| STAT1 | 0.895182391 | 5.605280854 | 8.658995788 | 1.51E-17 | 5.81E-17 | 28.29495004 |
| DCHS1 | -0.599014419 | 2.167993034 | -8.658281442 | 1.51E-17 | 5.85E-17 | 28.28912878 |
| LAMB2 | -0.71111453 | 5.366506413 | -8.654337637 | 1.57E-17 | 6.03E-17 | 28.25699808 |
| COPZ2 | -0.794371776 | 3.126171504 | -8.65214413 | 1.59E-17 | 6.14E-17 | 28.23913288 |
| CISH | 0.845078825 | 3.30892645 | 8.648801272 | 1.64E-17 | 6.31E-17 | 28.21191438 |
| GALK1 | 0.604864718 | 2.602611996 | 8.644236227 | 1.70E-17 | 6.54E-17 | 28.17475948 |
| CEACAM6 | 2.18143626 | 3.401155341 | 8.639699803 | 1.77E-17 | 6.79E-17 | 28.1378547 |
| OMD | -1.035411043 | 2.219092555 | -8.627596176 | 1.95E-17 | 7.49E-17 | 28.03947279 |
| TNN | -0.851755497 | 1.415326377 | -8.627504063 | 1.95E-17 | 7.50E-17 | 28.03872454 |
| FRMD6 | -0.735750162 | 3.158374284 | -8.625979948 | 1.98E-17 | 7.59E-17 | 28.02634483 |
| CAMP | 0.884429849 | 0.958098435 | 8.619666458 | 2.08E-17 | 7.98E-17 | 27.97508373 |
| SLPI | -1.986418611 | 4.939905147 | -8.607366639 | 2.30E-17 | 8.82E-17 | 27.8753131 |
| CLEC2B | -0.590776126 | 2.337081221 | -8.602282489 | 2.40E-17 | 9.19E-17 | 27.83410953 |
| C11orf96 | -0.817100494 | 2.299372919 | -8.600650175 | 2.43E-17 | 9.31E-17 | 27.8208853 |
| ETNK2 | 1.008361603 | 3.196420119 | 8.597034444 | 2.51E-17 | 9.58E-17 | 27.79160028 |
| APOL6 | -0.784477556 | 3.292934062 | -8.589306118 | 2.67E-17 | 1.02E-16 | 27.72904246 |
| RDX | -0.591818535 | 3.322904053 | -8.574423187 | 3.02E-17 | 1.15E-16 | 27.60871107 |
| VSIG4 | -0.874063862 | 2.865867145 | -8.572661286 | 3.06E-17 | 1.17E-16 | 27.59447798 |
| CENPW | 0.938430067 | 3.31628172 | 8.560973507 | 3.37E-17 | 1.28E-16 | 27.50012661 |
| C3orf52 | 0.730473331 | 1.624219459 | 8.560502234 | 3.38E-17 | 1.28E-16 | 27.49632458 |
| GLIS2 | 0.658928537 | 2.985004272 | 8.559603538 | 3.41E-17 | 1.29E-16 | 27.48907479 |
| USP18 | 0.72553858 | 2.56428267 | 8.552308048 | 3.62E-17 | 1.37E-16 | 27.43024693 |
| DUSP23 | 0.663219101 | 5.331763431 | 8.550503684 | 3.67E-17 | 1.39E-16 | 27.41570412 |
| ADAM15 | 0.631192302 | 5.09226222 | 8.547114541 | 3.77E-17 | 1.43E-16 | 27.38839566 |
| TSPAN4 | -0.589733438 | 2.91529765 | -8.545867865 | 3.81E-17 | 1.45E-16 | 27.37835281 |
| CHST2 | -0.646324972 | 1.432802632 | -8.544767298 | 3.85E-17 | 1.46E-16 | 27.36948806 |
| ALDH4A1 | -0.717761086 | 2.976477437 | -8.541736842 | 3.94E-17 | 1.49E-16 | 27.34508382 |
| CDCP1 | 0.767519842 | 3.072712329 | 8.534620267 | 4.18E-17 | 1.58E-16 | 27.28780424 |
| SREBF1 | 0.883766556 | 4.981796838 | 8.532884737 | 4.24E-17 | 1.60E-16 | 27.27384179 |
| H1F0 | 0.682526635 | 7.135212832 | 8.532165538 | 4.26E-17 | 1.61E-16 | 27.26805652 |
| TMEM145 | 0.793216358 | 0.889264281 | 8.53006824 | 4.34E-17 | 1.64E-16 | 27.25118825 |
| SLFN5 | -0.764938167 | 2.827417886 | -8.529415015 | 4.36E-17 | 1.65E-16 | 27.2459352 |
| HOOK1 | 0.637686239 | 2.870312479 | 8.527417865 | 4.43E-17 | 1.67E-16 | 27.22987691 |
| C1orf53 | 0.614200784 | 1.781291737 | 8.526598567 | 4.46E-17 | 1.69E-16 | 27.22329022 |
| HIST3H2A | 1.305224586 | 3.597436436 | 8.522956857 | 4.60E-17 | 1.74E-16 | 27.19401974 |
| NCAPD2 | 0.613510878 | 3.862015735 | 8.520109671 | 4.70E-17 | 1.77E-16 | 27.171143 |
| ABO | -0.609324858 | 0.739507294 | -8.504775346 | 5.33E-17 | 2.01E-16 | 27.04805039 |
| ANXA9 | 1.132429703 | 3.988258727 | 8.49693691 | 5.68E-17 | 2.14E-16 | 26.98520515 |
| UBE2E3 | -0.7936031 | 3.567241016 | -8.492770317 | 5.87E-17 | 2.21E-16 | 26.95182009 |
| RNF223 | 0.66008332 | 1.27737588 | 8.488982117 | 6.06E-17 | 2.28E-16 | 26.92147953 |
| MT-ND2 | -0.821016647 | 11.44074536 | -8.482866774 | 6.37E-17 | 2.39E-16 | 26.87252569 |
| NINJ1 | 0.594444052 | 5.318870358 | 8.477446437 | 6.65E-17 | 2.50E-16 | 26.82916159 |
| HIST1H2AG | 0.793759666 | 1.130179835 | 8.471461248 | 6.98E-17 | 2.62E-16 | 26.78130709 |
| DIRAS3 | -0.81385273 | 1.106810826 | -8.468058213 | 7.18E-17 | 2.69E-16 | 26.75411155 |
| DLK1 | -0.930906483 | 0.373870302 | -8.466168447 | 7.29E-17 | 2.73E-16 | 26.73901356 |
| TMSB10 | 0.77265369 | 10.7173305 | 8.466037684 | 7.30E-17 | 2.73E-16 | 26.73796896 |
| HSPA6 | 0.766002931 | 1.680149807 | 8.462718946 | 7.50E-17 | 2.80E-16 | 26.71146203 |
| FAS | -0.602389509 | 2.23546585 | -8.455719151 | 7.93E-17 | 2.96E-16 | 26.65558456 |
| EVL | 1.010951628 | 4.224271329 | 8.45287721 | 8.12E-17 | 3.03E-16 | 26.63290983 |
| FAM222A | 0.626092245 | 1.712015911 | 8.452704785 | 8.13E-17 | 3.03E-16 | 26.63153434 |
| SLC2A4RG | 0.656772495 | 5.054402685 | 8.446745936 | 8.53E-17 | 3.18E-16 | 26.58401385 |
| GALNT6 | 1.227709266 | 4.290647755 | 8.441531187 | 8.90E-17 | 3.31E-16 | 26.54245181 |
| IL18 | 0.684888378 | 2.069834855 | 8.431179722 | 9.67E-17 | 3.60E-16 | 26.46001719 |
| COL15A1 | -0.745893927 | 4.26688308 | -8.426127029 | 1.01E-16 | 3.74E-16 | 26.41981235 |
| TNFRSF4 | 0.60631492 | 1.466524638 | 8.419699839 | 1.06E-16 | 3.94E-16 | 26.36870143 |
| MIA | -1.130391014 | 1.116710152 | -8.419358206 | 1.06E-16 | 3.95E-16 | 26.36598562 |
| CCDC124 | 0.672618888 | 4.627763124 | 8.413984941 | 1.11E-16 | 4.12E-16 | 26.32328397 |
| ZNF469 | 0.600459657 | 1.438904597 | 8.41299366 | 1.12E-16 | 4.15E-16 | 26.31540884 |
| SYT8 | -0.858354569 | 0.829191519 | -8.409927797 | 1.15E-16 | 4.25E-16 | 26.29105765 |
| NAT1 | 1.862138991 | 3.852187132 | 8.402099481 | 1.22E-16 | 4.52E-16 | 26.22891557 |
| ROPN1B | -0.834286997 | 0.617737189 | -8.390171731 | 1.35E-16 | 4.97E-16 | 26.13433064 |
| IGSF3 | 0.72084694 | 3.597038734 | 8.379855157 | 1.46E-16 | 5.39E-16 | 26.05261843 |
| ACER2 | -0.680571192 | 1.644029767 | -8.375717536 | 1.51E-16 | 5.57E-16 | 26.01987161 |
| ATP6V0E2 | 0.71582217 | 4.142118 | 8.374996173 | 1.52E-16 | 5.60E-16 | 26.01416393 |
| SFXN2 | 0.665103024 | 2.146805408 | 8.371021985 | 1.57E-16 | 5.78E-16 | 25.98272654 |
| PYGL | -0.924439257 | 3.651532306 | -8.361611837 | 1.69E-16 | 6.23E-16 | 25.90834153 |
| GMDS | 0.605206019 | 2.907512936 | 8.356102085 | 1.77E-16 | 6.50E-16 | 25.86482281 |
| HIST1H4E | 0.907465193 | 1.19000999 | 8.349567798 | 1.86E-16 | 6.84E-16 | 25.81324489 |
| PPIF | 0.688486599 | 5.01637311 | 8.347979116 | 1.89E-16 | 6.93E-16 | 25.80071017 |
| ZNF552 | 1.062114948 | 3.983066509 | 8.347092259 | 1.90E-16 | 6.98E-16 | 25.79371379 |
| GALNT7 | 0.913976009 | 3.787683056 | 8.340599948 | 2.00E-16 | 7.33E-16 | 25.74251632 |
| GPER1 | -0.793750078 | 1.209043403 | -8.340372173 | 2.01E-16 | 7.35E-16 | 25.74072075 |
| CCR4 | 0.593757353 | 0.966456838 | 8.336164412 | 2.07E-16 | 7.59E-16 | 25.70755869 |
| OAS1 | 0.958478347 | 3.470586228 | 8.333422119 | 2.12E-16 | 7.75E-16 | 25.68595424 |
| SLC16A6 | 1.235802876 | 2.475745112 | 8.328790941 | 2.20E-16 | 8.04E-16 | 25.64948307 |
| MAP2 | -0.640630856 | 1.051642212 | -8.327719151 | 2.22E-16 | 8.11E-16 | 25.64104515 |
| PAQR6 | 0.742893099 | 1.5802375 | 8.3244202 | 2.28E-16 | 8.32E-16 | 25.61507945 |
| SYCE3 | 0.686657095 | 1.231617314 | 8.321657027 | 2.33E-16 | 8.50E-16 | 25.59333786 |
| ATP11B | -0.676129694 | 2.592372572 | -8.315587763 | 2.44E-16 | 8.91E-16 | 25.54560538 |
| ERGIC1 | 0.594504636 | 4.509796284 | 8.306488622 | 2.63E-16 | 9.57E-16 | 25.47410221 |
| ANG | -0.745022186 | 3.01539883 | -8.298133089 | 2.81E-16 | 1.02E-15 | 25.40850395 |
| H1FX | 0.640757664 | 6.085996283 | 8.292820813 | 2.93E-16 | 1.06E-15 | 25.36682853 |
| EIF4EBP1 | 0.918754515 | 5.149140505 | 8.292106535 | 2.95E-16 | 1.07E-15 | 25.36122675 |
| GPD1L | -0.753065075 | 4.027079429 | -8.284187468 | 3.14E-16 | 1.14E-15 | 25.29914972 |
| MS4A15 | 0.881099869 | 0.895749328 | 8.279179088 | 3.27E-16 | 1.18E-15 | 25.25991668 |
| TP53INP1 | 0.764378925 | 4.088442349 | 8.277638779 | 3.31E-16 | 1.20E-15 | 25.24785495 |
| PRLR | 1.006669917 | 3.891593313 | 8.272746075 | 3.44E-16 | 1.25E-15 | 25.20955485 |
| IGFBP3 | -0.740135196 | 4.825576382 | -8.267924628 | 3.57E-16 | 1.29E-15 | 25.17183231 |
| KLK6 | -1.433141963 | 1.466561505 | -8.265899821 | 3.63E-16 | 1.31E-15 | 25.15599628 |
| PACSIN3 | 0.783227939 | 3.700004478 | 8.262638836 | 3.72E-16 | 1.35E-15 | 25.13049934 |
| AMOT | -0.674639402 | 2.37516367 | -8.244835234 | 4.29E-16 | 1.55E-15 | 24.99145523 |
| GADD45G | 0.956500702 | 2.918867416 | 8.243329233 | 4.34E-16 | 1.56E-15 | 24.97970581 |
| IGF2BP2 | -0.754464105 | 0.932801457 | -8.241808692 | 4.39E-16 | 1.58E-15 | 24.9678449 |
| IL20RA | -0.991150503 | 2.04460331 | -8.239550306 | 4.47E-16 | 1.61E-15 | 24.95023208 |
| DPM3 | 0.711493892 | 4.876174297 | 8.239256645 | 4.48E-16 | 1.61E-15 | 24.94794218 |
| NPNT | 1.265497562 | 4.237989322 | 8.23918547 | 4.49E-16 | 1.61E-15 | 24.94738718 |
| HIST1H4D | 0.682157769 | 0.75498792 | 8.231727912 | 4.76E-16 | 1.71E-15 | 24.88925969 |
| GSR | 0.687784744 | 4.437992588 | 8.230524884 | 4.80E-16 | 1.73E-15 | 24.87988716 |
| SERPINF2 | -0.68498725 | 1.436368117 | -8.230458617 | 4.81E-16 | 1.73E-15 | 24.87937093 |
| GDF15 | 1.220478259 | 2.552795943 | 8.219838501 | 5.23E-16 | 1.87E-15 | 24.79668562 |
| RIPK4 | -0.745160816 | 1.991621108 | -8.212483701 | 5.54E-16 | 1.99E-15 | 24.73947909 |
| HMGCS2 | -1.901096402 | 2.315522986 | -8.209205197 | 5.68E-16 | 2.04E-15 | 24.71399323 |
| SLC18B1 | -0.641807225 | 2.769966337 | -8.204908285 | 5.88E-16 | 2.11E-15 | 24.68060442 |
| CLU | -1.155500081 | 6.337375227 | -8.198465476 | 6.19E-16 | 2.21E-15 | 24.63057038 |
| PCDH19 | -0.599033228 | 0.70234125 | -8.191569749 | 6.53E-16 | 2.33E-15 | 24.57705795 |
| COL12A1 | 1.07654912 | 5.016703646 | 8.186308089 | 6.81E-16 | 2.43E-15 | 24.53625336 |
| SLFN11 | -0.614163214 | 2.381103241 | -8.186185641 | 6.81E-16 | 2.43E-15 | 24.53530404 |
| PLAU | 0.908881453 | 4.565074942 | 8.183249824 | 6.97E-16 | 2.49E-15 | 24.51254704 |
| PTK7 | 0.826196993 | 3.85099133 | 8.182053522 | 7.04E-16 | 2.51E-15 | 24.50327599 |
| EFNA5 | -0.892805107 | 1.999598563 | -8.168450055 | 7.83E-16 | 2.79E-15 | 24.39793774 |
| HACD3 | 0.615926093 | 4.913555096 | 8.139953743 | 9.79E-16 | 3.47E-15 | 24.17778548 |
| PKIB | 1.146947666 | 3.26965676 | 8.138629673 | 9.89E-16 | 3.51E-15 | 24.16757294 |
| PPP1R16A | 0.616319042 | 2.471493826 | 8.127266764 | 1.08E-15 | 3.83E-15 | 24.07999209 |
| ATP13A4 | -0.714937669 | 0.772951477 | -8.116254913 | 1.18E-15 | 4.17E-15 | 23.99522165 |
| SOX18 | -0.677037995 | 2.422355575 | -8.107161708 | 1.27E-15 | 4.47E-15 | 23.92529879 |
| HIPK2 | -0.699127939 | 2.878560271 | -8.105536765 | 1.28E-15 | 4.52E-15 | 23.91281107 |
| HCAR1 | -0.964403779 | 2.765973692 | -8.089321178 | 1.45E-15 | 5.12E-15 | 23.78831691 |
| ELF3 | 0.767417984 | 4.450683877 | 8.081831454 | 1.54E-15 | 5.41E-15 | 23.7308905 |
| ISG20 | 0.60281212 | 1.768242935 | 8.080235486 | 1.56E-15 | 5.48E-15 | 23.7186598 |
| ZBTB41 | 0.589760138 | 3.003077008 | 8.073033689 | 1.65E-15 | 5.79E-15 | 23.6634958 |
| SH2D2A | 0.618744885 | 1.226720775 | 8.069047452 | 1.70E-15 | 5.97E-15 | 23.63298118 |
| PLPP5 | 0.761316976 | 3.800486027 | 8.068355782 | 1.71E-15 | 6.00E-15 | 23.62768782 |
| MRPL41 | 0.69616781 | 4.498681328 | 8.067739998 | 1.72E-15 | 6.03E-15 | 23.62297557 |
| CERS6 | 0.767782754 | 3.584648312 | 8.05997186 | 1.83E-15 | 6.40E-15 | 23.563558 |
| ASPN | 1.364319298 | 4.595517446 | 8.050473575 | 1.97E-15 | 6.88E-15 | 23.49097655 |
| PTPN1 | 0.619311841 | 4.62820011 | 8.050259057 | 1.97E-15 | 6.89E-15 | 23.48933819 |
| LRRCC1 | 0.595685034 | 2.872687735 | 8.046907895 | 2.02E-15 | 7.07E-15 | 23.46374913 |
| EGLN3 | 0.874156094 | 2.35981761 | 8.043506412 | 2.08E-15 | 7.26E-15 | 23.43778561 |
| SAMD11 | 0.876835622 | 1.282773976 | 8.034788062 | 2.22E-15 | 7.75E-15 | 23.37128343 |
| PROM1 | -1.486755203 | 2.254658688 | -8.031960383 | 2.27E-15 | 7.92E-15 | 23.34972825 |
| CASP1 | -0.634176488 | 2.459816299 | -8.011255594 | 2.66E-15 | 9.25E-15 | 23.19210495 |
| CYFIP2 | 0.733851701 | 2.116671174 | 8.010906408 | 2.67E-15 | 9.27E-15 | 23.18944977 |
| HIST1H3E | 0.76181259 | 1.938429469 | 8.00974433 | 2.69E-15 | 9.36E-15 | 23.18061417 |
| SLC35F2 | 0.661248294 | 2.41855666 | 7.994423615 | 3.03E-15 | 1.05E-14 | 23.06423436 |
| HCFC1R1 | 0.587349824 | 5.16438073 | 7.99250859 | 3.08E-15 | 1.07E-14 | 23.04970145 |
| EYA2 | -0.926236002 | 2.409793579 | -7.988214151 | 3.18E-15 | 1.10E-14 | 23.01712283 |
| RNF128 | -0.795977276 | 1.290119498 | -7.988184649 | 3.18E-15 | 1.10E-14 | 23.01689907 |
| ATP2A3 | 0.958863703 | 3.421304167 | 7.983697915 | 3.29E-15 | 1.14E-14 | 22.98287858 |
| RBM47 | 0.618482442 | 3.955679061 | 7.979662447 | 3.40E-15 | 1.17E-14 | 22.95229449 |
| CHST1 | 0.99144931 | 2.064625435 | 7.977728897 | 3.45E-15 | 1.19E-14 | 22.93764538 |
| CST6 | 1.037541006 | 1.818533428 | 7.971533319 | 3.62E-15 | 1.25E-14 | 22.89072749 |
| GATA3 | 1.497604789 | 6.678536966 | 7.968714031 | 3.70E-15 | 1.27E-14 | 22.86938843 |
| FSIP1 | 1.17115882 | 1.97610646 | 7.967569676 | 3.73E-15 | 1.28E-14 | 22.8607288 |
| SECTM1 | 0.913933336 | 3.177900495 | 7.965914221 | 3.78E-15 | 1.30E-14 | 22.84820352 |
| EGFL7 | -0.839013585 | 3.32054808 | -7.964418542 | 3.82E-15 | 1.32E-14 | 22.83688913 |
| JAG1 | -0.585066196 | 3.337620475 | -7.963278983 | 3.85E-15 | 1.33E-14 | 22.82826997 |
| RASL10B | -0.603821154 | 0.99793301 | -7.959493674 | 3.97E-15 | 1.36E-14 | 22.7996474 |
| ZNF587 | 0.65931491 | 2.654484212 | 7.957247236 | 4.04E-15 | 1.39E-14 | 22.78266678 |
| SMOC1 | -0.914711994 | 1.052755444 | -7.955572768 | 4.09E-15 | 1.41E-14 | 22.77001244 |
| PHLDA1 | -0.792183587 | 2.906813622 | -7.952840538 | 4.18E-15 | 1.44E-14 | 22.74936948 |
| KANK4 | -0.763663455 | 1.360971479 | -7.952539147 | 4.18E-15 | 1.44E-14 | 22.74709275 |
| PAX9 | 0.589341884 | 1.106990985 | 7.949383204 | 4.29E-15 | 1.47E-14 | 22.72325725 |
| LRRN2 | 0.828371728 | 2.325434365 | 7.946808481 | 4.37E-15 | 1.50E-14 | 22.70381777 |
| CEACAM5 | 1.555196656 | 1.797886975 | 7.942398804 | 4.52E-15 | 1.55E-14 | 22.67053732 |
| KCNJ11 | 0.862817709 | 2.291556883 | 7.941361327 | 4.56E-15 | 1.57E-14 | 22.66270975 |
| PLEKHG6 | -0.618068116 | 2.083134826 | -7.934832612 | 4.79E-15 | 1.64E-14 | 22.61347296 |
| C1S | -0.963086234 | 5.466168388 | -7.932279141 | 4.89E-15 | 1.67E-14 | 22.59422568 |
| FAAH | 0.699968938 | 3.540990432 | 7.931178811 | 4.93E-15 | 1.69E-14 | 22.58593345 |
| LSM1 | 0.63002383 | 3.897207311 | 7.920395212 | 5.35E-15 | 1.83E-14 | 22.50472171 |
| SMIM1 | 0.626175495 | 1.670885365 | 7.918670796 | 5.42E-15 | 1.86E-14 | 22.49174429 |
| GBP5 | 0.909198959 | 1.513487881 | 7.918600666 | 5.43E-15 | 1.86E-14 | 22.49121656 |
| VMP1 | 0.604644941 | 4.935940828 | 7.911645195 | 5.72E-15 | 1.96E-14 | 22.4388981 |
| C14orf132 | -0.750897816 | 1.905399024 | -7.909885129 | 5.80E-15 | 1.98E-14 | 22.4256656 |
| MYO6 | 0.645042971 | 4.56396222 | 7.906671768 | 5.94E-15 | 2.03E-14 | 22.40151381 |
| MGP | -1.532245802 | 8.722824163 | -7.901650566 | 6.18E-15 | 2.11E-14 | 22.36379189 |
| VAV3 | 1.062349667 | 4.215730584 | 7.898824789 | 6.31E-15 | 2.15E-14 | 22.34257266 |
| MYO15B | -0.598814614 | 1.634315859 | -7.876244498 | 7.50E-15 | 2.55E-14 | 22.17325926 |
| HRAS | 0.622823264 | 3.918635761 | 7.875658364 | 7.53E-15 | 2.56E-14 | 22.16887009 |
| DNAJA4 | 0.687225066 | 3.415260271 | 7.874703178 | 7.58E-15 | 2.58E-14 | 22.16171795 |
| F7 | 0.832500434 | 1.14787975 | 7.874359823 | 7.60E-15 | 2.58E-14 | 22.1591472 |
| ASNS | 0.622154644 | 2.904134742 | 7.871176307 | 7.79E-15 | 2.64E-14 | 22.1353166 |
| PIK3R3 | 0.590639216 | 3.960277708 | 7.868214396 | 7.97E-15 | 2.70E-14 | 22.11315266 |
| ABCC5 | 0.679478608 | 2.730972202 | 7.866378772 | 8.08E-15 | 2.74E-14 | 22.09942048 |
| PPP1R1B | -1.644120825 | 3.131888764 | -7.861773054 | 8.37E-15 | 2.84E-14 | 22.06497814 |
| KCNK1 | 1.056418504 | 2.614365566 | 7.853451002 | 8.91E-15 | 3.02E-14 | 22.00279059 |
| PLVAP | -0.609104623 | 5.509034436 | -7.851266572 | 9.06E-15 | 3.07E-14 | 21.98647702 |
| EPS8L2 | 0.641001121 | 3.721918576 | 7.844981895 | 9.50E-15 | 3.21E-14 | 21.93956517 |
| TRPM4 | 0.60681258 | 3.234569019 | 7.837821396 | 1.00E-14 | 3.39E-14 | 21.8861571 |
| TLE1 | -0.69798729 | 3.074107807 | -7.83137037 | 1.05E-14 | 3.55E-14 | 21.83807848 |
| TFRC | 0.743469202 | 4.076546794 | 7.824785642 | 1.11E-14 | 3.73E-14 | 21.78904025 |
| ENDOD1 | -0.606018434 | 3.733114302 | -7.821238139 | 1.14E-14 | 3.83E-14 | 21.76263649 |
| MMP10 | 0.929311675 | 1.150278751 | 7.820080001 | 1.15E-14 | 3.86E-14 | 21.75401891 |
| GJA4 | -0.615782631 | 2.96170023 | -7.817277608 | 1.17E-14 | 3.94E-14 | 21.73317138 |
| RELB | 0.600963892 | 2.893079026 | 7.816099814 | 1.18E-14 | 3.98E-14 | 21.72441156 |
| FOXC1 | -1.094231733 | 2.071718333 | -7.813796313 | 1.20E-14 | 4.05E-14 | 21.70728276 |
| RSAD2 | 0.920000716 | 2.632899388 | 7.798962173 | 1.35E-14 | 4.52E-14 | 21.5970856 |
| TCEA3 | 0.722027024 | 4.144004379 | 7.793587581 | 1.40E-14 | 4.70E-14 | 21.55720651 |
| ZNF704 | -0.611644069 | 2.413516788 | -7.791618998 | 1.42E-14 | 4.76E-14 | 21.54260598 |
| ENO2 | 0.871477834 | 3.141380483 | 7.789772241 | 1.44E-14 | 4.83E-14 | 21.52891204 |
| ROPN1 | -0.653660338 | 0.464395877 | -7.77977072 | 1.56E-14 | 5.20E-14 | 21.45480043 |
| SLC17A9 | 0.659006188 | 1.195729485 | 7.775287298 | 1.61E-14 | 5.38E-14 | 21.42160607 |
| ASCL2 | 0.796298144 | 1.292322152 | 7.751312108 | 1.93E-14 | 6.42E-14 | 21.24439227 |
| ITPR2 | -0.766293268 | 2.982226968 | -7.750250091 | 1.94E-14 | 6.47E-14 | 21.23655377 |
| CCL2 | -0.90711551 | 3.433491828 | -7.746762631 | 1.99E-14 | 6.64E-14 | 21.21082047 |
| CALCRL | -0.64206339 | 2.479868391 | -7.743406586 | 2.04E-14 | 6.80E-14 | 21.18606676 |
| AGRN | 0.645225091 | 4.46847956 | 7.742992345 | 2.05E-14 | 6.82E-14 | 21.18301205 |
| NKD2 | 0.641731064 | 1.63844396 | 7.740909812 | 2.08E-14 | 6.93E-14 | 21.16765721 |
| SLC2A10 | 0.849544589 | 3.837634948 | 7.735657067 | 2.17E-14 | 7.20E-14 | 21.1289445 |
| FAM174B | 0.727520098 | 3.790260531 | 7.735348706 | 2.17E-14 | 7.21E-14 | 21.12667262 |
| ATP2C2 | 0.632567346 | 1.619665013 | 7.730396932 | 2.25E-14 | 7.48E-14 | 21.09020115 |
| SCARA3 | -0.78772601 | 3.408476536 | -7.730365387 | 2.25E-14 | 7.48E-14 | 21.08996888 |
| SEMA3E | -0.708342118 | 1.24507227 | -7.730140477 | 2.26E-14 | 7.50E-14 | 21.08831285 |
| EVA1B | 0.989787425 | 3.801989434 | 7.721735782 | 2.40E-14 | 7.97E-14 | 21.02645974 |
| MVP | 0.585226399 | 4.789315302 | 7.718942751 | 2.45E-14 | 8.14E-14 | 21.00591833 |
| VSNL1 | -0.663394763 | 1.112055519 | -7.717564382 | 2.48E-14 | 8.22E-14 | 20.99578356 |
| TSPAN1 | 1.495670504 | 4.16239303 | 7.71289364 | 2.57E-14 | 8.51E-14 | 20.96145307 |
| PTHLH | -1.026618582 | 1.578420056 | -7.710325362 | 2.62E-14 | 8.67E-14 | 20.94258395 |
| FUT8 | 0.665867147 | 3.368629845 | 7.697780534 | 2.87E-14 | 9.50E-14 | 20.85049901 |
| ERBB2 | 1.080842722 | 5.263799404 | 7.695487468 | 2.92E-14 | 9.66E-14 | 20.83368151 |
| CDON | -0.585081169 | 1.722322401 | -7.687768682 | 3.10E-14 | 1.02E-13 | 20.77710478 |
| MAN1A1 | -0.81055221 | 4.028778475 | -7.68287612 | 3.21E-14 | 1.06E-13 | 20.7412702 |
| SCUBE3 | 0.859274928 | 1.282269053 | 7.676409293 | 3.37E-14 | 1.11E-13 | 20.69393695 |
| EFEMP2 | -0.591063361 | 3.248394209 | -7.656982515 | 3.89E-14 | 1.28E-13 | 20.5519621 |
| CLTC | 0.601236946 | 5.813908856 | 7.650915084 | 4.07E-14 | 1.33E-13 | 20.50768694 |
| RALGPS2 | 0.780193241 | 2.94687682 | 7.64853148 | 4.15E-14 | 1.36E-13 | 20.49030205 |
| COL8A1 | 0.872940694 | 3.204180908 | 7.634688546 | 4.59E-14 | 1.50E-13 | 20.38943535 |
| RIMS4 | 1.200891833 | 1.612583998 | 7.619231676 | 5.15E-14 | 1.68E-13 | 20.27700466 |
| FOXA1 | 1.567723835 | 5.417935122 | 7.618352681 | 5.18E-14 | 1.69E-13 | 20.27061721 |
| LALBA | -0.812570488 | 0.243916212 | -7.615633746 | 5.29E-14 | 1.72E-13 | 20.25086359 |
| TCEAL3 | 0.792167907 | 4.107175734 | 7.613661259 | 5.37E-14 | 1.75E-13 | 20.23653707 |
| MREG | 0.621534004 | 3.894587532 | 7.612993076 | 5.39E-14 | 1.76E-13 | 20.23168471 |
| LMTK3 | 0.613849872 | 1.520672333 | 7.612494818 | 5.41E-14 | 1.76E-13 | 20.2280666 |
| FKBP5 | -0.833012627 | 3.074671863 | -7.605417709 | 5.70E-14 | 1.85E-13 | 20.17669921 |
| BCAS1 | 1.022735748 | 1.88711789 | 7.590501125 | 6.36E-14 | 2.07E-13 | 20.06857313 |
| HSPG2 | -0.762462848 | 3.992116175 | -7.586537335 | 6.55E-14 | 2.13E-13 | 20.03987318 |
| SPDEF | 1.487884313 | 5.835627765 | 7.586163407 | 6.57E-14 | 2.13E-13 | 20.03716644 |
| NME2 | 0.652450446 | 4.268315692 | 7.585653715 | 6.60E-14 | 2.14E-13 | 20.03347716 |
| GGH | 0.856932098 | 3.002230803 | 7.57976454 | 6.89E-14 | 2.23E-13 | 19.99086604 |
| ENTPD8 | 0.65155522 | 0.900495423 | 7.570293516 | 7.39E-14 | 2.39E-13 | 19.92240156 |
| TREM2 | 0.718030212 | 3.866169571 | 7.570005136 | 7.40E-14 | 2.39E-13 | 19.92031813 |
| SUSD4 | 0.918135539 | 2.503280192 | 7.560211706 | 7.95E-14 | 2.57E-13 | 19.8496073 |
| IFI35 | 0.715348966 | 4.229862743 | 7.554565481 | 8.29E-14 | 2.68E-13 | 19.80887808 |
| TRIM22 | -0.673249713 | 3.225832716 | -7.553535964 | 8.35E-14 | 2.69E-13 | 19.80145461 |
| EPSTI1 | 0.659951731 | 2.176000058 | 7.55084586 | 8.52E-14 | 2.75E-13 | 19.78206159 |
| KCNS3 | 0.732622527 | 3.252564141 | 7.541671961 | 9.11E-14 | 2.94E-13 | 19.71597401 |
| CSRP2 | -0.586815237 | 1.601547819 | -7.527031809 | 1.01E-13 | 3.26E-13 | 19.61065964 |
| P4HTM | 0.587401275 | 2.690469035 | 7.526238706 | 1.02E-13 | 3.28E-13 | 19.60495975 |
| HLA-C | 0.681037011 | 8.353245319 | 7.52410762 | 1.04E-13 | 3.33E-13 | 19.58964671 |
| ARHGAP40 | -1.137913453 | 1.442316043 | -7.52049814 | 1.06E-13 | 3.42E-13 | 19.56371959 |
| TSPAN12 | -0.693859088 | 2.065434384 | -7.504767059 | 1.19E-13 | 3.82E-13 | 19.45085449 |
| DCAF10 | 0.659216877 | 3.588616699 | 7.501734036 | 1.22E-13 | 3.90E-13 | 19.42911834 |
| NFKBIZ | -0.930163386 | 2.48391695 | -7.4974815 | 1.26E-13 | 4.02E-13 | 19.39865601 |
| IGFALS | 0.756203301 | 1.033345605 | 7.493941797 | 1.29E-13 | 4.12E-13 | 19.37331194 |
| CADM4 | 0.654748802 | 4.079961494 | 7.490853158 | 1.32E-13 | 4.21E-13 | 19.35120637 |
| PKIA | -0.716675774 | 1.122233688 | -7.480482822 | 1.42E-13 | 4.54E-13 | 19.27704599 |
| CRYBG1 | -0.789964688 | 3.485531932 | -7.479430314 | 1.43E-13 | 4.57E-13 | 19.26952451 |
| AEBP1 | 0.908483129 | 6.756203662 | 7.478773324 | 1.44E-13 | 4.59E-13 | 19.26483 |
| LILRB4 | 0.604171436 | 1.940595345 | 7.465097316 | 1.59E-13 | 5.06E-13 | 19.16719356 |
| HOXD8 | -0.678067634 | 2.038831243 | -7.464797221 | 1.60E-13 | 5.07E-13 | 19.16505293 |
| NFE2L3 | 0.691252118 | 2.694988634 | 7.463678133 | 1.61E-13 | 5.11E-13 | 19.15707098 |
| C5AR2 | 0.722643203 | 1.717594719 | 7.460124677 | 1.65E-13 | 5.24E-13 | 19.13173301 |
| DOK7 | 0.807821848 | 1.25929344 | 7.457785622 | 1.68E-13 | 5.33E-13 | 19.11506036 |
| EPHX2 | -0.690395258 | 2.838322946 | -7.443064905 | 1.87E-13 | 5.91E-13 | 19.01024126 |
| ARL4C | -0.705512699 | 3.674166051 | -7.442681772 | 1.87E-13 | 5.93E-13 | 19.00751568 |
| GNG13 | 0.616269346 | 0.587859503 | 7.442111555 | 1.88E-13 | 5.95E-13 | 19.00345942 |
| NOP53 | -0.639534015 | 5.385948314 | -7.441588339 | 1.89E-13 | 5.97E-13 | 18.99973777 |
| SERPINE1 | 0.905221232 | 4.046560677 | 7.439728597 | 1.91E-13 | 6.05E-13 | 18.98651127 |
| ISYNA1 | 0.743625743 | 4.087298696 | 7.421188334 | 2.19E-13 | 6.91E-13 | 18.85481772 |
| PANX2 | 0.6114099 | 1.201690205 | 7.406976635 | 2.42E-13 | 7.64E-13 | 18.75407343 |
| IGSF1 | -0.824735797 | 0.766738677 | -7.395220385 | 2.64E-13 | 8.31E-13 | 18.6708686 |
| NFIA | -0.648451354 | 3.053967046 | -7.392079712 | 2.70E-13 | 8.49E-13 | 18.64866091 |
| ENC1 | 0.586368739 | 3.650229644 | 7.37504389 | 3.05E-13 | 9.58E-13 | 18.5283508 |
| COLEC12 | -0.758131641 | 2.440220791 | -7.372925584 | 3.10E-13 | 9.72E-13 | 18.51340865 |
| FBXO27 | -0.635360431 | 1.884383288 | -7.370062572 | 3.16E-13 | 9.91E-13 | 18.49321972 |
| SEMA4B | 0.59801568 | 3.91276689 | 7.365792423 | 3.26E-13 | 1.02E-12 | 18.46312147 |
| MPEG1 | -0.724065203 | 3.005171261 | -7.363342666 | 3.32E-13 | 1.04E-12 | 18.44586149 |
| NMU | 0.734105871 | 0.869980798 | 7.36291936 | 3.33E-13 | 1.04E-12 | 18.44287958 |
| AKR7A3 | 1.297269637 | 2.839416194 | 7.36174052 | 3.36E-13 | 1.05E-12 | 18.43457626 |
| MT1E | -1.046759003 | 4.286247899 | -7.337947071 | 3.98E-13 | 1.24E-12 | 18.26724372 |
| CD24 | 1.274422292 | 7.580435413 | 7.334876086 | 4.07E-13 | 1.27E-12 | 18.24568244 |
| HS6ST3 | 0.709627091 | 0.933395227 | 7.332704647 | 4.13E-13 | 1.29E-12 | 18.23044182 |
| FLNA | -0.66025704 | 6.296640191 | -7.311412597 | 4.81E-13 | 1.49E-12 | 18.08121854 |
| ABCG1 | 0.588675653 | 3.074797097 | 7.309562236 | 4.87E-13 | 1.51E-12 | 18.0682692 |
| DCLK1 | -0.780896464 | 1.63139029 | -7.308265756 | 4.92E-13 | 1.53E-12 | 18.05919786 |
| TGFA | -0.644952809 | 1.400501878 | -7.292052035 | 5.52E-13 | 1.71E-12 | 17.94587651 |
| C19orf33 | 1.41875508 | 3.544842447 | 7.275118369 | 6.23E-13 | 1.92E-12 | 17.82776926 |
| ZNF703 | 1.090446833 | 5.140061157 | 7.275091432 | 6.23E-13 | 1.92E-12 | 17.82758158 |
| FABP7 | -1.234323493 | 1.010230288 | -7.27238378 | 6.35E-13 | 1.96E-12 | 17.80871989 |
| MYO5B | 0.591499458 | 3.108330189 | 7.271996849 | 6.36E-13 | 1.96E-12 | 17.80602503 |
| HES4 | 0.742743896 | 2.48857734 | 7.266073156 | 6.64E-13 | 2.05E-12 | 17.76478462 |
| BMPR1B | 1.758119818 | 2.700362284 | 7.263416342 | 6.76E-13 | 2.08E-12 | 17.74629803 |
| HOXB13 | 0.781366928 | 0.750084336 | 7.256158207 | 7.12E-13 | 2.19E-12 | 17.69582618 |
| CSTA | -0.963745284 | 2.421968743 | -7.237102064 | 8.15E-13 | 2.50E-12 | 17.56353303 |
| SYT7 | 0.796909506 | 3.837834346 | 7.23388474 | 8.33E-13 | 2.56E-12 | 17.54122891 |
| FKBP2 | 0.683665762 | 4.779860308 | 7.219601926 | 9.22E-13 | 2.82E-12 | 17.44232297 |
| FAM84B | 0.633555756 | 4.550550486 | 7.215618076 | 9.48E-13 | 2.90E-12 | 17.41476749 |
| NUPR2 | 0.971239334 | 2.219533356 | 7.187905697 | 1.15E-12 | 3.50E-12 | 17.2234726 |
| LYPD3 | 0.851063596 | 3.897060745 | 7.185276657 | 1.17E-12 | 3.57E-12 | 17.20535976 |
| RCOR2 | 0.68445121 | 1.183836431 | 7.184643723 | 1.18E-12 | 3.58E-12 | 17.20100005 |
| DNAJC22 | 0.742780309 | 1.822516245 | 7.180391694 | 1.21E-12 | 3.69E-12 | 17.17172082 |
| MT-ND4L | -0.75306946 | 10.43486193 | -7.171801022 | 1.29E-12 | 3.91E-12 | 17.11261448 |
| HSD11B2 | 0.656660919 | 2.25016335 | 7.160852623 | 1.39E-12 | 4.22E-12 | 17.03738042 |
| PHYHD1 | -0.998127935 | 2.63687338 | -7.149747037 | 1.51E-12 | 4.55E-12 | 16.96117404 |
| RCN3 | 0.74586218 | 4.183293101 | 7.13678824 | 1.65E-12 | 4.97E-12 | 16.87238829 |
| TMEM205 | 0.602709462 | 5.42295318 | 7.136736476 | 1.65E-12 | 4.97E-12 | 16.87203394 |
| MAB21L4 | 1.084579082 | 1.516894136 | 7.129788271 | 1.73E-12 | 5.21E-12 | 16.82449034 |
| LTB | 0.960080821 | 2.033845648 | 7.128423052 | 1.75E-12 | 5.26E-12 | 16.81515374 |
| IFI16 | -0.62231236 | 3.935440418 | -7.122916225 | 1.81E-12 | 5.46E-12 | 16.77750973 |
| MEIS3 | 0.619178508 | 2.317542075 | 7.117817612 | 1.88E-12 | 5.65E-12 | 16.74268006 |
| KRTCAP3 | 0.830583463 | 3.837467246 | 7.116343141 | 1.90E-12 | 5.71E-12 | 16.73261192 |
| FAM241A | -0.623857061 | 2.337102211 | -7.1085234 | 2.01E-12 | 6.02E-12 | 16.67924839 |
| MT-ND4 | -0.62799949 | 12.2583009 | -7.105916438 | 2.04E-12 | 6.13E-12 | 16.66146992 |
| LAPTM5 | 0.63333566 | 5.792280797 | 7.097146527 | 2.17E-12 | 6.50E-12 | 16.60170656 |
| LGALS9 | 0.63482375 | 3.191229221 | 7.092975873 | 2.23E-12 | 6.69E-12 | 16.57330907 |
| ACP5 | 0.754513423 | 4.380430028 | 7.079060096 | 2.46E-12 | 7.37E-12 | 16.47866926 |
| ASS1 | -1.067373459 | 4.559582586 | -7.073602567 | 2.56E-12 | 7.64E-12 | 16.44159983 |
| CXXC5 | 0.698391834 | 4.610249018 | 7.071924236 | 2.59E-12 | 7.72E-12 | 16.43020531 |
| NPY5R | -0.622530814 | 0.510218746 | -7.071272519 | 2.60E-12 | 7.75E-12 | 16.42578135 |
| LAG3 | 0.62322993 | 1.158679981 | 7.069613962 | 2.63E-12 | 7.84E-12 | 16.41452448 |
| ANO1 | -0.89801892 | 4.310175476 | -7.056286161 | 2.88E-12 | 8.58E-12 | 16.32415495 |
| APLP1 | 0.81576326 | 1.508714687 | 7.055096238 | 2.90E-12 | 8.65E-12 | 16.31609427 |
| LIF | -0.635268969 | 1.417584707 | -7.039193765 | 3.24E-12 | 9.63E-12 | 16.20848923 |
| HMOX1 | 0.66106053 | 4.101640159 | 7.039190221 | 3.24E-12 | 9.63E-12 | 16.20846527 |
| UGT8 | -0.737871814 | 0.828938658 | -7.032064144 | 3.40E-12 | 1.01E-11 | 16.16031881 |
| QPRT | 0.824812892 | 2.505044718 | 7.015530743 | 3.81E-12 | 1.13E-11 | 16.04878591 |
| CXCL1 | -0.711749172 | 0.889798146 | -7.009901092 | 3.97E-12 | 1.17E-11 | 16.01086397 |
| RGS16 | 0.668542008 | 3.550021435 | 7.00638207 | 4.06E-12 | 1.20E-11 | 15.98717371 |
| MAST4 | -0.613474293 | 2.260688086 | -7.001376174 | 4.20E-12 | 1.24E-11 | 15.95349261 |
| KAZALD1 | 0.715959585 | 2.00531479 | 6.9999127 | 4.25E-12 | 1.25E-11 | 15.94365013 |
| F2R | 0.647267927 | 4.023090507 | 6.994853513 | 4.40E-12 | 1.30E-11 | 15.90963957 |
| LAPTM4B | 0.816592622 | 5.598050605 | 6.993208698 | 4.45E-12 | 1.31E-11 | 15.89858712 |
| CERS4 | 0.700922693 | 4.146179162 | 6.992842993 | 4.46E-12 | 1.32E-11 | 15.89613007 |
| CGNL1 | -0.685920509 | 2.460993327 | -6.988044661 | 4.61E-12 | 1.36E-11 | 15.8639026 |
| FERMT1 | -0.585629436 | 0.759887418 | -6.982293794 | 4.79E-12 | 1.41E-11 | 15.82530441 |
| CMPK2 | 0.682213899 | 2.028621875 | 6.977288685 | 4.96E-12 | 1.46E-11 | 15.79173539 |
| KRT6B | -1.446341538 | 2.456945919 | -6.976410511 | 4.99E-12 | 1.47E-11 | 15.7858478 |
| STMND1 | 0.877211859 | 1.563447344 | 6.975904214 | 5.01E-12 | 1.47E-11 | 15.78245372 |
| CORO1A | 0.718154006 | 3.113339152 | 6.965783096 | 5.36E-12 | 1.58E-11 | 15.71465212 |
| MT1A | -0.887660967 | 1.476895885 | -6.964496724 | 5.41E-12 | 1.59E-11 | 15.70604119 |
| CST4 | 1.068071275 | 0.996814506 | 6.963322729 | 5.45E-12 | 1.60E-11 | 15.69818378 |
| NELL2 | 1.079925387 | 1.863864778 | 6.961093148 | 5.54E-12 | 1.63E-11 | 15.68326483 |
| SYCP2 | 0.774538195 | 2.384144074 | 6.959319848 | 5.61E-12 | 1.64E-11 | 15.67140218 |
| SLC2A3 | -0.58504628 | 2.449885814 | -6.954223084 | 5.80E-12 | 1.70E-11 | 15.63732242 |
| STOM | -0.607507769 | 6.286578936 | -6.944936072 | 6.18E-12 | 1.81E-11 | 15.5752836 |
| ERP27 | 0.908151108 | 2.604507627 | 6.944761366 | 6.19E-12 | 1.81E-11 | 15.57411727 |
| CAPSL | 0.590156878 | 0.641818149 | 6.938247218 | 6.47E-12 | 1.89E-11 | 15.53064832 |
| SCCPDH | 0.706063176 | 5.166091851 | 6.937259581 | 6.51E-12 | 1.90E-11 | 15.52406109 |
| ARMCX1 | -0.63920258 | 3.778229796 | -6.934025861 | 6.66E-12 | 1.94E-11 | 15.50249928 |
| KCNK2 | -0.806308344 | 1.306202767 | -6.933271652 | 6.69E-12 | 1.95E-11 | 15.49747169 |
| CSN2 | -0.609260323 | 0.141516272 | -6.928516251 | 6.91E-12 | 2.02E-11 | 15.46578365 |
| SEZ6L2 | 1.008933847 | 3.675197161 | 6.915892939 | 7.53E-12 | 2.19E-11 | 15.38176442 |
| SNORC | -0.647738951 | 0.816525753 | -6.912801867 | 7.69E-12 | 2.24E-11 | 15.36121218 |
| MXRA5 | 0.799673061 | 4.866769596 | 6.90835007 | 7.93E-12 | 2.31E-11 | 15.33162754 |
| PAK1 | 0.598310694 | 3.710492794 | 6.893511746 | 8.77E-12 | 2.54E-11 | 15.23314581 |
| HSPB8 | -1.146511696 | 3.376167379 | -6.892305587 | 8.84E-12 | 2.56E-11 | 15.22514915 |
| SPON1 | -0.832468074 | 3.223732654 | -6.891792902 | 8.87E-12 | 2.57E-11 | 15.22175051 |
| SDC2 | -0.725622602 | 4.38848871 | -6.868196737 | 1.04E-11 | 3.01E-11 | 15.06558219 |
| MT-ATP8 | -0.754304615 | 10.70510343 | -6.862372349 | 1.08E-11 | 3.13E-11 | 15.02711038 |
| PYDC1 | 1.179297273 | 1.363348302 | 6.854975083 | 1.14E-11 | 3.28E-11 | 14.97829275 |
| PIM2 | 0.619717832 | 3.247116574 | 6.840823059 | 1.25E-11 | 3.60E-11 | 14.88503336 |
| MRPS30 | 0.864412512 | 3.761793596 | 6.825423964 | 1.39E-11 | 3.99E-11 | 14.78375854 |
| SUSD3 | 1.319350506 | 4.407951272 | 6.824803852 | 1.39E-11 | 4.00E-11 | 14.77968469 |
| NR4A2 | -0.776096505 | 2.623554894 | -6.823391724 | 1.41E-11 | 4.04E-11 | 14.77040894 |
| HOXC12 | 0.745433086 | 0.816657408 | 6.823225233 | 1.41E-11 | 4.04E-11 | 14.76931544 |
| FRZB | -0.771407278 | 2.590088321 | -6.822563068 | 1.41E-11 | 4.06E-11 | 14.76496663 |
| ITGB8 | -0.664009615 | 1.531166033 | -6.820531023 | 1.43E-11 | 4.12E-11 | 14.75162348 |
| FOXJ1 | 0.825871437 | 0.905365955 | 6.81982713 | 1.44E-11 | 4.13E-11 | 14.74700232 |
| KCNE4 | 1.187501042 | 2.993567923 | 6.810847223 | 1.53E-11 | 4.39E-11 | 14.68808668 |
| DHCR24 | 0.737305928 | 7.112769316 | 6.808454175 | 1.55E-11 | 4.46E-11 | 14.67239842 |
| CDK6 | -0.601526822 | 1.695358487 | -6.791486584 | 1.74E-11 | 4.97E-11 | 14.56130932 |
| ACOT4 | 0.585381161 | 2.493433881 | 6.788025246 | 1.78E-11 | 5.08E-11 | 14.538679 |
| CPVL | -0.588725906 | 2.705063138 | -6.787096767 | 1.79E-11 | 5.11E-11 | 14.5326104 |
| ABCA3 | 0.664462823 | 3.593691648 | 6.761501558 | 2.13E-11 | 6.04E-11 | 14.36562113 |
| CEBPD | -0.763892092 | 3.915630448 | -6.756416649 | 2.20E-11 | 6.24E-11 | 14.33251558 |
| SULF2 | 0.728891707 | 4.976913754 | 6.74669015 | 2.34E-11 | 6.65E-11 | 14.26925504 |
| LTF | -1.977700546 | 5.16833155 | -6.746596271 | 2.35E-11 | 6.65E-11 | 14.26864487 |
| RTN1 | -0.712333091 | 1.693083249 | -6.742523295 | 2.41E-11 | 6.83E-11 | 14.24217992 |
| FDCSP | -1.819428366 | 2.635892646 | -6.741829791 | 2.42E-11 | 6.86E-11 | 14.23767522 |
| BTG2 | -0.8642766 | 6.169161354 | -6.741493561 | 2.43E-11 | 6.87E-11 | 14.23549137 |
| S100A16 | 0.794295369 | 6.677602138 | 6.733814459 | 2.55E-11 | 7.22E-11 | 14.1856423 |
| SMARCA1 | -0.686990147 | 3.65941768 | -6.727332299 | 2.67E-11 | 7.53E-11 | 14.14360421 |
| IL20 | 0.899546603 | 1.150579226 | 6.724638885 | 2.71E-11 | 7.66E-11 | 14.12614793 |
| FPR3 | 0.734161706 | 3.178470579 | 6.718287336 | 2.83E-11 | 7.99E-11 | 14.08500859 |
| CXCR3 | 0.652633713 | 1.434531902 | 6.71521873 | 2.89E-11 | 8.15E-11 | 14.06514598 |
| CACNG1 | 0.612240668 | 0.847922689 | 6.69358588 | 3.33E-11 | 9.37E-11 | 13.92535878 |
| CNFN | 0.686816015 | 2.668570907 | 6.689739358 | 3.42E-11 | 9.60E-11 | 13.90054715 |
| GPNMB | -0.677857096 | 5.18408266 | -6.684132228 | 3.55E-11 | 9.95E-11 | 13.86440259 |
| HIST1H1D | 0.613722125 | 0.781760242 | 6.678893719 | 3.67E-11 | 1.03E-10 | 13.83065965 |
| CDH2 | 0.629703699 | 1.039118164 | 6.656939914 | 4.24E-11 | 1.18E-10 | 13.68951528 |
| RASGRP1 | 0.643662016 | 1.630358969 | 6.64383402 | 4.62E-11 | 1.29E-10 | 13.60546124 |
| RARRES1 | -1.147427452 | 3.306889157 | -6.632480582 | 4.98E-11 | 1.38E-10 | 13.53277093 |
| CHRM1 | -0.596141641 | 0.812580631 | -6.627153028 | 5.15E-11 | 1.43E-10 | 13.49870112 |
| CALML3 | -0.91467398 | 1.310875546 | -6.602605174 | 6.05E-11 | 1.68E-10 | 13.342046 |
| CA11 | -0.677296123 | 2.045751898 | -6.602551624 | 6.05E-11 | 1.68E-10 | 13.34170486 |
| PRAME | 1.234953047 | 1.328434745 | 6.598643459 | 6.20E-11 | 1.72E-10 | 13.3168145 |
| MX1 | 0.957670242 | 3.793153641 | 6.595638485 | 6.33E-11 | 1.75E-10 | 13.29768571 |
| B3GNT3 | -0.788865221 | 1.377493333 | -6.591850138 | 6.49E-11 | 1.79E-10 | 13.27358175 |
| KCNF1 | 0.865964143 | 1.251815624 | 6.583483036 | 6.85E-11 | 1.89E-10 | 13.22039037 |
| TSC22D3 | -0.589275842 | 5.066431189 | -6.579932805 | 7.01E-11 | 1.93E-10 | 13.19783982 |
| GLRA3 | -0.652688699 | 0.495984292 | -6.56173926 | 7.88E-11 | 2.17E-10 | 13.08245474 |
| TM4SF1 | -0.9215109 | 5.785635451 | -6.553256618 | 8.33E-11 | 2.29E-10 | 13.02875871 |
| SMOC2 | -0.804675919 | 4.283848609 | -6.546122922 | 8.72E-11 | 2.40E-10 | 12.98365171 |
| DIO1 | 1.020076013 | 1.404566233 | 6.540965848 | 9.02E-11 | 2.47E-10 | 12.95107157 |
| NNMT | -0.680506946 | 4.742049597 | -6.539375762 | 9.11E-11 | 2.50E-10 | 12.94103091 |
| DPYSL3 | -0.644712945 | 4.391026641 | -6.517381226 | 1.05E-10 | 2.87E-10 | 12.80237898 |
| MT-ATP6 | -0.586692183 | 11.84996022 | -6.511556001 | 1.09E-10 | 2.98E-10 | 12.76573009 |
| NUCB2 | 0.585693331 | 3.986192195 | 6.501386586 | 1.16E-10 | 3.18E-10 | 12.7018233 |
| SELE | -0.649152003 | 1.333148633 | -6.496868065 | 1.20E-10 | 3.27E-10 | 12.67345782 |
| CSN3 | -0.908366976 | 0.41366939 | -6.489455186 | 1.26E-10 | 3.42E-10 | 12.62696252 |
| ARFGEF3 | 0.669958015 | 3.152283881 | 6.486559588 | 1.28E-10 | 3.49E-10 | 12.60881409 |
| LRRC6 | 0.587489119 | 1.909520204 | 6.481993997 | 1.32E-10 | 3.59E-10 | 12.58021418 |
| PHLDA3 | -0.59160544 | 3.835022315 | -6.480775238 | 1.33E-10 | 3.62E-10 | 12.57258277 |
| LRIG1 | -0.600399405 | 4.488658184 | -6.479029304 | 1.34E-10 | 3.65E-10 | 12.56165272 |
| VSTM2L | 0.758179636 | 1.201385139 | 6.47269547 | 1.40E-10 | 3.80E-10 | 12.52202413 |
| GSTP1 | -0.911388898 | 6.012346759 | -6.460661102 | 1.51E-10 | 4.10E-10 | 12.44682891 |
| MYB | 0.866136859 | 3.638280239 | 6.450133162 | 1.62E-10 | 4.38E-10 | 12.38115346 |
| GPT | -0.622525133 | 1.314817518 | -6.4366196 | 1.76E-10 | 4.76E-10 | 12.29699957 |
| FCMR | 0.64924981 | 2.508499615 | 6.434952577 | 1.78E-10 | 4.81E-10 | 12.28662983 |
| CLIC3 | 0.721818346 | 1.830900618 | 6.431474638 | 1.82E-10 | 4.91E-10 | 12.26500332 |
| CCNO | 0.994481629 | 2.090036079 | 6.429237705 | 1.85E-10 | 4.98E-10 | 12.25109939 |
| ITGB4 | -0.774421517 | 4.195528842 | -6.427601058 | 1.86E-10 | 5.03E-10 | 12.24092947 |
| NOSTRIN | -0.670415343 | 2.349138077 | -6.421579414 | 1.94E-10 | 5.22E-10 | 12.20353256 |
| PCP2 | 0.761312135 | 1.82067503 | 6.415062563 | 2.02E-10 | 5.44E-10 | 12.16309707 |
| AGTR1 | -1.111228122 | 1.724223149 | -6.414736191 | 2.02E-10 | 5.45E-10 | 12.16107302 |
| NTS | -0.723900608 | 0.431519565 | -6.399181724 | 2.23E-10 | 6.00E-10 | 12.06472087 |
| HEPACAM2 | -0.683070439 | 0.930849704 | -6.395791533 | 2.28E-10 | 6.13E-10 | 12.04374934 |
| TGFB3 | 0.630157626 | 3.786863972 | 6.37344266 | 2.63E-10 | 7.04E-10 | 11.90576027 |
| STEAP2 | -0.669092332 | 2.040847359 | -6.371112472 | 2.67E-10 | 7.13E-10 | 11.89139893 |
| MLPH | 1.119269994 | 4.771320911 | 6.364544895 | 2.78E-10 | 7.43E-10 | 11.85094827 |
| PIP | -2.233841917 | 5.500633014 | -6.351239905 | 3.02E-10 | 8.07E-10 | 11.76912045 |
| NXPH4 | 0.729456444 | 1.293267903 | 6.347136435 | 3.10E-10 | 8.27E-10 | 11.74391576 |
| CACNA1H | 0.852378552 | 1.639661198 | 6.321202671 | 3.65E-10 | 9.70E-10 | 11.58497556 |
| THBS2 | 0.82492928 | 5.364418798 | 6.316464203 | 3.76E-10 | 9.98E-10 | 11.5560007 |
| MPZL2 | -0.603797849 | 2.693209768 | -6.311693652 | 3.87E-10 | 1.03E-09 | 11.5268502 |
| UGCG | 0.73293767 | 4.643718017 | 6.309637277 | 3.92E-10 | 1.04E-09 | 11.51429106 |
| AGR2 | 1.675904249 | 6.086330186 | 6.292882679 | 4.36E-10 | 1.15E-09 | 11.41210647 |
| BPIFB1 | 1.10337695 | 1.871003989 | 6.292457474 | 4.37E-10 | 1.16E-09 | 11.4095165 |
| GLIPR2 | -0.606919788 | 3.410615567 | -6.283398634 | 4.62E-10 | 1.22E-09 | 11.35437699 |
| UNC5A | 0.634066986 | 1.067202458 | 6.27891173 | 4.75E-10 | 1.25E-09 | 11.32709358 |
| DNAJC12 | 1.112818748 | 3.40911914 | 6.267931902 | 5.09E-10 | 1.34E-09 | 11.26040575 |
| KLK10 | -0.912845209 | 1.237842889 | -6.260446498 | 5.33E-10 | 1.40E-09 | 11.21500458 |
| FADS2 | 0.999479216 | 3.153777903 | 6.251434363 | 5.64E-10 | 1.48E-09 | 11.16041077 |
| GPT2 | -0.598911929 | 2.809000275 | -6.246761053 | 5.80E-10 | 1.52E-09 | 11.13212975 |
| SYT17 | 0.605947341 | 1.667476898 | 6.225991172 | 6.60E-10 | 1.73E-09 | 11.00667851 |
| TMPRSS6 | 0.675320428 | 1.070069387 | 6.222329629 | 6.75E-10 | 1.77E-09 | 10.9846032 |
| SKAP1 | 0.686270487 | 2.420538619 | 6.218528894 | 6.91E-10 | 1.81E-09 | 10.96170157 |
| PEG3 | -0.849165542 | 1.493271477 | -6.193059762 | 8.08E-10 | 2.11E-09 | 10.80857401 |
| CHPT1 | -0.595415393 | 3.673805359 | -6.183174299 | 8.59E-10 | 2.24E-09 | 10.74929866 |
| CD14 | -0.585134812 | 4.910519473 | -6.178736595 | 8.83E-10 | 2.30E-09 | 10.72271813 |
| BMP4 | -0.686508303 | 2.065749518 | -6.165043017 | 9.60E-10 | 2.49E-09 | 10.64081057 |
| BST2 | 0.878613338 | 7.006394858 | 6.160178179 | 9.89E-10 | 2.56E-09 | 10.61175281 |
| TCN1 | -1.221585991 | 1.77589058 | -6.150027199 | 1.05E-09 | 2.72E-09 | 10.5511902 |
| TUBA3D | 0.806732964 | 1.242575438 | 6.149755876 | 1.05E-09 | 2.73E-09 | 10.54957272 |
| UGT2B28 | -0.6684592 | 0.421310639 | -6.148358756 | 1.06E-09 | 2.75E-09 | 10.54124493 |
| ECM1 | 0.962482229 | 4.210326683 | 6.120248187 | 1.26E-09 | 3.25E-09 | 10.37406437 |
| KCNJ3 | 1.236106791 | 1.480968729 | 6.085713368 | 1.56E-09 | 3.99E-09 | 10.16966273 |
| LAMP3 | 0.704351346 | 1.587881409 | 6.048525324 | 1.95E-09 | 4.97E-09 | 9.950773673 |
| CYBA | 0.640967095 | 3.959160833 | 6.023098487 | 2.27E-09 | 5.76E-09 | 9.801837855 |
| HRASLS2 | 0.759297598 | 2.088566823 | 6.004285888 | 2.54E-09 | 6.43E-09 | 9.692024632 |
| HLA-DQB1 | 0.726930616 | 4.356124173 | 6.00377484 | 2.55E-09 | 6.44E-09 | 9.689046043 |
| JUNB | -0.651538986 | 6.055883749 | -5.994540958 | 2.69E-09 | 6.79E-09 | 9.635268553 |
| GREM1 | 0.592594457 | 1.837658225 | 5.980441947 | 2.93E-09 | 7.37E-09 | 9.553307309 |
| KLRG2 | 0.644855526 | 1.439516984 | 5.969033027 | 3.14E-09 | 7.87E-09 | 9.487117383 |
| TOX3 | 0.746626142 | 2.147757477 | 5.963143236 | 3.25E-09 | 8.14E-09 | 9.452993817 |
| GRB7 | 0.743909809 | 3.36671248 | 5.958402861 | 3.34E-09 | 8.37E-09 | 9.425552669 |
| FST | -0.690128752 | 2.450833994 | -5.956035652 | 3.39E-09 | 8.48E-09 | 9.411857033 |
| INSYN2 | 0.772931041 | 0.979528027 | 5.953175295 | 3.45E-09 | 8.62E-09 | 9.395315107 |
| TFCP2L1 | -0.763417291 | 1.87454057 | -5.937823878 | 3.77E-09 | 9.42E-09 | 9.306663283 |
| SELL | 0.711410099 | 2.320398341 | 5.918093042 | 4.24E-09 | 1.06E-08 | 9.19303809 |
| CXCL13 | 1.292191149 | 2.674812703 | 5.914369565 | 4.34E-09 | 1.08E-08 | 9.171635489 |
| CRIP2 | 0.60825008 | 5.031040739 | 5.902007354 | 4.66E-09 | 1.16E-08 | 9.100668453 |
| ITGB2 | 0.588721057 | 3.5372027 | 5.86953035 | 5.65E-09 | 1.39E-08 | 8.914897363 |
| H2AFJ | 0.70190246 | 4.850860627 | 5.864227621 | 5.82E-09 | 1.44E-08 | 8.884657238 |
| VGLL1 | -0.978503614 | 1.185411621 | -5.861391585 | 5.92E-09 | 1.46E-08 | 8.868494637 |
| RARRES3 | 0.874422636 | 5.899549117 | 5.832892094 | 6.99E-09 | 1.72E-08 | 8.706485708 |
| S100A6 | -0.707890438 | 8.198347628 | -5.829625212 | 7.13E-09 | 1.75E-08 | 8.687962374 |
| FAM83E | 0.801521861 | 1.8969784 | 5.807704514 | 8.10E-09 | 1.98E-08 | 8.563924955 |
| CLCA2 | -0.960576507 | 1.269039713 | -5.778830478 | 9.57E-09 | 2.34E-08 | 8.401216656 |
| CCL8 | -0.65434879 | 1.74836826 | -5.775421081 | 9.76E-09 | 2.38E-08 | 8.382054967 |
| SLC38A1 | 0.666916587 | 4.943888172 | 5.764061904 | 1.04E-08 | 2.54E-08 | 8.318290675 |
| CCR7 | 0.610547705 | 1.47475018 | 5.763469428 | 1.05E-08 | 2.55E-08 | 8.31496809 |
| IFI27 | 0.945096172 | 4.983052342 | 5.732644508 | 1.25E-08 | 3.03E-08 | 8.142549091 |
| LRG1 | 1.03802831 | 2.995503151 | 5.732028658 | 1.25E-08 | 3.04E-08 | 8.139113253 |
| CYP4F22 | -0.836653465 | 1.461850955 | -5.72192292 | 1.33E-08 | 3.21E-08 | 8.082783137 |
| PLAT | -0.831942184 | 3.958731266 | -5.704089405 | 1.47E-08 | 3.55E-08 | 7.983607474 |
| TENT5B | -0.667075138 | 2.799962371 | -5.683696363 | 1.65E-08 | 3.98E-08 | 7.870557232 |
| IQGAP2 | -0.633049915 | 2.765050181 | -5.666220497 | 1.83E-08 | 4.39E-08 | 7.773983876 |
| PGAP3 | 0.625344404 | 3.942162259 | 5.653862541 | 1.96E-08 | 4.70E-08 | 7.705862764 |
| CA9 | 0.789567038 | 0.911987672 | 5.616259184 | 2.42E-08 | 5.78E-08 | 7.499448392 |
| FOXI1 | -0.885343098 | 1.811177235 | -5.610038496 | 2.51E-08 | 5.98E-08 | 7.465427429 |
| PVALB | 1.097428106 | 1.405263222 | 5.607488313 | 2.54E-08 | 6.06E-08 | 7.451490814 |
| RHOBTB3 | -0.615936504 | 3.057090031 | -5.569229748 | 3.15E-08 | 7.48E-08 | 7.243131816 |
| RASEF | 0.60187302 | 3.23283885 | 5.559425374 | 3.33E-08 | 7.89E-08 | 7.189954507 |
| ORM1 | 0.829261123 | 0.915432054 | 5.553744161 | 3.44E-08 | 8.14E-08 | 7.159181267 |
| AQP5 | -0.995650756 | 1.426059232 | -5.552979034 | 3.45E-08 | 8.17E-08 | 7.155039113 |
| TMEM139 | -0.681769606 | 1.906704291 | -5.546793116 | 3.57E-08 | 8.44E-08 | 7.121570434 |
| PHGDH | -0.774656196 | 3.292817328 | -5.542058299 | 3.67E-08 | 8.66E-08 | 7.095976828 |
| SDR16C5 | 0.792788274 | 1.881321486 | 5.538807266 | 3.74E-08 | 8.81E-08 | 7.078415701 |
| TSPAN8 | -0.655064759 | 0.756211242 | -5.532415949 | 3.87E-08 | 9.12E-08 | 7.043920229 |
| LYZ | 0.916163176 | 5.406693961 | 5.518128421 | 4.19E-08 | 9.86E-08 | 6.9669439 |
| PREX1 | 0.716706952 | 4.423661659 | 5.472654975 | 5.39E-08 | 1.26E-07 | 6.723208359 |
| IFITM1 | 0.712275263 | 6.935546125 | 5.471505249 | 5.42E-08 | 1.27E-07 | 6.71707075 |
| FBP1 | 0.777073607 | 5.139966925 | 5.464841205 | 5.63E-08 | 1.31E-07 | 6.681520075 |
| BARX2 | -0.731308528 | 2.517427668 | -5.456020355 | 5.91E-08 | 1.38E-07 | 6.634526886 |
| REPS2 | 0.631683978 | 2.335584808 | 5.453153961 | 6.00E-08 | 1.40E-07 | 6.61927169 |
| MMP7 | -1.098569601 | 3.695183436 | -5.449609072 | 6.12E-08 | 1.42E-07 | 6.600416037 |
| GPC4 | 0.620451785 | 3.327810242 | 5.429742621 | 6.82E-08 | 1.58E-07 | 6.4949602 |
| KLHDC7B | 0.757701748 | 1.602614843 | 5.424308147 | 7.03E-08 | 1.63E-07 | 6.466176597 |
| LAD1 | 0.802967797 | 3.76102252 | 5.421531668 | 7.14E-08 | 1.65E-07 | 6.45148161 |
| SPDYC | 0.771372 | 0.755083555 | 5.381702593 | 8.86E-08 | 2.04E-07 | 6.241468318 |
| LY6E | 0.671203893 | 6.599216564 | 5.3814088 | 8.88E-08 | 2.04E-07 | 6.239924672 |
| FAM234B | 0.74503271 | 3.219986732 | 5.376467167 | 9.12E-08 | 2.10E-07 | 6.213972352 |
| BCL2A1 | 0.594129039 | 1.787075207 | 5.363064109 | 9.80E-08 | 2.25E-07 | 6.143696974 |
| CCDC74A | 0.814865078 | 2.839531915 | 5.353360547 | 1.03E-07 | 2.37E-07 | 6.092923277 |
| SFN | 0.697649764 | 4.922616726 | 5.341825814 | 1.10E-07 | 2.52E-07 | 6.032682059 |
| SOX11 | 0.585330169 | 0.827944209 | 5.331514622 | 1.16E-07 | 2.65E-07 | 5.978935799 |
| ARMT1 | 0.732846428 | 4.743835443 | 5.323626328 | 1.21E-07 | 2.77E-07 | 5.937885572 |
| PLEKHS1 | -0.731154282 | 2.507364048 | -5.313194984 | 1.28E-07 | 2.92E-07 | 5.883690493 |
| FCRLB | 0.588902331 | 0.926261788 | 5.311403866 | 1.29E-07 | 2.95E-07 | 5.874395106 |
| CA12 | 1.044944834 | 5.017468298 | 5.296944956 | 1.40E-07 | 3.18E-07 | 5.799467052 |
| IL32 | 0.59947507 | 3.36998426 | 5.294919435 | 1.41E-07 | 3.22E-07 | 5.788986092 |
| EHF | -0.894451738 | 3.818540213 | -5.289056635 | 1.46E-07 | 3.31E-07 | 5.758670877 |
| ACTL8 | 0.622085527 | 0.581174821 | 5.254855448 | 1.75E-07 | 3.95E-07 | 5.582463408 |
| TMEM64 | -0.589140857 | 2.363187971 | -5.246026996 | 1.84E-07 | 4.14E-07 | 5.537155732 |
| COL6A3 | 0.648541968 | 5.591800327 | 5.239266135 | 1.90E-07 | 4.28E-07 | 5.502508151 |
| SLC39A6 | 0.923132944 | 7.32810425 | 5.237562585 | 1.92E-07 | 4.32E-07 | 5.49378465 |
| BCL2 | -0.641395257 | 3.339499731 | -5.234400772 | 1.95E-07 | 4.39E-07 | 5.477600897 |
| FGFR4 | 0.649159107 | 1.544527555 | 5.226483794 | 2.04E-07 | 4.57E-07 | 5.437118768 |
| MUC5B | 0.783231138 | 1.422647516 | 5.215769677 | 2.15E-07 | 4.83E-07 | 5.382427181 |
| HLA-DQA1 | 0.611589157 | 3.980774025 | 5.194171116 | 2.41E-07 | 5.40E-07 | 5.272500668 |
| SCD | -0.823002938 | 6.460422026 | -5.192392025 | 2.44E-07 | 5.45E-07 | 5.26346537 |
| TFF1 | 1.782812333 | 5.424988718 | 5.163066316 | 2.84E-07 | 6.34E-07 | 5.114958289 |
| AGR3 | 1.407154797 | 5.025177561 | 5.154613754 | 2.97E-07 | 6.61E-07 | 5.072303396 |
| PLPPR3 | 0.622967326 | 1.38143423 | 5.109457011 | 3.75E-07 | 8.31E-07 | 4.845558233 |
| TMEM158 | 0.615561265 | 2.016383367 | 5.101542884 | 3.91E-07 | 8.65E-07 | 4.806015808 |
| INSM1 | 0.588555653 | 0.624451278 | 5.093494775 | 4.08E-07 | 9.01E-07 | 4.765864147 |
| CASP14 | 0.875631929 | 0.920648247 | 5.087377282 | 4.21E-07 | 9.29E-07 | 4.735384858 |
| C4A | 0.7552041 | 2.653033462 | 5.075081189 | 4.48E-07 | 9.88E-07 | 4.674227969 |
| GZMK | 0.592537168 | 1.675763191 | 5.062629211 | 4.78E-07 | 1.05E-06 | 4.612440251 |
| HPGD | -0.60856593 | 1.048299283 | -5.054258774 | 4.99E-07 | 1.10E-06 | 4.570987216 |
| FAM3B | -0.725798771 | 1.35907492 | -5.048707622 | 5.13E-07 | 1.13E-06 | 4.543532426 |
| CST5 | 0.868694283 | 0.862419698 | 5.036035989 | 5.48E-07 | 1.20E-06 | 4.480969634 |
| MSMB | 1.051411059 | 2.240150934 | 5.034171132 | 5.53E-07 | 1.21E-06 | 4.471775122 |
| CBLN2 | 0.640678746 | 0.639537017 | 5.032383735 | 5.58E-07 | 1.22E-06 | 4.462965587 |
| KLK11 | -0.777534039 | 1.110871019 | -5.031586073 | 5.60E-07 | 1.23E-06 | 4.459035125 |
| SLC7A8 | 0.658811463 | 4.171230942 | 4.983632547 | 7.15E-07 | 1.56E-06 | 4.223842806 |
| HLA-DQB2 | 0.618223486 | 2.766915516 | 4.980475442 | 7.27E-07 | 1.58E-06 | 4.208434313 |
| CCND1 | 0.710785596 | 6.208133916 | 4.975069451 | 7.47E-07 | 1.62E-06 | 4.182071718 |
| PCSK1N | 0.670240439 | 1.057987544 | 4.962971521 | 7.94E-07 | 1.72E-06 | 4.123175082 |
| PSCA | 0.744035603 | 1.468322852 | 4.952716663 | 8.36E-07 | 1.81E-06 | 4.073358898 |
| C4B | 0.749044937 | 2.769178406 | 4.947300968 | 8.59E-07 | 1.86E-06 | 4.047090379 |
| AARD | 0.873410011 | 3.323999861 | 4.930500571 | 9.35E-07 | 2.02E-06 | 3.965776597 |
| DSP | 0.659570176 | 5.86374508 | 4.911299679 | 1.03E-06 | 2.22E-06 | 3.873169744 |
| MMP12 | 0.65119311 | 1.166111094 | 4.910992757 | 1.03E-06 | 2.22E-06 | 3.871692261 |
| BNIPL | 0.674173679 | 3.145354371 | 4.891049989 | 1.14E-06 | 2.45E-06 | 3.7758806 |
| GP2 | 0.885629515 | 1.830210487 | 4.889298471 | 1.15E-06 | 2.47E-06 | 3.767483623 |
| IRX2 | -0.721296159 | 3.22732861 | -4.881217152 | 1.20E-06 | 2.56E-06 | 3.728778294 |
| APOE | 0.628931145 | 6.43409862 | 4.875896163 | 1.23E-06 | 2.63E-06 | 3.703327112 |
| S100A7 | 1.376410271 | 1.952808581 | 4.844132774 | 1.44E-06 | 3.06E-06 | 3.551952684 |
| HPX | 0.706350828 | 1.898639396 | 4.839908169 | 1.47E-06 | 3.13E-06 | 3.531891208 |
| SYT1 | 0.738222306 | 1.490822628 | 4.83588871 | 1.50E-06 | 3.19E-06 | 3.512819546 |
| SBSPON | -0.650659636 | 1.798213204 | -4.831968573 | 1.53E-06 | 3.25E-06 | 3.494233826 |
| CTGF | -0.615640611 | 6.81787131 | -4.820169147 | 1.62E-06 | 3.44E-06 | 3.438379203 |
| SLC30A8 | 0.825529487 | 0.948535149 | 4.81113998 | 1.69E-06 | 3.58E-06 | 3.395726813 |
| ORM2 | 0.647717785 | 0.844328716 | 4.786414371 | 1.91E-06 | 4.03E-06 | 3.279320798 |
| RAMP3 | -0.616206363 | 3.701249797 | -4.751784988 | 2.26E-06 | 4.75E-06 | 3.117259377 |
| SIX1 | 0.615021583 | 2.061922523 | 4.747210725 | 2.31E-06 | 4.85E-06 | 3.095937098 |
| CLEC3A | 1.371437043 | 1.903536132 | 4.729270525 | 2.52E-06 | 5.28E-06 | 3.012502336 |
| OR2I1P | 0.62906741 | 1.590061969 | 4.716226479 | 2.68E-06 | 5.61E-06 | 2.952029297 |
| ENPP5 | 0.627134055 | 3.172389365 | 4.682352685 | 3.16E-06 | 6.56E-06 | 2.795740068 |
| UGT2B11 | -0.958572941 | 1.204852631 | -4.681231409 | 3.18E-06 | 6.60E-06 | 2.790585221 |
| PADI2 | -0.762111954 | 3.132638651 | -4.644117371 | 3.79E-06 | 7.84E-06 | 2.620632575 |
| CGA | 0.731919763 | 0.698686338 | 4.608786491 | 4.48E-06 | 9.22E-06 | 2.460058067 |
| SCGB1D2 | -1.664803401 | 4.879740405 | -4.597202979 | 4.73E-06 | 9.71E-06 | 2.407670067 |
| VSTM2A | 0.683600851 | 0.740798157 | 4.529098422 | 6.51E-06 | 1.32E-05 | 2.102233256 |
| IL6ST | -0.597906695 | 5.448985116 | -4.459123807 | 9.00E-06 | 1.80E-05 | 1.79299951 |
| SPTSSB | 0.692949307 | 1.979612055 | 4.444969676 | 9.60E-06 | 1.92E-05 | 1.731015503 |
| CHI3L1 | -0.831646221 | 3.565221609 | -4.434748054 | 1.01E-05 | 2.01E-05 | 1.686371386 |
| S100A1 | -0.825076915 | 3.172349737 | -4.375133455 | 1.32E-05 | 2.61E-05 | 1.42798032 |
| CHST8 | 0.586010621 | 1.384225363 | 4.363705008 | 1.39E-05 | 2.74E-05 | 1.378832203 |
| IFIT1 | 0.641942985 | 3.575074332 | 4.357875252 | 1.43E-05 | 2.81E-05 | 1.353809264 |
| HLA-DQA2 | 0.701193301 | 3.450112129 | 4.327155199 | 1.64E-05 | 3.21E-05 | 1.222485727 |
| CDH3 | -0.653655532 | 2.986789254 | -4.32525525 | 1.65E-05 | 3.24E-05 | 1.214393299 |
| CRABP1 | -1.005379134 | 2.307506705 | -4.317985907 | 1.70E-05 | 3.34E-05 | 1.183462898 |
| ALOX15B | -0.830868022 | 2.057755644 | -4.300345671 | 1.84E-05 | 3.61E-05 | 1.108615004 |
| SFRP4 | -0.663732082 | 4.392518368 | -4.299554647 | 1.85E-05 | 3.62E-05 | 1.105265634 |
| BAMBI | 0.695106365 | 4.401199229 | 4.285504826 | 1.97E-05 | 3.84E-05 | 1.045875127 |
| TFF3 | 1.186164027 | 5.662637729 | 4.194981577 | 2.93E-05 | 5.65E-05 | 0.667744411 |
| SLC27A2 | 0.615136042 | 2.042417872 | 4.123987973 | 3.98E-05 | 7.59E-05 | 0.37667827 |
| KRT23 | -0.80561298 | 3.260405192 | -4.104172423 | 4.33E-05 | 8.24E-05 | 0.296298629 |
| PPP1R14C | -0.590528139 | 1.350495985 | -4.096723479 | 4.47E-05 | 8.51E-05 | 0.266180141 |
| GLYATL2 | -0.764390359 | 2.113446356 | -4.050045237 | 5.45E-05 | 0.0001029 | 0.078656657 |
| CYP4B1 | -0.623594088 | 2.185198418 | -3.921512918 | 9.30E-05 | 0.000172538 | -0.426890684 |
| UBD | 0.607376783 | 2.160057539 | 3.907445101 | 9.85E-05 | 0.000182328 | -0.48125818 |
| KIF12 | 0.625015211 | 3.092796945 | 3.877204801 | 0.000111376 | 0.000205187 | -0.59748209 |
| CHI3L2 | -0.637034897 | 2.467451821 | -3.853418206 | 0.00012263 | 0.000225134 | -0.688283357 |
| SFRP2 | 0.597641444 | 7.303673163 | 3.80551234 | 0.000148631 | 0.00027091 | -0.869500917 |
| REEP6 | 0.637153821 | 4.071078247 | 3.803925556 | 0.000149575 | 0.00027253 | -0.87546552 |
| CDH1 | 0.586915767 | 5.863051275 | 3.783719373 | 0.000162106 | 0.000294702 | -0.951206673 |
| AREG | -0.746756798 | 3.313141111 | -3.617738379 | 0.000309511 | 0.000545918 | -1.558450474 |
| CP | -0.668598145 | 2.037662705 | -3.591853707 | 0.000341581 | 0.000600209 | -1.650748766 |
| ESR1 | 0.767987368 | 4.104958144 | 3.568143994 | 0.000373664 | 0.000654523 | -1.734722451 |
| SLC1A1 | 0.587399923 | 2.344202852 | 3.468668599 | 0.000541534 | 0.000932522 | -2.081101184 |
| CLSTN2 | -0.588900385 | 2.900214662 | -3.409685939 | 0.000671928 | 0.001146455 | -2.281948605 |
| SCGB2A2 | -1.307721337 | 5.463535036 | -3.336582408 | 0.000874059 | 0.001474343 | -2.526190511 |
| VTCN1 | -0.700075204 | 4.484329547 | -3.218363007 | 0.001323658 | 0.00219241 | -2.910165641 |
| CRISP3 | 0.61371362 | 1.203552931 | 3.20744413 | 0.001374497 | 0.002272764 | -2.944943455 |
| KRT16 | -0.633153459 | 2.000001163 | -3.075428395 | 0.00214941 | 0.003480191 | -3.356227418 |
| NPY1R | -0.744497676 | 3.158308172 | -2.999963593 | 0.002755686 | 0.004410316 | -3.583688674 |
| DHRS2 | 0.661758629 | 1.917291658 | 2.959594184 | 0.003140762 | 0.005001623 | -3.70308212 |
| CALML5 | 0.631244879 | 3.344421532 | 2.195111528 | 0.028346122 | 0.040262247 | -5.662091718 |

Supplementary Table 3

| AIFM1 |
| --- |
| AKT3 |
| BAX |
| BCL2 |
| CASP10 |
| CASP3 |
| CASP6 |
| CFLAR |
| ENDOD1 |
| FADD |
| FAS |
| IL1R1 |
| IL3RA |
| IRAK1 |
| IRAK3 |
| PIK3CA |
| PIK3R1 |
| PIK3R3 |
| PRKAR2B |
| TNFRSF10D |
| TRAF2 |

Supplementary Table 4

| NAME | GS<br> follow link to MSigDB | GS DETAILS | SIZE | ES | NES | NOM p-val | FDR q-val | FWER p-val | RANK AT MAX | LEADING EDGE |  |  |  |
| --- | --- | --- | --- | --- | --- | --- | --- | --- | --- | --- | --- | --- | --- |
| GO_CELLULAR_RESPONSE_TO_HEAT | GO_CELLULAR_RESPONSE_TO_HEAT | Details ... | 120 | 0.5925117 | 2.304447 | 0 | 0.01081152 | 0.011 | 3440 | tags=49%, list=18%, signal=59% |  |  |  |
| GO_REGULATION_OF_CELLULAR_RESPONSE_TO_HEAT | GO_REGULATION_OF_CELLULAR_RESPONSE_TO_HEAT | Details ... | 78 | 0.6434205 | 2.2814229 | 0 | 0.007658866 | 0.014 | 3430 | tags=56%, list=18%, signal=68% |  |  |  |
| GO_SPINDLE_MICROTUBULE | GO_SPINDLE_MICROTUBULE | Details ... | 66 | 0.666724 | 2.227986 | 0 | 0.018729988 | 0.038 | 4189 | tags=67%, list=21%, signal=85% |  |  |  |
| HP_ABNORMAL_ASTROCYTE_MORPHOLOGY | HP_ABNORMAL_ASTROCYTE_MORPHOLOGY | Details ... | 37 | 0.6503697 | 2.2278986 | 0 | 0.01404749 | 0.038 | 2642 | tags=49%, list=14%, signal=56% |  |  |  |
| GO_MITOTIC_SPINDLE_ASSEMBLY | GO_MITOTIC_SPINDLE_ASSEMBLY | Details ... | 65 | 0.67410225 | 2.1948807 | 0 | 0.018740512 | 0.06 | 3903 | tags=68%, list=20%, signal=84% |  |  |  |
| GO_ENDOPLASMIC_RETICULUM_ORGANIZATION | GO_ENDOPLASMIC_RETICULUM_ORGANIZATION | Details ... | 66 | 0.5696256 | 2.1930485 | 0 | 0.015936313 | 0.062 | 3208 | tags=47%, list=16%, signal=56% |  |  |  |
| GO_POSITIVE_REGULATION_OF_VIRAL_GENOME_REPLICATION | GO_POSITIVE_REGULATION_OF_VIRAL_GENOME_REPLICATION | Details ... | 37 | 0.63886106 | 2.1887107 | 0 | 0.014267694 | 0.066 | 2793 | tags=54%, list=14%, signal=63% |  |  |  |
| GO_TRICARBOXYLIC_ACID_CYCLE | GO_TRICARBOXYLIC_ACID_CYCLE | Details ... | 34 | 0.7001184 | 2.1844854 | 0 | 0.013859231 | 0.069 | 3422 | tags=65%, list=18%, signal=78% |  |  |  |
| GO_MITOTIC_SPINDLE_ORGANIZATION | GO_MITOTIC_SPINDLE_ORGANIZATION | Details ... | 117 | 0.6169791 | 2.1727715 | 0 | 0.014671803 | 0.08 | 3905 | tags=59%, list=20%, signal=73% |  |  |  |
| GO_CHROMOSOMAL_REGION | GO_CHROMOSOMAL_REGION | Details ... | 328 | 0.59061533 | 2.1696136 | 0 | 0.013992449 | 0.085 | 3750 | tags=55%, list=19%, signal=67% |  |  |  |
| GO_NUCLEAR_CHROMOSOME_TELOMERIC_REGION | GO_NUCLEAR_CHROMOSOME_TELOMERIC_REGION | Details ... | 102 | 0.6030397 | 2.1688714 | 0 | 0.012796567 | 0.086 | 3514 | tags=54%, list=18%, signal=65% |  |  |  |
| GO_DNA_GEOMETRIC_CHANGE | GO_DNA_GEOMETRIC_CHANGE | Details ... | 113 | 0.64541465 | 2.1671927 | 0 | 0.012166036 | 0.088 | 3893 | tags=62%, list=20%, signal=77% |  |  |  |
| GO_NUCLEAR_PORE | GO_NUCLEAR_PORE | Details ... | 83 | 0.6159601 | 2.1626844 | 0 | 0.012550245 | 0.098 | 3670 | tags=53%, list=19%, signal=65% |  |  |  |
| GO_MICROTUBULE_CYTOSKELETON_ORGANIZATION_INVOLVED_IN_MITOSIS | GO_MICROTUBULE_CYTOSKELETON_ORGANIZATION_INVOLVED_IN_MITOSIS | Details ... | 143 | 0.59972614 | 2.161568 | 0 | 0.01201426 | 0.102 | 3905 | tags=59%, list=20%, signal=74% |  |  |  |
| GO_SISTER_CHROMATID_COHESION | GO_SISTER_CHROMATID_COHESION | Details ... | 56 | 0.7066748 | 2.1582181 | 0 | 0.011741685 | 0.106 | 3697 | tags=66%, list=19%, signal=81% |  |  |  |
| GO_NEGATIVE_REGULATION_OF_DNA_REPLICATION | GO_NEGATIVE_REGULATION_OF_DNA_REPLICATION | Details ... | 34 | 0.68105525 | 2.1580725 | 0 | 0.01100783 | 0.106 | 2729 | tags=56%, list=14%, signal=65% |  |  |  |
| GO_CHROMOSOME_SEGREGATION | GO_CHROMOSOME_SEGREGATION | Details ... | 320 | 0.5675337 | 2.157792 | 0 | 0.01036031 | 0.106 | 3932 | tags=54%, list=20%, signal=67% |  |  |  |
| GO_REGULATION_OF_SPINDLE_ASSEMBLY | GO_REGULATION_OF_SPINDLE_ASSEMBLY | Details ... | 24 | 0.7187853 | 2.1397488 | 0 | 0.01348291 | 0.135 | 2959 | tags=67%, list=15%, signal=78% |  |  |  |
| GO_VESICLE_TARGETING | GO_VESICLE_TARGETING | Details ... | 91 | 0.56480986 | 2.1350396 | 0 | 0.013737385 | 0.144 | 4044 | tags=53%, list=21%, signal=66% |  |  |  |
| KEGG_UBIQUITIN_MEDIATED_PROTEOLYSIS | KEGG_UBIQUITIN_MEDIATED_PROTEOLYSIS | Details ... | 133 | 0.52324027 | 2.1347375 | 0 | 0.013154572 | 0.145 | 4486 | tags=55%, list=23%, signal=71% |  |  |  |
| GO_PROTEIN_LOCALIZATION_TO_CHROMOSOME_TELOMERIC_REGION | GO_PROTEIN_LOCALIZATION_TO_CHROMOSOME_TELOMERIC_REGION | Details ... | 29 | 0.70313704 | 2.134241 | 0 | 0.012718069 | 0.147 | 3344 | tags=69%, list=17%, signal=83% |  |  |  |
| HP_ABNORMAL_TONGUE_PHYSIOLOGY | HP_ABNORMAL_TONGUE_PHYSIOLOGY | Details ... | 35 | 0.624397 | 2.1319938 | 0 | 0.012570532 | 0.153 | 4315 | tags=49%, list=22%, signal=62% |  |  |  |
| GO_CHROMOSOME_CENTROMERIC_REGION | GO_CHROMOSOME_CENTROMERIC_REGION | Details ... | 192 | 0.6267883 | 2.127194 | 0 | 0.013358995 | 0.167 | 3743 | tags=61%, list=19%, signal=75% |  |  |  |
| GO_SISTER_CHROMATID_SEGREGATION | GO_SISTER_CHROMATID_SEGREGATION | Details ... | 187 | 0.6050675 | 2.123279 | 0 | 0.013784685 | 0.174 | 3905 | tags=59%, list=20%, signal=73% |  |  |  |
| GO_CYCLIN_DEPENDENT_PROTEIN_KINASE_HOLOENZYME_COMPLEX | GO_CYCLIN_DEPENDENT_PROTEIN_KINASE_HOLOENZYME_COMPLEX | Details ... | 42 | 0.6272458 | 2.1212468 | 0 | 0.013661753 | 0.177 | 3608 | tags=52%, list=18%, signal=64% |  |  |  |
| GO_PROTEIN_LOCALIZATION_TO_CHROMOSOME | GO_PROTEIN_LOCALIZATION_TO_CHROMOSOME | Details ... | 81 | 0.6144693 | 2.1182895 | 0 | 0.013827777 | 0.186 | 3344 | tags=63%, list=17%, signal=76% |  |  |  |
| GO_REGULATION_OF_DNA_REPLICATION | GO_REGULATION_OF_DNA_REPLICATION | Details ... | 111 | 0.5771988 | 2.1177678 | 0.002118644 | 0.013345692 | 0.186 | 2729 | tags=51%, list=14%, signal=59% |  |  |  |
| HP_OLIGOHYDRAMNIOS | HP_OLIGOHYDRAMNIOS | Details ... | 115 | 0.51960963 | 2.114504 | 0 | 0.013557511 | 0.189 | 3245 | tags=43%, list=17%, signal=52% |  |  |  |
| GO_REGULATION_OF_DNA_DEPENDENT_DNA_REPLICATION | GO_REGULATION_OF_DNA_DEPENDENT_DNA_REPLICATION | Details ... | 52 | 0.68864226 | 2.1132033 | 0.002040816 | 0.013449756 | 0.192 | 3175 | tags=62%, list=16%, signal=73% |  |  |  |
| GO_RIBONUCLEOPROTEIN_COMPLEX_BINDING | GO_RIBONUCLEOPROTEIN_COMPLEX_BINDING | Details ... | 131 | 0.55289716 | 2.113024 | 0 | 0.013057582 | 0.193 | 3279 | tags=47%, list=17%, signal=56% |  |  |  |
| GO_REGULATION_OF_CELL_CYCLE_G2_M_PHASE_TRANSITION | GO_REGULATION_OF_CELL_CYCLE_G2_M_PHASE_TRANSITION | Details ... | 211 | 0.5361177 | 2.112673 | 0 | 0.012732389 | 0.194 | 3454 | tags=45%, list=18%, signal=55% |  |  |  |
| GO_NUCLEAR_CHROMOSOME_SEGREGATION | GO_NUCLEAR_CHROMOSOME_SEGREGATION | Details ... | 260 | 0.55846214 | 2.1121233 | 0 | 0.012411214 | 0.194 | 3932 | tags=53%, list=20%, signal=66% |  |  |  |
| GO_REGULATION_OF_TRANSLATIONAL_INITIATION | GO_REGULATION_OF_TRANSLATIONAL_INITIATION | Details ... | 76 | 0.5711384 | 2.1118698 | 0 | 0.012114608 | 0.194 | 3568 | tags=58%, list=18%, signal=71% |  |  |  |
| GO_SPINDLE_POLE | GO_SPINDLE_POLE | Details ... | 163 | 0.5234233 | 2.1109345 | 0 | 0.012004015 | 0.197 | 3495 | tags=45%, list=18%, signal=55% |  |  |  |
| HP_PSYCHOMOTOR_RETARDATION | HP_PSYCHOMOTOR_RETARDATION | Details ... | 87 | 0.51974434 | 2.1108644 | 0 | 0.011661043 | 0.197 | 3651 | tags=45%, list=19%, signal=55% |  |  |  |
| GO_DNA_CONFORMATION_CHANGE | GO_DNA_CONFORMATION_CHANGE | Details ... | 275 | 0.5645219 | 2.1102645 | 0 | 0.011402721 | 0.198 | 3803 | tags=49%, list=19%, signal=60% |  |  |  |
| GO_ATPASE_ACTIVITY | GO_ATPASE_ACTIVITY | Details ... | 410 | 0.51236993 | 2.1084106 | 0 | 0.011637693 | 0.201 | 3959 | tags=48%, list=20%, signal=59% |  |  |  |
| GO_GOLGI_VESICLE_BUDDING | GO_GOLGI_VESICLE_BUDDING | Details ... | 80 | 0.548035 | 2.1076624 | 0 | 0.011435572 | 0.204 | 3138 | tags=44%, list=16%, signal=52% |  |  |  |
| HP_LOWER_LIMB_HYPERREFLEXIA | HP_LOWER_LIMB_HYPERREFLEXIA | Details ... | 45 | 0.56925815 | 2.1061044 | 0 | 0.011456114 | 0.208 | 3254 | tags=40%, list=17%, signal=48% |  |  |  |
| GO_MITOTIC_SISTER_CHROMATID_SEGREGATION | GO_MITOTIC_SISTER_CHROMATID_SEGREGATION | Details ... | 158 | 0.60248363 | 2.1052372 | 0.00204918 | 0.011383428 | 0.213 | 3709 | tags=59%, list=19%, signal=72% |  |  |  |
| GO_DNA_DEPENDENT_ATPASE_ACTIVITY | GO_DNA_DEPENDENT_ATPASE_ACTIVITY | Details ... | 106 | 0.6671734 | 2.1050804 | 0 | 0.011105782 | 0.213 | 3514 | tags=62%, list=18%, signal=76% |  |  |  |
| GO_POSITIVE_REGULATION_OF_VIRAL_LIFE_CYCLE | GO_POSITIVE_REGULATION_OF_VIRAL_LIFE_CYCLE | Details ... | 61 | 0.53885645 | 2.1013393 | 0 | 0.011409441 | 0.219 | 2793 | tags=46%, list=14%, signal=53% |  |  |  |
| GO_SPINDLE_ORGANIZATION | GO_SPINDLE_ORGANIZATION | Details ... | 180 | 0.54451394 | 2.1001172 | 0 | 0.011490118 | 0.224 | 3914 | tags=52%, list=20%, signal=65% |  |  |  |
| GO_RETROGRADE_VESICLE_MEDIATED_TRANSPORT_GOLGI_TO_ENDOPLASMIC_RETICULUM | GO_RETROGRADE_VESICLE_MEDIATED_TRANSPORT_GOLGI_TO_ENDOPLASMIC_RETICULUM | Details ... | 87 | 0.53812414 | 2.1000948 | 0 | 0.01122898 | 0.224 | 2908 | tags=44%, list=15%, signal=51% |  |  |  |
| GO_VESICLE_TARGETING_TO_FROM_OR_WITHIN_GOLGI | GO_VESICLE_TARGETING_TO_FROM_OR_WITHIN_GOLGI | Details ... | 73 | 0.5520171 | 2.0979369 | 0 | 0.011366704 | 0.228 | 3138 | tags=44%, list=16%, signal=52% |  |  |  |
| GO_PROTEIN_IMPORT | GO_PROTEIN_IMPORT | Details ... | 199 | 0.4647695 | 2.0969343 | 0 | 0.011234774 | 0.229 | 3753 | tags=42%, list=19%, signal=52% |  |  |  |
| GO_PROTEASOMAL_PROTEIN_CATABOLIC_PROCESS | GO_PROTEASOMAL_PROTEIN_CATABOLIC_PROCESS | Details ... | 474 | 0.43412197 | 2.0962827 | 0 | 0.011153158 | 0.231 | 3766 | tags=41%, list=19%, signal=49% |  |  |  |
| HP_ABNORMAL_GLYCOSYLATION | HP_ABNORMAL_GLYCOSYLATION | Details ... | 37 | 0.6253833 | 2.0954142 | 0 | 0.011151917 | 0.235 | 4676 | tags=73%, list=24%, signal=96% |  |  |  |
| HP_TONGUE_FASCICULATIONS | HP_TONGUE_FASCICULATIONS | Details ... | 19 | 0.7073724 | 2.0878437 | 0 | 0.012672549 | 0.259 | 3008 | tags=42%, list=15%, signal=50% |  |  |  |
| GO_ENDOPLASMIC_RETICULUM_TUBULAR_NETWORK_ORGANIZATION | GO_ENDOPLASMIC_RETICULUM_TUBULAR_NETWORK_ORGANIZATION | Details ... | 18 | 0.78713423 | 2.0878284 | 0 | 0.012419099 | 0.259 | 2356 | tags=67%, list=12%, signal=76% |  |  |  |
| GO_DNA_REPLICATION | GO_DNA_REPLICATION | Details ... | 271 | 0.5595797 | 2.0832396 | 0.002057613 | 0.01304333 | 0.279 | 3274 | tags=49%, list=17%, signal=59% |  |  |  |
| GO_ENDOPLASMIC_RETICULUM_SUBCOMPARTMENT | GO_ENDOPLASMIC_RETICULUM_SUBCOMPARTMENT | Details ... | 26 | 0.6393299 | 2.0813928 | 0 | 0.013201638 | 0.283 | 4384 | tags=46%, list=22%, signal=59% |  |  |  |
| GO_NUCLEOBASE_CONTAINING_COMPOUND_TRANSPORT | GO_NUCLEOBASE_CONTAINING_COMPOUND_TRANSPORT | Details ... | 256 | 0.5002695 | 2.0806599 | 0 | 0.013069074 | 0.284 | 3430 | tags=41%, list=18%, signal=49% |  |  |  |
| GO_HISTONE_UBIQUITINATION | GO_HISTONE_UBIQUITINATION | Details ... | 48 | 0.60039055 | 2.078379 | 0 | 0.013214706 | 0.29 | 3766 | tags=58%, list=19%, signal=72% |  |  |  |
| GO_RNA_DEPENDENT_DNA_BIOSYNTHETIC_PROCESS | GO_RNA_DEPENDENT_DNA_BIOSYNTHETIC_PROCESS | Details ... | 70 | 0.5729951 | 2.0773032 | 0 | 0.013126398 | 0.293 | 3391 | tags=50%, list=17%, signal=60% |  |  |  |
| GO_ER_NUCLEUS_SIGNALING_PATHWAY | GO_ER_NUCLEUS_SIGNALING_PATHWAY | Details ... | 50 | 0.5814325 | 2.077053 | 0 | 0.012961478 | 0.294 | 2787 | tags=46%, list=14%, signal=54% |  |  |  |
| HP_ABNORMAL_GLIAL_CELL_MORPHOLOGY | HP_ABNORMAL_GLIAL_CELL_MORPHOLOGY | Details ... | 130 | 0.47304922 | 2.0751805 | 0 | 0.013164429 | 0.301 | 3732 | tags=45%, list=19%, signal=56% |  |  |  |
| GO_PROTEIN_KINASE_COMPLEX | GO_PROTEIN_KINASE_COMPLEX | Details ... | 102 | 0.5168365 | 2.0747845 | 0 | 0.013031224 | 0.303 | 3608 | tags=39%, list=18%, signal=48% |  |  |  |
| GO_SPINDLE_ASSEMBLY | GO_SPINDLE_ASSEMBLY | Details ... | 113 | 0.5676074 | 2.0742295 | 0 | 0.012999177 | 0.305 | 3914 | tags=58%, list=20%, signal=72% |  |  |  |
| GO_KINETOCHORE | GO_KINETOCHORE | Details ... | 134 | 0.6279471 | 2.0741684 | 0.00209205 | 0.012782524 | 0.305 | 2634 | tags=57%, list=13%, signal=65% |  |  |  |
| GO_RNA_LOCALIZATION | GO_RNA_LOCALIZATION |  | 232 | 0.53333837 | 2.073 | 0 | 0.012727698 | 0.309 | 3430 | tags=44%, list=18%, signal=53% |  |  |  |
| GO_POSITIVE_REGULATION_OF_CELL_CYCLE_G2_M_PHASE_TRANSITION | GO_POSITIVE_REGULATION_OF_CELL_CYCLE_G2_M_PHASE_TRANSITION |  | 30 | 0.67361546 | 2.0711737 | 0 | 0.012949353 | 0.313 | 3447 | tags=57%, list=18%, signal=69% |  |  |  |
| GO_TRANSFERASE_COMPLEX_TRANSFERRING_PHOSPHORUS_CONTAINING_GROUPS | GO_TRANSFERASE_COMPLEX_TRANSFERRING_PHOSPHORUS_CONTAINING_GROUPS |  | 246 | 0.46917853 | 2.0711615 | 0 | 0.012743807 | 0.313 | 3640 | tags=41%, list=19%, signal=49% |  |  |  |
| GO_REGULATION_OF_CHROMOSOME_SEGREGATION | GO_REGULATION_OF_CHROMOSOME_SEGREGATION |  | 101 | 0.6215328 | 2.0703843 | 0 | 0.01261841 | 0.315 | 3920 | tags=63%, list=20%, signal=79% |  |  |  |
| GO_COPII_COATED_VESICLE_BUDDING | GO_COPII_COATED_VESICLE_BUDDING |  | 72 | 0.54011333 | 2.0699701 | 0 | 0.012480213 | 0.315 | 3138 | tags=44%, list=16%, signal=53% |  |  |  |
| GO_RECOMBINATIONAL_REPAIR | GO_RECOMBINATIONAL_REPAIR |  | 136 | 0.57399285 | 2.0699444 | 0 | 0.012305958 | 0.316 | 3257 | tags=54%, list=17%, signal=64% |  |  |  |
| HP_CHROMOSOME_BREAKAGE | HP_CHROMOSOME_BREAKAGE |  | 26 | 0.7492271 | 2.0689027 | 0 | 0.012263523 | 0.317 | 1714 | tags=54%, list=9%, signal=59% |  |  |  |
| GO_PROTEASOME_ACCESSORY_COMPLEX | GO_PROTEASOME_ACCESSORY_COMPLEX |  | 24 | 0.7842404 | 2.068072 | 0 | 0.012183729 | 0.317 | 1774 | tags=54%, list=9%, signal=60% |  |  |  |
| GO_ORGANELLE_FISSION | GO_ORGANELLE_FISSION |  | 457 | 0.48734203 | 2.0678186 | 0 | 0.01207568 | 0.317 | 3718 | tags=43%, list=19%, signal=52% |  |  |  |
| GO_TELOMERE_ORGANIZATION | GO_TELOMERE_ORGANIZATION |  | 146 | 0.5572589 | 2.0668082 | 0 | 0.012126522 | 0.324 | 3456 | tags=51%, list=18%, signal=61% |  |  |  |
| GO_REGULATION_OF_MRNA_CATABOLIC_PROCESS | GO_REGULATION_OF_MRNA_CATABOLIC_PROCESS |  | 204 | 0.48267156 | 2.0657747 | 0 | 0.012166886 | 0.328 | 3787 | tags=41%, list=19%, signal=51% |  |  |  |
| GO_MITOTIC_SISTER_CHROMATID_COHESION | GO_MITOTIC_SISTER_CHROMATID_COHESION |  | 25 | 0.7232149 | 2.0655866 | 0 | 0.012028873 | 0.328 | 2970 | tags=68%, list=15%, signal=80% |  |  |  |
| GO_SPINDLE | GO_SPINDLE |  | 360 | 0.48216677 | 2.0649042 | 0 | 0.011947288 | 0.33 | 3719 | tags=43%, list=19%, signal=52% |  |  |  |
| GO_NCRNA_EXPORT_FROM_NUCLEUS | GO_NCRNA_EXPORT_FROM_NUCLEUS |  | 37 | 0.7572679 | 2.0640447 | 0 | 0.01205719 | 0.33 | 3430 | tags=78%, list=18%, signal=95% |  |  |  |
| GO_PROTEIN_K48_LINKED_UBIQUITINATION | GO_PROTEIN_K48_LINKED_UBIQUITINATION |  | 56 | 0.5708203 | 2.063896 | 0 | 0.011956221 | 0.331 | 4321 | tags=57%, list=22%, signal=73% |  |  |  |
| GO_GOLGI_VESICLE_TRANSPORT | GO_GOLGI_VESICLE_TRANSPORT |  | 370 | 0.4502056 | 2.063884 | 0 | 0.011798902 | 0.331 | 3151 | tags=39%, list=16%, signal=45% |  |  |  |
| GO_TELOMERE_MAINTENANCE_VIA_TELOMERE_LENGTHENING | GO_TELOMERE_MAINTENANCE_VIA_TELOMERE_LENGTHENING |  | 80 | 0.56509507 | 2.063679 | 0 | 0.011686817 | 0.331 | 3391 | tags=50%, list=17%, signal=60% |  |  |  |
| GO_PROTEIN_POLYUBIQUITINATION | GO_PROTEIN_POLYUBIQUITINATION |  | 330 | 0.44510475 | 2.0607975 | 0 | 0.012099375 | 0.338 | 4486 | tags=45%, list=23%, signal=58% |  |  |  |
| GO_PROTEASOME_BINDING | GO_PROTEASOME_BINDING |  | 17 | 0.72211355 | 2.0606754 | 0.001956947 | 0.011946218 | 0.338 | 2039 | tags=53%, list=10%, signal=59% |  |  |  |
| GO_MITOTIC_CYTOKINESIS | GO_MITOTIC_CYTOKINESIS |  | 71 | 0.5539908 | 2.059854 | 0 | 0.011926455 | 0.338 | 2331 | tags=45%, list=12%, signal=51% |  |  |  |
| GO_DNA_DOUBLE_STRAND_BREAK_PROCESSING | GO_DNA_DOUBLE_STRAND_BREAK_PROCESSING |  | 25 | 0.71414024 | 2.0587401 | 0 | 0.011915709 | 0.341 | 2313 | tags=56%, list=12%, signal=63% |  |  |  |
| GO_ESTABLISHMENT_OF_RNA_LOCALIZATION | GO_ESTABLISHMENT_OF_RNA_LOCALIZATION |  | 199 | 0.5310438 | 2.0576508 | 0 | 0.011985351 | 0.342 | 3430 | tags=44%, list=18%, signal=52% |  |  |  |
| GO_UBIQUITIN_LIKE_PROTEIN_CONJUGATING_ENZYME_ACTIVITY | GO_UBIQUITIN_LIKE_PROTEIN_CONJUGATING_ENZYME_ACTIVITY |  | 42 | 0.62171406 | 2.057143 | 0.002123142 | 0.011936965 | 0.345 | 4907 | tags=64%, list=25%, signal=86% |  |  |  |
| GO_SINGLE_STRANDED_DNA_BINDING | GO_SINGLE_STRANDED_DNA_BINDING |  | 108 | 0.5827075 | 2.056078 | 0 | 0.011957825 | 0.347 | 3257 | tags=47%, list=17%, signal=56% |  |  |  |
| GO_DNA_DEPENDENT_DNA_REPLICATION | GO_DNA_DEPENDENT_DNA_REPLICATION |  | 151 | 0.6298116 | 2.05593 | 0 | 0.011841469 | 0.347 | 3274 | tags=56%, list=17%, signal=67% |  |  |  |
| HP_BREAST_CARCINOMA | HP_BREAST_CARCINOMA |  | 60 | 0.5888006 | 2.055794 | 0 | 0.011712479 | 0.347 | 3019 | tags=52%, list=15%, signal=61% |  |  |  |
| GO_CONDENSED_CHROMOSOME | GO_CONDENSED_CHROMOSOME |  | 211 | 0.5718129 | 2.0551555 | 0 | 0.011655159 | 0.351 | 3709 | tags=53%, list=19%, signal=64% |  |  |  |
| GO_CELL_CYCLE_G2_M_PHASE_TRANSITION | GO_CELL_CYCLE_G2_M_PHASE_TRANSITION |  | 266 | 0.49769104 | 2.0545747 | 0 | 0.011658549 | 0.354 | 3454 | tags=43%, list=18%, signal=52% |  |  |  |
| GO_DOUBLE_STRANDED_RNA_BINDING | GO_DOUBLE_STRANDED_RNA_BINDING |  | 76 | 0.59049606 | 2.0543473 | 0 | 0.011547052 | 0.355 | 2905 | tags=49%, list=15%, signal=57% |  |  |  |
| GO_CHROMOSOME_TELOMERIC_REGION | GO_CHROMOSOME_TELOMERIC_REGION |  | 139 | 0.5474842 | 2.05272 | 0 | 0.011627027 | 0.36 | 3803 | tags=51%, list=19%, signal=63% |  |  |  |
| HP_POSTNATAL_MICROCEPHALY | HP_POSTNATAL_MICROCEPHALY |  | 130 | 0.48132282 | 2.0496473 | 0 | 0.012053408 | 0.369 | 4612 | tags=50%, list=24%, signal=65% |  |  |  |
| GO_DOUBLE_STRAND_BREAK_REPAIR | GO_DOUBLE_STRAND_BREAK_REPAIR |  | 243 | 0.5233581 | 2.049316 | 0 | 0.011940027 | 0.37 | 3968 | tags=54%, list=20%, signal=67% |  |  |  |
| GO_CHROMOSOME_ORGANIZATION_INVOLVED_IN_MEIOTIC_CELL_CYCLE | GO_CHROMOSOME_ORGANIZATION_INVOLVED_IN_MEIOTIC_CELL_CYCLE |  | 68 | 0.57417476 | 2.047411 | 0 | 0.01215282 | 0.373 | 1663 | tags=32%, list=9%, signal=35% |  |  |  |
| GO_PROTEIN_MODIFICATION_BY_SMALL_PROTEIN_REMOVAL | GO_PROTEIN_MODIFICATION_BY_SMALL_PROTEIN_REMOVAL |  | 288 | 0.4572735 | 2.0438185 | 0 | 0.012679618 | 0.386 | 3936 | tags=43%, list=20%, signal=53% |  |  |  |
| GO_TRNA_TRANSPORT | GO_TRNA_TRANSPORT |  | 36 | 0.7520604 | 2.0412803 | 0.002008032 | 0.012993005 | 0.395 | 3430 | tags=75%, list=18%, signal=91% |  |  |  |
| KEGG_OOCYTE_MEIOSIS | KEGG_OOCYTE_MEIOSIS |  | 112 | 0.50707763 | 2.0409675 | 0 | 0.012909704 | 0.396 | 3052 | tags=41%, list=16%, signal=48% |  |  |  |
| HP_SLOPING_FOREHEAD | HP_SLOPING_FOREHEAD |  | 150 | 0.54221886 | 2.0390525 | 0 | 0.013166326 | 0.405 | 4073 | tags=53%, list=21%, signal=66% |  |  |  |
| HP_CAFE_AU_LAIT_SPOT | HP_CAFE_AU_LAIT_SPOT |  | 105 | 0.58145714 | 2.03885 | 0 | 0.013092817 | 0.406 | 3162 | tags=53%, list=16%, signal=63% |  |  |  |
| GO_FEMALE_MEIOTIC_NUCLEAR_DIVISION | GO_FEMALE_MEIOTIC_NUCLEAR_DIVISION |  | 31 | 0.63936377 | 2.0369494 | 0 | 0.013324426 | 0.408 | 3363 | tags=48%, list=17%, signal=58% |  |  |  |
| GO_NUCLEAR_IMPORT_SIGNAL_RECEPTOR_ACTIVITY | GO_NUCLEAR_IMPORT_SIGNAL_RECEPTOR_ACTIVITY |  | 20 | 0.73756623 | 2.0366213 | 0 | 0.01326396 | 0.408 | 1699 | tags=50%, list=9%, signal=55% |  |  |  |
| GO_CYTOSKELETON_DEPENDENT_CYTOKINESIS | GO_CYTOSKELETON_DEPENDENT_CYTOKINESIS |  | 87 | 0.5267739 | 2.0355353 | 0 | 0.013286806 | 0.415 | 2058 | tags=41%, list=11%, signal=46% |  |  |  |
| GO_HELICASE_ACTIVITY | GO_HELICASE_ACTIVITY |  | 158 | 0.59882885 | 2.0347543 | 0.00203666 | 0.01326943 | 0.416 | 3959 | tags=55%, list=20%, signal=69% |  |  |  |
| GO_MITOTIC_NUCLEAR_DIVISION | GO_MITOTIC_NUCLEAR_DIVISION |  | 274 | 0.52214783 | 2.0325122 | 0 | 0.01349247 | 0.424 | 3718 | tags=51%, list=19%, signal=62% |  |  |  |
| GO_VESICLE_BUDDING_FROM_MEMBRANE | GO_VESICLE_BUDDING_FROM_MEMBRANE |  | 108 | 0.48920408 | 2.0324907 | 0.00203666 | 0.013389891 | 0.424 | 3138 | tags=40%, list=16%, signal=47% |  |  |  |
| GO_REGULATION_OF_CENTROSOME_CYCLE | GO_REGULATION_OF_CENTROSOME_CYCLE |  | 59 | 0.5862513 | 2.0314658 | 0 | 0.013397148 | 0.427 | 3462 | tags=51%, list=18%, signal=62% |  |  |  |
| GO_RNA_EXPORT_FROM_NUCLEUS | GO_RNA_EXPORT_FROM_NUCLEUS |  | 141 | 0.5754418 | 2.031237 | 0.002016129 | 0.013328884 | 0.428 | 3530 | tags=50%, list=18%, signal=61% |  |  |  |
| HP_SIDEROBLASTIC_ANEMIA | HP_SIDEROBLASTIC_ANEMIA |  | 33 | 0.7231562 | 2.0311463 | 0 | 0.013211324 | 0.428 | 3125 | tags=76%, list=16%, signal=90% |  |  |  |
| GO_PROTEIN_CONTAINING_COMPLEX_LOCALIZATION | GO_PROTEIN_CONTAINING_COMPLEX_LOCALIZATION |  | 286 | 0.47747162 | 2.0309405 | 0 | 0.013122411 | 0.428 | 3530 | tags=39%, list=18%, signal=47% |  |  |  |
| GO_POSTREPLICATION_REPAIR | GO_POSTREPLICATION_REPAIR |  | 52 | 0.5842006 | 2.030203 | 0 | 0.013150734 | 0.433 | 4420 | tags=63%, list=23%, signal=82% |  |  |  |
| GO_REGULATION_OF_MITOTIC_SPINDLE_ASSEMBLY | GO_REGULATION_OF_MITOTIC_SPINDLE_ASSEMBLY |  | 17 | 0.7415697 | 2.0260568 | 0 | 0.013868699 | 0.445 | 2712 | tags=65%, list=14%, signal=75% |  |  |  |
| HP_TETRAPLEGIA_TETRAPARESIS | HP_TETRAPLEGIA_TETRAPARESIS |  | 62 | 0.48574147 | 2.024261 | 0 | 0.014124631 | 0.455 | 3228 | tags=42%, list=17%, signal=50% |  |  |  |
| GO_REGULATION_OF_TRANSLATION_IN_RESPONSE_TO_STRESS | GO_REGULATION_OF_TRANSLATION_IN_RESPONSE_TO_STRESS |  | 20 | 0.68232226 | 2.0206575 | 0 | 0.014712847 | 0.466 | 2684 | tags=60%, list=14%, signal=69% |  |  |  |
| GO_SCF_DEPENDENT_PROTEASOMAL_UBIQUITIN_DEPENDENT_PROTEIN_CATABOLIC_PROCESS | GO_SCF_DEPENDENT_PROTEASOMAL_UBIQUITIN_DEPENDENT_PROTEIN_CATABOLIC_PROCESS |  | 92 | 0.534165 | 2.0205777 | 0 | 0.014609076 | 0.466 | 3454 | tags=45%, list=18%, signal=54% |  |  |  |
| GO_ENDOPLASMIC_RETICULUM_TO_GOLGI_VESICLE_MEDIATED_TRANSPORT | GO_ENDOPLASMIC_RETICULUM_TO_GOLGI_VESICLE_MEDIATED_TRANSPORT |  | 203 | 0.46428144 | 2.0203307 | 0.002008032 | 0.014506293 | 0.467 | 3138 | tags=38%, list=16%, signal=45% |  |  |  |
| GO_ESTABLISHMENT_OF_ORGANELLE_LOCALIZATION | GO_ESTABLISHMENT_OF_ORGANELLE_LOCALIZATION |  | 426 | 0.41617045 | 2.0179174 | 0 | 0.014856436 | 0.474 | 3943 | tags=42%, list=20%, signal=52% |  |  |  |
| GO_SITE_OF_DNA_DAMAGE | GO_SITE_OF_DNA_DAMAGE |  | 79 | 0.5674402 | 2.0177462 | 0 | 0.01476338 | 0.475 | 4126 | tags=59%, list=21%, signal=75% |  |  |  |
| HP_ABNORMALITY_OF_CHROMOSOME_STABILITY | HP_ABNORMALITY_OF_CHROMOSOME_STABILITY |  | 47 | 0.6728539 | 2.0169494 | 0.002109705 | 0.014754571 | 0.477 | 3125 | tags=60%, list=16%, signal=71% |  |  |  |
| GO_RESPONSE_TO_HEAT | GO_RESPONSE_TO_HEAT |  | 161 | 0.46635243 | 2.0162 | 0.002066116 | 0.014725907 | 0.479 | 3440 | tags=42%, list=18%, signal=50% |  |  |  |
| GO_POSITIVE_REGULATION_OF_CHROMOSOME_SEGREGATION | GO_POSITIVE_REGULATION_OF_CHROMOSOME_SEGREGATION |  | 32 | 0.6718027 | 2.0158892 | 0 | 0.014672807 | 0.483 | 3541 | tags=66%, list=18%, signal=80% |  |  |  |
| HP_INTRAUTERINE_GROWTH_RETARDATION | HP_INTRAUTERINE_GROWTH_RETARDATION |  | 448 | 0.43052807 | 2.0129807 | 0 | 0.015055458 | 0.491 | 3701 | tags=38%, list=19%, signal=46% |  |  |  |
| HP_ABNORMAL_FALLOPIAN_TUBE_MORPHOLOGY | HP_ABNORMAL_FALLOPIAN_TUBE_MORPHOLOGY |  | 27 | 0.65132326 | 2.0124137 | 0 | 0.015028716 | 0.492 | 3252 | tags=70%, list=17%, signal=84% |  |  |  |
| GO_REGULATION_OF_SISTER_CHROMATID_SEGREGATION | GO_REGULATION_OF_SISTER_CHROMATID_SEGREGATION |  | 81 | 0.5928413 | 2.0121572 | 0 | 0.014972337 | 0.493 | 3867 | tags=59%, list=20%, signal=74% |  |  |  |
| HP_ABNORMALITY_OF_THE_ENDOMETRIUM | HP_ABNORMALITY_OF_THE_ENDOMETRIUM |  | 25 | 0.6306896 | 2.0101767 | 0 | 0.015221094 | 0.5 | 2788 | tags=52%, list=14%, signal=61% |  |  |  |
| GO_PROTEIN_TRANSMEMBRANE_TRANSPORT | GO_PROTEIN_TRANSMEMBRANE_TRANSPORT |  | 60 | 0.54324645 | 2.0098577 | 0 | 0.015142239 | 0.5 | 3539 | tags=45%, list=18%, signal=55% |  |  |  |
| GO_PROTEIN_TARGETING_TO_LYSOSOME | GO_PROTEIN_TARGETING_TO_LYSOSOME |  | 21 | 0.68750495 | 2.0087714 | 0 | 0.015264508 | 0.504 | 3332 | tags=62%, list=17%, signal=75% |  |  |  |
| GO_REGULATION_OF_TELOMERE_MAINTENANCE_VIA_TELOMERE_LENGTHENING | GO_REGULATION_OF_TELOMERE_MAINTENANCE_VIA_TELOMERE_LENGTHENING |  | 61 | 0.5777221 | 2.0070674 | 0 | 0.015401839 | 0.506 | 3391 | tags=52%, list=17%, signal=63% |  |  |  |
| GO_CELLULAR_PROTEIN_CONTAINING_COMPLEX_LOCALIZATION | GO_CELLULAR_PROTEIN_CONTAINING_COMPLEX_LOCALIZATION |  | 25 | 0.6205811 | 2.0066395 | 0.002061856 | 0.015400774 | 0.508 | 2366 | tags=40%, list=12%, signal=45% |  |  |  |
| GO_PROTEIN_MONOUBIQUITINATION | GO_PROTEIN_MONOUBIQUITINATION |  | 67 | 0.5593835 | 2.0064538 | 0.00203252 | 0.015301548 | 0.509 | 3309 | tags=55%, list=17%, signal=66% |  |  |  |
| HP_ASTROCYTOMA | HP_ASTROCYTOMA |  | 18 | 0.73134553 | 2.0060785 | 0 | 0.015257423 | 0.509 | 3422 | tags=61%, list=18%, signal=74% |  |  |  |
| HP_ABNORMAL_PROTEIN_GLYCOSYLATION | HP_ABNORMAL_PROTEIN_GLYCOSYLATION |  | 31 | 0.62264174 | 2.0048258 | 0 | 0.01538528 | 0.511 | 4676 | tags=74%, list=24%, signal=97% |  |  |  |
| GO_CONDENSED_CHROMOSOME_CENTROMERIC_REGION | GO_CONDENSED_CHROMOSOME_CENTROMERIC_REGION |  | 119 | 0.6128706 | 2.0031857 | 0.002066116 | 0.015705507 | 0.52 | 3709 | tags=63%, list=19%, signal=77% |  |  |  |
| HP_RELATIVE_MACROCEPHALY | HP_RELATIVE_MACROCEPHALY |  | 33 | 0.61935824 | 2.0027406 | 0 | 0.01569393 | 0.521 | 1856 | tags=42%, list=10%, signal=47% |  |  |  |
| HP_PROGRESSIVE_SPASTICITY | HP_PROGRESSIVE_SPASTICITY |  | 23 | 0.61368436 | 2.002067 | 0 | 0.015704297 | 0.525 | 851 | tags=26%, list=4%, signal=27% |  |  |  |
| HP_MACROTIA | HP_MACROTIA |  | 194 | 0.4623203 | 2.0011673 | 0 | 0.015805624 | 0.528 | 3701 | tags=43%, list=19%, signal=52% |  |  |  |
| GO_ENDOPEPTIDASE_COMPLEX | GO_ENDOPEPTIDASE_COMPLEX |  | 69 | 0.6310701 | 2.0011077 | 0.001996008 | 0.015709538 | 0.528 | 3454 | tags=52%, list=18%, signal=63% |  |  |  |
| GO_REGULATION_OF_DNA_METHYLATION | GO_REGULATION_OF_DNA_METHYLATION |  | 23 | 0.61735404 | 2.0009272 | 0 | 0.015621533 | 0.528 | 2311 | tags=39%, list=12%, signal=44% |  |  |  |
| GO_REGULATION_OF_TELOMERE_MAINTENANCE | GO_REGULATION_OF_TELOMERE_MAINTENANCE |  | 80 | 0.55114746 | 2.0009036 | 0 | 0.015513196 | 0.528 | 3391 | tags=51%, list=17%, signal=62% |  |  |  |
| GO_VESICLE_COAT | GO_VESICLE_COAT |  | 55 | 0.546908 | 1.9978933 | 0.001956947 | 0.016002048 | 0.538 | 3015 | tags=47%, list=15%, signal=56% |  |  |  |
| GO_NUCLEOBASE_BIOSYNTHETIC_PROCESS | GO_NUCLEOBASE_BIOSYNTHETIC_PROCESS |  | 18 | 0.7529424 | 1.9973868 | 0 | 0.016004084 | 0.54 | 2500 | tags=61%, list=13%, signal=70% |  |  |  |
| GO_ATTACHMENT_OF_SPINDLE_MICROTUBULES_TO_KINETOCHORE | GO_ATTACHMENT_OF_SPINDLE_MICROTUBULES_TO_KINETOCHORE |  | 34 | 0.691669 | 1.9967221 | 0.006147541 | 0.015991086 | 0.541 | 3920 | tags=74%, list=20%, signal=92% |  |  |  |
| KEGG_MISMATCH_REPAIR | KEGG_MISMATCH_REPAIR |  | 23 | 0.7341393 | 1.9951389 | 0 | 0.016235152 | 0.549 | 1663 | tags=52%, list=9%, signal=57% |  |  |  |
| GO_NUCLEAR_TRANSPORT | GO_NUCLEAR_TRANSPORT |  | 353 | 0.465833 | 1.9949242 | 0 | 0.01613961 | 0.55 | 3773 | tags=41%, list=19%, signal=50% |  |  |  |
| HP_MYELODYSPLASIA | HP_MYELODYSPLASIA |  | 71 | 0.57483816 | 1.9938313 | 0 | 0.016269064 | 0.555 | 3949 | tags=58%, list=20%, signal=72% |  |  |  |
| GO_DNA_HELICASE_ACTIVITY | GO_DNA_HELICASE_ACTIVITY |  | 76 | 0.6550479 | 1.993271 | 0 | 0.016282978 | 0.556 | 3514 | tags=59%, list=18%, signal=72% |  |  |  |
| GO_REGULATION_OF_SISTER_CHROMATID_COHESION | GO_REGULATION_OF_SISTER_CHROMATID_COHESION |  | 21 | 0.7153133 | 1.991693 | 0 | 0.016543444 | 0.564 | 3541 | tags=71%, list=18%, signal=87% |  |  |  |
| GO_RIBOSOME_BINDING | GO_RIBOSOME_BINDING |  | 56 | 0.55391574 | 1.9896765 | 0 | 0.016880797 | 0.572 | 2853 | tags=48%, list=15%, signal=56% |  |  |  |
| GO_PEPTIDASE_COMPLEX | GO_PEPTIDASE_COMPLEX |  | 89 | 0.5711842 | 1.98939 | 0.002024292 | 0.016834186 | 0.573 | 3886 | tags=51%, list=20%, signal=63% |  |  |  |
| KEGG_CITRATE_CYCLE_TCA_CYCLE | KEGG_CITRATE_CYCLE_TCA_CYCLE |  | 30 | 0.6841349 | 1.9893754 | 0.002074689 | 0.01672044 | 0.573 | 3422 | tags=63%, list=18%, signal=77% |  |  |  |
| HP_NEOPLASM_OF_THE_BREAST | HP_NEOPLASM_OF_THE_BREAST |  | 81 | 0.5045114 | 1.9892517 | 0 | 0.016631719 | 0.574 | 3019 | tags=43%, list=15%, signal=51% |  |  |  |
| HP_CELLULAR_IMMUNODEFICIENCY | HP_CELLULAR_IMMUNODEFICIENCY |  | 31 | 0.57048666 | 1.9892068 | 0 | 0.016520841 | 0.574 | 3084 | tags=39%, list=16%, signal=46% |  |  |  |
| GO_NUCLEOTIDYLTRANSFERASE_ACTIVITY | GO_NUCLEOTIDYLTRANSFERASE_ACTIVITY |  | 124 | 0.48430222 | 1.9880807 | 0 | 0.016575761 | 0.578 | 3239 | tags=42%, list=17%, signal=50% |  |  |  |
| HP_ABNORMALITY_OF_THE_PREPUTIUM | HP_ABNORMALITY_OF_THE_PREPUTIUM |  | 28 | 0.73898387 | 1.9879768 | 0 | 0.016511241 | 0.579 | 3125 | tags=75%, list=16%, signal=89% |  |  |  |
| GO_NUCLEAR_ENVELOPE | GO_NUCLEAR_ENVELOPE |  | 465 | 0.4031253 | 1.986638 | 0 | 0.016696272 | 0.586 | 3940 | tags=36%, list=20%, signal=44% |  |  |  |
| GO_ORGANELLE_MEMBRANE_CONTACT_SITE | GO_ORGANELLE_MEMBRANE_CONTACT_SITE |  | 26 | 0.62329304 | 1.9842068 | 0 | 0.017134523 | 0.597 | 3531 | tags=50%, list=18%, signal=61% |  |  |  |
| GO_NUCLEOCYTOPLASMIC_CARRIER_ACTIVITY | GO_NUCLEOCYTOPLASMIC_CARRIER_ACTIVITY |  | 30 | 0.7015445 | 1.98374 | 0 | 0.017113889 | 0.598 | 4234 | tags=70%, list=22%, signal=89% |  |  |  |
| GO_MICROTUBULE_ORGANIZING_CENTER_LOCALIZATION | GO_MICROTUBULE_ORGANIZING_CENTER_LOCALIZATION |  | 31 | 0.6376746 | 1.9833685 | 0 | 0.017103523 | 0.601 | 2385 | tags=48%, list=12%, signal=55% |  |  |  |
| GO_REPLICATION_FORK | GO_REPLICATION_FORK |  | 68 | 0.6417168 | 1.978921 | 0.002074689 | 0.017964883 | 0.608 | 4420 | tags=74%, list=23%, signal=95% |  |  |  |
| GO_ENDOPLASMIC_RETICULUM_TUBULAR_NETWORK | GO_ENDOPLASMIC_RETICULUM_TUBULAR_NETWORK |  | 22 | 0.6584805 | 1.9785788 | 0.002020202 | 0.017970225 | 0.612 | 4384 | tags=55%, list=22%, signal=70% |  |  |  |
| HP_EPIPHYSEAL_STIPPLING | HP_EPIPHYSEAL_STIPPLING |  | 31 | 0.5756016 | 1.9778512 | 0 | 0.017986074 | 0.615 | 3831 | tags=58%, list=20%, signal=72% |  |  |  |
| GO_NEGATIVE_REGULATION_OF_CELL_CYCLE_PROCESS | GO_NEGATIVE_REGULATION_OF_CELL_CYCLE_PROCESS |  | 327 | 0.43969214 | 1.9775223 | 0.002074689 | 0.017981796 | 0.615 | 3628 | tags=41%, list=19%, signal=49% |  |  |  |
| GO_NEGATIVE_REGULATION_OF_CELL_CYCLE_G2_M_PHASE_TRANSITION | GO_NEGATIVE_REGULATION_OF_CELL_CYCLE_G2_M_PHASE_TRANSITION |  | 103 | 0.5372898 | 1.9769162 | 0.002057613 | 0.018018626 | 0.616 | 2769 | tags=44%, list=14%, signal=51% |  |  |  |
| GO_OLIGOSACCHARIDE_LIPID_INTERMEDIATE_BIOSYNTHETIC_PROCESS | GO_OLIGOSACCHARIDE_LIPID_INTERMEDIATE_BIOSYNTHETIC_PROCESS |  | 21 | 0.6689653 | 1.9758697 | 0.001992032 | 0.018133283 | 0.621 | 4544 | tags=71%, list=23%, signal=93% |  |  |  |
| GO_POSITIVE_REGULATION_OF_MITOTIC_SISTER_CHROMATID_SEGREGATION | GO_POSITIVE_REGULATION_OF_MITOTIC_SISTER_CHROMATID_SEGREGATION |  | 21 | 0.71091175 | 1.9747437 | 0 | 0.01827373 | 0.625 | 3541 | tags=71%, list=18%, signal=87% |  |  |  |
| GO_NUCLEAR_EXPORT | GO_NUCLEAR_EXPORT |  | 201 | 0.5104339 | 1.9741437 | 0 | 0.018397024 | 0.627 | 3430 | tags=44%, list=18%, signal=53% |  |  |  |
| GO_RAN_GTPASE_BINDING | GO_RAN_GTPASE_BINDING |  | 41 | 0.59116983 | 1.9741204 | 0 | 0.018290251 | 0.627 | 2789 | tags=44%, list=14%, signal=51% |  |  |  |
| GO_ESTABLISHMENT_OF_PROTEIN_LOCALIZATION_TO_TELOMERE | GO_ESTABLISHMENT_OF_PROTEIN_LOCALIZATION_TO_TELOMERE |  | 18 | 0.75433004 | 1.9735042 | 0 | 0.018304024 | 0.633 | 3344 | tags=78%, list=17%, signal=94% |  |  |  |
| HP_MULTIPLE_CAFE_AU_LAIT_SPOTS | HP_MULTIPLE_CAFE_AU_LAIT_SPOTS |  | 59 | 0.57515377 | 1.9720894 | 0 | 0.01851113 | 0.639 | 4125 | tags=63%, list=21%, signal=79% |  |  |  |
| GO_REGULATION_OF_GENE_SILENCING | GO_REGULATION_OF_GENE_SILENCING |  | 96 | 0.5369623 | 1.9696637 | 0 | 0.018968675 | 0.65 | 3430 | tags=53%, list=18%, signal=64% |  |  |  |
| GO_DNA_STRAND_ELONGATION | GO_DNA_STRAND_ELONGATION |  | 26 | 0.7797629 | 1.9690291 | 0.00625 | 0.01901481 | 0.653 | 2471 | tags=69%, list=13%, signal=79% |  |  |  |
| GO_NEGATIVE_REGULATION_OF_DNA_DEPENDENT_DNA_REPLICATION | GO_NEGATIVE_REGULATION_OF_DNA_DEPENDENT_DNA_REPLICATION |  | 19 | 0.7326756 | 1.9676876 | 0.002061856 | 0.019254062 | 0.658 | 2729 | tags=63%, list=14%, signal=73% |  |  |  |
| GO_MAGNESIUM_ION_TRANSPORT | GO_MAGNESIUM_ION_TRANSPORT |  | 17 | 0.75027364 | 1.9659926 | 0 | 0.0196487 | 0.669 | 2052 | tags=41%, list=11%, signal=46% |  |  |  |
| HP_SHORT_PALPEBRAL_FISSURE | HP_SHORT_PALPEBRAL_FISSURE |  | 85 | 0.53731155 | 1.9653836 | 0 | 0.01966044 | 0.672 | 3864 | tags=51%, list=20%, signal=63% |  |  |  |
| GO_MEIOTIC_CELL_CYCLE | GO_MEIOTIC_CELL_CYCLE |  | 248 | 0.47922635 | 1.964014 | 0 | 0.019863881 | 0.678 | 3078 | tags=35%, list=16%, signal=41% |  |  |  |
| GO_NEGATIVE_REGULATION_OF_DNA_RECOMBINATION | GO_NEGATIVE_REGULATION_OF_DNA_RECOMBINATION |  | 33 | 0.6355755 | 1.9638692 | 0.002136752 | 0.01978319 | 0.679 | 3893 | tags=64%, list=20%, signal=79% |  |  |  |
| GO_HISTONE_MRNA_METABOLIC_PROCESS | GO_HISTONE_MRNA_METABOLIC_PROCESS |  | 25 | 0.64323705 | 1.9637426 | 0 | 0.019711945 | 0.679 | 4134 | tags=60%, list=21%, signal=76% |  |  |  |
| HP_CEREBRAL_HYPOPLASIA | HP_CEREBRAL_HYPOPLASIA |  | 40 | 0.6238586 | 1.962319 | 0.00203252 | 0.019889228 | 0.682 | 5903 | tags=73%, list=30%, signal=104% |  |  |  |
| HP_CLUBBING_OF_TOES | HP_CLUBBING_OF_TOES |  | 29 | 0.6946302 | 1.9613249 | 0 | 0.020141996 | 0.689 | 3125 | tags=66%, list=16%, signal=78% |  |  |  |
| GO_PROTEIN_SUMOYLATION | GO_PROTEIN_SUMOYLATION |  | 80 | 0.553464 | 1.9598941 | 0.001996008 | 0.020387843 | 0.689 | 4006 | tags=58%, list=21%, signal=72% |  |  |  |
| GO_MACROAUTOPHAGY | GO_MACROAUTOPHAGY |  | 304 | 0.42948854 | 1.9594688 | 0 | 0.020364886 | 0.692 | 4251 | tags=42%, list=22%, signal=53% |  |  |  |
| GO_DNA_INTEGRITY_CHECKPOINT | GO_DNA_INTEGRITY_CHECKPOINT |  | 158 | 0.50190127 | 1.9586378 | 0.004 | 0.020443039 | 0.696 | 2694 | tags=45%, list=14%, signal=52% |  |  |  |
| GO_DNA_SYNTHESIS_INVOLVED_IN_DNA_REPAIR | GO_DNA_SYNTHESIS_INVOLVED_IN_DNA_REPAIR |  | 51 | 0.55143493 | 1.9584689 | 0.002070393 | 0.020393783 | 0.698 | 4420 | tags=59%, list=23%, signal=76% |  |  |  |
| HP_GLIOMA | HP_GLIOMA |  | 36 | 0.60460466 | 1.9580492 | 0 | 0.020402206 | 0.7 | 2967 | tags=50%, list=15%, signal=59% |  |  |  |
| GO_MAGNESIUM_ION_TRANSMEMBRANE_TRANSPORTER_ACTIVITY | GO_MAGNESIUM_ION_TRANSMEMBRANE_TRANSPORTER_ACTIVITY |  | 16 | 0.75908095 | 1.9575627 | 0 | 0.020422924 | 0.701 | 2052 | tags=44%, list=11%, signal=49% |  |  |  |
| GO_CYTOPLASMIC_MICROTUBULE | GO_CYTOPLASMIC_MICROTUBULE |  | 78 | 0.49957794 | 1.9575397 | 0.00204918 | 0.020311931 | 0.701 | 4435 | tags=47%, list=23%, signal=61% |  |  |  |
| GO_SPINDLE_LOCALIZATION | GO_SPINDLE_LOCALIZATION |  | 49 | 0.5866084 | 1.9570992 | 0 | 0.020288605 | 0.701 | 3790 | tags=57%, list=19%, signal=71% |  |  |  |
| KEGG_PROTEIN_EXPORT | KEGG_PROTEIN_EXPORT |  | 23 | 0.7030395 | 1.9569656 | 0.002070393 | 0.020210775 | 0.701 | 3279 | tags=61%, list=17%, signal=73% |  |  |  |
| KEGG_RNA_DEGRADATION | KEGG_RNA_DEGRADATION |  | 57 | 0.57209 | 1.956771 | 0.002118644 | 0.02014315 | 0.702 | 4938 | tags=61%, list=25%, signal=82% |  |  |  |
| HP_ABNORMAL_ILEUM_MORPHOLOGY | HP_ABNORMAL_ILEUM_MORPHOLOGY |  | 62 | 0.5632437 | 1.9565101 | 0 | 0.02013466 | 0.702 | 3562 | tags=50%, list=18%, signal=61% |  |  |  |
| GO_CHROMOSOME_LOCALIZATION | GO_CHROMOSOME_LOCALIZATION |  | 78 | 0.55364954 | 1.9564415 | 0 | 0.020044127 | 0.702 | 3905 | tags=56%, list=20%, signal=70% |  |  |  |
| GO_UNFOLDED_PROTEIN_BINDING | GO_UNFOLDED_PROTEIN_BINDING |  | 108 | 0.5127673 | 1.9564404 | 0.00209205 | 0.019938633 | 0.702 | 3441 | tags=44%, list=18%, signal=54% |  |  |  |
| GO_REGULATION_OF_DNA_METABOLIC_PROCESS | GO_REGULATION_OF_DNA_METABOLIC_PROCESS |  | 345 | 0.4389643 | 1.9561301 | 0 | 0.01989912 | 0.704 | 3246 | tags=39%, list=17%, signal=46% |  |  |  |
| GO_REGULATION_OF_CELL_CYCLE_PHASE_TRANSITION | GO_REGULATION_OF_CELL_CYCLE_PHASE_TRANSITION |  | 442 | 0.44120392 | 1.956097 | 0 | 0.019799108 | 0.704 | 3628 | tags=40%, list=19%, signal=48% |  |  |  |
| GO_PROTEIN_DNA_COMPLEX_SUBUNIT_ORGANIZATION | GO_PROTEIN_DNA_COMPLEX_SUBUNIT_ORGANIZATION |  | 206 | 0.5040011 | 1.9549567 | 0 | 0.01995273 | 0.705 | 3646 | tags=44%, list=19%, signal=54% |  |  |  |
| GO_RNA_POLYADENYLATION | GO_RNA_POLYADENYLATION |  | 48 | 0.5800223 | 1.9544181 | 0 | 0.01996024 | 0.707 | 3907 | tags=56%, list=20%, signal=70% |  |  |  |
| GO_PORE_COMPLEX_ASSEMBLY | GO_PORE_COMPLEX_ASSEMBLY |  | 20 | 0.6653813 | 1.9540094 | 0 | 0.019964842 | 0.708 | 3430 | tags=70%, list=18%, signal=85% |  |  |  |
| GO_THIOESTER_BIOSYNTHETIC_PROCESS | GO_THIOESTER_BIOSYNTHETIC_PROCESS |  | 53 | 0.53367347 | 1.9532917 | 0 | 0.020041905 | 0.708 | 4458 | tags=53%, list=23%, signal=68% |  |  |  |
| GO_REGULATION_OF_SPINDLE_ORGANIZATION | GO_REGULATION_OF_SPINDLE_ORGANIZATION |  | 38 | 0.593137 | 1.953117 | 0.002123142 | 0.019958947 | 0.708 | 2959 | tags=53%, list=15%, signal=62% |  |  |  |
| HP_THICK_HAIR | HP_THICK_HAIR |  | 16 | 0.757455 | 1.9519662 | 0 | 0.020170813 | 0.713 | 3387 | tags=69%, list=17%, signal=83% |  |  |  |
| KEGG_CELL_CYCLE | KEGG_CELL_CYCLE |  | 124 | 0.56180674 | 1.9501013 | 0.006198347 | 0.020506041 | 0.718 | 3598 | tags=57%, list=18%, signal=70% |  |  |  |
| GO_NUCLEOSIDE_BISPHOSPHATE_BIOSYNTHETIC_PROCESS | GO_NUCLEOSIDE_BISPHOSPHATE_BIOSYNTHETIC_PROCESS |  | 67 | 0.50284934 | 1.9497452 | 0 | 0.020501582 | 0.72 | 4458 | tags=51%, list=23%, signal=66% |  |  |  |
| GO_NEGATIVE_REGULATION_OF_DNA_REPAIR | GO_NEGATIVE_REGULATION_OF_DNA_REPAIR |  | 34 | 0.6011529 | 1.9497386 | 0.001992032 | 0.020399584 | 0.72 | 4109 | tags=62%, list=21%, signal=78% |  |  |  |
| HP_PROGRESSIVE_MICROCEPHALY | HP_PROGRESSIVE_MICROCEPHALY |  | 68 | 0.4900368 | 1.9491162 | 0.001949318 | 0.020418512 | 0.721 | 4612 | tags=51%, list=24%, signal=67% |  |  |  |
| GO_CELL_CYCLE_CHECKPOINT | GO_CELL_CYCLE_CHECKPOINT |  | 216 | 0.4862688 | 1.947709 | 0.004024145 | 0.020700667 | 0.724 | 3709 | tags=50%, list=19%, signal=60% |  |  |  |
| HP_DEEP_PHILTRUM | HP_DEEP_PHILTRUM |  | 42 | 0.5358736 | 1.9459256 | 0.002074689 | 0.021146802 | 0.733 | 4811 | tags=57%, list=25%, signal=76% |  |  |  |
| GO_REGULATION_OF_CENTROSOME_DUPLICATION | GO_REGULATION_OF_CENTROSOME_DUPLICATION |  | 44 | 0.579643 | 1.9444329 | 0 | 0.021466916 | 0.736 | 3337 | tags=50%, list=17%, signal=60% |  |  |  |
| GO_REGULATION_OF_POSTTRANSCRIPTIONAL_GENE_SILENCING | GO_REGULATION_OF_POSTTRANSCRIPTIONAL_GENE_SILENCING |  | 86 | 0.52796525 | 1.943767 | 0.002008032 | 0.021546336 | 0.74 | 3430 | tags=51%, list=18%, signal=62% |  |  |  |
| GO_CELL_CYCLE_DNA_REPLICATION | GO_CELL_CYCLE_DNA_REPLICATION |  | 65 | 0.6714533 | 1.9431632 | 0.002070393 | 0.02161075 | 0.744 | 4428 | tags=71%, list=23%, signal=91% |  |  |  |
| GO_N_TERMINAL_PROTEIN_AMINO_ACID_MODIFICATION | GO_N_TERMINAL_PROTEIN_AMINO_ACID_MODIFICATION |  | 29 | 0.5808071 | 1.9431522 | 0 | 0.021510748 | 0.744 | 2648 | tags=41%, list=14%, signal=48% |  |  |  |
| GO_DNA_REPLICATION_INDEPENDENT_NUCLEOSOME_ORGANIZATION | GO_DNA_REPLICATION_INDEPENDENT_NUCLEOSOME_ORGANIZATION |  | 38 | 0.6955018 | 1.9429368 | 0 | 0.021454027 | 0.744 | 3601 | tags=68%, list=18%, signal=84% |  |  |  |
| GO_REGULATION_OF_MRNA_3_END_PROCESSING | GO_REGULATION_OF_MRNA_3_END_PROCESSING |  | 28 | 0.6343002 | 1.9419259 | 0 | 0.021592163 | 0.75 | 3026 | tags=61%, list=16%, signal=72% |  |  |  |
| HP_BICORNUATE_UTERUS | HP_BICORNUATE_UTERUS |  | 43 | 0.59949493 | 1.9409404 | 0 | 0.02172761 | 0.753 | 4018 | tags=58%, list=21%, signal=73% |  |  |  |
[truncated: 754,852 more chars]
